# Structural and functional characterization of *peste des petits ruminants* virus coded hemagglutinin protein using various *in-silico* approaches

**DOI:** 10.3389/fmicb.2024.1427606

**Published:** 2024-06-20

**Authors:** Sharad Kumar Gaur, Yash Chaudhary, Juhi Jain, Rashmi Singh, Rajeev Kaul

**Affiliations:** Department of Microbiology, University of Delhi South Campus, New Delhi, India

**Keywords:** PPRV, hemagglutinin, neuraminidase activity, secondary structure analysis, comparative modeling, amino acid substitutions, TLR 2, CD150

## Abstract

*Peste des petits ruminants* (PPR), a disease of socioeconomic importance has been a serious threat to small ruminants. The causative agent of this disease is PPR virus (PPRV) which belongs to the genus *Morbillivirus*. Hemagglutinin (H) is a PPRV coded transmembrane protein embedded in the viral envelope and plays a vital role in mediating the entry of virion particle into the cell. The infected host mounts an effective humoral response against H protein which is important for host to overcome the infection. In the present study, we have investigated structural, physiological and functional properties of hemagglutinin protein using various computational tools. The sequence analysis and structure prediction analysis show that hemagglutinin protein comprises of beta sheets as the predominant secondary structure, and may lack neuraminidase activity. PPRV-H consists of several important domains and motifs that form an essential scaffold which impart various critical roles to the protein. Comparative modeling predicted the protein to exist as a homo-tetramer that binds to its cognate cellular receptors. Certain amino acid substitutions identified by multiple sequence alignment were found to alter the predicted structure of the protein. PPRV-H through its predicted interaction with TLR-2 molecule may drive the expression of CD150 which could further propagate the virus into the host. Together, our study provides new insights into PPRV-H protein structure and its predicted functions.

## 1 Introduction

*Peste des petits ruminants* (PPR) is an important transboundary and fatal disease similar to other morbilliviruses that causes diseases such as measles in humans, and canine distemper disease in canines. Although the major target of this virus includes small ruminants such as goats and sheep, the seroprevalence of PPRV has also been reported from large ruminants such as buffaloes and camels (Balamurugan et al., 2012; Rahman et al., 2020). The characteristic symptoms of the disease include pyrexia, mucus filled discharge from nose and eyes of the infected animal accompanied with crust formation on eyelids. Abortion can also occur in the pregnant animals. In addition, the milk quality and quantity are severely affected (Baron et al., 2011). Mortality and morbidity rates of this disease can reach up to 90% which leads to huge economic losses mainly to small farmers (Mapaco et al., 2019). In 2015, the PPRV eradication strategy was adopted by Food and Agriculture Organization (FAO) and World Organization for Animal Health (OIE) with an aim to exterminate this disease by 2030.

The etiological agent PPR virus is enveloped and carries negative sense, single stranded, non-segmented RNA as genome. The genome codes for several structural and non-structural proteins that work in tandem to establish a successful infection in the host. The structural proteins are nucleocapsid (N), phosphoprotein (P), fusion (F), hemagglutinin (H) matrix (M) and large polymerase (L). The two non-structural proteins are C and V proteins which are translated from P gene transcript via utilization of an alternate open reading frame and an addition of extra guanosine in the P transcript, respectively (Mahapatra et al., 2003). PPR virus exists as a single serotype but have different lineages (Sevik and Sait, 2015; Rajko-Nenow et al., 2017). Distinction of different lineages of PPRV is based on partial gene sequencing of nucleocapsid and fusion genes. Till date, four different lineages of PPRV have been reported. The virus belonging to lineage 1 and 2 have been reported from Western and Central Africa, the lineage 3 being dominant in Eastern Africa and Middle East, and lineage 4 is found to be circulating in Asia (Sevik and Sait, 2015; Rajko-Nenow et al., 2017).

Virus mediates entry into the host cell by binding to its cognate receptors present on the host cell membrane. Morbilliviruses possess two transmembrane proteins on their envelope namely fusion (F), and hemagglutinin (H). The H protein is a type-2 transmembrane protein consisting of large extracellular domain, a single membrane spanning region and a short cytoplasmic tail. Cellular receptors recognition via viral proteins play a pivotal role in establishing a proficient infection and also gives an edge in shaping host range and tissue tropism. PPR virus is both lymphotropic and epitheliotropic in nature. While mediating entry into the host cell, the morbilliviruses coded H protein binds to cellular receptor signaling lymphocyte activation molecule (SLAM, also known as CD150) expressed on the surface of immune cells including macrophages, lymphocytes and dendritic cells (Cocks et al., 1995; Tatsuo et al., 2000, 2001). Interaction of the viral hemagglutinin with SLAM induces conformational changes into fusion protein which mediates fusion between the virus envelope and the target cell membrane. Besides SLAM receptor, morbilliviruses also use nectin-2 receptor present on the epithelial cells (Muhlebach et al., 2011; Noyce et al., 2011, 2013; Pratakpiriya et al., 2012).

Monomers of measles virus (MeV) coded H protein have been previously shown to oligomerize to form hemagglutinin tetramer that actively participates in receptor binding (Brindley and Plemper, 2010; Hashiguchi et al., 2011). The crystal structure of MeV-H protein has been deciphered using single wavelength anomalous dispersion (SAD) and resolved at 2.6 Å resolution. Unlike hemagglutinin-neuraminidase (HN) protein from other human paramyxoviruses that possess globular symmetry, the MeV-H protein has an overall typical cubical symmetry (Hashiguchi et al., 2007). The crystal structure of MeV-H protein showed that amino acids 157–607 constitute the head domain, which dimerize to form a homodimer subunit that shows β propeller sheets namely (β1–β6). Out of these propeller sheets, β5 forms a receptor binding unit. Several amino acids such as D505, D507, Y529, D530, T531, R533, F552 and P554 are involved in binding to the SLAM receptor. It has also been reported that critical amino acids involved in the recognition of sialic acid residues are found to be absent in MeV hemagglutinin protein (Hashiguchi et al., 2007). The supramolecular assembly of canine distemper virus (CDV) hemagglutinin has also been previously studied using cryo-EM. The head domain of this protein was found to exist as tetramer and asymmetric. Each monomer constituted an ectodomain which is further bifurcated into 3 domains, i.e., head, neck and stalk. Each monomer interacts with the other one to form β propeller structure with 6 blades (Kalbermatter et al., 2023).

Although the structural details of hemagglutinin protein of MeV and CDV have been reported and extensively studied, however no investigations or studies on structure of PPRV hemagglutinin have been performed. In this manuscript we have used computational approaches to describe the biophysiochemical, structural and functional properties of hemagglutinin protein from genus *Morbillivirus* with a key emphasis on PPRV-H protein.

## 2 Materials and methods

### 2.1 Analysis of biophysiological and structural properties of PPRV hemagglutinin protein using computational approaches

A number of physiological properties of hemagglutinin protein of PPR virus along with other members of this genus were predicted using ExPASy ProtParam tool^1^ (Gasteiger et al., 2003). Various parameters such as isoelectric point (pI), extinction coefficient, half-life, instability index was analyzed. These included H gene sequences of field strains from MeV, CDV and Rinderpest virus (RPV) whose Genbank references are listed in Table 1. The analysis was also performed using 40 PPRV-H sequences accessed from Genbank database (Supplementary file 1). These included H gene sequences of field strains from India, China and Africa. A consensus sequence of PPRV-H was generated from these 40 PPRV-H (Supplementary file 1) sequences using European Molecular Biology Open Software Suite (EMBOSS) cons tool^2^ and was subjected to similar analysis using ProtParam tool (Rice et al., 2000).

**Table 1:** Physiological properties of hemagglutinin protein of genus *Morbillivirus* computed using Expasy-Prot Param tool.

Morbilliviruses	Molecular weight (kDa)	Theoretical pI	Extinction coefficient (M^−1^ cm^−1^)	Estimated half life	Instability index	Aliphatic index	GRAVY^a^
*Peste des petits* ruminants Virus (PPRV) (ABY61988.1)	68.8	7.58	72,030	>20 (yeast) >10 (*E. coli*)	43.77	97.70	−0.112
*Peste des petits* ruminants virus (PPRV) (consensus)	68.8	6.64	70,540	>20 (yeast) >10 (*E. coli*)	46.20	98.34	−0.096
Measles virus (MV) (AAK64474.1)	69.10	6.76	86,010	>20 (yeast) >10 (*E. coli*)	29.95	92.22	−0.102
Canine distemper virus (CDV) (CAP17286.1)	68.5	6.41	79,020	>20 (yeast) >10 (*E. coli*)	46.19	96.33	−0.066
Rinderpest virus (RPV) (AAD25093.1)	68.02	7.51	86,470	>20 (yeast) >10 (*E. coli*)	38.73	96.68	−0.103

a Grand average of hydropathy.

The secondary structure of the protein was predicted using PsiPred^3^ (McGuffin et al., 2000). In addition, the Self-Optimized Prediction Method from Alignment (SOPMA) was used for prediction of information regarding the secondary structure of a protein in terms of percent alpha helices, beta sheets, coils and turns^4^ (Geourjon and Deleage, 1995). Different domains and family of hemagglutinin protein were identified using InterPro database^5^ (Paysan-Lafosse et al., 2023). Conserved Domain Database (CDD), a web program that identifies the location of conserved domains by deriving the information from the primary sequence of an uncharacterized protein was used to predict conserved domains of H protein.^6^ Identification of such domains is relevant in accessing the function of a protein or their evolutionary history (Marchler-Bauer et al., 2011). Motif elucidation of hemagglutinin protein was done using Multiple Expectation maximization for Motif Elicitation (MEME suite).^7^ The algorithm chosen to decipher the sequence motif was at least one motif per sequence, where assigned length of motifs could vary between 5 to 15 amino acids. The other parameters used in the program were kept as default (Bailey et al., 2009).

Swiss model is a user interactive web server that generates a 3 dimensional model of an unknown protein using a suitable template.^8^ The template then selected is further aligned against the query sequence and a suitable tertiary and/or quaternary structure is generated. Once the 3-D model is generated, the predicted structure is superimposed against the relevant and previously resolved 3-D structures of H protein using PyMol (Schwede et al., 2003; Rosignoli and Paiardini, 2022). All the known structures of hemagglutinin protein used in the current study were derived from Protein Data Bank (PDB) (Berman et al., 2000). This includes H protein from MeV (PDB ID-2ZB5), NDV (PDB ID-3T1E), CDV (PDB ID-7ZNY) and MuV (PDB ID-5B2D).

### 2.2 Effect of amino acid substitution on the secondary structure of the PPRV-H protein

The full length PPRV-H protein sequences were retrieved from National Centre of Biotechnology Institute (NCBI) database.^9^ The multiple sequence alignment was done using Clustal Omega and various amino acid substitutions were identified in the sequences. Thus, a standalone database was generated (Supplementary files 2, 3A). To assess the effect of amino acid substitutions on the secondary structure of a protein, PentUnFOLD algorithm was used. This algorithm works in conjunction with a PDB file and predicts the fragments of secondary structure (alpha helices and beta sheets) that can convert into random coils and disorder state (Poboinev et al., 2022). The PPRV-H tetramer predicted using homology modelling was used as template to identify non-stable secondary structures in the protein. Amino acid substitutions identified using MSA were then traced back on to these non-stable structures and their effects were predicted.

### 2.3 Identification of neuraminidase activity motifs in PPRV-H protein

To identify the key residues/motifs in the PPRV hemagglutinin protein, if any, that may possess neuraminidase activity, the multiple sequence alignment (MSA) analysis was performed. First, the peptide sequence of paramyxoviruses coded hemagglutinin proteins that are known to possess neuraminidase were obtained from Uniprot database.^10^ These include H proteins of Human Parainfluenza virus 3 (HPIV3), New Castle disease virus (NDV) and Mumps virus (MuV). These sequences were subjected to multiple sequence alignment with PPRV-H protein sequences using Clustal Omega to identify if PPRV H protein also contains motifs that may possess neuraminidase activity^11^ (Sievers et al., 2011).

### 2.4 PPRV-H and TLR-2 signaling cascade

Previous studies have shown that MeV-H protein interacts with host’s Toll like receptor 2 (TLR-2) and induces the expression of SLAM molecules on monocytes (Bieback et al., 2002). We identified the homologous protein of human TLR-2 and its downstream signaling proteins in family *Bovidae* using Uniprot and NCBI database. The various signaling molecules involved in the cascade mediated by TLR-2 were predicted using sequence and structural homology.

## 3 Results

### 3.1 Predicted physiological properties of PPRV-H protein

Various biophysical properties of hemagglutinin protein of PPRV-H along with others members in the genus *Morbillivirus* were analyzed using protparam tool. These included parameters such as isoelectric point, extinction coefficient, aliphatic index, stability and half-life of the proteins. In general, the hemagglutinin protein of genus *Morbillivirus* are likely to have high aliphatic index, molecular weight and, isoelectric point (theoretical) in the range 68–70 kDa and 6–7.5, respectively, (Table 1). The predicted parameters for the PPRV hemagglutinin protein included molecular weight of 68.8 kDa, isoelectric point at pH 7.58, extinction coefficient value to be 72,030 M^−1^ cm^−1^ at 280 nm if measured in water. The stability index of the protein is 43.7 which suggest that the protein is likely to be unstable under *in vitro* conditions. The aliphatic index of the protein is 97.7 indicating that the maximum portion of protein is occupied by aliphatic amino acids. Grand average of hydropathy (GRAVY) value of PPRV-H was found to be negative suggesting its hydrophilic nature. When the PPRV-H gene consensus sequence of 40 filed isolates was analyzed, similar results were obtained except for isoelectric point where it was predicted to be slightly acidic, i.e., 6.64 (Table 1).

### 3.2 Beta sheets are the dominant secondary structure in genus *Morbillivirus* including PPRV-H protein

**Figure 1:**
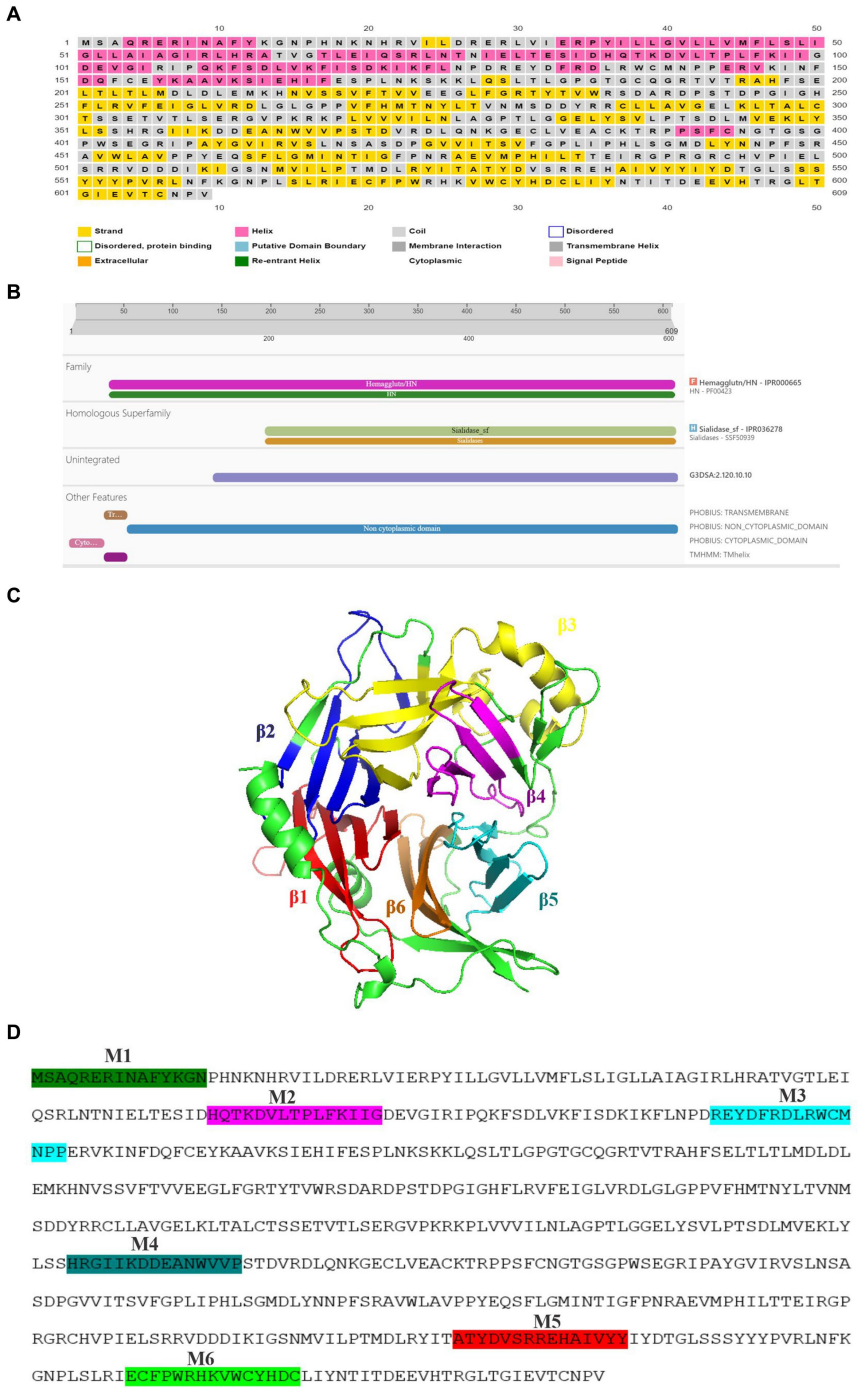
Structural features of PPRV-H protein. **(A)** Beta sheets (yellow) are the predominant secondary structure present in the hemagglutinin protein, predicted using PsiPred tool. **(B)** PPRV-H consist of three different domain: a non-cytoplasmic domain (blue) followed by transmembrane domain (brown) and cytoplasmic tail (pink) as predicted using InterPro database. **(C)** Presence of 6 different domain predicted using conserved domain database (each labelled using different color). **(D)** PPRV-H consists of six motifs (highlighted using different colors) that are conserved across the genus *Morbillivirus*.

Secondary structure content of a protein can be identified using several prediction tools. We have used PsiPred analysis tool in this study which revealed that hemagglutinin protein of morbilliviruses including PPRV are rich in beta strands followed by alpha helices (Figure 1A). The SOPMA analysis showed that in PPRV-H protein, the beta sheets (27%) and alpha helices (26%) are similar in proportion in comparison to H protein of other morbilliviruses in which the proportion of alpha helices (19–22%) is lower than beta sheets (27–28%) (Supplementary file 4).

### 3.3 PPRV-H protein sequences predicts it to be a type 2 transmembrane protein with multiple domains

The structural data acquired from the detailed analysis of MeV-H protein, revealed that MeV-H protein comprises of head domain that is primarily involved in receptor binding (Hashiguchi et al., 2007, 2011). The transmembrane helix as well as cytoplasmic domain is crucial for lipid raft association, virus growth and structural stability (Barman et al., 2004). We have used InterPro online tool for classification of protein families, predicting domains and important sites. Our analysis predicts that the PPRV hemagglutinin protein consist of three major physical domains: a short cytoplasmic tail ranging from 1st to 35th amino acid, a single pass transmembrane helix from 36th to 58th amino acid, which is followed by a large extracellular domain ranging from 59th to 609th amino acid. PPRV-H is designated as a type 2 transmembrane protein, i.e., having an amino terminus cytoplasmic tail and extracellular domain towards carboxyl side (Figure 1B). In our analysis, the PPRV-H protein is predicted to belong to hemagglutinin family and sialidase superfamily of proteins. The H protein of other morbilliviruses were also predicted to have similar features (Supplementary file 5).

### 3.4 CDD identified several conserved domains constituting the head region of PPRV-H

Previous studies on the structural analysis of hemagglutinin protein from morbilliviruses including MeV (crystal structure) and CDV (electron micrograph) have shown the presence of 6 domains rich in beta sheets (Hashiguchi et al., 2007, 2011, Kalbermatter et al., 2023). We then identified the conserved domains of PPRV hemagglutinin protein using Conserved Domain database (CDD) from NCBI. Our results showed that PPRV-H consists of 6 propeller domains which constitute the ectodomain of PPRV-H protein. These include domain 1 (216–279aa), domain 2 (284–334aa), domain 3 (347–430aa), domain 4 (439–481aa), domain 5 (507–553aa) and domain 6 (564–601aa) (Figure 1C). As all of these are present in extracellular domain, therefore it is likely that they may be critical for the receptor binding ability of H protein (Supplementary file 6) (Hashiguchi et al., 2011; Kalbermatter et al., 2023). Similar domains in MeV-H protein interact with those of other monomers via various covalent interactions such as disulphide linkages to form dimer or tetramer. Therefore, it is highly likely that the PPRV-H domains may perform similar functions as well.

### 3.5 Prediction of motifs in PPRV hemagglutinin which are conserved across different morbilliviruses

The conserved motifs present in the genus *Morbillivirus* were identified using MEME motif tool (Figure 1D). The presence of motifs can also predict the function and structure of a protein (Bailey, 2008). In our study, a total of six motifs were identified: motif 2 (86–100aa), motif 3 (129–144aa), motif 4 (354–368aa), motif 5 (527–541aa) and motif 6 (569–583aa). These were found to be localized on non-cytoplasmic domain, i.e., extracellular domain, indicating that they may have an important role in receptor binding. The motif 1 (1–15aa) was localized in cytoplasmic tail region which might play a role in viral growth and stability (Supplementary file 7).

### 3.6 PPRV-H protein predicted to exist as homo-tetramer and share structural homology with H protein coded by other morbilliviruses

**Figure 2:**
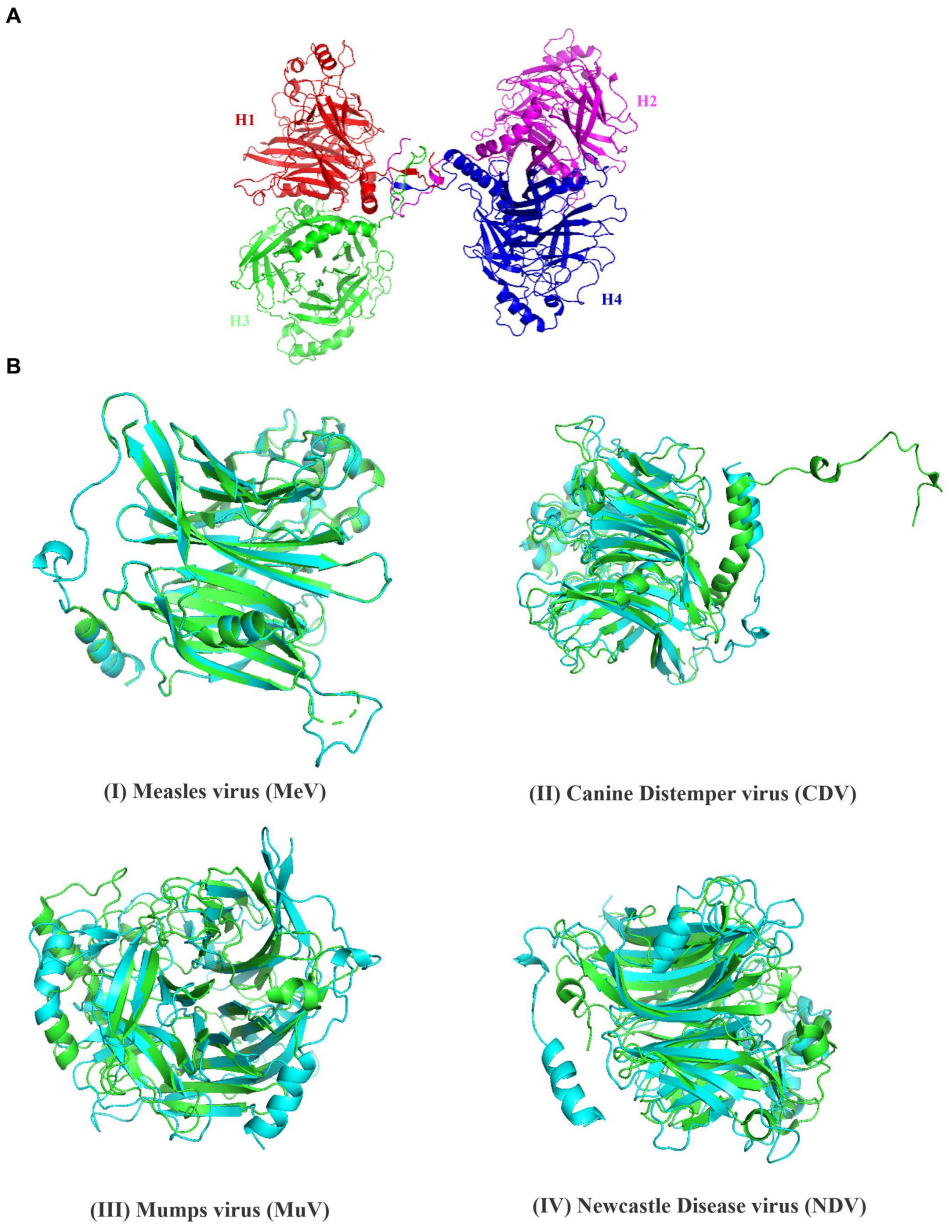
Structural analysis of hemagglutinin protein of PPRV. **(A)** The structure of PPRV-H tetramer was predicted using Swiss model. **(B)** Superimposed structure of the hemagglutinin protein from (I) Measles virus (green) (II) Canine distemper vinis (green) (III) Mumps virus (green) (IV) Newcastle disease virus (green) and predicted PPRV-H (monomer, cyan color).

By using various biophysical approaches, it has been previously established that hemagglutinin protein of many morbilliviruses exists as homo-tetramer. Previous studies have suggested that due to significant sequence similarity between the different members of morbilliviruses it is likely that the PPRV-H protein may also exist a homo-tetramer (Hashiguchi et al., 2011, Kalbermatter et al., 2023). In present study, we have modeled PPRV-H protein structure by homology modeling using Swiss model. The template used for building PPRV-H was Canine Distemper virus (CDV) H protein (PDB ID-7ZNY) having sequence identity and similarity of 0.35 and 0.38, respectively, (Figure 2A). The Global Model Quality Estimate value (GMQE) of the model is 0.60. The model also predicted the PPRV-H protein to be a homo-tetramer (Supplementary file 8). The PPRV-H protein homo-tetramer participates in receptor binding which includes SLAM and nectin located on lymphocyte and epithelial cells, respectively, and further mediating the fusion process (Prajapati et al., 2019). Using the same set of instructions which were used to model the tetramer of PPRV-H, the structure of PPRV-H monomer was also predicted. This structure was further superimposed with other resolved structures of H protein available in PDB and their root mean square deviation (RMSD) values were calculated. The RMSD values upon superimposition were found to be in the range of 0.14 to 4.52 Å. This study revealed that PPRV-H was structurally similar to the H protein of MeV (0.149 Å) and CDV (1.586 Å). In comparison RMSD values were higher when compared to New castle disease virus (NDV) (4.507 Å) or Mumps virus (MuV) (4.523 Å) (Figure 2B).

### 3.7 Amino acid substitutions might alter the structure of PPRV-H protein

Envelope or attachment proteins which includes hemagglutinin protein are major structural proteins that are under immense immune pressure and hence are continuously evolving. The polymorphism at any single amino acid position can also alter the structure as well as function of any protein. There are previous studies that suggest that amino acid substitutions in CDV-H protein can affect functional aspects of the protein such as CDV-H with R580Q substitution resulted in reduced surface expression as well as loss of fusion activity as compared to wild type strain. Most likely, such substitutions alter the overall structure of the CDV-H protein which renders the protein non-functional (Sattler et al., 2014). Another study had suggested that if substituted amino acids are present on protein surface, then they might be involved in virulence or defying the immune response in vaccinated candidates (Chen et al., 2018). With an aim to study the effect of amino acid polymorphism on the predicted structure of a protein, we analyzed PPRV-H sequences from 40 different isolates which includes field samples as well as vaccine strains and identified amino acid polymorphisms. Using PentUNFOLD algorithm, it was predicted that most of substitutions are occurring in coil or the stable region of the protein (Supplementary file 3B). However, some substitutions such as substitutions at position E155, V160, P170, S179, R240, D283, S302, T596 (Table 2) have a potential to alter structure of protein and convert the functional protein into disordered state. However, these predictions need to be validated using additional experimental approaches.

**Table 2:** Effect of amino acid substitution on the secondary structure of protein predicted using PentUnFOLD.

Susceptible amino acid position	Secondary structure region where susceptible amino acid is present	Amino acid substitution deduced from MSA	Effect on the secondary structure
E155	Present in non-stable helix	E155G	Can turn into random coil or disorder state
V160	Present in non-stable helix	V160K/D	Can turn into random coil or disorder state
P170	Present in non-stable helix	P170S	Can turn into random coil
K176	Present in non-stable helix	K176R	Can turn into random coil or disorder state
S179	Present in non-stable helix	S179L	Can turn into random coil
R240	Present in coil region	R240G	Can turn into disorder state
T233	Present in stable beta sheet	T233I	No structural change
D283	Present in coil region	D283V	Can turn into disorder state
G264	Present in coil region	G264E	No structural change
P267	Present in coil region	P267A	No structural change
V269	Present in stable beta sheet	V269I	No structural change
S302	Present in coil region	S302P	Can turn into disorder state
L344	Present in coil region	L344P	No structural change
M345	Present in coil region	M345T	No structural change
S399	Present in coil region	S399I	No structural change
R450	Present in coil region	R450K	No structural change
V452	Present in stable beta sheet	V452A	No structural change
M520	Present in coil region	M520K/R	No structural change
G546	Present in coil region	G546S	No structural change
R574	Present in coil region	R574Y/S	No structural change
I589	Present in coil region	I589M	No structural change
T596	Present in non-stable beta sheet	T596M	Can turn into random coil or disorder state

### 3.8 Neuraminidase activity of H protein of morbilliviruses

**Figure 3:**
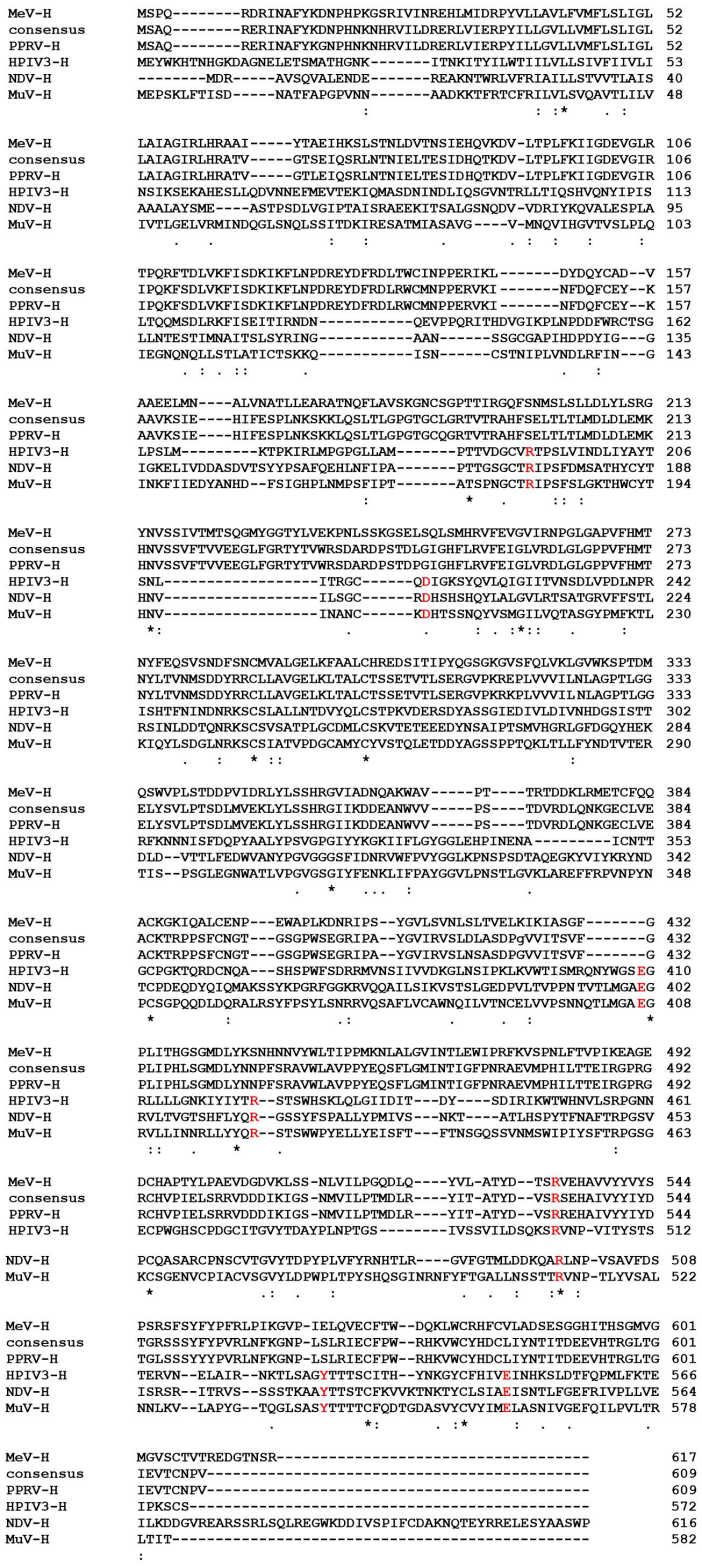
Multiple sequence alignment of PPRV-H protein with its homologs from other viruses, MeV (Measles virus), HPIV3 (Human Parainfluenza virus 3), NDV (Newcastle disease virus) and MuV (Mumps virus). The highlighted amino acids (red color) indicates amino acids that form sialic acid binding sites in the hemagglutinin protein.

Viruses such as Influenza A and B are known to code for proteins that have neuraminidase activity which aids in the release of progeny virus from the infected cells, and facilitate efficient propagation of virus into the host (Matrosovich et al., 2004). Viruses belonging to genera *Respirovirus, Avulavirus* and *Rubulavirus* in family *Paramyxoviridae* are also known to code for proteins which exhibit neuraminidase activity, whereas the viruses belonging to genus *Morbillivirus* are known to lack neuraminidase activity in general. A previous study on the structural analysis of MeV-H protein had clearly indicated that H protein lacks active site residues responsible for neuraminidase activity (Hashiguchi et al., 2007). Similarly, CDV-H protein has also been shown to be also devoid of neuraminidase activity (Rendon-Marin et al., 2019). Studies have shown that Human Parainfluenza Virus Type 3 coded H protein amino acid residues R192, D216, E409, R424, R502, Y530 and E549 are essential to mediate its binding to sialic acid (Chu et al., 2021). In present study, our data from multiple sequence alignment analysis has shown that PPRV hemagglutinin protein lacks the necessary residues critical for neuraminidase activity. However, such residues were present in related viruses including HPIV 3, NDV and MuV (Figure 3). Our study therefore predicts that PPRV-H is likely to lack neuraminidase activity. Further studies may be required to validate the same before concluding that the protein may be designated as hemagglutinin (H) instead of hemagglutinin-neuraminidase (HN).

### 3.9 PPRV-H protein may initiate Toll like receptor-2 signaling cascade

**Figure 4:**
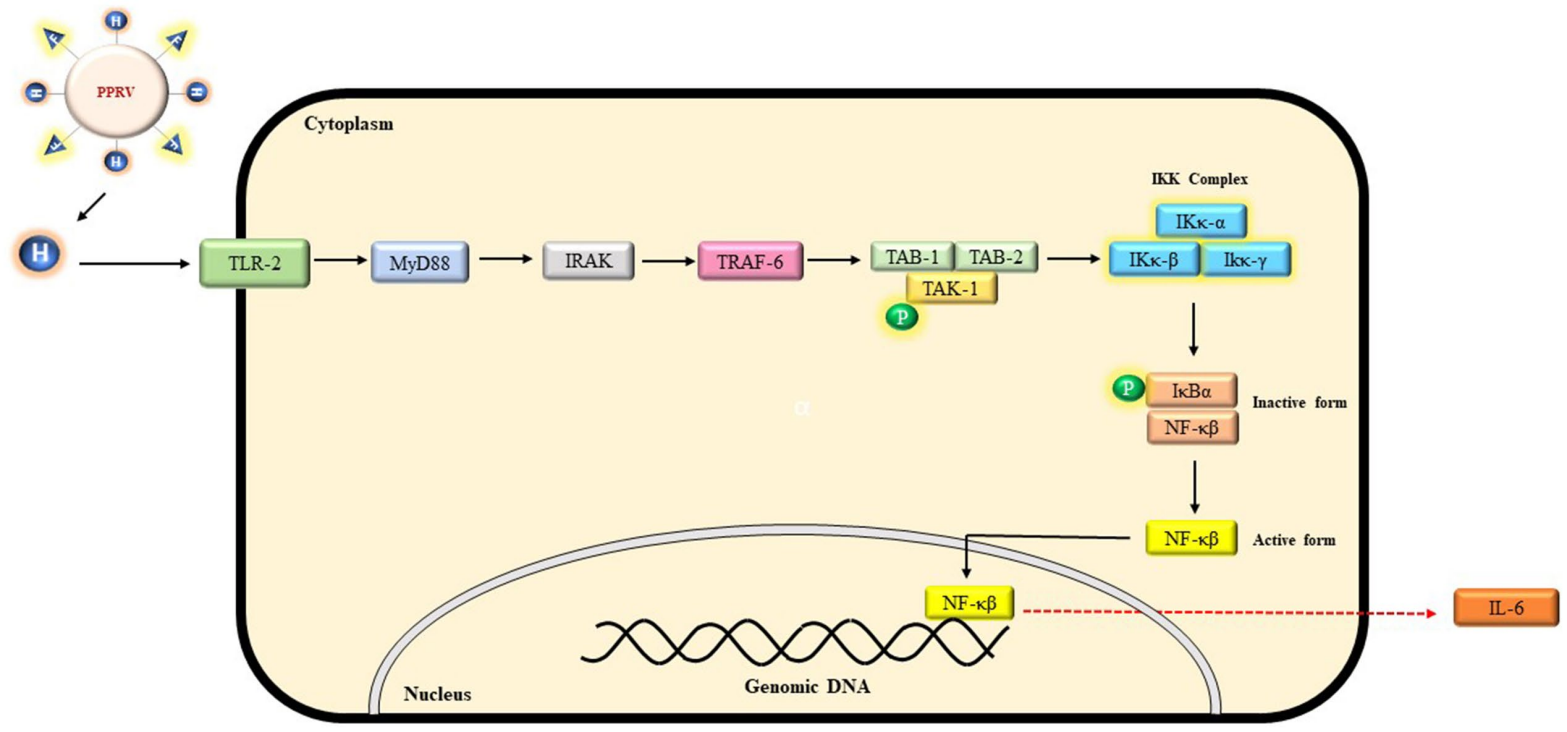
A probable schematic representation of TLR-2 signaling mediated by PPRV-H protein. Binding of H protein initiates a signaling cascade involving whereby an adaptor protein MyD88 recruits IRAK proteins which further activates TRAF-6 and a complex consisting of TAK-1 and TAK binding proteins (TABS). Phosphorylation of TAK-1 activates IKK complex which further degrades IκBα and NF-κB translocate into nucleus leading to the expression of IL-6.

MeV is known to interact with TLR which belongs to the family of pathogen recognition receptors (PRR). Different TLR are expressed in different compartments of the cell and recognize different component of the pathogen. For example, MeV genome is recognized by TLR 7 and 8 (Clifford et al., 2012). Previous studies have suggested that MeV encoded hemagglutinin protein forms a stable interaction with host’s TLR-2 molecule and induces the surface expression of CD150 via TLR-2 signaling mechanism (Bieback et al., 2002). PPRV can also possibly initiate similar signaling cascade in small ruminants through its H protein. In order to identify host proteins which could be critical in this process, we first compiled a list of various protein involved in TLR-2 signaling in humans from KEGG database (Kanehisa Laboratories, 2020). The primary sequences of each of these human proteins were retrieved using Uniprot and NCBI database. The human proteins sequences were analyzed for sequence and structural similarity to identify their homolog in bovines using Swiss model (Supplementary file 9). The bovine proteins were used as representatives for *Capra hircus* proteins as no reviewed protein sequences or their structures were available in the Uniprot database. Since both *Capra hircus* and *Bos taurus* belong to the family *Bovidae* and were derived from a common ancestor (De Lorenzi et al., 2015); so it could be predicted that both the genus might have some moderate level of genetic similarity. The bovine proteins thus identified were found to be structurally similar to the human homologs when Swiss modeling was performed (Table 3). Our study therefore has predicted bovine proteins that could be a part of TLR-2 mediated signaling upon activation by PPRV-H protein resulting in SLAM expression as observed in measles infection (Figure 4).

**Table 3:** List of components involved in TLR-2 signaling modeled using homology modelling (Swiss model).

Protein Modelled (*Bovidae*)	Template used (homo) PDB ID	Sequence identity	Sequence similarity	Coverage	GMQE^a^ value
Toll like receptor-2 (TLR-2)	6NIG	62.45	0.47	0.67	0.51
Myeloid differentiation primary response protein (MyD88)	B3Y683	98.65	0.61	1.00	0.81
IL-1 receptor associated kinase-4 (IRAK-4)	Q9NWZ3	90.22	0.58	1.00	0.84
IL-1 receptor associated kinase-1 (IRAK-1)	6BFN	86.63	0.57	0.46	0.37
TNF receptor associated factor 6 (TRAF-6)	Q9Y4K3	93.30	0.61	0.96	0.82
TGF-β activated kinase-1 (TAK-1)	8GW3	99.66	0.63	0.50	0.42
TAK-1 binding protein-1 (TAB-1)	5NZZ	100	0.61	1.00	0.80
TAK-1 binding protein-2 (TAB-2)	Q9NYJ8	96.68	0.61	1.00	0.52
Inhibitor of nuclear factor kappa-B kinase subunit α (IKK-α)	5TQW	98.31	0.61	0.88	0.68
Inhibitor of nuclear factor kappa-B kinase subunit β (IKK-β)	4KIK	95.48	0.60	0.88	0.71
NF-kappa B transcription factor p65	Q04206	92.66	0.59	0.99	0.72
Interleukin-6	1P9M	51.67	0.45	0.87	0.57

a GMQE, Global Mean Quality Estimate.

## 4 Discussion

Members of genus *Morbillivirus* cause serious illness in both humans and animals. One such disease is PPR caused by PPR virus that primarily affects goats and sheep. The disease is extremely contagious and endemic in various parts of the world. Virus genome codes for various structural proteins out of which, two are transmembrane proteins namely fusion protein and hemagglutinin protein. Hemagglutinin is an envelope protein which binds to its host cellular receptor proteins viz. SLAM and nectin. Binding of hemagglutinin protein to its receptor induces conformational changes in the fusion protein which mediates the fusion between the virion envelope and the host cell membrane (Navaratnarajah et al., 2009). In the present study, we have attempted to generate critical insights about structural and functional properties of PPRV hemagglutinin protein. PPRV can be used as a model to study important aspects of virus mediated pathogenesis such as disease progression and/or immune suppression in its natural host. The studies done on PPRV can then be extrapolated to understand the pathogenesis of other members of this genus where limited information about virus or their proteins are available or which cannot be investigated in their natural host such as MeV.

The present study focused on biochemical and biophysical characterization of hemagglutinin protein from the genus *Morbillivirus* with the special reference to PPRV. The pI determined using *in silico* approaches can be used for various applications such as protein purification and/or crystallization. The isoelectric point of PPRV-H is around neutral pH, i.e., 7.58 and the similar results were obtained when the H protein of other members were analyzed by ProtParam tool. The analysis showed that the isoelectric point of H protein of morbilliviruses is near to neutral in the range of 6.5–7.5. The instability index of a protein accounts for the stability of a protein under *in vitro* conditions. Studies have suggested that the instability of protein is due to the presence of certain dipeptides. The stability index with values lower than 40 are considered as stable. As per our studies, MeV and RPV H proteins are stable while H proteins derived from PPRV and CDV are identified as relatively unstable. Aliphatic index is one of the important parameters used to measure thermostability of a protein by taking into account the fact that the presence of aliphatic amino acids such as alanine, valine, leucine and isoleucine increases the thermostability of a protein. Aliphatic index of all members of this genus was found to be high. Such a high aliphatic index might account for the high thermal stability of hemagglutinin protein. Grand average of hydropathy (GRAVY) is a program that considers hydrophobic and hydrophilic nature of all the standard amino acids and calculates the mean hydropathy of a polypeptide chain of fixed length. It has values that can range from −2 to +2; negative value represents hydrophilic nature while positive value indicates hydrophobic nature of the protein. Our result shows the H proteins of all morbilliviruses have high ratio of hydrophilic residues located on the surface that can mediate interaction with solvents and play a critical role in maintaining or regulating the protein folding (Gasteiger et al., 2003).

PsiPred is a structure prediction software that accurately predicts the secondary structure and topology of transmembrane proteins. The original version of this tool utilized the PSI-BLAST to predict the secondary structure of a protein, whereas the current version enhances the accuracy by taking predictions from the 4 independent datasets into the account. Being one of the leading structure prediction tools, the accuracy of each residue is around 78% as per an independent evaluation (Bryson et al., 2005). When H protein sequences were subjected for the analysis of secondary structure prediction by PsiPred and SOPMA, our results showed that H proteins of different members of morbilliviruses are rich in beta sheets followed by alpha helices. Similar studies were done wherein it had been reported that Porcine Rubulavirus hemagglutinin protein is rich is beta sheets suggesting that hemagglutinin proteins of genus *Morbillivirus* are beta sheets enriched structures (Zenteno-Cuevas et al., 1998). Besides that, supramolecular assembly of MeV and CDV clearly depicts the presence of high ratio of beta sheets in their structures (Hashiguchi et al., 2011; Kalbermatter et al., 2023). SOPMA predictions suggested high proportions of alpha helices in PPRV-H protein as compared to any other morbillivirus (MeV, CDV and RPV). Higher proportion of alpha helices tends to enhance the stability of a protein as alpha helices can tolerate more mutations without altering the structure of the protein (Abrusan and Marsh, 2016). Higher proportion of alpha helices could even contribute to better thermal stability of proteins as seen in thermophilic bacteria (Yakimov et al., 2016).

Our analysis of PPRV-H and other H proteins of these genera have identified that hemagglutinin proteins are rich in beta sheets. The specific arrangement of these beta sheets around a central axis gives them beta propeller structure. Such a structure is observed in MeV-H protein. Crystal structure of MeV-H clearly indicates the presence of beta propeller fold consisting of 6 beta sheets and beta sheet 5 is primarily involved in mediating the attachment to the host’s receptor molecule (Hashiguchi et al., 2007). Six conserved domains were identified and these domains were a part of extracellular domain. PPRV-H domain 5 consist of several amino acids such as R533, Y541, Y543, F552 and Y553 that are involved in binding to the SLAM receptor (Meng et al., 2020).

The quaternary structure of protein was modeled using Swiss model and PPRV-H was identified as a homo-tetramer. GMQE value of the predicted model is 0.60, in which the values closer to 1 indicate high confidence level of the predicted model (Biasini et al., 2014). The quality of the predicted model was then assessed using Ramachandran plot. The Ramachandran plot showed that most of the residues were located in the sterically allowed regions. Our predictions coincide with the previous data whereby the active form of MeV and CDV hemagglutinin proteins were identified to be a homo-tetramer (Hashiguchi et al., 2007; Kalbermatter et al., 2023). Structure superimposition is a method to study the physical similarity between the two structures. The superimposition was performed using PyMol and RMSD values were calculated. The lower RMSD value indicates the conservation of H protein among different viral species. Our studies clearly indicate that PPRV-H shares a significant structural homology to the hemagglutinin protein from MeV as well as CDV.

The primary sequence of a protein plays an important role in framing the structure of protein and imparts a particular function to the protein. Amino acid substitutions can severely impact the protein at structural and functional levels (Sattler et al., 2014; Chen et al., 2018). While there are some substitutions that are neutral in nature and do not disrupt either the structure or function of a protein (Taverna and Goldstein, 2002). In our study, we have identified specific substitutions in PPRV-H protein and investigated their impact on the predicted secondary structure of the protein. Our study indicates that there were certain substitutions such as G264E, P267A, and M345T that occurred in the coil region and did not alter the secondary structure. However, some other substitutions such as E155G, P170S, T596M that occurred in the non-stable alpha helix and beta sheet regions have a potential to alter the helix or sheet structure into random coil or disordered structure. Other than altering the structure of protein, these substitutions can have an implication on the protein–protein interactions or might have some effects on antigenic sites of the protein which may help the virus to escape the humoral immune response of the host (Harvey et al., 2021).

Several viruses such as Influenza A and B which belongs to the family *Orthomyxoviridae* are known to catalyze the removal of terminal sialic acid and hence they are known as sialidases or neuraminidases (McAuley et al., 2019). Similarly, reports have suggested that the three genus of family *Paramyxoviridae* have neuraminidase activity. These genera are *Respirovirus, Avulavirus* and *Rubulavirus.* The structural analysis of MeV-H protein in a previous study had clearly indicated that the H protein lacks active site residues involved in the neuraminidase activity (Hashiguchi et al., 2007). Though our analysis from InterPro suggests that PPRV-H might have neuraminidase activity, however, upon critical analysis using multiple sequence alignment we found that PPRV-H lacks critical amino acids responsible for interacting with the sialic acid residues. Hence, it is highly likely that PPRV-H lacks any neuraminidase activity. Although there have been some old reports which had suggested that PPRV-H could have neuraminidase activity. In one such study by Seth and Shaila (2001), when PPRV-H was overexpressed in mammalian cells they exhibited neuraminidase activities. They have also shown that substrates with α-2,3 glycosidic linkage served as a better substrate as compared to the substrates that have α-2,6 glycosidic linkage. However, in the same study, they had also shown that PPRV TN 87/1 virus strain showed no neuraminidase activity (Seth and Shaila, 2001). In another study, it has been reported that PPRV showed mild neuraminidase activity when bovine submaxillary mucin was used as substrate while no activity could be observed in MeV and CDV (Langedijk et al., 1997). Therefore, it is possible that PPRV or other morbilliviruses might have different mechanism of catalysis or may exploit some different active sites as limited neuraminidase activity was observed.

Homology or comparative modeling is one of the easiest method to decode the structure of an uncharacterized protein, provided that a suitable template having a minimum of 30% sequence identity is available in the database (Xiang, 2006). Structural similarity can be used to predict the function of the uncharacterized or novel protein (Li et al., 2018; Bartas et al., 2022). In a previous study, it was shown that MeV-H protein can activate TLR-2 signaling (Bieback et al., 2002). Using Swiss model, various proteins involved in TLR-2 signaling pathways were modelled. Since no native protein structures and reviewed protein sequence for *Capra* was available in the database, the proteins from *Bos taurus* were used as representative. Moreover, TLR-2 signaling has been reported to be critical for host defense in bovines as well (Bartens et al., 2021).

Toll-like receptors (TLRs) are essential component of innate immune system that recognize the pathogen-associated molecular patterns (PAMPs) associated with various viruses, bacteria and fungal pathogens to elicit an inflammatory immune response (Akira et al., 2006). Different components of pathogens are recognized by different TLRs. For instance, bacterial lipopolysaccharide (LPS) is recognized by TLR-4, viral single strand RNA is recognized by TLR-7 etc. In the present study, we suggest that PPRV-H may activate TLR-2 signaling pathway in myeloid differentiation primary response 88 (MyD88) dependent manner. Upon activation of TLR-2 by PPRV-H protein, MyD88 could then interact with IL-1 receptor-associated kinase 4 (IRAK-4) which in turn can activate (IRAK-1). Both the IRAK proteins can then further interact with the tumor necrosis factor receptor associated factor 6 (TRAF-6). TRAF-6 is a ubiquitin ligase which along with E2 and ubiquitin conjugating enzymes promotes Lys63 linked polyubiquitination of TRAF6 and IKκ-γ. These ubiquitinated proteins further activate a complex consisting of transforming growth factor-β activated kinase-1 (TAK1) and TAK binding proteins (TAB1 and TAB2). This TAB-TAK complex can then activate IKκ complex and MAP kinase pathway which leads to the translocation of NF-κB into the nucleus leading to the expression of interleukin-6 (IL-6) (Kawai and Akira, 2007; Oliveira-Nascimento et al., 2012; Caplan and Maguire-Zeiss, 2018).

Using human proteins as a reference, the protein homologs from *Bos taurus* were identified and subjected to homology modeling. Several parameters such as sequence coverage, similarity, GMQE value were used to generate high quality 3-D structures. Since most of the proteins involved in the pathway share high structural similarity with its human homologue, and PPRV-H is also similar to MeV-H protein, it can be predicted that similar type of TLR-2 signaling might be occurring in small ruminant likewise in humans which induces the surface expression of SLAM molecules and further propagates the virus into the body. Identification of lead molecules that can inhibit TLR-2 can also serve as potential drug that can further halt the multiplication of virus into the host.

## Data availability statement

The original contributions presented in the study are included in the article/Supplementary material, further inquiries can be directed to the corresponding author.

## Author contributions

SG: Conceptualization, Data curation, Formal analysis, Investigation, Methodology, Visualization, Writing – original draft. YC: Methodology, Writing – original draft. JJ: Methodology, Writing – original draft. RS: Methodology, Writing – original draft. RK: Conceptualization, Funding acquisition, Project administration, Resources, Software, Supervision, Validation, Visualization, Writing – review & editing.

## Footnotes

^1^https://web.expasy.org/protparam/

^2^https://www.ebi.ac.uk/Tools/msa/emboss_cons/

^3^http://bioinf.cs.ucl.ac.uk/psipred/

^4^https://npsa-pbil.ibcp.fr/cgi-bin/npsa_automat.pl?page=/NPSA/npsa_sopma.html

^5^https://www.ebi.ac.uk/interpro/

^6^https://www.ncbi.nlm.nih.gov/Structure/cdd/wrpsb.cgi

^7^https://meme-suite.org/meme/tools/meme

^8^https://swissmodel.expasy.org/

^9^https://www.ncbi.nlm.nih.gov/

^10^https://www.uniprot.org/

^11^https://www.ebi.ac.uk/Tools/msa/clustalo/
