# Supplementary material for: Structural and functional characterization of peste des petits ruminants virus coded hemagglutinin protein using various in-silico approaches
Source: Front Microbiol. 2024 Jun 20;15:1427606. doi: 10.3389/fmicb.2024.1427606 (PMC11222573; doi:10.3389/fmicb.2024.1427606)
Supplement: Supplementary file 1 [file Data_Sheet_1.PDF]

Supplementary file 1: List of PPRV-H sequences used in the present study

Genbank ID	Country	Sample Type
AKQ09544.1	India	Field Sample
AMX28327.1	India	Field Sample
AKR81281.1	India	Field Sample
ANS59483.1	India	Field Sample
AHG50444.1	India	Field Sample
AKG94169.1	India	Field Sample
ALO02851.1	India	Field Sample
ABY61986.1	India	Field Sample
CAD54790.2	India	Vaccine
ADN03213.1	India	Vaccine
ACN62119.1	India	Field Sample
ABY61988.1	India	Field Sample
AAS68031.1	India	Vaccine
ADJ05525.1	china	Field Sample
ASN63873.1	china	Field Sample
AJE30397.1	china	Field Sample
ALM55670.1	china	Field Sample
AJE30404.1	china	Field Sample
ASN63994.1	china	Field Sample
ASN64054.1	china	Field Sample
ASN64006.1	china	Field Sample
AKN58853.1	china	Field Sample
AJA39814.1	china	Field Sample
AIK97759.1	china	Field Sample
AJE30413.1	china	Field Sample
ANG60369.1	Nigeria	Field Sample
ANG60361.1	Nigeria	Field Sample
ABX75312.1	Nigeria	Field Sample
CAJ01700.1	Nigeria	Vaccine
ADX95995.1	Nigeria	Vaccine
AJT59441.1	Senegal	Field Sample
AIK19904.1	Senegal	Field Sample
AKT04315.1	Benin	Field Sample
AKT04307.1	Benin	Field Sample
AGG09146.1	Morocco	Field Sample
AIL54004.1	Ethopia	Field Sample
AID07002.1	Ghana	Field Sample
AIL29370.1	Turkey	Field Sample
ART66998.1	Algeria	Field Sample
ARP51875.1	Mongolia	Field Sample
