# Supplementary material for: Structural and functional characterization of peste des petits ruminants virus coded hemagglutinin protein using various in-silico approaches
Source: Front Microbiol. 2024 Jun 20;15:1427606. doi: 10.3389/fmicb.2024.1427606 (PMC11222573; doi:10.3389/fmicb.2024.1427606)
Supplement: Supplementary file 2 [file Data_Sheet_2.PDF]

CLUSTAL O(1.2.4) multiple sequence alignment

```
ANG60361.1      MSAQRERINAFYKDNPHNKNHRVILDRERLIIERPYILLGVLLVMFLSLIGLLAIAGIRL 60
AKQ09544.1      MSAQRERINAFYKGNPHNKNHRVILDRERLIIERPYILLGALLVMFLSLIGLLAIAGIRL 60
AMX28327.1      MSAQRERINAFYKDNPHNKNHRVILDRERLIIERPYILLGALLVMFLSLIGLLAIAGIRL 60
AKR81281.1      MSAQRERINAFYKDNPHNKNHRVILDRERLIIERPYILLGALLVMFLSLIGLLAIAGIRL 60
ANS59483.1      MSAQRERINAFYKDNPHNKNHRVILDRERLIIERPYILLGALLVMFLSLIGLLAIAGIRL 60
AHG50444.1      MSAQRERINAFYKDNPHNKNHRVILDRERLVIERPYILLGVLLVMFLSLIGLLAIAGIRL 60
AKG94169.1      MSAQRERINAFYKDNPHNKNHRVILDRERLVIERPYILLGVLLVMFLSLIGLLAIAGIRL 60
ALO02851.1      MSAQRERINAFYKGNPHNKNHRVILDRERLVIERPYILLGVLLVMFLSLIGLLAIAGIRL 60
ABY61986.1      MSAQRERINAFYKGNPHNKNHRVILDRERLVIERPYILLGVLLVMFLSLIGLLAIAGIRL 60
CAD54790.2      MSAQRERINAFYKGNPHNKNHRVILDRERLVIERPYILLGVLLVMFLSLIGLLAIAGIRL 60
ADN03213.1      MSAQRERINAFYKGNPHNKNHRVILDRERLVIERPYILLGVLLVMFLSLIGLLAIAGIRL 60
ACN62119.1      MSAQRERINAFYKGNPHNKNHRVILDRERLVIERPYILLGVLLVMFLSLIGLLAIAGIRL 60
ABY61988.1      MSAQRERINAFYKGNPHNKNHRVILDRERLVIERPYILLGVLLVMFLSLIGLLAIAGIRL 60
AAS68031.1      MSAQRERINAFYKGNPHNKNHRVILDRERLVIERPYILLGVLLVMFLSLIGLLAIAGIRL 60
AIL29370.1      MSAQREMINAFYKDNPHNKNHRVILDRERLVIERPYILLGVLLVMFLSLIGLLAIAGIRL 60
ADJ05525.1      MSAQRERINAFFKDNPHNKNHRVILDRERLVIERPYILLGVLLVMFLSLIGLLAIAGIRL 60
ASN63873.1      MSAQRERINAFYKDNPHNKNHRVILDRERLVIERPYILLGVLLVMFLSLIGLLAIAGIRL 60
ARP51875.1      MSAQRERINAFYKDNPHNKNHRVILDRERLVIERPYILLGVLLVMFLSLIGLLAIAGIRL 60
AJE30397.1      MSAQRERINAFYKDNPHNKNHRVILDRERLVIERPYILLGVLLVMFLSLIGLLAIAGIRL 60
ALM55670.1      MSAQRERINAFYKDNPHNKNHRVILDRERLVIERPYILLGVLLVMFLSLIGLLAIAGIRL 60
AJE30404.1      MSAQRERINAFYKDNPHNKNHRVILDRERLVIERPYILLGVLLVMFLSLIGLLAIAGIRL 60
ASN63994.1      MSAQRERINAFYKDNPHNKNHRVILDRERLVIERPYILLGVLLVMFLSLIGLLAIAGIRL 60
ASN64054.1      MSAQRERINAFYKDNPHNKNYRVILDRERLVIERPYILLGVLLVMFLSLIGLLAIAGIRL 60
ASN64006.1      MSAQRERINAFYKDNPHNKNHRVILDRERLVIERPYILLGVLLVMFLSLIGLLAIAGIRL 60
AKN58853.1      MSAQRERINAFYKDNPHNKNHRVILDRERLVIERPYILLGVLLVMFLSLIGLLAIAGIRL 60
AJA39814.1      MSAQRERINAFYKDNPHNKNHRVILDRERLVIERPYILLGVLLVMFLSLIGLLAIAGIRL 60
AIK97759.1      MSAQRERINAFYKDNPHNKNHRVILDRERLVIERPYILLGVLLVMFLSLIGLLAIAGIRL 60
AJE30413.1      MSAQRERINAFYKDNPHNKNHRVILDRERLVIERPYILLGVLLVMFLSLIGLLAIAGIRL 60
AIL54004.1      MSAQRERINAFYKDNPHNKNHRVILDRERLVIERPYILLGVLLVMFLSLIGLLAIAGIRL 60
AGG09146.1      MSAQRERINAFYKDNPHNKNHRVILDRERLVIERPYILLGVLLVMFLSLIGLLAIAGIRL 60
ART66998.1      MSAQRERINAFYKDNPHNKNHRVILDRERLVIERPYILLGVLLVMFLSLIGLLAIAGIRL 60
AJT59441.1      MSAQRERINAFYKDNLHNKNHRVILDRERLTIERPYILLGVLLVMFLSLIGLLAIAGIRL 60
ABX75312.1      MSAQRERINAFYKDNPHNKNHRIILDRERLTIERPYILLGVLLVMFLSLIGLLAIAGIRL 60
AKT04315.1      MSAQRERINAFYKDNLHNKNHRVILDRERLTIERPYILLGVLLVMFLSLIGLLAIAGIRL 60
CAJ01700.1      MSAQRERINAFYKDNLHNKTHRVILDRERLTIERPYILLGVLLVMFLSLIGLLAIAGIRL 60
ADX95995.1      MSAQRERINAFYKDNLHNKTHRVILDRERLTIERPYILLGVLLVMFLSLIGLLAIAGIRL 60
AIK19904.1      MSAQRERINAFYKDNPHNKNHRIILDRERLTIERPYILLGVLLVMFLSLIGLLAIAGIRL 60
AID07002.1      MSAQRERINAFYKDNPHNKNHRIILDRERLTIERPYILLGVLLVMFLSLIGLLAIAGIRL 60
AKT04307.1      MSAQRERINAFYKDNPHNKNHRIILDRERLTIERPYILLGVLLVMFLSLIGLLAIAGIRL 60
ANG60369.1      MSAQRERINAFYKDNPHNKNHRIILDRERLTIERPYILLGVLLVMFLSLIGLLAIAGIRL 60
                ****** ****:*.* ***.:*:******* *********.*******************
```

```
ANG60361.1      HRATVGTSEIQSRLNTNIELTESIDHQTKDVLTPLFKIIGDEVGIRIPQKFSDLVKFISD 120
AKQ09544.1      HRATVGTSEIQSRLNTNIKLTESIDHQTKDVLTPLFKIIGDEVGIRIPQKFSDLVKFISD 120
AMX28327.1      HRATVGTSEIQSRLNTNIKLTESIDHQTKDVLTPLFKIIGDEVGIRIPQKFSDLVKFISD 120
AKR81281.1      HRATVGTSEIQSRLNTNIKLTESIDHQTKDVLTPLFKIIGDEVGIRIPQKFSDLVKFISD 120
ANS59483.1      HRATVGTSEIQSRLNTNIKLTESIDHQTKDVLTPLFKIIGDEVGIRIPQKFSDLVKFISD 120
AHG50444.1      HRATVGTLEIQSRLNTNIELTESIDHQTKDVLTPLFKIIGDEVGIRIPQKFSDLVKFISD 120
AKG94169.1      HRATVGTLEIQSRLNTNIELTESIDHQTKDVLTPLFKIIGDEVGIRIPQKFSDLVKFISD 120
ALO02851.1      HRATVGTLEIQSRLNTNIELTESIDHQTKDVLTPLFKIIGDEVGIRIPQKFSDLVKFISD 120
ABY61986.1      HRATVGTLEIQSRLNTNIELTESIDHQTKDVLTPLFKIIGDEVGIRIPQKFSDLVKFISD 120
CAD54790.2      HRATVGTLEIQSRLNTNIELTESIDHQTKDVLTPLFKIIGDEVGIRIPQKFSDLVKFISD 120
ADN03213.1      HRATVGTLEIQSRLNTNIELTESIDHQTKDVLTPLFKIIGDEVGIRIPQKFSDLVKFISD 120
ACN62119.1      HRATVGTLEIQSRLNTNIELTESIDHQTKDVLTPLFKIIGDEVGIRIPQKFSDLVKFISD 120
ABY61988.1      HRATVGTLEIQSRLNTNIELTESIDHQTKDVLTPLFKIIGDEVGIRIPQKFSDLVKFISD 120
AAS68031.1      HRATVGTLEIQSRLNTNIELTESIDHQTKDVLTPLFKIIGDEVGIRIPQKFSDLVKFISD 120
AIL29370.1      HRATVGTSEIQSRLNTNIELTESIDHQTKDVLTPLFKIIGDEVGIRIPQKFSDLVKFISD 120
ADJ05525.1      HRATVGTSEIQSRLNTNIKLTESIDHQTKDVLTPLFKIIGDEVGIRIPQKFSDLVKFISD 120
ASN63873.1      HRATVGTSEIQSRLNTNIKLTESIDHQTKDVLTPLFKIIGDEVGIRIPQKFSDLVKFISD 120
ARP51875.1      HRATVGTSEIQSRLNTNIKLTESIDHQTKDVLTPLFKIIGDEVGIRIPQKFSDLVKFISD 120
AJE30397.1      HRATVGTSEIQSRLNTNIKLTESIDHQTKDVLTPLFKIIGDEVGIRIPQKFSDLVKFISD 120
ALM55670.1      HRATVGTSEIQSRLNTNIKLTESIDHQTKDVLTPLFKIIGDEVGIRIPQKFSDLVKFISD 120
AJE30404.1      HRATVGTSEIQSRLNTNIKLTESIDHQTKDVLTPLFKIIGDEVGIRIPQKFSDLVKFISD 120
ASN63994.1      HRATVGTSEIQSRLNTNIKLTESIDHQTKDVLTPLFKIIGDEVGIRIPQKFSDLVKFISD 120
ASN64054.1      HRATVGTSEIQSRLNTNIKLTESIDHQTKDVLTPLFKIIGDEVGIRIPQKFSDLVKFISD 120
ASN64006.1      HRATVGTSEIQSRLNTNIKLTESIDHQTKDVLTPLFKIIGDEVGIRIPQKFSDLVKFISD 120
AKN58853.1      HRATVGTSEIQSRLNTNIKLTESIDHQTKDVLTPLFKIIGDEVGIRIPQKFSDLVKFISD 120
AJA39814.1      HRATVGTSEIQSRLNTNIKLTESIDHQTKDVLTPLFKIIGDEVGIRIPQKFSDLVKFISD 120
AIK97759.1      HRATVGTSEIQSRLNTNIKLTESIDHQTKDVLTPLFKIIGDEVGIRIPQKFSDLVKFISD 120
AJE30413.1      HRATVGTSEIQSRLNTNIKLTESIDHQTKDVLTPLFKIIGDEVGIRIPQKFSDLVKFISD 120
AIL54004.1      HRATVGTSEIQNRLNTNIELTESIDHQTKDVLTPLFKIIGDEVGIRIPQKFSDLVKFISD 120
AGG09146.1      HRATVGTSEIQSRLNTNIELTESIDHQTKDVLTPLFKIIGDEVGIRIPQKFSDLVKFISD 120
ART66998.1      HRATVGTSEIQSRLNTNIELTESIDHQTKDVLTPLFKIIGDEVGIRIPQKFSDLVKFISD 120
AJT59441.1      HRATVGTAEIQSRLNTNIELTESIDHQTKNVLTPLFKVIGDEVGIRIPQKFSDLVKFISD 120
ABX75312.1      HRATVGTAEIQSRLKTNIELTESIDHQTKDVLTPLFKIIGDEVGIRIPQKFSDLVKFISD 120
AKT04315.1      HRATVGTAEIQSRLNTNIELTESIDHQTKDVLTPLFKIIGDEVGIRIPQKFSDLVKFISD 120
CAJ01700.1      HRATVGTAEIQSRLNTNIELTESIDHQTKDVLTPLFKIIGDEVGIRIPQKFSDLVKFISD 120
ADX95995.1      HRATVGTAEIQSRLNTNIELTESIDHQTKDVLTPLFKIIGDEVGIRIPQKFSDLVKFISD 120
AIK19904.1      HRATVGTAEIQSRLNTNIELTESIDHQTKDVLTPLFKIIGDEVGIRIPQKFSDLVKFISD 120
AID07002.1      HRATVGTAEIQSRLNTNIELTESIDHQTKDVLTPLFKIIGDEVGIRIPQKFSDLVKFISD 120
AKT04307.1      HRATVGTAEIQSRLNTNIELTESIDHQTKDVLTPLFKIIGDEVGIRIPQKFSDLVKFISD 120
ANG60369.1      HRATVGTAEIQSRLNTNIELTESIDHQTKDVLTPLFKIIGDEVGIRIPQKFSDLVKFISD 120
                ******* ***.**:***:**********:*******:**********************
```

```
ANG60361.1      KIKFLNPDREYDFRDLRWCMNPPERVKINFDQFCEYKAAVKSIEHIFESPLSKSKELRSL 180
AKQ09544.1      KIKFLNPDREYDFRDLRWCMSPPERVKINFDQFCEYKAAVNSIEHIFESPLNKSKKLQSL 180
AMX28327.1      KIKFLNPDREYDFRDLRWCMSPPERVKINFDQFCEYKAAVNSIEHIFESPLNKSKKLQSL 180
AKR81281.1      KIKFLNPDREYDFRDLRWCMSPPERVKINFDQFCEYKAAVNSIEHIFESPLNKSKKLQSL 180
ANS59483.1      KIKFLNPDREYDFRDLRWCMSPPERVKINFDQFCEYKAAVNSIEHIFESPLNKSKKLQSL 180
AHG50444.1      KIKFLNPDREYDFRDLRWCMNPPERVKINFDQFCEYKAAVKSIEHIFESPLNKSKKLQSL 180
AKG94169.1      KIKFLNPDREYDFRDLRWCMNPPERVKINFDQFCEYKAAVKSIEHIFESPLNKSKKLQSL 180
ALO02851.1      KIKFLNPDREYDFRDLRWCMNPPERVKINFDQFCEYKAAVKSIEHIFESPLNKSKKLQSL 180
ABY61986.1      KIKFLNPDREYDFRDLRWCMNPPERVKINFDQFCEYKAAVKSIEHIFESPLNKSKKLQSL 180
CAD54790.2      KIKFLNPDREYDFRDLRWCMNPPERVKINFDQFCEYKAAVKSIEHIFESPLNKSKKLQSL 180
ADN03213.1      KIKFLNPDREYDFRDLRWCMNPPERVKINFDQFCEYKAAVKSIEHIFESPLNKSKKLQSL 180
ACN62119.1      KIKFLNPDREYDFRDLRWCMNPPERVKINFDQFCEYKAAVKSIEHIFESPLNKSKKLQSL 180
ABY61988.1      KIKFLNPDREYDFRDLRWCMNPPERVKINFDQFCEYKAAVKSIEHIFESPLNKSKKLQSL 180
AAS68031.1      KIKFLNPDREYDFRDLRWCMNPPERVKINFDQFCEYKAAVKSIEHIFESPLNKSKKLQSL 180
AIL29370.1      KIKFLNPDREYDFRDLRWCMNPPERVKINFDQFCEYKAAVKSIEHIFESPLNKSKKLQSL 180
ADJ05525.1      KIKFLNPDREYDFRDLRWCMNPPERVKINFDQFCEYKAAVKSIEHIFESPLNKSKKLQSL 180
ASN63873.1      KIKFLNPDREYDFRDLRWCMNPPERVKINFDQFCEYKAAVKSIEHIFESPLNKSKKLQSL 180
ARP51875.1      KIKFLNPDREYDFRDLRWCMNPPERVKINFDQFCEYRAAVKSIEHIFESPLNKSKKLQSL 180
AJE30397.1      KIKFLNPDREYDFRDLRWCMNPPERVKINFDQFCEYKAAVKSIEHIFESPLNKSKKLQSL 180
ALM55670.1      KIKFLNPDREYDFRDLRWCMNPPERVKINFDQFCEYKAAVKSIEHIFESPLNKSKKLQSL 180
AJE30404.1      KIKFLNPDREYDFRDLRWCMNPPERVKINFDQFCEYKAAVKSIEHIFESPLNKSKKLQSL 180
ASN63994.1      KIKFLNPDREYDFRDLRWCMNPPERVKINFDQFCEYKAAVKSIEHIFESPLNKSKKLQSL 180
ASN64054.1      KIKFLNPDREYDFRDLRWCMNPPERVKINFDQFCEYKAAVKSIEHIFESPLNKSKKLQSL 180
ASN64006.1      KIKFLNPDREYDFRDLRWCMNPPERVKINFDQFCEYKAAVKSIEHIFESPLNKSKKLQSL 180
AKN58853.1      KIKFLNPDREYDFRDLRWCMNPPERVKINFDQFCEYKAAVKSIEHIFESPLNKSKKLQSL 180
AJA39814.1      KIKFLNPDREYDFRDLRWCMNPPERVKINFDQFCEYKAAVKSIEHIFESPLNKSKKLQSL 180
AIK97759.1      KIKFLNPDREYDFRDLRWCMNPPERVKINFDQFCEYKAAVKSIEHIFESPLNKSKKLQSL 180
AJE30413.1      KIKFLNPDREYDFRDLRWCMNPPERVKINFDQFCEYKAAVKSIEHIFESPLNKSKKLQSL 180
AIL54004.1      KIKFLNPDREYDFRDLRWCMNPPERVKINFDQFCEYKAAAKSIEHIFESPLNKSKKLQSL 180
AGG09146.1      KIKFLNPDREYDFRDLRWCMNPPERVKIDFDQFCGYKAAVKSIEHIFESPLNKSKKLQSL 180
ART66998.1      KIKFLNPDREYDFRDLRWCMNPPERVKINFDQFCEYKAAVKSIEHIFESPLNKSKKLQSL 180
AJT59441.1      KIKFLNPDREYDFRDLRWCMNPPERVKINFDQFCEYKAADKSIEHIFESSLNRSRRLRLL 180
ABX75312.1      KIKFLNPDREYDFRDLRWCMNPPERVKINFDQFCEYKAAVKSVEHIFESSLNRSEMLRLL 180
AKT04315.1      KIKFLNPDREYDFRDLRWCMNPPERVKINFDQFCEYKAAVKSVEHIFESSLNRSERLRLL 180
CAJ01700.1      KIKFLNPDREYDFRDLRWCMNPPERVKINFDQFCEYKAAVKSVEHIFESSLNRSERLRLL 180
ADX95995.1      KIKFLNPDREYDFRDLRWCMNPPERVKINFDQFCEYKAAVKSVEHIFESSLNRSERLRLL 180
AIK19904.1      KIKFLNPDREYDFRDLRWCMNPPERVKINFDQFCEYKAAVKSVEHIFESSFNRSERLRLL 180
AID07002.1      KIKFLNPDREYDFRDLRWCMNPPERVKINFDQFCEYKAAVKSVEHIFESSFNRSERLRLL 180
AKT04307.1      KIKFLNPDREYDFRDLRWCMNPPERVKINFDQFCEYKAAVKSVEHIFESSFNRSERLRLL 180
ANG60369.1      KIKFLNPDREYDFRDLRWCMNPPERVKINFDQFCEYKAAVKSVEHIFESSFNRSERLRLL 180
                ********************.*******:***** *:** :*:****** :.:*. *: *
```

```
ANG60361.1      TLGPRTGCLGRTVTRAHFSELTLTLMDLDLEMKHNVSSVFTVVEEGLFGRTYTVWRSDAR 240
AKQ09544.1      TLGPGTGCQGRTVTRAHFSELTLTLMDLDLEMKHNVSSVFTVVEEGLFGRTYTVWRSDAR 240
AMX28327.1      TLGPGTSCLGRTVTRAHFSELTLTLMDLDLEMKHNVSSVFTVVEEGLFGRTYTVWRSDAR 240
AKR81281.1      TLGPGTSCLGRTVTRAHFSELTLTLMDLDLEMKHNVSSVFTVVEEGLFGRTYTVWRSDAR 240
ANS59483.1      TLGPGTSCLGRTVTRAHFSELTLTLMDLDLEMKHNVSSVFTVVEEGLFGRTYTVWRSDAR 240
AHG50444.1      TLGPGTGCQGRTVTRAHFSELTLTLMDLDLEMKHNVSSVFTVVEEGLFGRTYTVWRSDAR 240
AKG94169.1      TLGPGTGCQGRTVTRAHFSELTLTLMDLDLEMKHNVSSVFTVVEEGLFGRTYTVWRSDAR 240
ALO02851.1      TLGPGTGCQGRTVTRAHFSELTLTLMDLDLEMKHNVSSVFTVVEEGLFGRTYTVWRSDAR 240
ABY61986.1      TLGPGTGCQGRTVTRAHFSELTLTLMDLDLEMKHNVSSVFTVVEEGLFGRTYTVWRSDAR 240
CAD54790.2      TLGPGTGCQGRTVTRAHFSELTLTLMDLDLEMKHNVSSVFTVVEEGLFGRTYTVWRSDAR 240
ADN03213.1      TLGPGTGCQGRTVTRAHFSELTLTLMDLDLEMKHNVSSVFTVVEEGLFGRTYTVWRSDAR 240
ACN62119.1      TLGPGTGCQGRTVTRAHFSELTLTLMDLDLEMKHNVSSVFTVVEEGLFGRTYTVWRSDAR 240
ABY61988.1      TLGPGTGCQGRTVTRAHFSELTLTLMDLDLEMKHNVSSVFTVVEEGLFGRTYTVWRSDAR 240
AAS68031.1      TLGPGTGCQGRTVTRAHFSELTLTLMDLDLEMKHNVSSVFTVVEEGLFGRTYTVWRSDAR 240
AIL29370.1      TLGPGTGCLGRTVTRAHFSELTLTLMDLDLEMKHNVSSVFTVVEEGLFGRTYTVRRSDAR 240
ADJ05525.1      TLGPGTGCLGRTVTKAHFSELTLTLMDLDLEMKHNVSSVFTVVEEGLFGRTYTVWRSDAR 240
ASN63873.1      TLGPGTGCLGRTVTKAHFSELTLTLMDLDLEMKHNVSSVFTVVEEGLFGRTYTVWRSDAR 240
ARP51875.1      TLGPGTGCLGRTVTRAHFSELTMTLMDLDLEMKHNVSSVFTVVEEGLFGRTYTVWRSDAR 240
AJE30397.1      TLGPGTGCLGRTVTRAHFSELTMTLMDLDLEMKHNVSSVFTVVEEGLFGRTYTVWRSDAR 240
ALM55670.1      TLGPGTGCLGRTVTRAHFSELTMTLMDLDLEMKHNVSSVFTVVEEGLFGRTYTVWRSDAR 240
AJE30404.1      TLGPGTGCLGRTVTRAHFSELTMTLMDLDLEMKHNVSSVFTVVEEGLFGRTYTVWRSDAR 240
ASN63994.1      TLGPGTGCLGRTVTRAHFSELTMTLMDLDLEMKHNVSSVFTVVEEGLFGRTYTVWRSDAR 240
ASN64054.1      TLGPGTGCLGRTVTRAHFSELTMTLMDLDLEMKHNVSSVFTVVEEGLFGRTYTVWRSDAR 240
ASN64006.1      TLGPGTGCLGRTVTRAHFSELTMTLMDLDLEMKHNVSSVFTVVEEGLFGRTYTVWRSDAR 240
AKN58853.1      TLGPGTGCLGRTVTRAHFSELTMTLMDLDLEMKHNVSSVFTVVEEGLFGRTYTVWRSDAR 240
AJA39814.1      TLGPGTGCLGRTVTRAHFSELTMTLMDLDLEMKHNVSSVFTVVEEGLFGRTYTVWRSDAR 240
AIK97759.1      TLGPGTGCLGRTVTRAHFSELTMTLMDLDLEMKHNVSSVFTVVEEGLFGRTYTVWRSDAR 240
AJE30413.1      TLGPGTGCLGRTVTRAHFSELTMTLMDLDLEMKHNVSSVFTVVEEGLFGRTYTVWRSDAR 240
AIL54004.1      ILGPGSGCLGRTVTRAHFSELTLTLMDLDLEKKHNVSSVFTVVEEGLFGRTYTVWRFDAR 240
AGG09146.1      TLGPGTGCLGRTVTRARSSELTLTLMDLDIEMKHNVSSVFTVVEEGLFGRTYTVWRFDAR 240
ART66998.1      TLGPGTGCLGRTVTRARSSELTLTLMDLDLEMKHNVSSVFTVVEGGLFGRTYTVRRSDAR 240
AJT59441.1      TLGPGTSCLGRAVTRAQFSKLTLTLLDLDLEMKHNVSSVFTVVEEGLFGRTYTVWRSDAG 240
ABX75312.1      TLGPGTGCLGRTVTRAQFSELTLTLMDLDLEMKHNVSSVFTVVEEGLFGRTYTVWRSDTG 240
AKT04315.1      TLGPGTGCLGRTVTRAQFSELTLTLMDLDLEMKHNVSSVLTVVEEGLFGRTYTVWRSDTG 240
CAJ01700.1      TLGPGTGCLGRTVTRAQFSELTLTLMDLDLEIKHNVSSVFTVVEEGLFGRTYTVWRSDTG 240
ADX95995.1      TLGPGTGCLGRTVTRAQFSELTLTLMDLDLEMKHNVSSVFTVVEEGLFGRTYTVWRSDTG 240
AIK19904.1      TLGPGTGCLGRTVTRAQFSELTMTLMDLDLETKHNVSSVFTVVEEGLFGRTYIVWRSDTG 240
AID07002.1      TLGPGTGCLGRTVTRAQFSELTLTLMDLDLEMKHNVSSVFTVVEEGLFGRTYIVWRSDTG 240
AKT04307.1      TLGPGTGCLGRAVTRAQFSELTLTLMDLDLEMKHNVSSVFTVVEEGLFGRTYIVWRSDTR 240
ANG60369.1      TLGPGTGCLGRTVTRAQFSELTLTLMDLDLEMKHNVSSVFTVVEEGLFGRTYIVWRSDTR 240
                 *** :.* **:**:*: *:**:**:***:* *******:**** ******* * * *: 
```

```
ANG60361.1        DLSTDIDTGHFLRVFEIGLVRDLGLGPPVFHMTNYLTVNMSDDYRRCLLAVGGLKLTALC 300
AKQ09544.1        DPSTDLGIGHFLRVFEIGLVRDLGLGPPVFHMTNYLTVNMSDVYRRCLLAVGELKLTALC 300
AMX28327.1        DPSTDLGIGHFLRVFEIGLVRDLGLGPPVFHMTNYLTVNMSDVYRRCILAVGELKLTALC 300
AKR81281.1        DPSTDLGIGHFLRVFEIGLVRDLGLGPPVFHMTNYLTVNMSDVYRRCLLAVGELKLTALC 300
ANS59483.1        DPSTDLGIGHFLRVFEIGLVRDLGLGPPVFHMTNYLTVNMSDVYRRCLLAVGELKLTALC 300
AHG50444.1        DPSTDPGIGHFLRVFEIGLVRDLGLGPPVFHMTNYLTVNMSDDYRRCLLAVGELKLTALC 300
AKG94169.1        DPSTDPGIGHFLRVFEIGLVRDLGLGPPVFHMTNYLTVNMSDDYRRCLLAVGELKLTALC 300
ALO02851.1        DPSTDLGIGHFLRVFEIGLVRDLGLGPPVFHMTNYLTVNMSDVYRRCLLAVGELKLTALC 300
ABY61986.1        DPSTDPGIGHFLRVFEIGLVRDLGLGPPAFHMTNYLTVNMSDDYRRCLLAVGELKLTALC 300
CAD54790.2        DPSTDPGIGHFLRVFEIGLVRDLGLGPPVFHMTNYLTVNMSDDYRRCLLAVGELKLTALC 300
ADN03213.1        DPSTDPGIGHFLRVFEIGLVRDLGLGPPVFHMTNYLTVNMSDDYRRCLLAVGELKLTALC 300
ACN62119.1        DPSTDPGIGHFLRVFEIGLVRDLGLGPPVFHMTNYLTVNMSDDYRRCLLAVGELKLTALC 300
ABY61988.1        DPSTDPGIGHFLRVFEIGLVRDLGLGPPVFHMTNYLTVNMSDDYRRCLLAVGELKLTALC 300
AAS68031.1        DPSTDPGIGHFLRVFEIGLVRDLGLGPPVFHMTNYLTVNMSDDYRRCLLAVGELKLTALC 300
AIL29370.1        DPSTDLGIGHFLRVFEIGLVRDLGLGPPVFHMTNYLTVNMSDDYRRCLLAVGELKLTALC 300
ADJ05525.1        DPSTDLGIGHFLRVFEIGLIRDLGLGPPVFHMTNYLTVNMSDDYRRCLLAVGELKLTALC 300
ASN63873.1        DPSTDLGIGHFLRVFEIGLIRDLGLGPPVFHMTNYLTVNMSDDYRRCLLAVGELKLTALC 300
ARP51875.1        DPSADLGIGHFLRVFEIGLVRDFGLGPPVFHMTNYLTVNMSDDYRRCLLAVGELKLTALC 300
AJE30397.1        DPSTDLGIGHFLRVFEIGLVRDLGLGPPVFHMTNYLTVNMSDDYRRCLLAVGELKLTALC 300
ALM55670.1        DPSTDLGIGHFLRVFEIGLVRDLGLGPPVFHMTNYLTVNMSDDYRRCLLAVGELKLTALC 300
AJE30404.1        DPSTDLGIGHFLRVFEIGLVRDLGLGPPVFHMTNYLTVNMSDDYRRCLLAVGELKLTALC 300
ASN63994.1        DPSTDLGIGHFLRVFEIGLVRDLGLGPPVFHMTNYLTVNMSDDYRRCLLAVGELKLTALC 300
ASN64054.1        DPSTDLGIGHFLRVFEIGLVRDLGLGPPVFHMTNYLTVNMSDDYRRCLLAVGELKLTALC 300
ASN64006.1        DPSTDLGIGHFLRVFEIGLVRDLGLGPPVFHMTNYLTVNMSDDYRRCLLAVGELKLTALC 300
AKN58853.1        DPSTDLGIGHFLRVFEIGLVRDLGLGPPVFHMTNYLTVNMSDDYRRCLLAVGELKLTALC 300
AJA39814.1        DPSTDLGIGHFLRVFEIGLVSDLGLGPPVFHMTNYLTVNMSDDYRRCLLAVGELKLTALC 300
AIK97759.1        DPSTDLGIGHFLRVFEIGLVRDLGLGPPVFHMTNYLTVNMSDDYRRCLLAVGELKLTALC 300
AJE30413.1        DPSTDLGIGHFLRVFEIGLVRDLGLGPPVFHMTNYLTVNMSDDYRRCLLAVGELKLTALC 300
AIL54004.1        DPSTDLGIGHFLRVFEIGLVRDLGLGPPVFQMTNYLTVNMSDDYRRCLLAVGELKLTALC 300
AGG09146.1        DPSTDLGIGHFLRVFEIGLVRDLGLGPPVFHMTNYLTVNMSDDYRRCLLAVGELKLTALC 300
ART66998.1        DPSTDLGIGHFLRVFEIGLVRDLGLGPPVFQMTNYLTVNMSDDYRRCLLAVGELKLTALC 300
AJT59441.1        NPSTDPGTGHFLRVFEIGLVRDLGLSAPVFHMTNHLTVNMSDGYRSCLLAVGELKLAALC 300
ABX75312.1        KPSTSPGIGHFLRVFEIGLVRDLELGAPIFHMTNYLTVNMSDDYRSCLLAVGELKLTALC 300
AKT04315.1        KPSTSPGIGHFLRVFEIGLVRDLELGAPIFHMTNYLTVNMSDDYRSCLLAVGELKLTALC 300
CAJ01700.1        KPSTSPGIGHFLRVFEIGLVRDLELGAPIFHMTNYLTVNMSDDYRSCLLAVGELKLTALC 300
ADX95995.1        KPSTSPGIGHFLRVFEIGLVRDLELGAPIFHMTNYLTVNMSDDYRSCLLAVGELKLTALC 300
AIK19904.1        KPSTSLDIGQFLRVFEIGLVRDLELGAPIFHMTNYLTVNMSDDYRNCLLAVGELKLTALC 300
AID07002.1        KPSTSLDIGQFLRVFEIGLVRDLELGAPIFHMTNYLTVNMSDDYRSCLLAVGELKLTALC 300
AKT04307.1        KPSTSPVIGQFLRVFEIGLVRDLELGAPIFHMTNYLTVNMSDDYRSCLLAVGELKLTALC 300
ANG60369.1        KPSTSPDIGQFLRVFEIGLVRDLELGAPIFHMTNYLTVNMSDDYRSCLLAVGELKLTALC 300
                  . *:.   *:*********: *: *. * *:***:******* ** *:**** ***:***
```

```
ANG60361.1      TPSETVTLSERGVPKREPLVVVILNLAGPTLGGELYSVLPTSDLMVEKLYLSSHRGIIKD 360
AKQ09544.1      TSSETVTLSERGVPKRKPLVVVILNLAGPTLGGELYSILPTSDLMVEKLYLSSHRGIIKD 360
AMX28327.1      TSSETVTLSERGVPKREPLVVVILNLAGPTLGGELYSVLPTSDLMVEKLYLSSHRGIIKD 360
AKR81281.1      TSSETVTLSERGVPKREPLVVVILNLAGPTLGGELYSILPTSDLMVEKLYLSSHRGIIKD 360
ANS59483.1      TSSETVTLSERGVPKREPLVVVILNLAGPTLGGELYSVLPTSDLMVEKLYLSSHRGIIKD 360
AHG50444.1      TSSETVTLSERGVPKRKPLVVVILNLAGPTLEGELYSVLPTSDLMVEKLYLSSHRGIIKD 360
AKG94169.1      TSSETVTLGERGVPKRKPLVVVILNLAGPTLGGELYSVLPTSDLMVEKLYLSSHRGIIKD 360
ALO02851.1      TSSETVTLSERGVPKREPLVVVILNLAGPTLGGELYSVLPTSDLMVEKLYLSSHRGIIKD 360
ABY61986.1      TSSETVTLSERGVPKRKPLVVVILNLAGPTLGGELYSVLPTSDLMVEKLYLSSHRGIIKD 360
CAD54790.2      TSSETVTLSERGVPKRKPLVVVILNLAGPTLGGELYSVLPTSDLMVEKLYLSSHRGIIKD 360
ADN03213.1      TSSETVTLSERGVPKRKPLVVVILNLAGPTLGGELYSVLPTSDLMVEKLYLSSHRGIIKD 360
ACN62119.1      TSSETVTLSERGVPKRKPLVVVILNLAGPTLGGELYSVLPTSDLMVEKLYLSSHRGIIKD 360
ABY61988.1      TSSETVTLSERGVPKRKPLVVVILNLAGPTLGGELYSVLPTSDLMVEKLYLSSHRGIIKD 360
AAS68031.1      TSSETVTLSERGVPKRKPLVVVILNLAGPTLGGELYSVLPTSDLMVEKLYLSSHRGIIKD 360
AIL29370.1      TSSETVTLSERGVPKREPLVVVILNLAGPTLGGELYSILPTSDPMVEKLYLSSHRGIIKD 360
ADJ05525.1      TSSETVTLSERGAPKREPLVVVILNLAGPTLGGELYSVLPTSDLMVEKLYLSSHRGIIKD 360
ASN63873.1      TSSETVTLSERGAPKREPLVVVILNLAGPTLGGELYSVLPTSDLMVEKLYLSSHRGIIKD 360
ARP51875.1      TSSETVTLSERGVPRREPLVVVILNLAGPTLGGELYSVLPTSDLMVEKLYLSSHRGIIKD 360
AJE30397.1      TSSETVTLSERGVPRREPLVVVILNLAGPTLGGELYSVLPTSDLMVEKLYLSSHRGIIKD 360
ALM55670.1      TSSETVTLSERGVPRREPLVVVILNLAGPTLGGELYSVLPTSDLMVEKLYLSSHRGIIKD 360
AJE30404.1      TSSETVTLSERGVPRREPLVVVILNLAGPTLGGELYSVLPTSDLMVEKLYLSSHRGIIKD 360
ASN63994.1      TSSETVTLSERGVPRREPLVVVILNLAGPNLGGELYSVLPTSDLMVEKLYLSSHRGIIKD 360
ASN64054.1      TSSETVTLSERGVPRREPLVVVILNLAGPTLGGELYSVLPTSDLMVEKLYLSSHRGIIKD 360
ASN64006.1      TSSEAVTLSERGVPRREPLVVVILNLAGPTLGGELYSVLPTSDLMVEKLYLSSHRGIIKD 360
AKN58853.1      TSSETVTLSERGVPRREPLVVVILNLAGPTLGGELYSVSPTSDLMVEKLYLSSHRGIIKD 360
AJA39814.1      TSSETVTLSERGVPRREPLVVVILNLAGPTLGGELYSVLPTSDLMVEKLYLSSHRGIIKD 360
AIK97759.1      TSSETVTLSERGVPRREPLVVVILNLAGPTLGGELYSVLPTSDLMVEKLYLSSHRGIIKD 360
AJE30413.1      TSSETVTLSERGVPRREPLVVVILNLAGPTLGGELYSVLPTSDLMVEKLYLSSHRGIIKD 360
AIL54004.1      TSSETVTLSEGGVPKREPLAVVILNLVGPTLGGELYSVLPTSDLMVEKLYLSSHRGVIKD 360
AGG09146.1      TSSETVTLSERGVPKREPLVVVILNLAGPTLGGELYSVLPTSDLMVEKLYLSSHRGVIKD 360
ART66998.1      TSSETVTLSERGVPKREPLVVVILNLAGPTLGGELYSVLPTSDLMVEKLYLSSHRGVIKD 360
AJT59441.1      TPSETVTLSERGVPKREPLVVVILNLAGPTLGGELYSVLPTSDLTVEKLHLSSHRGIIKD 360
ABX75312.1      TPSETVTLSERGIPKREPLVVVILNLAGPTLGGELYSVLPTSDLMVEKLYLSSHRGIIKD 360
AKT04315.1      TPSETVTLSERGVPKREPLVVVILNLAGPTLGGELYSVLPTSDPTVEKLYLSSHRGIIKD 360
CAJ01700.1      TPSETVTLSESGVPKREPLVVVILNLAGPTLGGELYSVLPTTDPTVEKLYLSSHRGIIKD 360
ADX95995.1      TPSETVTLSESGVPKREPLVVVILNLAGPTLGGELYSVLPTTDPTVEKLYLSSHRGIIKD 360
AIK19904.1      TPSETVTLSERGVPKREPLVVVILNLVGPTLGGELYSVLPTSDLMVEKLHLSSHRGIIKD 360
AID07002.1      TPSETVTLSERGVPKREPLVVVILNLVGPTLGGELYSVLPTSDLMVEKLHLSSHRGIIKD 360
AKT04307.1      TPSETVTLSERGVPKREPLVVVILNLVGPTLGGELYSVLPTSDLMVEKLYLSSHRGIIKD 360
ANG60369.1      TPSETVTLSERGVPKREPLVVVILNLVGPTLGGELYSVLPTSDLTVEKLYLSSHRGIIKD 360
                * **:***.* * *:*:**.******.**.* *****: **:*  ****:******:***
```

```
ANG60361.1      DEASWVVPSTDVRDLQNKGECLVEACKTRPPSFCNGTGSGPWSEGRIPAYGVIRVSLDLA 420
AKQ09544.1      DEANWVVPSTDVRDLQNKGECLVEACKTRPPSFCNGTGSGPWSEGRIPAYGVIRVSLDLA 420
AMX28327.1      DEANWVVPSTDVRDLQNKGECLVEACKTRPPSFCNGTGSGPWSEGRIPAYGVIRVSLDLA 420
AKR81281.1      DEANWVVPSTDVRDLQNKGECLVEACKTRPPSFCNGTGSGPWSEGRIPAYGVIRVSLDLA 420
ANS59483.1      DEANWVVPSTDVRDLQNKGECLVEACKTRPPSFCNGTGSGPWSEGRIPAYGVIRVSLDLA 420
AHG50444.1      DEANWVVPSTDVRDLQNKGECLVEACKTRPPSFCNGTGSGPWSEGRIPAYGVIRVSLDLA 420
AKG94169.1      DEANWVVPSTDVRDLQNKGECLVEACKTRPPSFCNGTGSGPWSEGRIPAYGVIRVSLDLA 420
ALO02851.1      DEANWVVPSTDVRDLQNKGECLVEACKTRPPSFCNGTGSGPWSEGRIPAYGVIRVSLDLA 420
ABY61986.1      DEANWVVPSTDVRDLQNKGECLVEACKTRPPSFCNGTGSGPWSEGRIPAYGVIRVSLDSA 420
CAD54790.2      NEANWVVPSTDVRDLQNKGECLVEACKTRPPSFCNGTGSGPWSEGRIPAYGVIRVSLDLA 420
ADN03213.1      DEANWVVPSTDVRDLQNKGECLVEACKTRPPSFCNGTGSGPWSEGRIPAYGVIRVSLDSA 420
ACN62119.1      DEANWVVPSTDVRDLQNKGECLVEACKTRPPSFCNGTGSGPWSEGRIPAYGVIRVSLDSA 420
ABY61988.1      DEANWVVPSTDVRDLQNKGECLVEACKTRPPSFCNGTGSGPWSEGRIPAYGVIRVSLDSA 420
AAS68031.1      DEANWVVPSTDVRDLQNKGECLVEACKTRPPSFCNGTGSGPWSEGRIPAYGVIRVSLNSA 420
AIL29370.1      DEANWVVPSTDVRDLQNKGECLIEACKTRPPSFCNGTGSGPWSEGRIPAYGVIRVSLDLA 420
ADJ05525.1      DEANWVVPSTDVRDLQNKGECLVEACKTRPPSFCNGTGSGPWSEGRIPAYGVIRVSLDLA 420
ASN63873.1      DEANWVVPSTDVRDLQNKGECLVEACKTRPPSFCNGTGSGPWSEGRIPAYGVIRVSLDLA 420
ARP51875.1      DEANWVVPSTDVRDLQNKGECLVEACKTRPPSFCNGTGSGPWSEGRIPAYGVIRVSLDLA 420
AJE30397.1      DEANWVVPSTDVRDLQNKGECLVEACKTRPPSFSNGTGSGPWSEGRIPACGVIRVSLDLA 420
ALM55670.1      DEANWVVPSTDVRDLQNKGECLVEACKTRPPSFCNGTGSGPWSEGRIPAYGVIRVSLDLA 420
AJE30404.1      DEANWVVPSTDVRDLQNKGECLVEACKTRPPSFCNGTGSGPWSEGRIPAYGVIRVSLDLA 420
ASN63994.1      DEANWVVPSTDVRDLQNKGECLVEACKTRPPSFCNGTGSGPWSEGRIPAYGVIRVSLDLA 420
ASN64054.1      DEANWVVPSTDVRDLQNKGECLVEACKTRPPSFCNGTGSGPWSEGRIPAYGVIRVSLDLA 420
ASN64006.1      DEANWVVPSTDVRDLQNKGECLVEACKTRPPSFCNGTGSGPWSEGRIPAYGVIRVSLDLA 420
AKN58853.1      DEANWVVPSTDVRDLQNKGECLVEACKTRPPSFCNGTGSGPWSEGRIPAYGVIRVSLDLA 420
AJA39814.1      DEANWVVPSTDVRDLQNKGECLVEACKTRPPSFCNGTGSGPWSEGRIPAYGVIRVSLDLA 420
AIK97759.1      DEANWVVPSTDVRDLQNKGECLVEACKTRPPSFCNGTGSGPWSEGRIPAYGVIRVSLDLA 420
AJE30413.1      DEANWVVPSTDVRDLQNKGECLVEACKTRPPSFCNGTGSGPWSEGRIPAYGVIRVSLDLA 420
AIL54004.1      DEANWVVPSTDVRDLQNKGECLVEACKTRPPSFCNGTGSGPWSEGRIPAYGVIRVSLDLA 420
AGG09146.1      DEANWVVPSTDVRDLQNKGECLVEACKTRPPSFCNGTGSGPWSEGRIPAYGVIRVSLDLA 420
ART66998.1      DEANWVVPSTDVRDLQNKGECLVEACKTRPPSFCNGTGSGPWSEGRIPAYGVIRVSLDLA 420
AJT59441.1      NEANWVVPSTDVRDLQNKGECLVEACKTRPPSFCNGTGVGPWSEGRIPAYGVIRVSLDLA 420
ABX75312.1      NEANWVVPSTDVRDLQNKGECLVEACKTRPPSFCNGTGIGPWSEGRIPAYGVIRVSLDLA 420
AKT04315.1      NEANWVVPSTDVRDLQNKGECLVEACKTRPPSFCNGTGIGPWSEGRIPAYGVIRVSLDLA 420
CAJ01700.1      NEANWVVPSTDVRDLQNKGECLVEACKTRPPSFCNGTGIGPWSEGRIPAYGVIRVSLDLA 420
ADX95995.1      NEANWVVPSTDVRDLQNKGECLVEACKTRPPSFCNGTGIGPWSEGRIPAYGVIRVSLDLA 420
AIK19904.1      NEANWVVPSTDVRDLQNKGECLVEACKTRPPSFCNGTGIGPWSEGRIPAYGVIRVSLDLA 420
AID07002.1      NEANWVVPSTDVRDLQNKGECLVEACKTRPPSFCNGTGIGPWSEGRIPAYGVIRVSLDLA 420
AKT04307.1      NEANWVVPSTDVRDLQNKGECLVEACKTRPPSFCNGTGIGPWSEGRIPAYGVIRVSLDLA 420
ANG60369.1      NEANWVVPSTDVRDLQNKGECLVEACKTRPPSFCNGTGIGPWSEGRIPAYGVIRVSLDLA 420
                :**.******************:**********.**** ********** *******: *
```

```
ANG60361.1      RDPGVVINSVFGPLIPHLSGMDLYNNPFSRAVWLAVPPYEQSFLGMINTIGFPNRAEVMP 480
AKQ09544.1      SDPDVVITSVFGPLIPHLSGMDLYNNPFSRAVWLAVPPYEQSFLGMINTIGFPNRTEVMP 480
AMX28327.1      SDPDVVITSVFGPLIPHLSGMDLYNNPFSRAVWLAVPPYEQSFLGMINTIGFPNRTEVMP 480
AKR81281.1      SDPDVVITSVFGPLIPHLSGMDLYNNPFSRAVWLAVPPYEQSFLGMINTIGFPNRTEVMP 480
ANS59483.1      SDPDVVITSVFGPLIPHLSGMDLYNNPFSRAVWLAVPPYEQSFLGMINTIGFPNRTEVMP 480
AHG50444.1      SDPGVVITSVFGPLIPHLSGMDLYNNPFSRAVWLAVPPYEQSFLGMINTIGFPNRAEVMP 480
AKG94169.1      SDPGVVITSVFGPLIPHLSGMDLYNNPFSRAVWLAVPPYEQSFLGMINTIGFPNRAEVMP 480
ALO02851.1      SDPDVVITSVFGPLIPHLSGMDLYNNPFSRAVWLAVPPYEQSFLGMINTIGFPNRAEVMP 480
ABY61986.1      SDPGVVITSVFGPLIPHLSGMDLYNNPFSRAVWLAVPPYEQSFLGMINTIGFPNRAEVMP 480
CAD54790.2      SDPGVVITSVFGPLIPHLSGMDLYNNPFSRAVWLAVPPYEQSFLGMINTIGFPNRAEVMP 480
ADN03213.1      SDPGVVITSVFGPLIPHLSGMDLYNNPFSRAVWLAVPPYEQSFLGMINTIGFPNRAEVMP 480
ACN62119.1      SDPGVVITSVFGPLIPHLSGMDLYNNPFSRAVWLAVPPYEQSFLGMINTIGFPNRAEVMP 480
ABY61988.1      SDPGVVITSVFGPLIPHLSGMDLYNNPFSRAVWLAVPPYEQSFLGMINTIGFPNRAEVMP 480
AAS68031.1      SDPGVVITSVFGPLIPHLSGMDLYNNPFSRAVWLAVPPYEQSFLGMINTIGFPNRAEVMP 480
AIL29370.1      SDPDVVITSVFGPLIPHLSGMDLYNNPFSGAVWLAVPPYEQSFLGMINTIGFPNRAEVMP 480
ADJ05525.1      SDPDVVITSVFGPLIPHLSGMDLYNNPFSRAIWLAVPPYEQSFLGMINTIGFPNRAEVMP 480
ASN63873.1      SDPDVVITSVFGPLIPHLSGMDLYNNPFSRAIWLAVPPYEQSFLGMINTIGFPNRAEVMP 480
ARP51875.1      SDPDVVITSVFGPLIPHLSGMDLYNNPFSKAVWLAVPPYEQSFLGMINTIGFPNRAEVMP 480
AJE30397.1      SDPDVVITSVFGPLIPHLSGMDLYNNPFSKAVWLAVPPYEQSFLGMINTIGFPNRAEVMP 480
ALM55670.1      SDPDVVITSVFGPLIPHLSGMDLYNNPFSKAVWLTVPPYEQSFLGMINTIGFPNRAEVMP 480
AJE30404.1      SDPDVVITSVFGPLIPHLSDMDLYNNPFSKAVWLAVPPYEQSFLGMINTIGFPNRAEVMP 480
ASN63994.1      SDPDVVITSVFGPLIPHPSGMDLYNNPFSKAVWLAVPPYEQSFLGMINTIGFPNRAEVMP 480
ASN64054.1      SDPDVVITSVFGPLIPHLSGMDLYNNPFSKAVWLAVPPYEQSFLGMINTIGFPNRAEVMP 480
ASN64006.1      SDPDVVITSVFGPLIPHLSGMDLYNNPFSKAVWLAVPPYEQSFLGMINTIGFPNRAEVMP 480
AKN58853.1      SDPDVVITSVFGPLIPHLSGMDLYNNPFSKAVWLAVPPYEQSFLGMINTIGFPNRAEVMP 480
AJA39814.1      SDPDVVITSVFGPLIPHLSGMDLYNNPFSKAVWLAVPPYEQSFLGMINTIGFPNRAEVMP 480
AIK97759.1      SDPDVVITSVFGPLIPHPSGMDLYNNPFSKAVWLAVPPYEQSFLGMINTIGFPNRAEVMP 480
AJE30413.1      SDPDVVITSVFGPLIPHLSGMDLYNNPFSKAVWLAVPPYEQSFLGMINTIGFPNRAEVMP 480
AIL54004.1      SDPGVVITSVFGPLIPHLSGMDLYNNPFSRAVWLAVPPYEQSFLGMINTIGFPNRAEVMP 480
AGG09146.1      SDPGVVITSVFGPLIPHLSGMDLYNNPFSRDVWLAVPPYEQSFLGMINTVGFPNRAEVMP 480
ART66998.1      TDPGVVITSVFGPLIPHLSGMDLYNNPFSRAVWLAVPPYEQSFLGMINTIGFPNRAEVMP 480
AJT59441.1      SDPDVVITSVFGPLIPHLSGMDLYNNPFSSAVWLAVPPYEQSYLGMINTIGLPNRAEVMP 480
ABX75312.1      SDPGVVITSVFGPLIPHLSGMDLYNNPFSRAAWLAVPPYEQSFLGMINTIGFPDRVEVMP 480
AKT04315.1      SDPGVVITSVFGPLIPHLSGMDLYNNPFSRAAWLAVPPYEQSFLGMINTIGFPDRVEVMP 480
CAJ01700.1      SDPGVVITSVFGPLIPHLSGMDLYNNPFSRAAWLAVPPYEQSFLGMINTIGFPDRAEVMP 480
ADX95995.1      SDPGVVITSVFGPLIPHLSGMDLYNNPFSRAAWLAVPPYEQSFLGMINTIGFPDRAEVMP 480
AIK19904.1      SDPGVVITSVFGPLIPHLSGMDLYNNPFSRAAWLAVPPYEQSFLGMINTIGFPDRAEVMP 480
AID07002.1      SDPGVVITSVFGPLIPHLSGMDLYNNPFSRAAWLAVPPYEQSFLGMINTIGFPDRAEVMP 480
AKT04307.1      SDPGVVITSVFGPLIPHLSGMDLYNNPFSRGAWLAVPPYEQSFLGMINTIDFPDRAEVMP 480
ANG60369.1      SDPGVVITSVFGPLIPHLSGMDLYNNPFSRGAWLAVPPYEQSFLGMINTIGFPDRAEVMP 480
                 **.***.********* *.*********   **:*******:******:.:*:*.****
```

```
ANG60361.1      HILTTEIRGPRGRCHVPIELSRRVDDDIKIGSNMVILPTKDLRYITATYDVSRSEHAIVY 540
AKQ09544.1      HILTTEIRGPRGHCHVPIELSRRVDDDIKIGSNMVILPTMDLRYITATYDVSRSEHAIVY 540
AMX28327.1      HILTTEIRGPRGHCHVPIELSRRVDDDIKIGSNMVILPTMDLRYITATYDVSRSEHAIVY 540
AKR81281.1      HILTTEIRGPRGHCHVPIELSRRVDDDIKIGSNMVILPTMDLRYITATYDVSRSEHAIVY 540
ANS59483.1      HILTTEIRGPRGHCHVPIELSRRVDDDIKIGSNMVILPTMDLRYITATYDVSRSEHAIVY 540
AHG50444.1      HILTTEIRGPRGRCHVPIELSRRVDDDIKIGSNMVILPTMDLRYITATYDVSRSEHAIVY 540
AKG94169.1      HILTTEIRGPRGRCHVPIELSRRVDDDIKIGSNMVILPTMDLRYITATYDVSRSEHAIVY 540
ALO02851.1      HILTTEIRGPRGRCHVPIELSRRVDDDIKIGSNMVILPTMDLRYITATYDVSRREHAIVY 540
ABY61986.1      HILTTEIRGPRGRCHVPIELSRRVDDDIKIGSNMVILPTMDLRYITATYDVSRREHAIVY 540
CAD54790.2      HILTTEIRGPRGRCHVPIELSRRVDDDIKIGSNMVILPTMDLRYITATYDVSRREHAIVC 540
ADN03213.1      HILTTEIRGPRGRCHVPIELSRRVDDDIKIGSNMVILPTMDLRYITATYDVSRREHAIVY 540
ACN62119.1      HILTTEIRGPRGRCHVPIELSRRVDDDIKIGSNMVILPTMDLRYITATYDVSRREHAIVY 540
ABY61988.1      HILTTEIRGPRGRCHVPIELSRRVDDDIKIGSNMVILPTMDLRYITATYDVSRREHAIVY 540
AAS68031.1      HILTTEIRGPRGRCHVPIELSRRVDDDIKIGSNMVILPTMDLRYITATYDVSRREHAIVY 540
AIL29370.1      HILTTEIRGPRGRCHVPIELSRRVDDDIKIGSNMVILPTMDLRYITATYDVSRSEHAIVY 540
ADJ05525.1      HILTTEIRGPRGRCHVPIELSRRVDDDIKIGSNMVILPTMDLRYITATYDVSRSEHAIVY 540
ASN63873.1      HILTTEIRGPRGRCHVPIELSRRVDDDIKIGSNMVILPTMDLRYITATYDVSRSEHAIVY 540
ARP51875.1      HILTTEIRGPRGRCHVPIELSRRVDNDIKIGSNMVILPTMDLRYITATYDVSRSEHAIVY 540
AJE30397.1      HILTTEIRGPRGRCHVPIELSRRVDDDIKIGSHMVILPTMDLRYITATYDVSRSEHAIVY 540
ALM55670.1      HILTTEIRGPRGRCHVPIELSRRVDDDIKIGSNMVILPTMDLRYITATYDVSRSEHAIVY 540
AJE30404.1      HILTTEIRGPRGRCHVPIELSRRVDDDIKIGSNMVILPTMDLRYITATYDVSRSEHAIVY 540
ASN63994.1      HILTTEIRGPRGRCHVPIELSRRVDDDIKIGSNMVILPTMDLRYITATYDVSRSEHAIVY 540
ASN64054.1      HILTTEIRGPRGRCHVPIELSRRVDDDIKIGSNMVILPTMDLRYITATYDVSRSEHAIVY 540
ASN64006.1      HILTTEIRGPRGRCHVPIELSRRVDDDIKIGSNMVILPTMDLRYITATYDVSRSEHAIVY 540
AKN58853.1      HILTTEIRGPRGRCHVPIELSRRVDDDIKIGSNMVILPTMDLRYITATYDVSRSEHAIVY 540
AJA39814.1      HILTTEIRGPRGRCHVPIELSRRVDDDIKIGSNMVILPTMDLRYITATYDVSRSEHAIVY 540
AIK97759.1      HILTTEIRGPRGRCHVPIELSRRVDDDIKIGSNMVILPTMDLRYITATYDVSRSEHAIVY 540
AJE30413.1      HILTTEIRGPRGRCHVPIELSRRVDDDIKIGSNMVILPTMDLRYITATYDVSRSEHAIVY 540
AIL54004.1      HILTTEIRGPRGRCHVPIELSRRVDDDIKIGSNMVILPTMDLRYITATYDVSRSEHAIVY 540
AGG09146.1      HILTTEIRGPRGRCHVPIELSRRVDDDIKIGSNMVILPTMDLRYITATYDVSRSEHAIVY 540
ART66998.1      HILTTEIRGPRGRCHVPIELSRRVDDDIKIGSNMVILPTMDLRYITATYDVSRSEHAIVY 540
AJT59441.1      HILTTEIKGPRGRCHVPIELSRRIDDDIKIGSNMVVLPTKDLRYITATYDVSRSEHAIVY 540
ABX75312.1      HILTTEIRGPRGRCHIPIELSRRIDDDIKIGSNMVVLPTKDLRYITATYDVSRSEHAIVY 540
AKT04315.1      HILTTEIRGPRGRCHVPIELSRRIDDDIKIGSNMVVLPTKDLRYITATYDVSRSEHAIVY 540
CAJ01700.1      HILTTEIRGPRGRCHVPIELSSRIDDDIKIGSNMVVLPTKDLRYITATYDVSRSEHAIVY 540
ADX95995.1      HILTTEIRGPRGRCHVPIELSSRIDDDIKIGSNMVVLPTKDLRYITATYDVSRSEHAIVY 540
AIK19904.1      HILTTEIKGPRGRCHVPIELSRRIDDDIKIGSNMVVLPTRDLRYITATYDVSRSEHAIVY 540
AID07002.1      HILTTEIKGPRGRCHVPIELSRRIDDDIKIGSNMVVLPTRDLRYITAAYDVSRSEHAIVY 540
AKT04307.1      HILTTEIRGPRGRCHVPIELSRRIDDDIKIGSNMVVLPTRDLRYVTATYDVSRSEHAIVY 540
ANG60369.1      HILTTEIRGPRGRCHVPIELSRRIDDDIKIGSNMVVLPTRDLRYVTATYDVSRSEHAIVY 540
                *******:****:**:***** *:*:******:**:*** ****:**:***** *****
```

```
ANG60361.1      YIYDTGRSSSYFYPVRLNFKGNPLSLRIECFPWHHKVWCYHDCLIYNTTSGEEVHTRGLT 600
AKQ09544.1      YIYDTGRSSSYFYPVRLNFKGNPLSLRIECFPWRHKVWCYHDCLIYNTITDEEVHMRGLT 600
AMX28327.1      YIYDTGRSSSYFYPVRLNFKGNPLSLRIECFPWRHKVWCYHDCLIYNTITDEEVHMRGLT 600
AKR81281.1      YIYDTGRSSSYFYPVRLNFKGNPLSLRIECFPWRHKVWCYHDCLIYNTITDEEVHMRGLT 600
ANS59483.1      YIYDTGRSSSYFYPVRLNFKGNPLSLRIECFPWRHKVWCYHDCLIYNTITDEEVHMRGLT 600
AHG50444.1      YIYDTGRSSSYFYPVRLNFKGNPLSLRIECFPWRHKVWCYHDCLIYNTITDEEVHTRGLT 600
AKG94169.1      YIYDTGRSSSYFYPVRLNFKGNPLSLRIECFPWRHKVWCYHDCLIYNTITDEEVHTRGLT 600
ALO02851.1      YIYDTGLSSSYYYPVRLNFKGNPLSLRIECFPWRHKVWCYHDCLIYNTITDEEVHTRGLT 600
ABY61986.1      YIYDTGLSSSYYYPVRLNFKGNPLSLRIECFPWRHKVWCYHDCLIYNTITDEEVHTRGLT 600
CAD54790.2      YIYDTGLSSSYYYPVRLNFKGNPLSLRIECFPWRHKVWCYHDCLIYNTITDEEVHTRGLT 600
ADN03213.1      HIYDTGLSSSYYYPVRLNFKGNPLSLRIECFPWRHKVWCYHDCLIYNTITDEEVHTRGLT 600
ACN62119.1      YIYDTGLSSSYYYPVRLNFKGNPLSLRIECFPWRHKVWCYHDCLIYNTITDEEVHTRGLT 600
ABY61988.1      YIYDTGLSSSYYYPVRLNFKGNPLSLRIECFPWRHKVWCYHDCLIYNTITDEEVHTRGLT 600
AAS68031.1      YIYDTGLSSSYYYPVRLNFKGNPLSLRIECFPWRHKVWCYHDCLIYNTITDEEVHTRGLT 600
AIL29370.1      YIYDTGRSSSYFYPVRLNFKGNPLSLRIECFPWRQKVWCYHDCLIYNTITDEEVHTRGLT 600
ADJ05525.1      YIYDTGRSSSYFYPVRLNFKGNPLSLRIECFPWRHKVWCYHDCLIYNTITDEEVHTRGLT 600
ASN63873.1      YIYDTGRSSSYFYPVRLNFKGNPLSLRIECFPWRHKVWCYHDCLIYNTITDEEVHTRGLT 600
ARP51875.1      YIYDTSRSSSYFYPVRLNFKGNPLSLRIECFPWRHKVWCYHDCLIYNTITGEEVHTRGLT 600
AJE30397.1      YIYDTSRSSSYFYPVRLNFKGNPLSLRIECFPWRHKVWCYHDCLIYNTITGEEVHTRGLT 600
ALM55670.1      YIYDTSRSSSYFYPVRLNFKGNPLSLRIECFPWRHKVWCYHDCLIYNTITNEEVHTRGLT 600
AJE30404.1      YIYDTSRSSSYFYPVRLNFKGNPLSLRIECFPWRRKVWCYHDCLIYNTITGEEVHTRGLT 600
ASN63994.1      YIYDTSRSSSYFYPVRLNFKGNPLSLRIECFPWRHKVWCYHDCLIYNTITGEEVHTRGLT 600
ASN64054.1      YIYDTSRSSSYFYPVRLNFKGNPLSLRIECFPWRHKVWCYHDCLIYNTITGEEVHTRGLT 600
ASN64006.1      YIYDTSRSSSYFYPVRLNFKGNPLSLRIECFPWRHKVWCYHDCLIYNTITGEEVHTRGLT 600
AKN58853.1      YIYDTSRSSSYFYPVRLNFKGNPLSLRIECFPWRHKVWCYHDCLIYNTITGEEVHTRGLT 600
AJA39814.1      YIYDTSRSSSYFYPVRLNFKGNPLSLRIECFPWRHKVWCYHDCLIYNTITGEEVHTRGLT 600
AIK97759.1      YIYDTSRSSSYFYPVRLNFKGNPLSLRIECFPWRHKVWCYHDCLIYNTITGEEVHTRGLT 600
AJE30413.1      YIYDTSRSSSYFYPVRLNFKGNPLSLRIECFPWRHKVWCYHDCLIYNTITGEEVHTRGLT 600
AIL54004.1      YIYDTGRSSSYFYPVRLNFKGNPLSLRIECFPWRHKVWCYHDCLIYNTITDEEVHARGLT 600
AGG09146.1      YIYDTGRSSSYFYPVRLNFKGNPLSLRIECFPWRHKVWCYHDCLIYNTITDEEVHTRGLT 600
ART66998.1      YIYDTGRSSSYFYPVRLNFKGNPLSLRIECFPWRHKVWCYHDCLIYNTITDEEVHTRGLT 600
AJT59441.1      YIYDTGRSSSYFYPARLKFKGNPLSLRIECFPWHHKVWCYHDCLIYNTITNEEVHKRGLI 600
ABX75312.1      YIYDTGRSSSYFYPVRLNFRGNPLSLRIECFPWYHKVWCYHDCLIYNTITNEEVHMRGLT 600
AKT04315.1      YIYDTGRSSSYFYPVRLNFKGNPLSLRIECFPWYHKVWCYHDCLIYNTITNEEVHTRGLT 600
CAJ01700.1      YIYDTGRSSSYFYPVRLNFRGNPLSLRIECFPWYHKVWCYHDCLIYNTITNEEVHTRGLT 600
ADX95995.1      YIYDTGRSSSYFYPVRLNFRGNPLSLRIECFPWYHKVWCYHDCLIYNTITNEEVHTRGLT 600
AIK19904.1      YIYDTGRSSSYFYPVRLNFKGNPLSLRIECFPWSHKVWCYHDCLIYNTMTNEEVHTRGLT 600
AID07002.1      YIYDTGRSSSYFYPVRLNFKGNPLSLRIECFPWSHKVWCYHDCLIYNTMTNEEVHTRGLT 600
AKT04307.1      YIYDTGRSSSYFYPVRLNFRGNPLSLRIECFPWSHKVWCYHDCLIYNTMTNEEVHTRGLT 600
ANG60369.1      YIYDTDRSSSYFYPVRLNFRGNPLSLRIECFPWSHKVWCYHDCLIYNTMTNEEVHTRGLT 600
                :****. ****:**.**:*:************* :************* :.**** ***
```

```
ANG60361.1      GIEVTCNPA 609
AKQ09544.1      GIEVTCNPV 609
AMX28327.1      GIEVTCNPV 609
AKR81281.1      GIEVTCNPV 609
ANS59483.1      GIEVTCNPV 609
AHG50444.1      GIEVTCNPV 609
AKG94169.1      GIEVTCNPV 609
ALO02851.1      GIEVTCNPV 609
ABY61986.1      GIEVTCIQS 609
CAD54790.2      GIEVTCNPV 609
ADN03213.1      GIEVTCNPV 609
ACN62119.1      GIKVTCNPV 609
ABY61988.1      GIEVTCNPV 609
AAS68031.1      GIEVTCNPV 609
AIL29370.1      GIEVTCNPV 609
ADJ05525.1      GIEVTCNPV 609
ASN63873.1      GIEVTCNPV 609
ARP51875.1      GIEVTCNPV 609
AJE30397.1      GIEVTCNPV 609
ALM55670.1      GIEVTCNPV 609
AJE30404.1      GIEVTCNPV 609
ASN63994.1      GIEVTCNPV 609
ASN64054.1      GIEVTCNPV 609
ASN64006.1      GIEVTCNPV 609
AKN58853.1      GIEVTCNPV 609
AJA39814.1      GIEVTCNPV 609
AIK97759.1      GIEVTCNPV 609
AJE30413.1      GIEVTCNPV 609
AIL54004.1      GIEVTCNPV 609
AGG09146.1      GIEVTCNPV 609
ART66998.1      GIEVTCNPV 609
AJT59441.1      GIEVTCNPA 609
ABX75312.1      GIEVTCNPV 609
AKT04315.1      GIEVTCNPV 609
CAJ01700.1      GIEVTCNPV 609
ADX95995.1      GIEVTCNPV 609
AIK19904.1      GIEVTCNPV 609
AID07002.1      GIEVTCNPV 609
AKT04307.1      GIEVTCNPV 609
ANG60369.1      GIEVTCDPV 609
                **:***
```

Supplementary file 2: Multiple sequence alignment of PPRV-H sequences to identify amino acid substitutions
