# Supplementary material for: Structural and functional characterization of peste des petits ruminants virus coded hemagglutinin protein using various in-silico approaches
Source: Front Microbiol. 2024 Jun 20;15:1427606. doi: 10.3389/fmicb.2024.1427606 (PMC11222573; doi:10.3389/fmicb.2024.1427606)
Supplement: Supplementary file 3 [file Data_Sheet_3.PDF]

Supplementary file 3 (A): Amino acid substitutions identified in PPRV-H protein sequences using MSA which were further subjected to PentUnFOLD analysis.

E155G
V160K/D
P170S
K176R
S179L
R240G
T233I
D283V
G264E
P267A
V269I
S302P
L344P
M345T
S399I
R450K
V452A
M520 K/R
G546S
R574Y/S
I589M
T596M

Supplementary file 3 (B): Prediction obtained from PentUnFOLD algorithm

№	AA	user input	SS	user input	HELIX	SHEET	Disordered coil	Disordered helix	Disordered sheet	Completely disordered coil	Completely disordered helix	Completely disordered sheet
130	E		C									
131	Y		C									
132	D		C				CE					
133	F		C									
134	R		C									
135	D		C				CH					
136	L		C									
137	R		C				CE					
138	W		C				CH					
139	C		C									
140	M		C				C					
141	N		C									
142	P		C									
143	P		C									
144	E		C									
145	R		H		HN			D				
146	V		H		HN							
147	K		H		HN							
148	I		H		HN							
149	N		H		HN							
150	F		H		HN							
151	D		H		HN							
152	Q		H		HN							
153	F		H		HS							
154	C		H		HS							
155	E		H		HN			D				
156	Y		H		HN							
157	K		H		HS			D				
158	A		H		HS							
159	A		H		HS							
160	V		H		HN			D				
161	K		H		HS							
162	S		H		HN			D				
163	I		H		HS			D				
164	E		H		HN							
165	H		H		HS							
166	I		H		HS							
167	F		H		HN							
168	E		H		HN							
169	S		H		HN							
170	P		H		HN							
171	L		H		HN							
172	N		H		HN			D			D	
173	K		H		HN			D				
174	S		H		HN			D			D	
175	K		H		HN			D				
176	K		H		HN			D				
177	L		H		HS			D				

178	Q	H	HN				
179	S	H	HN				
180	L	C					
181	T	C			CE		
182	L	C					
183	G	C					
184	P	C					
185	G	C					
186	T	C					
187	G	C					
188	C	C					
189	Q	C					
190	G	C			CE		
191	R	C					
192	T	C					
193	V	E		ES			
194	T	E		EN			
195	R	E		EN			
196	A	E		EN			D
197	H	E		EN			
198	F	E		EN			
199	S	E		EN			
200	E	C			CH		
201	L	C					
202	T	C					
203	L	C			CE		
204	T	C					
205	L	C					
206	M	H	HS				
207	D	H	HS			D	
208	L	H	HS				
209	D	H	HN				
210	L	H	HS				
211	E	H	HS				
212	M	C					
213	K	C					
214	H	C					
215	N	C					
216	V	E		ES			
217	S	E		ES			
218	S	C					
219	V	C					
220	F	E		ES			
221	T	E		ES			
222	V	E		ES			
223	V	E		EN			D
224	E	C					
225	E	C					
226	G	C			CH		

227	L	E	EN			
228	F	E	ES			
229	G	E	ES			
230	R	E	ES		D	
231	T	E	EN			
232	Y	E	EN		D	
233	T	E	ES			
234	V	E	ES			
235	W	E	ES			
236	R	C		CE		
237	S	C		CH		
238	D	C		D		
239	A	C				
240	R	C		D		D
241	D	C				
242	P	C				
243	S	C		D		
244	T	C				
245	D	C		D		
246	P	C				
247	G	C				
248	I	C				
249	G	C				
250	H	C		CE		
251	F	E	ES		D	
252	L	E	ES			
253	R	E	ES			
254	V	E	ES			
255	F	E	ES			
256	E	E	ES			
257	I	E	ES			
258	G	E	ES			
259	L	E	ES			
260	V	E	ES			
261	R	E	ES			
262	D	C				
263	L	C				
264	G	C				
265	L	C				
266	G	C				
267	P	C				
268	P	C				
269	V	E	ES			
270	F	E	ES			
271	H	E	ES			
272	M	E	EN			
273	T	C				
274	N	E	ES			
275	Y	E	EN			

276	L	E	ES		
277	T	E	ES		
278	V	C		CE	
279	N	C		CE	
280	M	C			
281	S	C		D	
282	D	C			
283	D	C		D	
284	Y	C			
285	R	C			
286	R	E	ES		
287	C	E	ES		
288	L	C			
289	L	E	ES		
290	A	E	ES		
291	V	E	EN		
292	G	C			
293	E	C		CE	
294	L	C			
295	K	E	ES		
296	L	E	ES		
297	T	E	ES		
298	A	E	ES		
299	L	E	ES		
300	C	E	ES		
301	T	E	EN		
302	S	C		D	
303	S	C		D	
304	E	C			
305	T	E	EN		
306	V	E	EN		D
307	T	E	ES		
308	L	C		CE	
309	S	C			
310	E	C			
311	R	C		D	
312	G	C			
313	V	C			
314	P	C			
315	K	C		CE	
316	R	C			
317	K	C			
318	P	C			
319	L	E	ES		
320	V	E	ES		
321	V	E	ES		
322	V	E	ES		
323	I	E	ES		
324	L	E	ES		

325	N	E	ES			
326	L	C		CH		
327	A	C				
328	G	C				
329	P	C		C		
330	T	C				
331	L	C				
332	G	C				
333	G	E	ES		D	
334	E	E	EN			
335	L	E	EN		D	
336	Y	E	ES			
337	S	E	ES			
338	V	C				
339	L	C		CE		
340	P	C				
341	T	C				
342	S	C				
343	D	C				
344	L	C				
345	M	C				
346	V	C		CH		
347	E	C		CH		
348	K	E	ES		D	
349	L	E	EN			
350	Y	E	EN			
351	L	C		CH		
352	S	C		CE		
353	S	C				
354	H	C		D		
355	R	E	EN		D	
356	G	E	ES		D	
357	I	E	ES		D	
358	I	E	ES			
359	K	C		CE		
360	D	C		D		
361	D	C		D		
362	E	E	EN		D	D
363	A	E	EN			
364	N	E	ES			
365	W	E	ES			
366	V	E	ES			
367	V	E	ES			
368	P	E	ES			
369	S	E	EN			
370	T	E	EN			
371	D	C				
372	V	C				
373	R	C				

374	D	H	HN					
375	L	H	HN					
376	Q	H	HN			D		
377	N	H	HN					
378	K	H	HN			D		
379	G	H	HN			D		
380	E	H	HN			D		
381	C	H	HS					
382	L	H	HS					
383	V	C						
384	E	C						
385	A	C						
386	C	C			CH			
387	K	C						
388	T	C						
389	R	C						
390	P	C						
391	P	C						
392	S	C						
393	F	C						
394	C	C						
395	N	C						
396	G	C						
397	T	C						
398	G	C						
399	S	C						
400	G	C						
401	P	C						
402	W	C						
403	S	C						
404	E	C						
405	G	C						
406	R	C						
407	I	C						
408	P	C			CE			
409	A	E		ES				
410	Y	E		ES				
411	G	E		ES				
412	V	E		ES				
413	I	E		ES			D	
414	R	E		ES				
415	V	E		ES				
416	S	E		ES				
417	L	C			CE			
418	N	C						
419	S	C			CH			
420	A	C						
421	S	C			D			D
422	D	C						

423	P	C			
424	G	C			
425	V	E	ES		
426	V	E	ES		
427	I	E	ES		
428	T	E	ES		
429	S	E	ES		
430	V	E	ES		
431	F	C			
432	G	C			
433	P	C			
434	L	C			
435	I	C			
436	P	C			
437	H	C		CE	
438	L	C			
439	S	C			
440	G	C			
441	M	C			
442	D	E	EN		
443	L	E	EN		
444	Y	E	EN		
445	N	E	ES		
446	N	C			
447	P	C			
448	F	C			
449	S	C			
450	R	C			
451	A	E	ES		
452	V	E	ES		
453	W	E	ES		
454	L	E	ES		
455	A	E	ES		
456	V	E	ES		
457	P	C			
458	P	C			
459	Y	C			
460	E	C			
461	Q	C		CH	
462	S	C			
463	F	C		CH	
464	L	C			
465	G	C		CE	
466	M	E	ES		
467	I	E	ES		
468	N	E	ES		
469	T	E	EN		D
470	I	E	EN		
471	G	E	ES		

472	F	C				
473	P	C				
474	N	C				
475	R	C				
476	A	C				
477	E	C				
478	V	E	ES			
479	M	E	EN		D	
480	P	C				
481	H	C		CE		
482	I	C		CE		
483	L	C				
484	T	C		CE		
485	T	C				
486	E	C				
487	I	C		CE		
488	R	C				
489	G	C				
490	P	C				
491	R	C				
492	G	C				
493	R	C		CE		
494	C	C		CE		
495	H	C		CE		
496	V	C				
497	P	C		CE		
498	I	C				
499	E	C				
500	L	C				
501	S	C				
502	R	C				
503	R	C				
504	V	C				
505	D	C		D		
506	D	C				
507	D	C		D		
508	I	C		CE		
509	K	C		CE		
510	I	E	ES		D	
511	G	E	EN		D	D
512	S	C				
513	N	C				
514	M	C				
515	V	E	ES			
516	I	E	ES			
517	L	C		CE		
518	P	C				
519	T	C				
520	M	C				

521	D	C			
522	L	C			
523	R	C		CE	
524	Y	E	ES		
525	I	E	ES		
526	T	E	ES		D
527	A	E	ES		
528	T	E	EN		D
529	Y	E	ES		
530	D	C		CE	
531	V	C		CE	
532	S	C			
533	R	C			
534	R	C		D	
535	E	C			
536	H	C			
537	A	C			
538	I	E	ES		
539	V	E	ES		
540	Y	E	ES		
541	Y	E	ES		
542	I	E	ES		
543	Y	E	ES		
544	D	C			
545	T	C			
546	G	C			
547	L	C			
548	S	E	ES		
549	S	E	EN		
550	S	E	EN		
551	Y	E	ES		
552	Y	E	ES		
553	Y	E	ES		
554	P	C		CE	
555	V	C			
556	R	C			
557	L	C		CE	
558	N	C			
559	F	C			
560	K	C			
561	G	E	ES		
562	N	E	EN		
563	P	E	ES		
564	L	C			
565	S	E	EN		
566	L	E	ES		
567	R	E	EN		
568	I	E	ES		
569	E	E	ES		

570	C	E	ES	
571	F	E	ES	
572	P	E	ES	
573	W	C		
574	R	C		
575	H	C		
576	K	C		
577	V	E	ES	
578	W	E	ES	
579	C	E	ES	
580	Y	E	ES	
581	H	E	EN	
582	D	E	EN	
583	C	E	ES	
584	L	E	ES	
585	I	E	ES	
586	Y	E	ES	
587	N	E	ES	
588	T	C		
589	I	C		
590	T	C		
591	D	C		
592	E	E	EN	D
593	E	E	EN	D
594	V	E	EN	
595	H	E	EN	
596	T	E	EN	D
597	R	E	EN	D
598	G	E	EN	D
599	L	E	EN	
600	T	E	EN	
601	G	E	ES	D
602	I	E	EN	
603	E	E	ES	D
604	V	E	ES	
605	T	E	ES	
606	C	C		
