# Supplementary material for: Structural and functional characterization of peste des petits ruminants virus coded hemagglutinin protein using various in-silico approaches
Source: Front Microbiol. 2024 Jun 20;15:1427606. doi: 10.3389/fmicb.2024.1427606 (PMC11222573; doi:10.3389/fmicb.2024.1427606)
Supplement: Supplementary file 4 [file Data_Sheet_4.PDF]

A) Measles Virus (MeV)

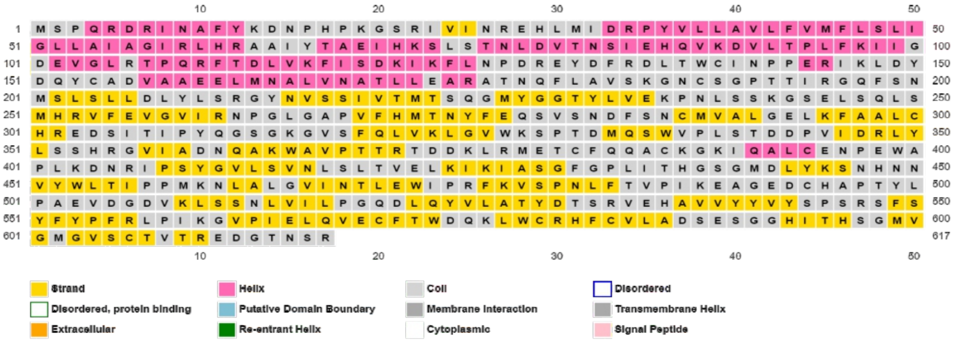

B) Rinderpest Virus (RPV)

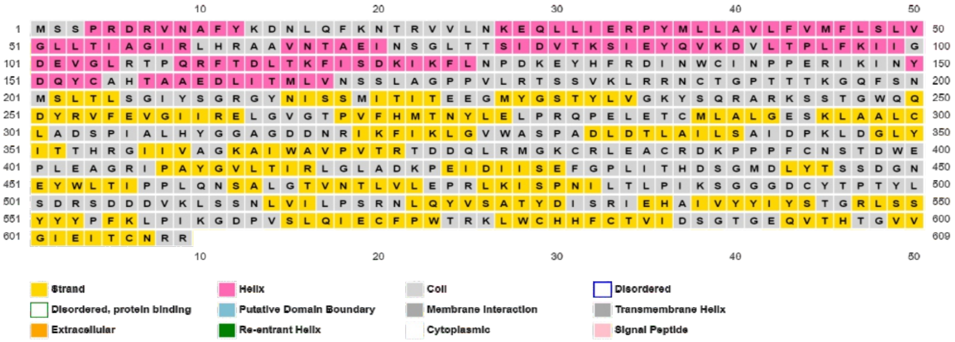

C) Canine Distemper Virus (CDV)

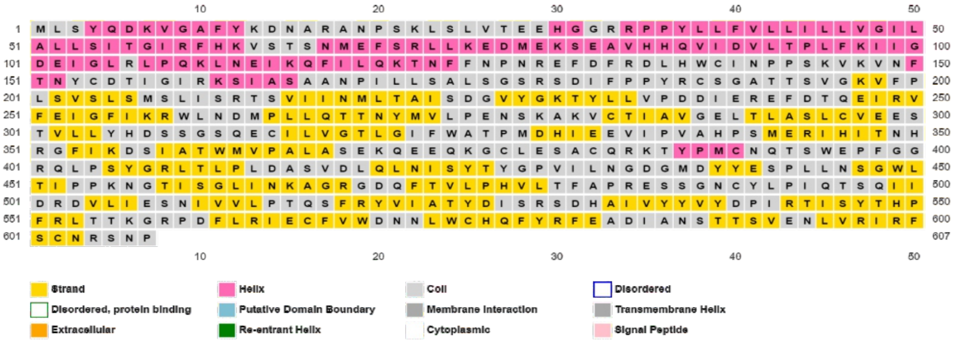

Supplementary file 4(A): Secondary structure of the different Hemagglutinin protein of genus *Morbillivirus* predicted using PSIPRED tool.

Supplementary file 4(B): % Secondary structure observed in Hemagglutinin protein of members in genus *Morbillivirus* predicted using SOPMA tool.

Virus	Secondary structure	%
Measles virus (AAK64474.1)	Alpha Helix	22.85%
	Beta sheets	27.71%
	Turns	4.70%
	Coils	44.73%
Canine Distemper virus (CAP17286.1)	Alpha Helix	18.29%
	Beta sheets	27.35%
	Turns	2.14%
	Coils	52.22%
Rinderpest virus (AAD25093.1)	Alpha Helix	19.05%
	Beta sheets	28.08%
	Turns	2.96%
	Coils	49.02%
