# Supplementary material for: Structural and functional characterization of peste des petits ruminants virus coded hemagglutinin protein using various in-silico approaches
Source: Front Microbiol. 2024 Jun 20;15:1427606. doi: 10.3389/fmicb.2024.1427606 (PMC11222573; doi:10.3389/fmicb.2024.1427606)
Supplement: Supplementary file 5 [file Data_Sheet_5.PDF]

A) Measles Virus (MeV)

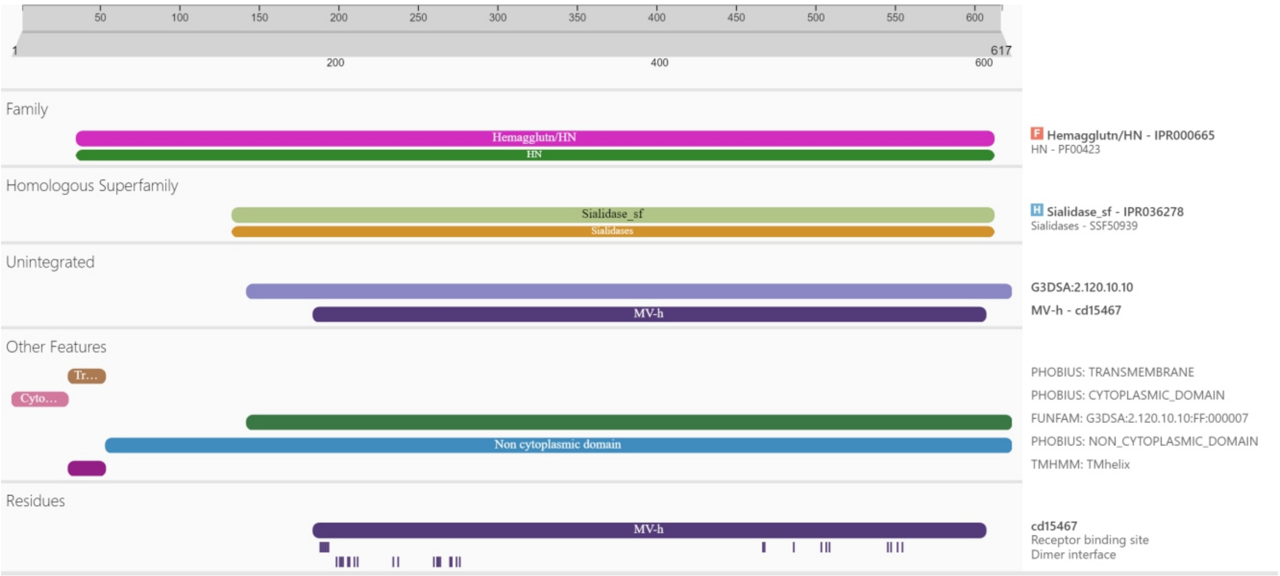

B) Rinderpest Virus (RPV)

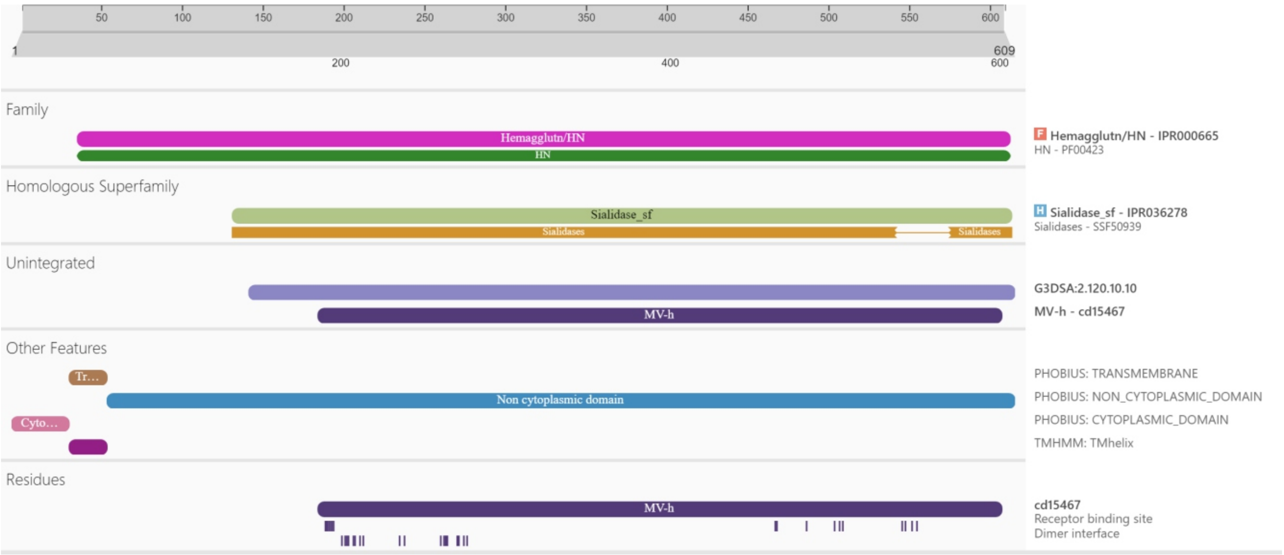

Supplementary file 5: The structural domains of Hemagglutinin protein were predicted using InterPro database. It shows Hemagglutinin protein comprises of short cytoplasmic domain followed by a transmembrane region and a large extracellular domain shown in pink, brown and blue color respectively.
