# Supplementary material for: Structural and functional characterization of peste des petits ruminants virus coded hemagglutinin protein using various in-silico approaches
Source: Front Microbiol. 2024 Jun 20;15:1427606. doi: 10.3389/fmicb.2024.1427606 (PMC11222573; doi:10.3389/fmicb.2024.1427606)
Supplement: Supplementary file 6 [file Data_Sheet_6.PDF]

HOME | SEARCH | GUIDE
NewSearch | Structure Home | 3D Macromolecular Structures | Conserved Domains | Pubchem

Conserved domains on [lcl|seqsig_MSAQR_35cdca228f929940e85809a3962dd08f]

View

Local query sequence

Protein Classification

sialidase/neuraminidase family protein(domain architecture ID 229403)
sialidase/neuraminidase family protein such as viral neuraminidase that catalyzes the removal of terminal sialic acid residues from viral and cellular

CATH: 2.120.10.10 | **CAZY:** GH33|GH34 | **EC:** 3.2.1.- | **Gene Ontology:** GO:0016997 | **PubMed:** 8994884|7934919 | **SCOP:** 3001607

Graphical summary

Zoom to residue level   show extra options »

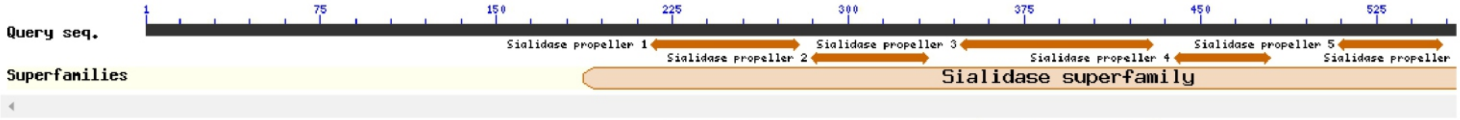

Search for similar domain architectures   Refine search

List of domain hits

Name	Accession	Description
Sialidase super family	cl21531	sialidases/neuraminidases; Sialidases or neuraminidases function to bind and hydrolyze ...

References:

- Marchler-Bauer A et al. (2017), *"CDD/SPARCLE: functional classification of proteins via subfamily domain architectures."*, **Nucleic Acids Res.45**(D)200-3.
- Marchler-Bauer A et al. (2015), *"CDD: NCBI's conserved domain database."*, **Nucleic Acids Res.43**(D)222-6.
- Marchler-Bauer A et al. (2011), *"CDD: a Conserved Domain Database for the functional annotation of proteins."*, **Nucleic Acids Res.39**(D)225-9.
- Marchler-Bauer A, Bryant SH (2004), *"CD-Search: protein domain annotations on the fly."*, **Nucleic Acids Res.32**(W)327-331.

Help | Disclaimer | Write to the Help Desk
NCBI | NLM | NIH

HHS Vulnerability Disclosure

Supplementary file 6(A): Different domains of PPRV-H protein identified using CDD

MSAQRERINAFYKGNPHNKNHRVILDRERLVIERPYILLGVLLVMFLSLIGLLAIAGIRLHRATVGTLEI
QSRLNTNIELTESIDHQTKDVLTPLFKIIGDEVGIRIPQKFSDLVKFISDKIKFLNPDREYDFRDLRWCM
NPPERVKINFDQFCEYKAAVKSIEHIFESPLNKSKKLQSLTLGPGTGCQGRTVTRAHFSELTLTLMDLDL
EMKHNVSSVFTVVEEGLFGRTYTVWRSDARDPSTDPGIGHFLRVFEIGLVRDLGLGPPVFHMTNYLTVNM
SDDYRRCLLAVGELKLTALCTSSETVTLSERGVPKRKPLVVVILNLAGPTLGGELYSVLPTSDLMVEKLY
LSSHRGIIKDDEANWVVPSTDVRDLQNKGECLVEACKTRPPSFCNGTGSGPWSEGRIPAYGVIRVSLNSA
SDPGVVITSVFGPLIPHLSGMDLYNNPFSRAVWLAVPPYEQSFLGMINTIGFPNRAEVMPHILTTEIRGP
RGRCHVPIELSRRVDDDIKIGSNMVILPTMDLRYITATYDVSRREHAIVYYIYDTGLSSSYYYPVRLNFK
GNPLSLRIECFPWRHKVWCYHDCLIYNTITDEEVHTRGLTGIEVTCNPV

Domain 1, Domain 2, Domain 3, Domain 4, Domain 5, Domain 6

Supplementary file 6(B): Sequences of different domains of PPRV-H protein identified using CDD
