# Supplementary material for: Structural and functional characterization of peste des petits ruminants virus coded hemagglutinin protein using various in-silico approaches
Source: Front Microbiol. 2024 Jun 20;15:1427606. doi: 10.3389/fmicb.2024.1427606 (PMC11222573; doi:10.3389/fmicb.2024.1427606)
Supplement: Supplementary file 7 [file Data_Sheet_7.PDF]

```
********************************************************************************
MEME - Motif discovery tool
********************************************************************************
MEME version 5.5.4 (Release date: Fri Jun 16 12:19:08 2023 -0700)

For further information on how to interpret these results please access https://meme-
suite.org/meme.
To get a copy of the MEME Suite software please access https://meme-suite.org.

********************************************************************************


********************************************************************************
REFERENCE
********************************************************************************
If you use this program in your research, please cite:

Timothy L. Bailey and Charles Elkan,
"Fitting a mixture model by expectation maximization to
discover motifs in biopolymers",
Proceedings of the Second International Conference on Intelligent Systems
for Molecular Biology, pp. 28-36, AAAI Press, Menlo Park, California, 1994.
********************************************************************************


********************************************************************************
TRAINING SET
********************************************************************************
PRIMARY SEQUENCES= sequences.fa
CONTROL SEQUENCES= --none--
ALPHABET= ACDEFGHIKLMNPQRSTVWY
Sequence name            Weight Length  Sequence name            Weight Length
-------------            ------ ------  -------------            ------ ------
Peste                    1.0000    609  Measles                  1.0000    617
Canine                   1.0000    607  Phocine                  1.0000    607
Rinderpest               1.0000    609
********************************************************************************

********************************************************************************
COMMAND LINE SUMMARY
********************************************************************************
This information can also be useful in the event you wish to report a
problem with the MEME software.

command: meme sequences.fa -protein -oc . -nostatus -time 14400 -mod zoops -nmotifs 6 -minw 5 -
maxw 15 -objfun classic -markov_order 0

model:  mod=         zoops    nmotifs=         6    evt=           inf
objective function:           em=       E-value of product of p-values
                              starts=   E-value of product of p-values
width:  minw=            5    maxw=           15
nsites: minsites=        2    maxsites=        5    wnsites=       0.8
theta:  spmap=         pam    spfuzz=        120
em:     prior=       megap    b=           15245    maxiter=        50
        distance=    1e-05
trim:   wg=             11    ws=              1    endgaps=       yes
data:   n=            3049    N=               5
sample: seed=            0    hsfrac=          0
        searchsize=   3049    norand=         no    csites=       1000
Dirichlet mixture priors file: prior30.plib
Letter frequencies in dataset:
A 0.0453 C 0.021 D 0.0541 E 0.0521 F 0.041 G 0.0613 H 0.0236 I 0.0751 K 0.0446
L 0.107 M 0.0203 N 0.0472 P 0.0561 Q 0.0279 R 0.0564 S 0.0771 T 0.0672 V 0.0708
W 0.0131 Y 0.0384
Background letter frequencies (from file dataset with add-one prior applied):
A 0.0453 C 0.021 D 0.0541 E 0.0522 F 0.041 G 0.0613 H 0.0236 I 0.0751 K 0.0446
L 0.107 M 0.0203 N 0.0472 P 0.0561 Q 0.0279 R 0.0564 S 0.0771 T 0.0672 V 0.0708
W 0.0131 Y 0.0384
Background model order: 0
********************************************************************************
```

```
********************************************************************************
MOTIF ECFPWDHKLWCHHFC MEME-1 width =  15  sites =   5  llr = 215  E-value = 2.1e-036
********************************************************************************
--------------------------------------------------------------------------------
         Motif ECFPWDHKLWCHHFC MEME-1 Description
--------------------------------------------------------------------------------
Simplified        A  :::::::::::::::
pos.-specific     C  :a:::::::a:::6
probability       D  :::::6::::::2:
matrix            E  a::::::::::::::
                  F  ::a:::::::::8:
                  G  :::::2::::::::
                  H  :::::22:::66::
                  I  :::::::::::::::
                  K  ::::::6:::::::
                  L  :::::::8::::::
                  M  :::::::::::::::
                  N  :::::22:::::::
                  P  :::4::::::::::
                  Q  :::::2:::::4::
                  R  ::::22::::2:::
                  S  :::::::::::::::
                  T  :::2:2::::::::
                  V  :::4::::2::::::
                  W  ::::a::::a:::::
                  Y  :::::::::::2::4

         bits    6.3     *    *
                 5.6  *  *    **
                 5.0  *  *    **
                 4.4 *** *    ** * *
Relative         3.8 *** *    ******
Entropy          3.1 *** *  * ******
(62.0 bits)      2.5 ***************
                 1.9 ***************
                 1.3 ***************
                 0.6 ***************
                 0.0 ---------------

Multilevel           ECFPWDGKLWCHHFC
consensus               V RHHV  RQDY
sequence                T TNN   Y
                          Q
                          R
--------------------------------------------------------------------------------

--------------------------------------------------------------------------------
         Motif ECFPWDHKLWCHHFC MEME-1 sites sorted by position p-value
--------------------------------------------------------------------------------
Sequence name            Start   P-value                 Site
-------------            ----- ---------            ---------------
Rinderpest                 569  1.26e-20 IKGDPVSLQI ECFPWTRKLWCHHFC TVIDSGTGEQ
Measles                    569  9.25e-20 IKGVPIELQV ECFTWDQKLWCRHFC VLADSESGGH
Phocine                    565  1.73e-19 TKGRPDILRI ECFVWDGHLWCHQFY RFQLDATNST
Canine                     565  2.15e-19 TKGRPDFLRI ECFVWDNNLWCHQFY RFEADIANST
Peste                      569  2.30e-17 FKGNPLSLRI ECFPWRHKVWCYHDC LIYNTITDEE
--------------------------------------------------------------------------------

--------------------------------------------------------------------------------
         Motif ECFPWDHKLWCHHFC MEME-1 block diagrams
--------------------------------------------------------------------------------
SEQUENCE NAME            POSITION P-VALUE  MOTIF DIAGRAM
-------------            ----------------  -------------
Rinderpest                        1.3e-20  568_[1]_26
Measles                           9.3e-20  568_[1]_34
Phocine                           1.7e-19  564_[1]_28
Canine                            2.2e-19  564_[1]_28
Peste                             2.3e-17  568_[1]_26
--------------------------------------------------------------------------------
```

```
--------------------------------------------------------------------------------
	Motif ECFPWDHKLWCHHFC MEME-1 in BLOCKS format
--------------------------------------------------------------------------------
BL   MOTIF ECFPWDHKLWCHHFC width=15 seqs=5
Rinderpest               (  569) ECFPWTRKLWCHHFC  1
Measles                  (  569) ECFTWDQKLWCRHFC  1
Phocine                  (  565) ECFVWDGHLWCHQFY  1
Canine                   (  565) ECFVWDNNLWCHQFY  1
Peste                    (  569) ECFPWRHKVWCYHDC  1
//

--------------------------------------------------------------------------------

--------------------------------------------------------------------------------
	Motif ECFPWDHKLWCHHFC MEME-1 position-specific scoring matrix
--------------------------------------------------------------------------------
log-odds matrix: alength= 20 w= 15 n= 2979 bayes= 10.1608 E= 2.1e-036
  -406   -565   -209    420   -592   -495   -440   -563   -483   -628   -512   -415   -625   -298
-546   -533   -538   -552   -554   -590
  -301    552   -524   -464   -471   -515   -459   -455   -493   -498   -362   -491   -552   -444
-488   -469   -410   -473   -532   -549
  -263   -252   -435   -407    440   -430   -342   -271   -397   -181   -167   -415   -415   -376
-451   -342   -415   -274   -165    -65
   -18   -181   -274   -205   -216   -235   -215   -120   -175   -189   -100   -239    277   -145
-242   -161     74    190   -278   -273
  -322   -340   -404   -385   -126   -394   -339   -431   -351   -276   -254   -380   -458   -295
-359   -414   -411   -362    609   -169
  -131   -328    358    -25   -346   -230   -148   -370    -98   -359   -240    -44   -328    -87
23   -183      0   -308   -334   -302
   -31   -259    -80    -18   -239     71    294   -285     36   -247   -118    139   -224    230
112    -91   -118   -216   -234   -160
  -123   -321   -151   -110   -357   -227    197   -353    365   -328   -206    117   -309    -23
-15   -177   -202   -303   -309   -284
  -207   -261   -465   -363   -125   -428   -324    -76   -326    278     35   -398   -395   -239
-340   -371   -288     56   -267   -291
  -322   -340   -404   -385   -126   -394   -339   -431   -351   -276   -254   -380   -458   -295
-359   -414   -411   -362    609   -169
  -301    552   -524   -464   -471   -515   -459   -455   -493   -498   -362   -491   -552   -444
-488   -469   -410   -473   -532   -549
  -227   -265   -225   -237   -161   -321    485   -392   -234   -299   -178    -62   -335     37
-6   -223   -256   -320   -180     78
  -208   -275   -210   -187   -206   -305    471   -393   -167   -305   -180    -70   -331    256
-132   -219   -252   -323   -207    -65
  -248   -246    -76   -365    433   -409   -324   -267   -367   -179   -164   -365   -404   -347
-431   -323   -395   -268   -161    -58
  -203    466   -429   -406    -21   -359   -158   -307   -347   -289   -220   -335   -433   -285
-370   -320   -335   -276    -97    309
--------------------------------------------------------------------------------

--------------------------------------------------------------------------------
	Motif ECFPWDHKLWCHHFC MEME-1 position-specific probability matrix
--------------------------------------------------------------------------------
letter-probability matrix: alength= 20 w= 15 nsites= 5 E= 2.1e-036
 0.000000  0.000000  0.000000  1.000000  0.000000  0.000000  0.000000  0.000000  0.000000
0.000000  0.000000  0.000000  0.000000  0.000000  0.000000  0.000000  0.000000  0.000000
0.000000  0.000000
 0.000000  1.000000  0.000000  0.000000  0.000000  0.000000  0.000000  0.000000  0.000000
0.000000  0.000000  0.000000  0.000000  0.000000  0.000000  0.000000  0.000000  0.000000
0.000000  0.000000
 0.000000  0.000000  0.000000  0.000000  1.000000  0.000000  0.000000  0.000000  0.000000
0.000000  0.000000  0.000000  0.000000  0.000000  0.000000  0.000000  0.000000  0.000000
0.000000  0.000000
 0.000000  0.000000  0.000000  0.000000  0.000000  0.000000  0.000000  0.000000  0.000000
0.000000  0.000000  0.000000  0.400000  0.000000  0.000000  0.000000  0.200000  0.400000
0.000000  0.000000
 0.000000  0.000000  0.000000  0.000000  0.000000  0.000000  0.000000  0.000000  0.000000
0.000000  0.000000  0.000000  0.000000  0.000000  0.000000  0.000000  0.000000  0.000000
1.000000  0.000000
 0.000000  0.000000  0.600000  0.000000  0.000000  0.000000  0.000000  0.000000  0.000000
0.000000  0.000000  0.000000  0.000000  0.000000  0.200000  0.000000  0.200000  0.000000
0.000000  0.000000
```

```
 0.000000  0.000000  0.000000  0.000000  0.000000  0.200000  0.200000  0.000000  0.000000
0.000000  0.000000  0.200000  0.000000  0.200000  0.200000  0.000000  0.000000  0.000000
0.000000  0.000000
 0.000000  0.000000  0.000000  0.000000  0.000000  0.000000  0.200000  0.000000  0.600000
0.000000  0.000000  0.200000  0.000000  0.000000  0.000000  0.000000  0.000000  0.000000
0.000000  0.000000
 0.000000  0.000000  0.000000  0.000000  0.000000  0.000000  0.000000  0.000000  0.000000
0.800000  0.000000  0.000000  0.000000  0.000000  0.000000  0.000000  0.000000  0.200000
0.000000  0.000000
 0.000000  0.000000  0.000000  0.000000  0.000000  0.000000  0.000000  0.000000  0.000000
0.000000  0.000000  0.000000  0.000000  0.000000  0.000000  0.000000  0.000000  0.000000
1.000000  0.000000
 0.000000  1.000000  0.000000  0.000000  0.000000  0.000000  0.000000  0.000000  0.000000
0.000000  0.000000  0.000000  0.000000  0.000000  0.000000  0.000000  0.000000  0.000000
0.000000  0.000000
 0.000000  0.000000  0.000000  0.000000  0.000000  0.000000  0.600000  0.000000  0.000000
0.000000  0.000000  0.000000  0.000000  0.000000  0.200000  0.000000  0.000000  0.000000
0.000000  0.200000
 0.000000  0.000000  0.000000  0.000000  0.000000  0.000000  0.600000  0.000000  0.000000
0.000000  0.000000  0.000000  0.000000  0.400000  0.000000  0.000000  0.000000  0.000000
0.000000  0.000000
 0.000000  0.000000  0.200000  0.000000  0.800000  0.000000  0.000000  0.000000  0.000000
0.000000  0.000000  0.000000  0.000000  0.000000  0.000000  0.000000  0.000000  0.000000
0.000000  0.000000
 0.000000  0.600000  0.000000  0.000000  0.000000  0.000000  0.000000  0.000000  0.000000
0.000000  0.000000  0.000000  0.000000  0.000000  0.000000  0.000000  0.000000  0.000000
0.000000  0.400000
--------------------------------------------------------------------------------

--------------------------------------------------------------------------------
         Motif ECFPWDHKLWCHHFC MEME-1 regular expression
--------------------------------------------------------------------------------
ECF[PVT]W[DRT][GHNQR][KHN][LV]WC[HRY][HQ][FD][CY]
--------------------------------------------------------------------------------




Time  0.45 secs.

********************************************************************************


********************************************************************************
MOTIF REYDFRDLHWCINPP MEME-2 width =  15  sites =   5  llr = 210  E-value = 3.3e-033
********************************************************************************
--------------------------------------------------------------------------------
         Motif REYDFRDLHWCINPP MEME-2 Description
--------------------------------------------------------------------------------
Simplified        A  :::::::::::::::
pos.-specific     C  ::::::::::a::::
probability       D  :::8::8::::::::
matrix            E  :a::::2::::::::
                  F  ::4:a::::::::::
                  G  :::::::::::::::
                  H  :::2::::4::::::
                  I  :::::::2:::8:::
                  K  2::::::::::::::
                  L  :::::::8:::::::
                  M  :::::::::::2:::
                  N  ::::::::2:::a::
                  P  :::::::::::::aa
                  Q  :::::::::::::::
                  R  8::::a::2::::::
                  S  :::::::::::::::
                  T  ::::::::2::::::
                  V  :::::::::::::::
                  W  :::::::::a:::::
                  Y  ::6::::::::::::

         bits    6.3          *
```

```
                 5.6          **
                 5.0          **
                 4.4  *  **   ** ***
Relative         3.8 *******  ** ***
Entropy          3.1 *******  ******
(60.7 bits)      2.5 ***************
                 1.9 ***************
                 1.3 ***************
                 0.6 ***************
                 0.0 ---------------

Multilevel           REYDFRDLHWCINPP
consensus            K FH  EIN  M
sequence                     R
                             T

--------------------------------------------------------------------------------

--------------------------------------------------------------------------------
         Motif REYDFRDLHWCINPP MEME-2 sites sorted by position p-value
--------------------------------------------------------------------------------
Sequence name            Start   P-value                Site
-------------            ----- ---------            ---------------
Canine                     129  1.23e-20 LQKTNFFNPN REFDFRDLHWCINPP SKVKVNFTNY
Measles                    129  1.23e-19 SDKIKFLNPD REYDFRDLTWCINPP ERIKLDYDQY
Phocine                    129  2.16e-19 VQKTNFFNPN REFDFRELHWCINPP SKVKVNFTQY
Peste                      129  3.07e-19 SDKIKFLNPD REYDFRDLRWCMNPP ERVKINFDQF
Rinderpest                 129  1.06e-16 SDKIKFLNPD KEYHFRDINWCINPP ERIKINYDQY
--------------------------------------------------------------------------------

--------------------------------------------------------------------------------
         Motif REYDFRDLHWCINPP MEME-2 block diagrams
--------------------------------------------------------------------------------
SEQUENCE NAME            POSITION P-VALUE  MOTIF DIAGRAM
-------------            ----------------  -------------
Canine                            1.2e-20  128_[2]_464
Measles                           1.2e-19  128_[2]_474
Phocine                           2.2e-19  128_[2]_464
Peste                             3.1e-19  128_[2]_466
Rinderpest                        1.1e-16  128_[2]_466
--------------------------------------------------------------------------------

--------------------------------------------------------------------------------
         Motif REYDFRDLHWCINPP MEME-2 in BLOCKS format
--------------------------------------------------------------------------------
BL   MOTIF REYDFRDLHWCINPP width=15 seqs=5
Canine                   (  129) REFDFRDLHWCINPP  1
Measles                  (  129) REYDFRDLTWCINPP  1
Phocine                  (  129) REFDFRELHWCINPP  1
Peste                    (  129) REYDFRDLRWCMNPP  1
Rinderpest               (  129) KEYHFRDINWCINPP  1
//

--------------------------------------------------------------------------------

--------------------------------------------------------------------------------
         Motif REYDFRDLHWCINPP MEME-2 position-specific scoring matrix
--------------------------------------------------------------------------------
log-odds matrix: alength= 20 w= 15 n= 2979 bayes= 9.46819 E= 3.3e-033
  -250   -287   -407   -343   -443   -365   -169   -419     38   -393   -307   -287   -362   -127
392   -343   -354   -436   -277   -395
  -406   -565   -209    420   -592   -495   -440   -563   -483   -628   -512   -415   -625   -298
-546   -533   -538   -552   -554   -590
  -186   -247   -344   -300    292   -354    -20   -288   -272   -237   -165   -277   -366   -219
-295   -272   -313   -259    -15    377
  -233   -370    395    -60   -396   -318      8   -433   -299   -445   -336    -68   -444   -246
-362   -297   -360   -383   -373   -366
  -263   -252   -435   -407    440   -430   -342   -271   -397   -181   -167   -415   -415   -376
-451   -342   -415   -274   -165    -65
  -270   -290   -427   -401   -449   -379   -187   -435    -79   -414   -335   -315   -371   -166
399   -367   -383   -467   -283   -414
```

```
  -229   -375    395     13   -403   -332   -237   -430   -298   -442   -332    -76   -444   -231
-367   -310   -366   -379   -376   -376
  -222   -272   -475   -372   -117   -441   -330     57   -336    279     41   -409   -401   -243
-347   -386   -300   -107   -263   -294
  -106   -263   -131    -99   -219   -222    428   -330    -39   -276   -150    107   -275     42
68   -141     28   -261   -218    -94
  -322   -340   -404   -385   -126   -394   -339   -431   -351   -276   -254   -380   -458   -295
-359   -414   -411   -362    609   -169
  -301    552   -524   -464   -471   -515   -459   -455   -493   -498   -362   -491   -552   -444
-488   -469   -410   -473   -532   -549
  -204   -281   -403   -369   -203   -416   -376    315   -314    -51    151   -357   -446   -301
-391   -349   -267     75   -317   -300
  -279   -329   -202   -351   -364   -315    -90   -362   -260   -430   -303    421   -392   -201
-342   -187   -270   -377   -315   -355
  -107   -333   -313   -272   -371   -307   -284   -398   -239   -361   -300   -325    396   -204
-308   -238   -277   -328   -402   -419
  -107   -333   -313   -272   -371   -307   -284   -398   -239   -361   -300   -325    396   -204
-308   -238   -277   -328   -402   -419
--------------------------------------------------------------------------------

--------------------------------------------------------------------------------
         Motif REYDFRDLHWCINPP MEME-2 position-specific probability matrix
--------------------------------------------------------------------------------
letter-probability matrix: alength= 20 w= 15 nsites= 5 E= 3.3e-033
 0.000000  0.000000  0.000000  0.000000  0.000000  0.000000  0.000000  0.000000  0.200000
0.000000  0.000000  0.000000  0.000000  0.000000  0.800000  0.000000  0.000000  0.000000
0.000000  0.000000
 0.000000  0.000000  0.000000  1.000000  0.000000  0.000000  0.000000  0.000000  0.000000
0.000000  0.000000  0.000000  0.000000  0.000000  0.000000  0.000000  0.000000  0.000000
0.000000  0.000000
 0.000000  0.000000  0.000000  0.000000  0.400000  0.000000  0.000000  0.000000  0.000000
0.000000  0.000000  0.000000  0.000000  0.000000  0.000000  0.000000  0.000000  0.000000
0.000000  0.600000
 0.000000  0.000000  0.800000  0.000000  0.000000  0.000000  0.200000  0.000000  0.000000
0.000000  0.000000  0.000000  0.000000  0.000000  0.000000  0.000000  0.000000  0.000000
0.000000  0.000000
 0.000000  0.000000  0.000000  0.000000  1.000000  0.000000  0.000000  0.000000  0.000000
0.000000  0.000000  0.000000  0.000000  0.000000  0.000000  0.000000  0.000000  0.000000
0.000000  0.000000
 0.000000  0.000000  0.000000  0.000000  0.000000  0.000000  0.000000  0.000000  0.000000
0.000000  0.000000  0.000000  0.000000  0.000000  1.000000  0.000000  0.000000  0.000000
0.000000  0.000000
 0.000000  0.000000  0.800000  0.200000  0.000000  0.000000  0.000000  0.000000  0.000000
0.000000  0.000000  0.000000  0.000000  0.000000  0.000000  0.000000  0.000000  0.000000
0.000000  0.000000
 0.000000  0.000000  0.000000  0.000000  0.000000  0.000000  0.000000  0.200000  0.000000
0.800000  0.000000  0.000000  0.000000  0.000000  0.000000  0.000000  0.000000  0.000000
0.000000  0.000000
 0.000000  0.000000  0.000000  0.000000  0.000000  0.000000  0.400000  0.000000  0.000000
0.000000  0.000000  0.200000  0.000000  0.000000  0.200000  0.000000  0.200000  0.000000
0.000000  0.000000
 0.000000  0.000000  0.000000  0.000000  0.000000  0.000000  0.000000  0.000000  0.000000
0.000000  0.000000  0.000000  0.000000  0.000000  0.000000  0.000000  0.000000  0.000000
1.000000  0.000000
 0.000000  1.000000  0.000000  0.000000  0.000000  0.000000  0.000000  0.000000  0.000000
0.000000  0.000000  0.000000  0.000000  0.000000  0.000000  0.000000  0.000000  0.000000
0.000000  0.000000
 0.000000  0.000000  0.000000  0.000000  0.000000  0.000000  0.000000  0.800000  0.000000
0.000000  0.200000  0.000000  0.000000  0.000000  0.000000  0.000000  0.000000  0.000000
0.000000  0.000000
 0.000000  0.000000  0.000000  0.000000  0.000000  0.000000  0.000000  0.000000  0.000000
0.000000  0.000000  1.000000  0.000000  0.000000  0.000000  0.000000  0.000000  0.000000
0.000000  0.000000
 0.000000  0.000000  0.000000  0.000000  0.000000  0.000000  0.000000  0.000000  0.000000
0.000000  0.000000  0.000000  1.000000  0.000000  0.000000  0.000000  0.000000  0.000000
0.000000  0.000000
 0.000000  0.000000  0.000000  0.000000  0.000000  0.000000  0.000000  0.000000  0.000000
0.000000  0.000000  0.000000  1.000000  0.000000  0.000000  0.000000  0.000000  0.000000
0.000000  0.000000
--------------------------------------------------------------------------------
```

```
--------------------------------------------------------------------------------
         Motif REYDFRDLHWCINPP MEME-2 regular expression
--------------------------------------------------------------------------------
[RK]E[YF][DH]FR[DE][LI][HNRT]WC[IM]NPP
--------------------------------------------------------------------------------




Time  0.86 secs.

********************************************************************************


********************************************************************************
MOTIF HQVKDVLTPLFKIIG MEME-3 width =  15  sites =   5  llr = 206  E-value = 9.2e-031
********************************************************************************
--------------------------------------------------------------------------------
         Motif HQVKDVLTPLFKIIG MEME-3 Description
--------------------------------------------------------------------------------
Simplified        A  :::::::::::::::
pos.-specific     C  :::::::::::::::
probability       D  ::::a::::::::::
matrix            E  :::::::::::::::
                  F  ::::::::::a::::
                  G  ::::::::::::::a
                  H  8::::::::::::::
                  I  :::2::::::::aa:
                  K  :::8:::::::a:::
                  L  ::::::a::a:::::
                  M  :::::::::::::::
                  N  :::::::::::::::
                  P  ::::::::a::::::
                  Q  :a:::::::::::::
                  R  :::::::::::::::
                  S  :::::::::::::::
                  T  ::2::::a:::::::
                  V  ::8::a:::::::::
                  W  :::::::::::::::
                  Y  2::::::::::::::

         bits    6.3
                 5.6
                 5.0  *
                 4.4 **   *    * **
Relative         3.8 ** *** ** *****
Entropy          3.1 ***************
(59.5 bits)      2.5 ***************
                 1.9 ***************
                 1.3 ***************
                 0.6 ***************
                 0.0 ---------------

Multilevel           HQVKDVLTPLFKIIG
consensus            Y TI
sequence


--------------------------------------------------------------------------------

--------------------------------------------------------------------------------
         Motif HQVKDVLTPLFKIIG MEME-3 sites sorted by position p-value
--------------------------------------------------------------------------------
Sequence name            Start   P-value                Site
-------------            ----- ---------            ---------------
Phocine                     86  2.17e-19 EDMEKTEAVH HQVKDVLTPLFKIIG DEVGLRLPQK
Measles                     86  2.17e-19 TNLDVTNSIE HQVKDVLTPLFKIIG DEVGLRTPQR
Peste                       86  9.16e-19 TNIELTESID HQTKDVLTPLFKIIG DEVGIRIPQK
Rinderpest                  86  1.99e-18 TSIDVTKSIE YQVKDVLTPLFKIIG DEVGLRTPQR
Canine                      86  1.17e-17 EDMEKSEAVH HQVIDVLTPLFKIIG DEIGLRLPQK
--------------------------------------------------------------------------------
```

```
--------------------------------------------------------------------------------
	Motif HQVKDVLTPLFKIIG MEME-3 block diagrams
--------------------------------------------------------------------------------
SEQUENCE NAME            POSITION P-VALUE  MOTIF DIAGRAM
-------------            ----------------  -------------
Phocine                           2.2e-19  85_[3]_507
Measles                           2.2e-19  85_[3]_517
Peste                             9.2e-19  85_[3]_509
Rinderpest                          2e-18  85_[3]_509
Canine                            1.2e-17  85_[3]_507
--------------------------------------------------------------------------------

--------------------------------------------------------------------------------
	Motif HQVKDVLTPLFKIIG MEME-3 in BLOCKS format
--------------------------------------------------------------------------------
BL   MOTIF HQVKDVLTPLFKIIG width=15 seqs=5
Phocine                  (   86) HQVKDVLTPLFKIIG  1 
Measles                  (   86) HQVKDVLTPLFKIIG  1 
Peste                    (   86) HQTKDVLTPLFKIIG  1 
Rinderpest               (   86) YQVKDVLTPLFKIIG  1 
Canine                   (   86) HQVIDVLTPLFKIIG  1 
//

--------------------------------------------------------------------------------

--------------------------------------------------------------------------------
	Motif HQVKDVLTPLFKIIG MEME-3 position-specific scoring matrix
--------------------------------------------------------------------------------
log-odds matrix: alength= 20 w= 15 n= 2979 bayes= 9.21626 E= 9.2e-031 
  -245   -271   -238   -258   -127   -333    492   -397   -289   -302   -186    -73   -347     24
-162   -236   -270   -329   -156    105 
  -191   -322   -345   -100   -399   -389    -23   -400   -220   -303   -143   -243   -339    491
-216   -302   -317   -363   -293   -395 
   -24   -165   -348   -291   -250   -337   -274    -18   -280   -198   -128   -334   -337   -264
-303   -291    -10    334   -343   -371 
  -256   -359   -431   -343   -477   -415   -314   -182    429   -437   -314   -319   -438   -246
-43   -398   -362   -395   -379   -441 
  -248   -378    401    -71   -408   -341   -248   -441   -331   -454   -347    -85   -460   -272
-383   -324   -383   -392   -380   -383 
   -56   -190   -377   -325   -268   -366   -308    -29   -315   -207   -146   -369   -367   -300
-338   -331   -197    349   -370   -390 
  -245   -297   -472   -380   -141   -435   -335   -124   -341    298     17   -417   -406   -253
-352   -396   -326   -156   -279   -304 
  -145   -241   -346   -354   -358   -331   -311   -305   -271   -386   -208   -209   -382   -232
-325    -66    364   -243   -357   -396 
  -107   -333   -313   -272   -371   -307   -284   -398   -239   -361   -300   -325    396   -204
-308   -238   -277   -328   -402   -419 
  -245   -297   -472   -380   -141   -435   -335   -124   -341    298     17   -417   -406   -253
-352   -396   -326   -156   -279   -304 
  -263   -252   -435   -407    440   -430   -342   -271   -397   -181   -167   -415   -415   -376
-451   -342   -415   -274   -165    -65 
  -275   -370   -454   -379   -513   -430   -341   -429    434   -481   -347   -338   -451   -289
-51   -425   -387   -457   -391   -469 
  -215   -290   -415   -386   -236   -421   -397    331   -332    -81    -46   -373   -459   -325
-410   -366   -282     63   -346   -319 
  -215   -290   -415   -386   -236   -421   -397    331   -332    -81    -46   -373   -459   -325
-410   -366   -282     63   -346   -319 
  -133   -345   -267   -310   -438    387   -315   -484   -280   -504   -350   -236   -405   -304
-324   -260   -370   -405   -358   -415 
--------------------------------------------------------------------------------

--------------------------------------------------------------------------------
	Motif HQVKDVLTPLFKIIG MEME-3 position-specific probability matrix
--------------------------------------------------------------------------------
letter-probability matrix: alength= 20 w= 15 nsites= 5 E= 9.2e-031 
 0.000000  0.000000  0.000000  0.000000  0.000000  0.000000  0.800000  0.000000  0.000000
0.000000  0.000000  0.000000  0.000000  0.000000  0.000000  0.000000  0.000000  0.000000
0.000000  0.200000 
```

```
 0.000000  0.000000  0.000000  0.000000  0.000000  0.000000  0.000000  0.000000  0.000000
0.000000  0.000000  0.000000  0.000000  1.000000  0.000000  0.000000  0.000000  0.000000
0.000000  0.000000
 0.000000  0.000000  0.000000  0.000000  0.000000  0.000000  0.000000  0.000000  0.000000
0.000000  0.000000  0.000000  0.000000  0.000000  0.000000  0.000000  0.200000  0.800000
0.000000  0.000000
 0.000000  0.000000  0.000000  0.000000  0.000000  0.000000  0.000000  0.200000  0.800000
0.000000  0.000000  0.000000  0.000000  0.000000  0.000000  0.000000  0.000000  0.000000
0.000000  0.000000
 0.000000  0.000000  1.000000  0.000000  0.000000  0.000000  0.000000  0.000000  0.000000
0.000000  0.000000  0.000000  0.000000  0.000000  0.000000  0.000000  0.000000  0.000000
0.000000  0.000000
 0.000000  0.000000  0.000000  0.000000  0.000000  0.000000  0.000000  0.000000  0.000000
0.000000  0.000000  0.000000  0.000000  0.000000  0.000000  0.000000  0.000000  1.000000
0.000000  0.000000
 0.000000  0.000000  0.000000  0.000000  0.000000  0.000000  0.000000  0.000000  0.000000
1.000000  0.000000  0.000000  0.000000  0.000000  0.000000  0.000000  0.000000  0.000000
0.000000  0.000000
 0.000000  0.000000  0.000000  0.000000  0.000000  0.000000  0.000000  0.000000  0.000000
0.000000  0.000000  0.000000  0.000000  0.000000  0.000000  0.000000  1.000000  0.000000
0.000000  0.000000
 0.000000  0.000000  0.000000  0.000000  0.000000  0.000000  0.000000  0.000000  0.000000
0.000000  0.000000  0.000000  1.000000  0.000000  0.000000  0.000000  0.000000  0.000000
0.000000  0.000000
 0.000000  0.000000  0.000000  0.000000  0.000000  0.000000  0.000000  0.000000  0.000000
1.000000  0.000000  0.000000  0.000000  0.000000  0.000000  0.000000  0.000000  0.000000
0.000000  0.000000
 0.000000  0.000000  0.000000  0.000000  1.000000  0.000000  0.000000  0.000000  0.000000
0.000000  0.000000  0.000000  0.000000  0.000000  0.000000  0.000000  0.000000  0.000000
0.000000  0.000000
 0.000000  0.000000  0.000000  0.000000  0.000000  0.000000  0.000000  0.000000  1.000000
0.000000  0.000000  0.000000  0.000000  0.000000  0.000000  0.000000  0.000000  0.000000
0.000000  0.000000
 0.000000  0.000000  0.000000  0.000000  0.000000  0.000000  0.000000  1.000000  0.000000
0.000000  0.000000  0.000000  0.000000  0.000000  0.000000  0.000000  0.000000  0.000000
0.000000  0.000000
 0.000000  0.000000  0.000000  0.000000  0.000000  0.000000  0.000000  1.000000  0.000000
0.000000  0.000000  0.000000  0.000000  0.000000  0.000000  0.000000  0.000000  0.000000
0.000000  0.000000
 0.000000  0.000000  0.000000  0.000000  0.000000  1.000000  0.000000  0.000000  0.000000
0.000000  0.000000  0.000000  0.000000  0.000000  0.000000  0.000000  0.000000  0.000000
0.000000  0.000000
--------------------------------------------------------------------------------

--------------------------------------------------------------------------------
         Motif HQVKDVLTPLFKIIG MEME-3 regular expression
--------------------------------------------------------------------------------
[HY]Q[VT][KI]DVLTPLFKIIG
--------------------------------------------------------------------------------




Time  1.26 secs.

********************************************************************************


********************************************************************************
MOTIF ATYDISRSEHAIVYY MEME-4 width =  15  sites =   5  llr = 203  E-value = 1.4e-030
********************************************************************************
--------------------------------------------------------------------------------
         Motif ATYDISRSEHAIVYY MEME-4 Description
--------------------------------------------------------------------------------
Simplified        A  a::::::::a::::
pos.-specific     C  :::::::::::::::
probability       D  :::a::::4::::::
matrix            E  ::::::::6::::::
                  F  :::::::::::::::
                  G  :::::::::::::::
                  H  :::::::::a:::::
```

```
                I  ::::4::2:::8:::
                K  :::::::::::::::
                L  :::::::::::::::
                M  :::::::::::::::
                N  :::::::::::::::
                P  :::::::::::::::
                Q  :::::::::::::::
                R  :::::a2:::::::
                S  :::::a:4:::::::
                T  :a::2::::::::::
                V  ::::4::2:::2a::
                W  :::::::::::::::
                Y  ::a::::::::::aa

        bits    6.3
                5.6          *
                5.0   *      *   **
                4.4 * **  *  **  **
Relative        3.8 **** **  ** ***
Entropy         3.1 **** ** *******
(58.7 bits)     2.5 ******* *******
                1.9 ***************
                1.3 ***************
                0.6 ***************
                0.0 ---------------

Multilevel           ATYDISRSEHAIVYY
consensus                V  ID  V
sequence                 T  R
                            V

--------------------------------------------------------------------------------

--------------------------------------------------------------------------------
         Motif ATYDISRSEHAIVYY MEME-4 sites sorted by position p-value
--------------------------------------------------------------------------------
Sequence name            Start   P-value                Site
-------------            ----- ---------            ---------------
Rinderpest                 527  3.28e-19 LPSRNLQYVS ATYDISRIEHAIVYY IYSTGRLSSY
Peste                      527  4.90e-19 LPTMDLRYIT ATYDVSRREHAIVYY IYDTGLSSSY
Canine                     523  5.63e-19 LPTQSFRYVI ATYDISRSDHAIVYY VYDPIRTISY
Phocine                    523  6.90e-19 LPTQSFEYVV ATYDVSRSDHAIVYY VYDPARTVSY
Measles                    527  8.38e-18 LPGQDLQYVL ATYDTSRVEHAVVYY VYSPSRSFSY
--------------------------------------------------------------------------------

--------------------------------------------------------------------------------
         Motif ATYDISRSEHAIVYY MEME-4 block diagrams
--------------------------------------------------------------------------------
SEQUENCE NAME            POSITION P-VALUE  MOTIF DIAGRAM
-------------            ----------------  -------------
Rinderpest                        3.3e-19  526_[4]_68
Peste                             4.9e-19  526_[4]_68
Canine                            5.6e-19  522_[4]_70
Phocine                           6.9e-19  522_[4]_70
Measles                           8.4e-18  526_[4]_76
--------------------------------------------------------------------------------

--------------------------------------------------------------------------------
         Motif ATYDISRSEHAIVYY MEME-4 in BLOCKS format
--------------------------------------------------------------------------------
BL   MOTIF ATYDISRSEHAIVYY width=15 seqs=5
Rinderpest               (  527) ATYDISRIEHAIVYY  1
Peste                    (  527) ATYDVSRREHAIVYY  1
Canine                   (  523) ATYDISRSDHAIVYY  1
Phocine                  (  523) ATYDVSRSDHAIVYY  1
Measles                  (  527) ATYDTSRVEHAVVYY  1
//

--------------------------------------------------------------------------------

--------------------------------------------------------------------------------
```

```
         Motif ATYDISRSEHAIVYY MEME-4 position-specific scoring matrix
--------------------------------------------------------------------------------
log-odds matrix: alength= 20 w= 15 n= 2979 bayes= 9.46819 E= 1.4e-030
   418   -119   -363   -306   -318   -158   -328   -324   -298   -325   -204   -321   -385   -266
-339   -100   -211   -162   -330   -378
  -145   -241   -346   -354   -358   -331   -311   -305   -271   -386   -208   -209   -382   -232
-325    -66    364   -243   -357   -396
  -294   -338   -425   -407      0   -401   -130   -384   -364   -329   -268   -358   -440   -305
-382   -375   -407   -357    -99    451
  -248   -378    401    -71   -408   -341   -248   -441   -331   -454   -347    -85   -460   -272
-383   -324   -383   -392   -380   -383
  -115   -208   -398   -345   -219   -382   -334    246   -309   -106    -55   -350   -404   -291
-363   -323     30    232   -326   -311
   -87   -213   -321   -347   -360   -266   -312   -420   -275   -417   -284   -199   -346   -269
-320    341      3   -365   -364   -363
  -270   -290   -427   -401   -449   -379   -187   -435    -79   -414   -335   -315   -371   -166
399   -367   -383   -467   -283   -414
     3   -130   -209   -120   -117   -208   -124     99    -64    -95      6   -148   -250    -52
92    158    -60    124   -174   -156
  -139   -524    214    358   -505   -301   -248   -446   -134   -428   -308   -193   -332    -32
-301   -266   -291   -354   -491   -437
  -292   -325   -288   -311   -248   -357    517   -466   -328   -371   -257   -137   -389    -43
-222   -294   -328   -394   -253   -113
   418   -119   -363   -306   -318   -158   -328   -324   -298   -325   -204   -321   -385   -266
-339   -100   -211   -162   -330   -378
  -204   -279   -402   -371   -236   -421   -392    317   -318    -68    -34   -358   -453   -315
-400   -354   -266    122   -344   -316
   -56   -190   -377   -325   -268   -366   -308    -29   -315   -207   -146   -369   -367   -300
-338   -331   -197    349   -370   -390
  -294   -338   -425   -407      0   -401   -130   -384   -364   -329   -268   -358   -440   -305
-382   -375   -407   -357    -99    451
  -294   -338   -425   -407      0   -401   -130   -384   -364   -329   -268   -358   -440   -305
-382   -375   -407   -357    -99    451
--------------------------------------------------------------------------------

--------------------------------------------------------------------------------
         Motif ATYDISRSEHAIVYY MEME-4 position-specific probability matrix
--------------------------------------------------------------------------------
letter-probability matrix: alength= 20 w= 15 nsites= 5 E= 1.4e-030
 1.000000  0.000000  0.000000  0.000000  0.000000  0.000000  0.000000  0.000000  0.000000
0.000000  0.000000  0.000000  0.000000  0.000000  0.000000  0.000000  0.000000  0.000000
0.000000  0.000000
 0.000000  0.000000  0.000000  0.000000  0.000000  0.000000  0.000000  0.000000  0.000000
0.000000  0.000000  0.000000  0.000000  0.000000  0.000000  0.000000  1.000000  0.000000
0.000000  0.000000
 0.000000  0.000000  0.000000  0.000000  0.000000  0.000000  0.000000  0.000000  0.000000
0.000000  0.000000  0.000000  0.000000  0.000000  0.000000  0.000000  0.000000  0.000000
0.000000  1.000000
 0.000000  0.000000  1.000000  0.000000  0.000000  0.000000  0.000000  0.000000  0.000000
0.000000  0.000000  0.000000  0.000000  0.000000  0.000000  0.000000  0.000000  0.000000
0.000000  0.000000
 0.000000  0.000000  0.000000  0.000000  0.000000  0.000000  0.000000  0.400000  0.000000
0.000000  0.000000  0.000000  0.000000  0.000000  0.000000  0.000000  0.200000  0.400000
0.000000  0.000000
 0.000000  0.000000  0.000000  0.000000  0.000000  0.000000  0.000000  0.000000  0.000000
0.000000  0.000000  0.000000  0.000000  0.000000  0.000000  1.000000  0.000000  0.000000
0.000000  0.000000
 0.000000  0.000000  0.000000  0.000000  0.000000  0.000000  0.000000  0.000000  0.000000
0.000000  0.000000  0.000000  0.000000  0.000000  1.000000  0.000000  0.000000  0.000000
0.000000  0.000000
 0.000000  0.000000  0.000000  0.000000  0.000000  0.000000  0.000000  0.200000  0.000000
0.000000  0.000000  0.000000  0.000000  0.000000  0.200000  0.400000  0.000000  0.200000
0.000000  0.000000
 0.000000  0.000000  0.400000  0.600000  0.000000  0.000000  0.000000  0.000000  0.000000
0.000000  0.000000  0.000000  0.000000  0.000000  0.000000  0.000000  0.000000  0.000000
0.000000  0.000000
 0.000000  0.000000  0.000000  0.000000  0.000000  0.000000  1.000000  0.000000  0.000000
0.000000  0.000000  0.000000  0.000000  0.000000  0.000000  0.000000  0.000000  0.000000
0.000000  0.000000
```

```
 1.000000  0.000000  0.000000  0.000000  0.000000  0.000000  0.000000  0.000000  0.000000
0.000000  0.000000  0.000000  0.000000  0.000000  0.000000  0.000000  0.000000  0.000000
0.000000  0.000000
 0.000000  0.000000  0.000000  0.000000  0.000000  0.000000  0.000000  0.800000  0.000000
0.000000  0.000000  0.000000  0.000000  0.000000  0.000000  0.000000  0.000000  0.200000
0.000000  0.000000
 0.000000  0.000000  0.000000  0.000000  0.000000  0.000000  0.000000  0.000000  0.000000
0.000000  0.000000  0.000000  0.000000  0.000000  0.000000  0.000000  0.000000  1.000000
0.000000  0.000000
 0.000000  0.000000  0.000000  0.000000  0.000000  0.000000  0.000000  0.000000  0.000000
0.000000  0.000000  0.000000  0.000000  0.000000  0.000000  0.000000  0.000000  0.000000
0.000000  1.000000
 0.000000  0.000000  0.000000  0.000000  0.000000  0.000000  0.000000  0.000000  0.000000
0.000000  0.000000  0.000000  0.000000  0.000000  0.000000  0.000000  0.000000  0.000000
0.000000  1.000000
--------------------------------------------------------------------------------

--------------------------------------------------------------------------------
         Motif ATYDISRSEHAIVYY MEME-4 regular expression
--------------------------------------------------------------------------------
ATYD[IVT]SR[SIRV][ED]HA[IV]VYY
--------------------------------------------------------------------------------




Time  1.66 secs.

********************************************************************************


********************************************************************************
MOTIF MSSQRDRVNAFYKDN MEME-5 width =  15  sites =   5  llr = 193  E-value = 4.4e-023
********************************************************************************
--------------------------------------------------------------------------------
         Motif MSSQRDRVNAFYKDN MEME-5 Description
--------------------------------------------------------------------------------
Simplified        A  ::2::::::a:::::
pos.-specific     C  :::::::::::::::
probability       D  :::::8:::::::6:
matrix            E  :::::2:::::::::
                  F  :2::::::::a::::
                  G  ::::::::4::::2:
                  H  :::2:::::::::::
                  I  :::::::4:::::::
                  K  ::::::4:::::a::
                  L  :2:::::::::::::
                  M  a::::::::::::::
                  N  ::::::::6::::2a
                  P  ::22:::::::::::
                  Q  :::44::::::::::
                  R  ::::6:6::::::::
                  S  :66::::::::::::
                  T  :::::::::::::::
                  V  :::::::6:::::::
                  W  :::::::::::::::
                  Y  :::2:::::::a:::

         bits    6.3
                 5.6 *
                 5.0 *          *
                 4.4 *        **** *
Relative         3.8 *   **   **** *
Entropy          3.1 *  ************
(55.6 bits)      2.5 ***************
                 1.9 ***************
                 1.3 ***************
                 0.6 ***************
                 0.0 ---------------

Multilevel           MSSQRDRVNAFYKDN
```

```
consensus             FAHQEKIG    G
sequence              LPP         N
                        Y

--------------------------------------------------------------------------------

--------------------------------------------------------------------------------
         Motif MSSQRDRVNAFYKDN MEME-5 sites sorted by position p-value
--------------------------------------------------------------------------------
Sequence name            Start   P-value                Site
-------------            ----- ---------            ---------------
Measles                      1  3.82e-19          . MSPQRDRINAFYKDN PHPKGSRIVI
Rinderpest                   1  1.71e-18          . MSSPRDRVNAFYKDN LQFKNTRVVL
Phocine                      1  4.13e-17          . MFSHQDKVGAFYKNN ARANSSKLSP
Canine                       1  5.00e-17          . MLSYQDKVGAFYKDN ARANPSKLSL
Peste                        1  2.24e-16          . MSAQRERINAFYKGN PHNKNHRVIL
--------------------------------------------------------------------------------

--------------------------------------------------------------------------------
         Motif MSSQRDRVNAFYKDN MEME-5 block diagrams
--------------------------------------------------------------------------------
SEQUENCE NAME           POSITION P-VALUE  MOTIF DIAGRAM
-------------           ----------------  -------------
Measles                          3.8e-19  [5]_602
Rinderpest                       1.7e-18  [5]_594
Phocine                          4.1e-17  [5]_592
Canine                             5e-17  [5]_592
Peste                            2.2e-16  [5]_594
--------------------------------------------------------------------------------

--------------------------------------------------------------------------------
         Motif MSSQRDRVNAFYKDN MEME-5 in BLOCKS format
--------------------------------------------------------------------------------
BL   MOTIF MSSQRDRVNAFYKDN width=15 seqs=5
Measles                 (    1) MSPQRDRINAFYKDN  1
Rinderpest              (    1) MSSPRDRVNAFYKDN  1
Phocine                 (    1) MFSHQDKVGAFYKNN  1
Canine                  (    1) MLSYQDKVGAFYKDN  1
Peste                   (    1) MSAQRERINAFYKGN  1
//

--------------------------------------------------------------------------------

--------------------------------------------------------------------------------
         Motif MSSQRDRVNAFYKDN MEME-5 position-specific scoring matrix
--------------------------------------------------------------------------------
log-odds matrix: alength= 20 w= 15 n= 2979 bayes= 9.21626 E= 4.4e-023
  -363   -368   -520   -502   -375   -495   -453   -292   -440   -228    551   -488   -517   -416
-531   -471   -443   -324   -311   -351
   -55   -165   -288   -235    137   -258   -183   -135   -181     29    -44   -184   -302   -155
-239    263    -16   -114   -179   -100
   171   -154   -278   -247   -331   -178   -260   -364   -212   -360   -228   -183    116   -184
-272    281    -13   -263   -343   -344
   -97   -264   -181    -69   -148   -259    305   -309    -75   -253   -119   -106      8    385
-124   -170   -192   -253   -196    100
  -174   -313   -292   -166   -398   -300   -124   -372     71   -331   -223   -195   -340    283
339   -253   -256   -331   -287   -318
  -229   -375    395     13   -403   -332   -237   -430   -298   -442   -332    -76   -444   -231
-367   -310   -366   -379   -376   -376
  -197   -318   -347   -209   -428   -320   -135   -381    261   -342   -236   -216   -355    -32
338   -278   -274   -348   -292   -338
  -121   -211   -426   -376   -245   -420   -379    228   -350   -131    -86   -392   -428   -338
-402   -372   -229    281   -383   -365
  -129   -306    -66   -160   -388    236   -130   -451   -163   -451   -341    339   -328   -139
-271   -135   -220   -384   -377   -329
   418   -119   -363   -306   -318   -158   -328   -324   -298   -325   -204   -321   -385   -266
-339   -100   -211   -162   -330   -378
  -263   -252   -435   -407    440   -430   -342   -271   -397   -181   -167   -415   -415   -376
-451   -342   -415   -274   -165    -65
  -294   -338   -425   -407      0   -401   -130   -384   -364   -329   -268   -358   -440   -305
-382   -375   -407   -357    -99    451
```

```
  -275   -370   -454   -379   -513   -430   -341   -429    434   -481   -347   -338   -451   -289
-51   -425   -387   -457   -391   -469
  -202   -358    375    -66   -396     -6   -179   -444   -226   -446   -340     86   -389   -186
-320   -213   -290   -389   -380   -348
  -279   -329   -202   -351   -364   -315    -90   -362   -260   -430   -303    421   -392   -201
-342   -187   -270   -377   -315   -355
--------------------------------------------------------------------------------

--------------------------------------------------------------------------------
         Motif MSSQRDRVNAFYKDN MEME-5 position-specific probability matrix
--------------------------------------------------------------------------------
letter-probability matrix: alength= 20 w= 15 nsites= 5 E= 4.4e-023
 0.000000  0.000000  0.000000  0.000000  0.000000  0.000000  0.000000  0.000000  0.000000
0.000000  1.000000  0.000000  0.000000  0.000000  0.000000  0.000000  0.000000  0.000000
0.000000  0.000000
 0.000000  0.000000  0.000000  0.000000  0.200000  0.000000  0.000000  0.000000  0.000000
0.200000  0.000000  0.000000  0.000000  0.000000  0.000000  0.600000  0.000000  0.000000
0.000000  0.000000
 0.200000  0.000000  0.000000  0.000000  0.000000  0.000000  0.000000  0.000000  0.000000
0.000000  0.000000  0.000000  0.200000  0.000000  0.000000  0.600000  0.000000  0.000000
0.000000  0.000000
 0.000000  0.000000  0.000000  0.000000  0.000000  0.000000  0.200000  0.000000  0.000000
0.000000  0.000000  0.000000  0.200000  0.400000  0.000000  0.000000  0.000000  0.000000
0.000000  0.200000
 0.000000  0.000000  0.000000  0.000000  0.000000  0.000000  0.000000  0.000000  0.000000
0.000000  0.000000  0.000000  0.000000  0.400000  0.600000  0.000000  0.000000  0.000000
0.000000  0.000000
 0.000000  0.000000  0.800000  0.200000  0.000000  0.000000  0.000000  0.000000  0.000000
0.000000  0.000000  0.000000  0.000000  0.000000  0.000000  0.000000  0.000000  0.000000
0.000000  0.000000
 0.000000  0.000000  0.000000  0.000000  0.000000  0.000000  0.000000  0.000000  0.400000
0.000000  0.000000  0.000000  0.000000  0.000000  0.600000  0.000000  0.000000  0.000000
0.000000  0.000000
 0.000000  0.000000  0.000000  0.000000  0.000000  0.000000  0.000000  0.400000  0.000000
0.000000  0.000000  0.000000  0.000000  0.000000  0.000000  0.000000  0.000000  0.600000
0.000000  0.000000
 0.000000  0.000000  0.000000  0.000000  0.000000  0.400000  0.000000  0.000000  0.000000
0.000000  0.000000  0.600000  0.000000  0.000000  0.000000  0.000000  0.000000  0.000000
0.000000  0.000000
 1.000000  0.000000  0.000000  0.000000  0.000000  0.000000  0.000000  0.000000  0.000000
0.000000  0.000000  0.000000  0.000000  0.000000  0.000000  0.000000  0.000000  0.000000
0.000000  0.000000
 0.000000  0.000000  0.000000  0.000000  1.000000  0.000000  0.000000  0.000000  0.000000
0.000000  0.000000  0.000000  0.000000  0.000000  0.000000  0.000000  0.000000  0.000000
0.000000  0.000000
 0.000000  0.000000  0.000000  0.000000  0.000000  0.000000  0.000000  0.000000  0.000000
0.000000  0.000000  0.000000  0.000000  0.000000  0.000000  0.000000  0.000000  0.000000
0.000000  1.000000
 0.000000  0.000000  0.000000  0.000000  0.000000  0.000000  0.000000  0.000000  1.000000
0.000000  0.000000  0.000000  0.000000  0.000000  0.000000  0.000000  0.000000  0.000000
0.000000  0.000000
 0.000000  0.000000  0.600000  0.000000  0.000000  0.200000  0.000000  0.000000  0.000000
0.000000  0.000000  0.200000  0.000000  0.000000  0.000000  0.000000  0.000000  0.000000
0.000000  0.000000
 0.000000  0.000000  0.000000  0.000000  0.000000  0.000000  0.000000  0.000000  0.000000
0.000000  0.000000  1.000000  0.000000  0.000000  0.000000  0.000000  0.000000  0.000000
0.000000  0.000000
--------------------------------------------------------------------------------

--------------------------------------------------------------------------------
         Motif MSSQRDRVNAFYKDN MEME-5 regular expression
--------------------------------------------------------------------------------
M[SFL][SAP][QHPY][RQ][DE][RK][VI][NG]AFYK[DGN]N
--------------------------------------------------------------------------------




Time  2.06 secs.

********************************************************************************
```

```
********************************************************************************
MOTIF HRGIIKDSEATWAVP MEME-6 width =  15  sites =   5  llr = 189  E-value = 9.7e-022
********************************************************************************
--------------------------------------------------------------------------------
         Motif HRGIIKDSEATWAVP MEME-6 Description
--------------------------------------------------------------------------------
Simplified        A  :::::22::a::4::
pos.-specific     C  :::::::::::::::
probability       D  ::::::82:::::::
matrix            E  :::::::2:::::::
                  F  :::4:::::::::::
                  G  ::a:::2::::::::
                  H  a::::::::::::::
                  I  :::4a:::2:2::::
                  K  :::::6::2:2::::
                  L  :::::::::::::::
                  M  ::::::::::::4::
                  N  :::::::2::2::::
                  P  ::::::::::::::a
                  Q  :::::::2:::::::
                  R  :a:::::::::::::
                  S  :::::::4:::::::
                  T  ::::::::::4::::
                  V  :::2:2::2:::2a:
                  W  :::::::::::a:::
                  Y  :::::::::::::::

         bits    6.3           *
                 5.6 *         *
                 5.0 *         *
                 4.4 **       * *  *
Relative         3.8 *** * *  * * **
Entropy          3.1 *** ***  * ****
(54.6 bits)      2.5 *******  * ****
                 1.9 ***************
                 1.3 ***************
                 0.6 ***************
                 0.0 ---------------

Multilevel           HRGFIKDSEATWAVP
consensus               I AADI I M
sequence               V V GK K V
                           NQ N
                            V
--------------------------------------------------------------------------------

--------------------------------------------------------------------------------
         Motif HRGIIKDSEATWAVP MEME-6 sites sorted by position p-value
--------------------------------------------------------------------------------
Sequence name            Start   P-value                Site
-------------            ----- ---------            ---------------
Phocine                    350  3.75e-19 PSIEKIHITN HRGFIKDSVATWMVP ALALSEQGEQ
Canine                     350  4.58e-19 PSMERIHITN HRGFIKDSIATWMVP ALASEKQEEQ
Peste                      354  1.94e-17 LMVEKLYLSS HRGIIKDDEANWVVP STDVRDLQNK
Measles                    354  4.20e-17 PVIDRLYLSS HRGVIADNQAKWAVP TTRTDDKLRM
Rinderpest                 354  1.39e-14 PKLDGLYITT HRGIIVAGKAIWAVP VTRTDDQLRM
--------------------------------------------------------------------------------

--------------------------------------------------------------------------------
         Motif HRGIIKDSEATWAVP MEME-6 block diagrams
--------------------------------------------------------------------------------
SEQUENCE NAME           POSITION P-VALUE  MOTIF DIAGRAM
-------------           ----------------  -------------
Phocine                          3.7e-19  349_[6]_243
Canine                           4.6e-19  349_[6]_243
Peste                            1.9e-17  353_[6]_241
Measles                          4.2e-17  353_[6]_249
Rinderpest                       1.4e-14  353_[6]_241
--------------------------------------------------------------------------------
```

```
--------------------------------------------------------------------------------
         Motif HRGIIKDSEATWAVP MEME-6 in BLOCKS format
--------------------------------------------------------------------------------
BL   MOTIF HRGIIKDSEATWAVP width=15 seqs=5
Phocine                  (  350) HRGFIKDSVATWMVP  1
Canine                   (  350) HRGFIKDSIATWMVP  1
Peste                    (  354) HRGIIKDDEANWVVP  1
Measles                  (  354) HRGVIADNQAKWAVP  1
Rinderpest               (  354) HRGIIVAGKAIWAVP  1
//

--------------------------------------------------------------------------------

--------------------------------------------------------------------------------
         Motif HRGIIKDSEATWAVP MEME-6 position-specific scoring matrix
--------------------------------------------------------------------------------
log-odds matrix: alength= 20 w= 15 n= 2979 bayes= 9.46819 E= 9.7e-022
  -292   -325   -288   -311   -248   -357    517   -466   -328   -371   -257   -137   -389    -43
-222   -294   -328   -394   -253   -113
  -270   -290   -427   -401   -449   -379   -187   -435    -79   -414   -335   -315   -371   -166
399   -367   -383   -467   -283   -414
  -133   -345   -267   -310   -438    387   -315   -484   -280   -504   -350   -236   -405   -304
-324   -260   -370   -405   -358   -415
  -137   -199   -443   -372    266   -384   -323    229   -331    -73    -22   -363   -415   -287
-380   -327   -222    152   -266   -225
  -215   -290   -415   -386   -236   -421   -397    331   -332    -81    -46   -373   -459   -325
-410   -366   -282     63   -346   -319
    97   -259   -232   -129   -298   -260   -162   -245    377   -269   -156   -181   -307    -61
-40   -196   -197     17   -291   -284
   -56   -361    395    -52   -399   -318   -234   -428   -298   -441   -331    -73   -439   -239
-362   -294   -354   -374   -374   -373
  -113   -310    170    -81   -359    134    -88   -435    -92   -407   -294    200   -281    -68
-211    196   -146   -366   -369   -282
     4   -222    -78    145   -203   -167    -50     59    167   -183    -66    -63   -204    223
-51    -81    -92     80   -218   -178
   418   -119   -363   -306   -318   -158   -328   -324   -298   -325   -204   -321   -385   -266
-339   -100   -211   -162   -330   -378
   -35   -203   -138    -64   -222   -205    -99     35    143   -215    -82    117   -246     -3
-93    -38    237   -141   -239   -217
  -322   -340   -404   -385   -126   -394   -339   -431   -351   -276   -254   -380   -458   -295
-359   -414   -411   -362    609   -169
   289   -122   -337   -249   -142   -217   -227    -50   -215   -102    347   -261   -321   -177
-266   -155   -145    129   -218   -215
   -56   -190   -377   -325   -268   -366   -308    -29   -315   -207   -146   -369   -367   -300
-338   -331   -197    349   -370   -390
  -107   -333   -313   -272   -371   -307   -284   -398   -239   -361   -300   -325    396   -204
-308   -238   -277   -328   -402   -419
--------------------------------------------------------------------------------

--------------------------------------------------------------------------------
         Motif HRGIIKDSEATWAVP MEME-6 position-specific probability matrix
--------------------------------------------------------------------------------
letter-probability matrix: alength= 20 w= 15 nsites= 5 E= 9.7e-022
 0.000000  0.000000  0.000000  0.000000  0.000000  0.000000  1.000000  0.000000  0.000000
0.000000  0.000000  0.000000  0.000000  0.000000  0.000000  0.000000  0.000000  0.000000
0.000000  0.000000
 0.000000  0.000000  0.000000  0.000000  0.000000  0.000000  0.000000  0.000000  0.000000
0.000000  0.000000  0.000000  0.000000  0.000000  1.000000  0.000000  0.000000  0.000000
0.000000  0.000000
 0.000000  0.000000  0.000000  0.000000  0.000000  1.000000  0.000000  0.000000  0.000000
0.000000  0.000000  0.000000  0.000000  0.000000  0.000000  0.000000  0.000000  0.000000
0.000000  0.000000
 0.000000  0.000000  0.000000  0.000000  0.400000  0.000000  0.000000  0.400000  0.000000
0.000000  0.000000  0.000000  0.000000  0.000000  0.000000  0.000000  0.000000  0.200000
0.000000  0.000000
 0.000000  0.000000  0.000000  0.000000  0.000000  0.000000  0.000000  1.000000  0.000000
0.000000  0.000000  0.000000  0.000000  0.000000  0.000000  0.000000  0.000000  0.000000
0.000000  0.000000
```

```
 0.200000  0.000000  0.000000  0.000000  0.000000  0.000000  0.000000  0.000000  0.600000
0.000000  0.000000  0.000000  0.000000  0.000000  0.000000  0.000000  0.000000  0.200000
0.000000  0.000000
 0.200000  0.000000  0.800000  0.000000  0.000000  0.000000  0.000000  0.000000  0.000000
0.000000  0.000000  0.000000  0.000000  0.000000  0.000000  0.000000  0.000000  0.000000
0.000000  0.000000
 0.000000  0.000000  0.200000  0.000000  0.000000  0.200000  0.000000  0.000000  0.000000
0.000000  0.000000  0.200000  0.000000  0.000000  0.000000  0.400000  0.000000  0.000000
0.000000  0.000000
 0.000000  0.000000  0.000000  0.200000  0.000000  0.000000  0.000000  0.200000  0.200000
0.000000  0.000000  0.000000  0.000000  0.200000  0.000000  0.000000  0.000000  0.200000
0.000000  0.000000
 1.000000  0.000000  0.000000  0.000000  0.000000  0.000000  0.000000  0.000000  0.000000
0.000000  0.000000  0.000000  0.000000  0.000000  0.000000  0.000000  0.000000  0.000000
0.000000  0.000000
 0.000000  0.000000  0.000000  0.000000  0.000000  0.000000  0.000000  0.200000  0.200000
0.000000  0.000000  0.200000  0.000000  0.000000  0.000000  0.000000  0.400000  0.000000
0.000000  0.000000
 0.000000  0.000000  0.000000  0.000000  0.000000  0.000000  0.000000  0.000000  0.000000
0.000000  0.000000  0.000000  0.000000  0.000000  0.000000  0.000000  0.000000  0.000000
1.000000  0.000000
 0.400000  0.000000  0.000000  0.000000  0.000000  0.000000  0.000000  0.000000  0.000000
0.000000  0.400000  0.000000  0.000000  0.000000  0.000000  0.000000  0.000000  0.200000
0.000000  0.000000
 0.000000  0.000000  0.000000  0.000000  0.000000  0.000000  0.000000  0.000000  0.000000
0.000000  0.000000  0.000000  0.000000  0.000000  0.000000  0.000000  0.000000  1.000000
0.000000  0.000000
 0.000000  0.000000  0.000000  0.000000  0.000000  0.000000  0.000000  0.000000  0.000000
0.000000  0.000000  0.000000  1.000000  0.000000  0.000000  0.000000  0.000000  0.000000
0.000000  0.000000
--------------------------------------------------------------------------------

--------------------------------------------------------------------------------
         Motif HRGIIKDSEATWAVP MEME-6 regular expression
--------------------------------------------------------------------------------
HRG[FIV]I[KAV][DA][SDGN][EIKQV]A[TIKN]W[AMV]VP
--------------------------------------------------------------------------------




Time  2.48 secs.

********************************************************************************


********************************************************************************
SUMMARY OF MOTIFS
********************************************************************************

--------------------------------------------------------------------------------
         Combined block diagrams: non-overlapping sites with p-value < 0.0001
--------------------------------------------------------------------------------
SEQUENCE NAME            COMBINED P-VALUE  MOTIF DIAGRAM
-------------            ----------------  -------------
Peste                            1.70e-78  [5(2.24e-16)]_70_[3(9.16e-19)]_28_\
    [2(3.07e-19)]_210_[6(1.94e-17)]_11_[1(2.40e-05)]_132_[4(4.90e-19)]_27_\
    [1(2.30e-17)]_26
Measles                          5.74e-83  [5(3.82e-19)]_70_[3(2.17e-19)]_28_\
    [2(1.23e-19)]_210_[6(4.20e-17)]_70_[4(9.29e-05)]_73_[4(8.38e-18)]_27_\
    [1(9.25e-20)]_34
Canine                           6.21e-83  [5(5.00e-17)]_70_[3(1.17e-17)]_28_\
    [2(1.23e-20)]_206_[6(4.58e-19)]_158_[4(5.63e-19)]_27_[1(2.15e-19)]_28
Phocine                          1.40e-83  [5(4.13e-17)]_70_[3(2.17e-19)]_28_\
    [2(2.16e-19)]_206_[6(3.75e-19)]_158_[4(6.90e-19)]_27_[1(1.73e-19)]_28
Rinderpest                       2.53e-78  [5(1.71e-18)]_70_[3(1.99e-18)]_28_\
    [2(1.06e-16)]_210_[6(1.39e-14)]_158_[4(3.28e-19)]_27_[1(1.26e-20)]_26
--------------------------------------------------------------------------------

********************************************************************************
```

```
********************************************************************************
Stopped because requested number of motifs (6) found.
********************************************************************************

CPU: noble-meme.grid.gs.washington.edu

********************************************************************************
```

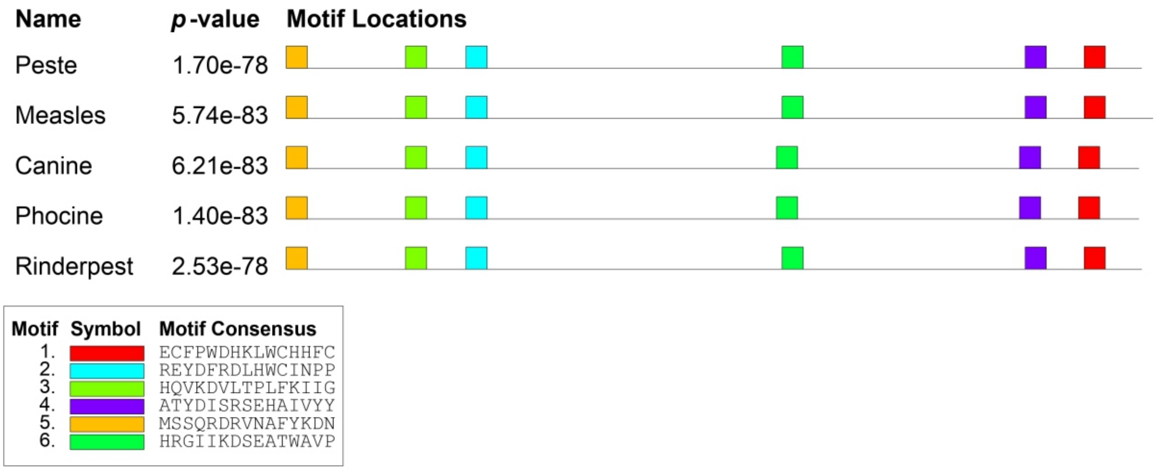

Supplementary file 7(B): Sequence and localization of different motifs in Hemagglutinin protein in the genus *Morbillivirus*
