# Supplementary material for: Structural and functional characterization of peste des petits ruminants virus coded hemagglutinin protein using various in-silico approaches
Source: Front Microbiol. 2024 Jun 20;15:1427606. doi: 10.3389/fmicb.2024.1427606 (PMC11222573; doi:10.3389/fmicb.2024.1427606)
Supplement: Supplementary file 9 [file Data_Sheet_9.ZIP › Ikk-alpha.pdf]

SWISS-MODEL Homology Modelling Report

Model Building Report

This document lists the results for the homology modelling project "Untitled Project" submitted to SWISS-MODEL workspace on Jan. 10, 2024, 6:44 a.m..The submitted primary amino acid sequence is given in Table T1.

If you use any results in your research, please cite the relevant publications:

- Waterhouse, A., Bertoni, M., Bienert, S., Studer, G., Tauriello, G., Gumienny, R., Heer, F.T., de Beer, T.A.P., Rempfer, C., Bordoli, L., Lepore, R., Schwede, T. SWISS-MODEL: homology modelling of protein structures and complexes. Nucleic Acids Res. 46(W1), W296-W303 (2018).
- Bienert, S., Waterhouse, A., de Beer, T.A.P., Tauriello, G., Studer, G., Bordoli, L., Schwede, T. The SWISS-MODEL Repository - new features and functionality. Nucleic Acids Res. 45, D313-D319 (2017).
- Studer, G., Tauriello, G., Bienert, S., Biasini, M., Johner, N., Schwede, T. ProMod3 - A versatile homology modelling toolbox. PLOS Comp. Biol. 17(1), e1008667 (2021).
- Studer, G., Rempfer, C., Waterhouse, A.M., Gumienny, G., Haas, J., Schwede, T. QMEANDisCo - distance constraints applied on model quality estimation. Bioinformatics 36, 1765-1771 (2020).
- Bertoni, M., Kiefer, F., Biasini, M., Bordoli, L., Schwede, T. Modeling protein quaternary structure of homo- and hetero-oligomers beyond binary interactions by homology. Scientific Reports 7 (2017).

Results

The SWISS-MODEL template library (SMTL version 2023-12-24, PDB release 2023-12-15) was searched with for evolutionary related structures matching the target sequence in Table T1. For details on the template search, see Materials and Methods. Overall 6564 templates were found (Table T2).

Models

The following model was built (see Materials and Methods "Model Building"):

Model #01	File	Built with	Oligo-State	Ligands	GMQE	QMEANDisCo Global
	PDB	ProMod3 3.4.0	homo-dimer (matching prediction)	None	0.68	0.71 ± 0.05

Template	Seq Identity	Oligo-state	QSQE	Found by	Method	Resolution	Seq Similarity	Range	Coverage	Description
5tqw.1.A	98.31	homo-dimer	0.67	HHblits	EM	-	0.61	14 - 660	0.88	Inhibitor of nuclear factor kappa-B kinase subunit alpha

The template contained no ligands.

```
Target    MERPPGLRPGAGGPWEMRERLGTGGFGNVCLYQHRELDLKIAIKSCRLELSTKNRERWCHEIQIMKKLNHANVVKACDVP
5tqw.1.A  ---------GAGGPWEMRERLGTGGFGNVCLYQHRELDLKIAIKSCRLELSTKNRERWCHEIQIMKKLNHANVVKACDVP

Target    EELNFLINDVPLLAMEYCSGGDLRKLLNKPENCCGLKESQILSLLSDIGSGIRYLHENKIIHRDLKPENIVLQDVGGKIM
5tqw.1.A  EELNILIHDVPLLAMEYCSGGDLRKLLNKPENCCGLKESQILSLLSDIGSGIRYLHENKIIHRDLKPENIVLQDVGGKII

Target    HKIIDLGYAKDVDQGSLCTSFVGTLQYLAPELFENKPYTATVDYWSFGTMVFECIAGYRPFLHHLQPFTWHEKIKKKDPK
5tqw.1.A  HKIIDLGYAKDVDQGELCTEFVGTLQYLAPELFENKPYTATVDYWSFGTMVFECIAGYRPFLHHLQPFTWHEKIKKKDPK

Target    CIFACEEMTGEVRFSSHLPQPNSLCSLIVEPMENWLQLMLNWDPQQRGGPVDLTLKQPRCFVLMDHILNLKIVHILNMTS
5tqw.1.A  CIFACEEMSGEVRFSSHLPQPNSLCSLIVEPMENWLQLMLNWDPQQRGGPVDLTLKQPRCFVLMDHILNLKIVHILNMTS

Target    AKIISFLLPPDESLHSLQSRIERETGINTGSQELLSEMGISLDPRKPASQCVLDGVRGCDSYMVYLFDKSKTVYEGPFAS
5tqw.1.A  AKIISFLLPPDESLHSLQSRIERETGINTGSQELLSETGISLDPRKPASQCVLDGVRGCDSYMVYLFDKSKTVYEGPFAS

Target    RSLSDCVNYIVQDSKIQLPIIQLRKVWAEAVHYVSGLKEDYSRLFQGQRAAMLSLLRYNTNLTKMKNTLISASQQLKAKL
5tqw.1.A  RSLSDCVNYIVQDSKIQLPIIQLRKVWAEAVHYVSGLKEDYSRLFQGQRAAMLSLLRYNANLTKMKNTLISASQQLKAKL
```

```
Target     EFFHKSIQLDLERYSEQMTYGISSEKMLKAWKEMEEKAIHYAEVGVIGYLEDQIMSLHTEIMELQKSPYGRRQGDLMESL
5tqw.1.A   EFFHKSIQLDLERYSEQMTYGISSEKMLKAWKEMEEKAIHYAEVGVIGYLEDQIMSLHAEIMELQKSPYGRRQGDLMESL

Target     EQRAIDLYKQLKHRPSDHSYSDSTEMVKIIVHTVQSQDRVLKELFGHLSKLLGCKQKIIDLLPKVEMALSNIKEADSTVM
5tqw.1.A   EQRAIDLYKQLKHRPSDHSYSDSTEMVKIIVHTVQSQDRVLKELFGHLSKLLGCKQKIIDLLPKVEVALSNIKEADNTVM

Target     FMQGKRQKEIWHLLKIACTQSSARSLVGSSLEGVTPQLPPTSAEREHPLSCVVTPQDGETLAQMIEENLNCLGHLSTIIH
5tqw.1.A   FMQGKRQKEIWHLLKIACTQ------------------------------------------------------------

Target     EANEKQGNNMMSLDWSWLTE
5tqw.1.A   --------------------


Target     MERPPGLRPGAGGPWEMRERLGTGGFGNVCLYQHRELDLKIAIKSCRLELSTKNRERWCHEIQIMKKLNHANVVKACDVP
5tqw.1.B   ---------GAGGPWEMRERLGTGGFGNVCLYQHRELDLKIAIKSCRLELSTKNRERWCHEIQIMKKLNHANVVKACDVP

Target     EELNFLINDVPLLAMEYCSGGDLRKLLNKPENCCGLKESQILSLLSDIGSGIRYLHENKIIHRDLKPENIVLQDVGGKIM
5tqw.1.B   EELNILIHDVPLLAMEYCSGGDLRKLLNKPENCCGLKESQILSLLSDIGSGIRYLHENKIIHRDLKPENIVLQDVGGKII

Target     HKIIDLGYAKDVDQGSLCTSFVGTLQYLAPELFENKPYTATVDYWSFGTMVFECIAGYRPFLHHLQPFTWHEKIKKKDPK
5tqw.1.B   HKIIDLGYAKDVDQGELCTEFVGTLQYLAPELFENKPYTATVDYWSFGTMVFECIAGYRPFLHHLQPFTWHEKIKKKDPK

Target     CIFACEEMTGEVRFSSHLPQPNSLCSLIVEPMENWLQLMLNWDPQQRGGPVDLTLKQPRCFVLMDHILNLKIVHILNMTS
5tqw.1.B   CIFACEEMSGEVRFSSHLPQPNSLCSLIVEPMENWLQLMLNWDPQQRGGPVDLTLKQPRCFVLMDHILNLKIVHILNMTS

Target     AKIISFLLPPDESLHSLQSRIERETGINTGSQELLSEMGISLDPRKPASQCVLDGVRGCDSYMVYLFDKSKTVYEGPFAS
5tqw.1.B   AKIISFLLPPDESLHSLQSRIERETGINTGSQELLSETGISLDPRKPASQCVLDGVRGCDSYMVYLFDKSKTVYEGPFAS

Target     RSLSDCVNYIVQDSKIQLPIIQLRKVWAEAVHYVSGLKEDYSRLFQGQRAAMLSLLRYNTNLTKMKNTLISASQQLKAKL
5tqw.1.B   RSLSDCVNYIVQDSKIQLPIIQLRKVWAEAVHYVSGLKEDYSRLFQGQRAAMLSLLRYNANLTKMKNTLISASQQLKAKL

Target     EFFHKSIQLDLERYSEQMTYGISSEKMLKAWKEMEEKAIHYAEVGVIGYLEDQIMSLHTEIMELQKSPYGRRQGDLMESL
5tqw.1.B   EFFHKSIQLDLERYSEQMTYGISSEKMLKAWKEMEEKAIHYAEVGVIGYLEDQIMSLHAEIMELQKSPYGRRQGDLMESL

Target     EQRAIDLYKQLKHRPSDHSYSDSTEMVKIIVHTVQSQDRVLKELFGHLSKLLGCKQKIIDLLPKVEMALSNIKEADSTVM
5tqw.1.B   EQRAIDLYKQLKHRPSDHSYSDSTEMVKIIVHTVQSQDRVLKELFGHLSKLLGCKQKIIDLLPKVEVALSNIKEADNTVM

Target     FMQGKRQKEIWHLLKIACTQSSARSLVGSSLEGVTPQLPPTSAEREHPLSCVVTPQDGETLAQMIEENLNCLGHLSTIIH
5tqw.1.B   FMQGKRQKEIWHLLKIACTQ------------------------------------------------------------

Target     EANEKQGNNMMSLDWSWLTE
5tqw.1.B   --------------------
```

Materials and Methods

Template Search

Template search with has been performed against the SWISS-MODEL template library (SMTL, last update: 2023-12-24, last included PDB release: 2023-12-15).

Model Building

Models are built based on the target-template alignment using ProMod3 (Studer et al.). Coordinates which are conserved between the target and the template are copied from the template to the model. Insertions and deletions are remodelled using a fragment library. Side chains are then rebuilt. Finally, the geometry of the resulting model is regularized by using a force field.

Model Quality Estimation

The global and per-residue model quality has been assessed using the QMEAN scoring function (Studer et al.).

Ligand Modelling

Ligands present in the template structure are transferred by homology to the model when the following criteria are met: (a) The ligands are annotated as biologically relevant in the template library, (b) the ligand is in contact with the model, (c) the ligand is not clashing with the protein, (d) the residues in contact with the ligand are conserved between the target and the template. If any of these four criteria is not satisfied, a certain ligand will not be included in the model. The model summary includes information on why and which ligand has not been included.

Oligomeric State Conservation

The quaternary structure annotation of the template is used to model the target sequence in its oligomeric form. The method (Bertoni et al.) is based on a supervised machine learning algorithm, Support Vector Machines (SVM), which combines interface conservation, structural clustering, and other template features to provide a quaternary structure quality estimate (QSQE). The QSQE score is a number between 0 and 1, reflecting the expected accuracy of the interchain contacts for a model built based a given alignment and template. Higher numbers indicate higher reliability. This complements the GMQE score which estimates the accuracy of the tertiary structure of the resulting model.

References

- **BLAST**
  Camacho, C., Coulouris, G., Avagyan, V., Ma, N., Papadopoulos, J., Bealer, K., Madden, T.L. BLAST+: architecture and applications. BMC Bioinformatics 10, 421-430 (2009).
- **HHblits**
  Steinegger, M., Meier, M., Mirdita, M., Vöhringer, H., Haunsberger, S. J., Söding, J. HH-suite3 for fast remote homology detection and deep protein annotation. BMC Bioinformatics 20, 473 (2019).

Table T1:

Primary amino acid sequence for which templates were searched and models were built.

```
MERPPGLRPGAGGPWEMRERLGTGGFGNVCLYQHRELDLKIAIKSCRLELSTKNRERWCHEIQIMKKLNHANVVKACDVPEELNFLINDVPLLAMEYCSG
GDLRKLLNKPENCCGLKESQILSLLSDIGSGIRYLHENKIIHRDLKPENIVLQDVGGKIMHKIIDLGYAKDVDQGSLCTSFVGTLQYLAPELFENKPYTA
TVDYWSFGTMVFECIAGYRPFLHHLQPFTWHEKIKKKDPKCIFACEEMTGEVRFSSHLPQPNSLCSLIVEPMENWLQLMLNWDPQQRGGPVDLTLKQPRC
FVLMDHILNLKIVHILNMTSAKIISFLLPPDESLHSLQSRIERETGINTGSQELLSEMGISLDPRKPASQCVLDGVRGCDSYMVYLFDKSKTVYEGPFAS
RSLSDCVNYIVQDSKIQLPIIQLRKVWAEAVHYVSGLKEDYSRLFQGQRAAMLSLLRYNTNLTKMKNTLISASQQLKAKLEFFHKSIQLDLERYSEQMTY
GISSEKMLKAWKEMEEKAIHYAEVGVIGYLEDQIMSLHTEIMELQKSPYGRRQGDLMESLEQRAIDLYKQLKHRPSDHSYSDSTEMVKIIVHTVQSQDRV
LKELFGHLSKLLGCKQKIIDLLPKVEMALSNIKEADSTVMFMQGKRQKEIWHLLKIACTQSSARSLVGSSLEGVTPQLPPTSAEREHPLSCVVTPQDGET
LAQMIEENLNCLGHLSTIIHEANEKQGNNMMSLDWSWLTE
```

Table T2:

Template	Seq Identity	Oligo-state	QSQE	Found by	Method	Resolution	Seq Similarity	Coverage	Description
5tqw.1.A	98.31	homo-dimer	0.67	HHblits	EM	NA	0.61	0.88	Inhibitor of nuclear factor kappa-B kinase subunit alpha
5tqx.1.A	98.31	homo-dimer	0.66	HHblits	EM	NA	0.61	0.88	Inhibitor of nuclear factor kappa-B kinase subunit alpha
5ebz.1.A	98.30	homo-hexamer	0.50	BLAST	X-ray	4.50Å	0.61	0.87	Inhibitor of nuclear factor kappa-B kinase subunit alpha
5ebz.1.A	98.31	homo-hexamer	0.49	HHblits	X-ray	4.50Å	0.61	0.88	Inhibitor of nuclear factor kappa-B kinase subunit alpha
5ebz.2.F	98.31	homo-hexamer	0.49	HHblits	X-ray	4.50Å	0.61	0.88	Inhibitor of nuclear factor kappa-B kinase subunit alpha
5ebz.2.E	98.31	homo-hexamer	0.49	HHblits	X-ray	4.50Å	0.61	0.88	Inhibitor of nuclear factor kappa-B kinase subunit alpha
5ebz.1.F	98.31	homo-hexamer	0.49	HHblits	X-ray	4.50Å	0.61	0.88	Inhibitor of nuclear factor kappa-B kinase subunit alpha
O15111.1.A	97.70	monomer	-	AFDB search	AlphaFold v2	NA	0.61	1.00	Inhibitor of nuclear factor kappa-B kinase subunit alpha

Template	Seq Identity	Oligo-state	QSQE	Found by	Method	Resolution	Seq Similarity	Coverage	Description
5tqy.1.A	98.31	homo-dimer	0.67	HHblits	EM	NA	0.61	0.88	Inhibitor of nuclear factor kappa-B kinase subunit alpha
4kik.1.B	54.69	homo-dimer	0.68	HHblits	X-ray	2.83Å	0.46	0.88	Inhibitor of nuclear factor kappa-B kinase subunit beta
4kik.1.A	54.69	homo-dimer	0.67	HHblits	X-ray	2.83Å	0.46	0.88	Inhibitor of nuclear factor kappa-B kinase subunit beta
4e3c.1.A	54.46	homo-dimer	0.56	HHblits	X-ray	3.98Å	0.46	0.88	Inhibitor of nuclear factor kappa-B kinase subunit beta
4e3c.1.B	54.46	homo-dimer	0.56	HHblits	X-ray	3.98Å	0.46	0.88	Inhibitor of nuclear factor kappa-B kinase subunit beta
4e3c.3.A	54.46	homo-dimer	0.54	HHblits	X-ray	3.98Å	0.46	0.88	Inhibitor of nuclear factor kappa-B kinase subunit beta
4e3c.3.B	54.46	homo-dimer	0.54	HHblits	X-ray	3.98Å	0.46	0.88	Inhibitor of nuclear factor kappa-B kinase subunit beta
4e3c.2.A	54.46	homo-dimer	0.50	HHblits	X-ray	3.98Å	0.46	0.88	Inhibitor of nuclear factor kappa-B kinase subunit beta
4e3c.2.B	54.46	homo-dimer	0.49	HHblits	X-ray	3.98Å	0.46	0.88	Inhibitor of nuclear factor kappa-B kinase subunit beta
4im0.1.A	27.57	homo-dimer	0.23	HHblits	X-ray	2.40Å	0.34	0.46	Serine/threonine-protein kinase TBK1
4eut.1.B	27.73	homo-dimer	0.21	HHblits	X-ray	2.60Å	0.34	0.46	Serine/threonine-protein kinase TBK1
4iw0.1.A	27.73	homo-dimer	0.18	HHblits	X-ray	4.00Å	0.34	0.46	Serine/threonine-protein kinase TBK1
4jlc.1.B	27.43	homo-dimer	0.12	HHblits	X-ray	3.00Å	0.34	0.46	Serine/threonine-protein kinase TBK1
4jl9.1.A	27.43	homo-dimer	0.11	HHblits	X-ray	3.10Å	0.34	0.46	Serine/threonine-protein kinase TBK1
6o8b.1.C	31.64	homo-dimer	0.13	BLAST	X-ray	3.40Å	0.36	0.50	Serine/threonine-protein kinase TBK1
6o8b.1.D	31.64	homo-dimer	0.13	BLAST	X-ray	3.40Å	0.36	0.50	Serine/threonine-protein kinase TBK1
6o8b.1.C	27.43	homo-dimer	0.14	HHblits	X-ray	3.40Å	0.34	0.46	Serine/threonine-protein kinase TBK1
4eut.1.A	27.73	homo-dimer	0.21	HHblits	X-ray	2.60Å	0.34	0.46	Serine/threonine-protein kinase TBK1
5w5v.1.A	32.00	homo-dimer	0.23	BLAST	X-ray	3.64Å	0.36	0.44	Serine/threonine-protein kinase TBK1
6rst.1.A	32.00	homo-dimer	0.20	BLAST	X-ray	3.29Å	0.36	0.44	Serine/threonine-protein kinase TBK1
6rsr.1.A	32.00	homo-dimer	0.14	BLAST	X-ray	3.15Å	0.36	0.44	Serine/threonine-protein kinase TBK1
4im3.1.A	27.57	homo-dimer	0.12	HHblits	X-ray	3.34Å	0.34	0.46	Serine/threonine-protein kinase TBK1
6bod.1.A	32.00	monomer	-	BLAST	X-ray	3.20Å	0.36	0.44	Serine/threonine-protein kinase TBK1
6boe.1.A	32.00	monomer	-	BLAST	X-ray	3.60Å	0.36	0.44	Serine/threonine-protein kinase TBK1
6bny.1.A	32.00	monomer	-	BLAST	X-ray	3.34Å	0.36	0.44	Serine/threonine-protein kinase TBK1

Template	Seq Identity	Oligo-state	QSQE	Found by	Method	Resolution	Seq Similarity	Coverage	Description
1oi9.2.A	25.98	monomer	-	HHblits	X-ray	2.10Å	0.33	0.38	CELL DIVISION PROTEIN KINASE 2
3zu7.1.A	24.73	monomer	-	HHblits	X-ray	1.97Å	0.32	0.38	MITOGEN-ACTIVATED PROTEIN KINASE 1
2erk.1.A	24.73	monomer	-	HHblits	X-ray	2.40Å	0.32	0.38	EXTRACELLULAR SIGNAL-REGULATED KINASE 2
4xj0.2.A	24.73	monomer	-	HHblits	X-ray	2.58Å	0.32	0.38	Mitogen-activated protein kinase 1
4qyy.1.A	24.73	monomer	-	HHblits	X-ray	1.65Å	0.32	0.38	Mitogen-activated protein kinase 1
4xj0.1.A	24.73	monomer	-	HHblits	X-ray	2.58Å	0.32	0.38	Mitogen-activated protein kinase 1
5ke0.1.A	24.30	monomer	-	HHblits	X-ray	1.68Å	0.32	0.38	Mitogen-activated protein kinase 1
2z7l.1.A	24.30	monomer	-	HHblits	X-ray	2.41Å	0.32	0.38	Mitogen-activated protein kinase 1
6cpw.1.A	24.30	monomer	-	HHblits	X-ray	1.85Å	0.32	0.38	Mitogen-activated protein kinase 1
3fi2.1.A	26.95	monomer	-	HHblits	X-ray	2.28Å	0.33	0.38	Mitogen-activated protein kinase 10
4y5h.1.A	25.89	monomer	-	HHblits	X-ray	2.06Å	0.32	0.38	Mitogen-activated protein kinase 10
3fv8.1.A	27.05	monomer	-	HHblits	X-ray	2.28Å	0.33	0.38	Mitogen-activated protein kinase 10
4w4y.1.A	25.89	monomer	-	HHblits	X-ray	2.30Å	0.32	0.38	c-jun NH2-terminal kinase 3
3ptg.1.A	25.89	monomer	-	HHblits	X-ray	2.43Å	0.32	0.38	Mitogen-activated protein kinase 10
3oy1.1.A	26.69	monomer	-	HHblits	X-ray	1.70Å	0.32	0.38	Mitogen-activated protein kinase 10
3oxi.1.A	26.69	monomer	-	HHblits	X-ray	2.20Å	0.32	0.38	Mitogen-activated protein kinase 10
3rtp.1.A	25.89	monomer	-	HHblits	X-ray	2.40Å	0.32	0.38	Mitogen-activated protein kinase 10

The table above shows the top 50 filtered templates. A further 5,495 templates were found which were considered to be less suitable for modelling than the filtered list.
1a06.1.A, 1a9u.1.A, 1ad5.1.A, 1apm.1.A, 1aq1.1.A, 1b39.1.A, 1b6c.5.D, 1bi7.1.A, 1bi8.1.A, 1bkx.1.A, 1bl7.1.A, 1blx.1.A, 1bmk.1.A, 1buh.1.A, 1bx6.1.A, 1byg.1.A, 1cdk.1.A, 1ckj.1.A, 1ckj.1.B, 1cm8.1.A, 1cmk.1.A, 1csn.1.A, 1ctp.1.A, 1di8.1.A, 1di9.1.A, 1dow.1.A, 1ds5.1.A, 1e9h.1.A, 1eh4.1.A, 1eh4.2.A, 1f0q.1.A, 1f3m.1.D, 1f5q.1.A, 1f5q.2.A, 1fgi.1.A, 1fin.1.A, 1fin.2.A, 1fmk.1.A, 1fot.1.A, 1fpu.1.A, 1fq1.1.B, 1fvr.1.A, 1fvv.1.A, 1g3n.4.C, 1g5s.1.A, 1gih.1.A, 1gij.1.A, 1gjo.1.A, 1gng.1.A, 1gol.1.A, 1gzk.1.A, 1gzn.1.A, 1h01.1.A, 1h1p.2.A, 1h1q.1.A, 1h1q.2.A, 1h1w.1.A, 1h25.2.A, 1h27.1.A, 1h28.2.A, 1h4l.1.A, 1h8f.1.A, 1hcl.1.A, 1how.1.A, 1i09.1.A, 1i44.1.A, 1ia8.1.A, 1ias.1.A, 1ias.1.B, 1ir3.1.C, 1irk.1.A, 1j1b.1.A, 1j1b.1.B, 1j3h.1.A, 1j7i.1.A, 1j7u.1.A, 1j7u.1.B, 1j91.1.A, 1j91.2.A, 1jbp.1.A, 1jks.1.A, 1jkt.1.A, 1jkt.2.A, 1jnk.1.A, 1jow.1.B, 1jpa.1.A, 1jqh.2.A, 1jst.1.A, 1jst.1.C, 1jsu.1.A, 1jvp.1.A, 1jwh.1.A, 1jwh.1.B, 1k2p.1.A, 1k2p.1.B, 1k3a.1.A, 1k9a.1.A, 1k9a.2.A, 1k9a.3.A, 1k9a.5.A, 1k9a.6.A, 1koa.1.A, 1ksw.1.A, 1kv1.1.A, 1kwp.3.B, 1kwp.3.C, 1l8t.1.A, 1lew.1.A, 1lp4.1.A, 1lq7.1.A, 1luf.1.A, 1m14.1.A, 1m17.1.A, 1m2p.1.A, 1m2q.1.A, 1m2r.1.A, 1m52.1.A, 1m7n.1.A, 1mp8.1.A, 1mq4.1.A, 1mqb.1.A, 1mqb.2.A, 1mru.1.A, 1mru.2.A, 1mrv.1.A, 1muo.1.A, 1na7.1.A, 1nd4.1.A, 1nw1.1.A, 1nxk.1.A, 1ny3.2.C, 1o6k.1.A, 1o6l.1.A, 1o6y.1.A, 1o9u.1.A, 1oit.1.A, 1okv.2.A, 1oky.1.A, 1ol2.1.A, 1ol5.1.A, 1ol6.1.A, 1ol7.1.A, 1omw.1.A, 1opk.1.A, 1opl.1.A, 1opl.2.A, 1ouk.1.A, 1oz1.1.A, 1p14.1.A, 1p2a.1.A, 1p4f.1.A, 1p4o.1.A, 1p5e.1.A, 1pf8.1.A, 1phk.1.A, 1pjk.1.A, 1pkg.1.A, 1pkg.2.A, 1pme.1.A, 1pmn.1.A, 1pmu.1.A, 1pmv.1.A, 1pxo.1.A, 1py5.1.A, 1pyx.1.A, 1pyx.1.B, 1q24.1.A, 1q5k.1.A, 1q5k.1.B, 1q8w.1.A, 1q8y.1.A, 1q8y.2.A, 1qcf.1.A, 1ql6.1.A, 1qmz.1.A, 1qpd.1.A, 1qpe.1.A, 1r0e.1.A, 1r0p.1.A, 1r39.1.A, 1r3c.1.A, 1r78.1.A, 1rdq.1.A, 1re8.1.A, 1rek.1.A, 1rjb.1.A, 1rqq.1.A, 1rw8.1.A, 1s35.1.A, 1s9i.1.A, 1s9i.1.B, 1s9j.1.A, 1sm2.1.A, 1sm2.2.A, 1smh.1.A, 1snu.2.A, 1stc.1.A, 1syk.1.A, 1szm.1.A, 1szm.2.A, 1t45.1.A, 1t46.1.A, 1tki.1.A, 1tqi.1.A, 1tvo.1.A, 1u46.1.A, 1u46.2.A, 1u54.1.A, 1u54.2.A, 1u59.1.A, 1u5q.1.A, 1u5q.2.A, 1u5r.2.A, 1u5r.3.B, 1ua2.1.A, 1ua2.2.A, 1ua2.3.A, 1ua2.4.A, 1ukh.1.A, 1uki.1.A, 1ung.2.A, 1unh.1.A, 1uu7.1.A, 1uu9.1.A, 1uv5.1.A, 1uwh.1.A, 1uwj.1.A, 1v0b.1.A, 1v0o.1.A, 1v0o.2.A, 1v0p.1.A, 1v0p.2.A, 1v1k.1.A, 1vjy.1.A, 1vr2.1.A, 1vyw.1.A, 1vyw.2.A, 1vzo.1.A, 1w7h.1.A, 1w82.1.A, 1w98.1.A, 1wak.1.A, 1wbo.1.A, 1wbv.1.A, 1wbw.1.A, 1wmk.1.A, 1wvw.1.A, 1wvx.1.A, 1wvy.1.A, 1wzy.1.A, 1x8b.1.A, 1xh4.1.A, 1xh6.1.A, 1xh9.1.A, 1xjd.1.A, 1xkk.1.A, 1xr1.1.A, 1xws.1.A, 1y57.1.A, 1y6a.1.A, 1y8g.1.A, 1ydt.1.A, 1yhv.1.A, 1yhw.1.A, 1yi3.1.A, 1yi4.1.A, 1yi6.1.A, 1yi6.2.A, 1ym7.2.A, 1yoj.1.A, 1yol.1.A, 1yol.2.A, 1yom.2.A, 1yqj.1.A, 1yrp.1.A, 1yvj.1.A, 1yw2.1.A, 1ywn.1.A,

1ywr.1.A, 1yxs.1.A, 1yxu.1.A, 1yzm.1.A, 1z0j.1.B, 1z0k.1.B, 1z0k.2.B, 1z9x.1.A, 1zlt.1.A, 1zmu.1.A, 1zmv.1.A, 1zoe.1.A, 1zog.1.A, 1zp9.1.A, 1zp9.3.A, 1zp9.4.A, 1zrz.1.A, 1ztf.1.A, 1zws.1.A, 1zxe.1.A, 1zxe.1.B, 1zxe.3.A, 1zxe.3.B, 1zy5.1.A, 1zy5.1.B, 1zyc.1.A, 1zyc.1.B, 1zyc.2.A, 1zyc.2.B, 1zyd.1.A, 1zyj.1.A, 1zyl.1.A, 1zys.1.A, 1zz2.1.A, 2a0c.1.A, 2a19.1.B, 2a19.1.C, 2a1a.1.B, 2a27.1.A, 2a2a.1.A, 2ac3.1.A, 2ac5.1.A, 2acx.1.A, 2auh.1.A, 2ayp.1.A, 2b0q.1.A, 2b1p.1.A, 2b4s.1.B, 2b52.1.A, 2b53.1.A, 2b54.1.A, 2b7a.1.A, 2b7a.2.A, 2b9f.1.A, 2b9h.1.A, 2baj.1.A, 2bak.1.A, 2bal.1.A, 2baq.1.A, 2bcj.1.A, 2bdf.1.A, 2bdw.1.A, 2bdw.1.B, 2bfy.1.A, 2bfy.2.A, 2biy.1.A, 2bkk.1.A, 2bkk.2.A, 2bmc.1.A, 2bts.1.A, 2bva.1.A, 2bva.2.A, 2c1b.1.A, 2c30.1.A, 2c3j.1.A, 2c47.1.A, 2c6d.1.A, 2c6e.1.A, 2c6e.2.A, 2cch.1.A, 2cdz.1.A, 2cgw.1.A, 2chl.1.A, 2cjm.2.A, 2cko.1.A, 2ckp.1.A, 2ckp.1.B, 2ckq.1.A, 2clq.1.A, 2cmw.1.A, 2cn5.1.A, 2cn8.1.B, 2cpk.1.A, 2csn.1.A, 2d3e.1.A, 2d3e.1.B, 2dq7.1.A, 2dwb.1.A, 2dyl.1.A, 2dzm.1.A, 2e2b.1.A, 2e9p.1.A, 2e9v.1.A, 2eb2.1.A, 2eb3.1.A, 2efr.1.A, 2efr.1.B, 2efs.1.A, 2efs.1.B, 2erz.1.A, 2etm.1.A, 2etm.2.A, 2eu9.1.A, 2euf.1.B, 2eva.1.A, 2ewa.1.A, 2exc.1.A, 2exe.1.A, 2exm.1.A, 2f2c.1.B, 2f2u.1.A, 2f2u.2.A, 2f49.3.A, 2f4j.1.A, 2f57.1.A, 2f57.2.A, 2fb8.1.A, 2fh9.1.A, 2fo0.1.A, 2fsl.1.A, 2fso.1.A, 2fst.1.A, 2fum.1.A, 2fum.3.A, 2fvd.1.A, 2fys.1.A, 2fys.2.A, 2g01.1.A, 2g01.2.A, 2g15.1.A, 2g1t.4.A, 2g2f.1.A, 2g2f.2.A, 2g2h.1.A, 2g2h.2.A, 2g2i.1.A, 2g2i.2.A, 2gcd.1.A, 2gcd.2.A, 2gdo.1.A, 2gfc.1.A, 2gfs.1.A, 2ghl.1.A, 2ghm.1.A, 2gmx.1.A, 2gmx.2.A, 2gnf.1.A, 2gnj.1.A, 2gph.1.A, 2gqg.1.A, 2gqg.2.A, 2gs6.1.C, 2gs7.1.A, 2gsf.1.A, 2gtm.1.A, 2gu8.1.A, 2h34.1.A, 2h34.2.A, 2h6d.1.A, 2h8h.1.A, 2h96.1.A, 2h96.2.A, 2h9v.1.A, 2hak.1.A, 2hak.2.A, 2hak.3.A, 2hak.6.A, 2hak.7.A, 2hck.1.B, 2hel.1.A, 2hen.1.A, 2hiw.1.A, 2hiw.2.A, 2hk5.1.A, 2hog.1.A, 2hw6.1.A, 2hw6.2.A, 2hw7.1.A, 2hwo.1.A, 2hwp.2.A, 2hxq.1.A, 2hyy.3.A, 2hz0.1.A, 2hz0.2.A, 2hz4.1.A, 2hz4.2.A, 2hz4.3.A, 2hz8.1.A, 2hzi.1.A, 2hzi.2.A, 2hzn.1.A, 2i0e.1.A, 2i0v.1.A, 2i0y.1.A, 2i1m.1.A, 2i40.2.A, 2i7q.1.A, 2ig7.1.A, 2ijm.1.A, 2in6.1.A, 2itn.1.A, 2itp.1.A, 2itx.1.A, 2itz.1.A, 2ivu.1.A, 2ivv.1.A, 2iw6.1.A, 2iw8.1.A, 2iwi.1.A, 2izs.1.A, 2j0i.1.A, 2j0j.1.A, 2j0k.1.A, 2j0k.2.A, 2j0l.1.A, 2j0m.1.B, 2j2i.1.A, 2j4z.1.A, 2j4z.2.A, 2j50.1.A, 2j5e.1.A, 2j5f.1.A, 2j90.1.A, 2j90.1.B, 2j9m.1.A, 2jam.1.A, 2jav.1.A, 2jbo.1.A, 2jbp.1.A, 2jbp.10.A, 2jc6.1.A, 2jed.1.A, 2jed.2.A, 2jfm.1.B, 2jgz.1.A, 2jii.1.A, 2jit.1.A, 2jit.2.A, 2jiu.1.A, 2jiu.2.A, 2jiv.1.A, 2jiv.2.A, 2jkm.1.A, 2jko.1.A, 2jkq.1.A, 2kty.1.A, 2kul.1.A, 2lav.1.A, 2lgc.1.A, 2lxy.1.A, 2mi7.1.A, 2no3.2.A, 2np8.1.A, 2npq.1.A, 2nru.1.A, 2nru.2.A, 2nru.3.A, 2nru.4.A, 2nry.1.A, 2nry.2.A, 2nry.3.A, 2nry.4.A, 2o5k.1.A, 2o8y.1.A, 2ofu.1.A, 2ofv.1.A, 2ofv.1.B, 2og8.1.A, 2ogv.1.A, 2oh4.1.A, 2oib.1.A, 2oib.2.A, 2oib.3.A, 2oic.2.A, 2oic.3.A, 2oid.1.A, 2oid.2.A, 2oid.3.A, 2oid.4.A, 2oiq.1.A, 2oj9.1.A, 2ojf.1.A, 2ojg.1.A, 2ojj.1.A, 2ok1.1.A, 2onl.1.B, 2onl.2.A, 2onl.2.B, 2ow3.1.A, 2owb.2.B, 2oxy.1.A, 2oza.1.A, 2oza.1.B, 2ozo.1.A, 2p2h.1.A, 2p2i.1.A, 2p3g.1.A, 2p4i.1.A, 2p4i.2.A, 2p55.1.B, 2pe2.1.A, 2phk.1.A, 2pih.1.A, 2pk9.2.A, 2pl0.1.A, 2pml.1.A, 2pmo.1.A, 2ppq.1.A, 2psq.1.A, 2ptk.1.A, 2pup.1.A, 2puu.1.A, 2pvf.1.A, 2pvj.1.A, 2pvy.1.B, 2pwl.1.A, 2py3.1.A, 2pyw.1.A, 2pz5.1.A, 2pzi.1.A, 2pzi.2.A, 2pzp.1.A, 2pzr.1.A, 2pzy.1.A, 2pzy.2.A, 2pzy.3.A, 2pzy.4.A, 2q0b.1.A, 2q0n.1.A, 2q83.1.A, 2qd9.1.A, 2qg5.1.A, 2qg5.2.A, 2qg7.1.A, 2qg7.1.B, 2qg7.2.A, 2qg7.2.B, 2qhm.1.A, 2qi8.1.A, 2qkr.1.A, 2qkw.1.B, 2qlq.1.A, 2qlq.2.A, 2qlu.1.A, 2qnj.1.A, 2qnj.1.B, 2qoc.1.A, 2qod.1.A, 2qof.1.A, 2qoh.1.A, 2qoi.1.A, 2qok.1.A, 2qol.1.A, 2qon.1.A, 2qoo.1.A, 2qu5.1.A, 2qu6.1.A, 2qur.1.A, 2qvs.1.A, 2r0i.1.A, 2r2p.1.A, 2r3n.1.A, 2r4b.1.A, 2r64.1.A, 2r7i.1.A, 2r9s.1.A, 2rei.1.A, 2rf9.1.A, 2rf9.2.A, 2rfd.1.A, 2rfd.2.A, 2rfe.2.A, 2rfe.4.A, 2rfn.1.A, 2rfs.1.A, 2rg5.1.A, 2rg6.1.A, 2rgp.1.A, 2rio.1.A, 2rku.1.A, 2rl5.1.A, 2rsv.1.A, 2src.1.A, 2uzb.2.A, 2uzd.1.A, 2uzl.1.A, 2uzv.1.B, 2v55.1.A, 2v5q.1.A, 2v5q.2.A, 2v62.1.A, 2v7a.1.A, 2vd5.1.A, 2vn9.1.A, 2vo6.1.A, 2vrx.1.A, 2vrx.2.A, 2vth.1.A, 2vtn.1.A, 2vtt.1.A, 2vuw.1.A, 2vwb.1.A, 2vwb.2.A, 2vwi.1.A, 2vwi.1.B, 2vwi.1.C, 2vwi.1.D, 2vwx.1.A, 2vx3.4.A, 2vz6.1.A, 2w1g.1.A, 2w1i.1.A, 2w1z.1.A, 2w4j.1.A, 2w4k.1.A, 2w4o.1.A, 2w5a.1.A, 2w5h.1.A, 2w7x.1.B, 2w96.1.B, 2w99.1.B, 2w9f.1.B, 2w9z.1.B, 2wb8.1.A, 2wd1.1.A, 2wei.1.A, 2wel.1.A, 2wma.1.A, 2wma.2.A, 2wmb.2.A, 2wnt.1.A, 2wo6.2.A, 2wot.1.A, 2wqb.1.A, 2wqe.1.A, 2wqn.1.A, 2wqo.1.A, 2wtk.1.B, 2wtk.1.C, 2wtk.2.C, 2wtv.1.A, 2wtv.2.A, 2wtw.1.A, 2wu6.1.A, 2wzj.1.A, 2x0g.1.A, 2x39.1.A, 2x4f.1.A, 2x4z.1.A, 2x6e.1.A, 2x7f.1.A, 2x7o.3.A, 2x8e.1.A, 2x8i.1.A, 2x9e.1.A, 2xa4.1.A, 2xba.1.A, 2xck.1.A, 2xik.1.A, 2xir.1.A, 2xj0.1.A, 2xk9.1.A, 2xm8.1.B, 2xne.1.A, 2xng.1.A, 2xnm.1.A, 2xru.1.A, 2xrw.1.A, 2xs0.1.A, 2xuu.1.A, 2xyn.3.A, 2xyu.1.A, 2xzs.1.A, 2y0a.1.A, 2y4i.1.A, 2y4i.1.B, 2y4p.1.B, 2y4p.2.A, 2y6m.1.A, 2y7j.1.A, 2y7j.1.B, 2y8o.1.A, 2ya9.1.A, 2yak.1.A, 2ycf.1.A, 2ycr.1.A, 2yex.1.A, 2yfx.1.A, 2yhv.1.A, 2yiw.1.A, 2yix.1.A, 2yiy.1.A, 2yjr.1.A, 2yjs.1.A, 2yn8.1.A, 2ywp.1.A, 2yza.1.A, 2z2w.2.A, 2z60.1.A, 2z7q.1.A, 2z8c.1.A, 2zb1.1.A, 2zdt.1.A, 2zdu.1.A, 2zjw.1.A, 2zm1.1.A, 2zm3.2.A, 2zm3.3.A, 2zmc.1.A, 2zmd.1.A, 2zoq.1.A, 2zoq.2.A, 2zv7.1.A, 3a2c.1.D, 3a2c.1.F, 3a2c.1.G, 3a2c.1.H, 3a2c.1.I, 3a2c.1.L, 3a4o.1.A, 3a4p.1.A, 3a60.1.A, 3a60.1.B, 3a61.1.A, 3a62.1.A, 3a7f.1.A, 3a7g.1.A, 3a7g.2.A, 3a7i.1.A, 3a8w.1.A, 3a8x.1.A, 3a99.1.A, 3ag9.1.A, 3ag9.3.B, 3agl.1.A, 3aln.1.A, 3aln.2.A, 3aln.3.A, 3alo.1.A, 3ama.1.A, 3amy.1.A, 3anr.1.A, 3aox.1.A, 3aqv.1.A, 3at4.1.A, 3ats.1.A, 3axw.1.A, 3b2t.1.A, 3b2w.1.A, 3b8r.1.A, 3b8r.2.A, 3bbt.1.A, 3bce.1.A, 3be2.1.A, 3bea.1.A, 3beg.1.A, 3bgq.1.A, 3bhh.1.A, 3bhh.3.A, 3bht.1.A, 3bht.2.A, 3bhy.1.A, 3bi6.1.A, 3bkb.1.A, 3blq.1.A, 3blr.1.A, 3bpr.4.A, 3bqr.1.B, 3brb.1.A, 3brb.2.A, 3brt.1.A, 3brt.1.C, 3brv.1.A, 3brv.1.C, 3bu3.1.A, 3bv2.1.A, 3bv3.1.A, 3bx5.1.A, 3bym.1.A, 3bys.1.A, 3byv.1.A, 3bz3.1.A, 3c0g.1.A, 3c0i.1.A, 3c1x.1.A, 3c4c.1.A, 3c4c.2.A, 3c4d.1.A, 3c4d.2.A, 3c4e.1.A, 3c4f.1.A, 3c4x.1.A, 3c4y.1.A, 3c4y.1.B, 3c4z.1.A, 3c50.1.B, 3c51.1.A, 3c51.1.B, 3c5i.1.A, 3c5u.1.A, 3c7q.1.A, 3c9w.1.A, 3cbl.1.A, 3cc6.1.A, 3ce3.1.A, 3cik.1.A, 3cjf.1.A, 3cjg.1.A, 3ckw.1.A, 3cly.1.A, 3coh.1.A, 3coi.1.A, 3cok.1.A, 3com.1.A, 3com.2.A, 3cp9.2.A, 3cqu.1.A, 3cs9.1.A, 3cs9.4.A, 3csv.1.A, 3cth.1.A, 3ctj.1.A, 3ctq.1.A, 3cxw.1.A, 3d0e.1.A, 3d14.1.A, 3d2k.1.A, 3d5u.1.A, 3d5v.1.A, 3d5x.1.A, 3d7t.1.A, 3d7t.2.B, 3d7u.1.A, 3d7u.1.B, 3d94.1.A, 3d9v.1.A, 3d9v.1.B, 3da6.1.A, 3dae.1.A, 3daj.1.A, 3dak.1.A, 3dak.1.C, 3dbq.1.A, 3dcv.1.A, 3ddp.1.A, 3ddq.1.A, 3dfa.1.A, 3dfc.1.A, 3dj5.1.A, 3dk3.1.A, 3dk3.2.A, 3dk6.1.A, 3dk6.2.A, 3dk7.1.A, 3dk7.2.A, 3dkc.1.A, 3dkf.1.A, 3dkg.1.A, 3dko.1.A, 3dls.1.A, 3dlz.1.A, 3dnd.1.A, 3dqw.1.A, 3dqw.3.A, 3dqx.1.A, 3dqx.2.A, 3ds6.1.A, 3ds6.2.A, 3ds6.3.A, 3ds6.4.A, 3dt1.1.A, 3dtc.1.A, 3du8.2.A, 3dv3.1.A, 3dxn.1.A, 3dxp.1.A, 3dxq.1.A, 3dy7.1.A, 3dzo.1.A, 3dzq.1.A, 3e3b.2.B, 3e3p.1.A, 3e5a.1.A, 3e64.1.A, 3e7o.1.A, 3e7o.1.B, 3e8c.2.A, 3e92.1.A, 3e93.1.A, 3eb0.1.A, 3efl.2.A, 3ej1.1.A, 3ekk.1.A, 3el7.1.A, 3el8.1.A, 3emg.1.A, 3en7.1.A, 3en9.1.A, 3en9.2.A, 3enh.1.A, 3enm.1.A, 3enm.1.B, 3enm.2.A, 3eqc.1.A, 3eqp.1.A, 3eqr.1.A, 3erk.1.A, 3eta.1.A, 3eyg.1.A, 3ezv.1.A, 3f2a.1.A, 3f3w.1.A, 3f3z.1.A, 3f5p.17.F, 3f5u.1.A, 3f61.1.A, 3f66.1.A, 3f66.2.A, 3f69.1.A, 3f6x.3.A, 3f7w.1.A, 3f7z.1.A, 3f82.1.A, 3f88.1.A, 3faa.1.A, 3faa.3.A, 3fc1.1.A, 3fe3.1.A, 3fe3.1.B, 3feg.1.A, 3fhi.1.A, 3fhr.1.A, 3fi3.1.A, 3fi8.1.A, 3fko.1.A, 3fl4.1.A, 3fln.1.A, 3fls.1.A, 3fme.1.A, 3fpm.1.A, 3fsk.1.A, 3fwq.1.A, 3fwq.2.A, 3fy0.1.A, 3fy2.1.A, 3fzo.1.A, 3fzr.1.A, 3fzt.1.A, 3g0e.1.A, 3g0f.1.A, 3g0f.1.B, 3g2f.1.A, 3g2f.2.A, 3g33.1.A, 3g33.1.C, 3g51.1.A, 3g6h.1.A, 3g6h.2.A, 3g90.1.A, 3gb2.1.A, 3gbz.1.A, 3gc7.1.A, 3gc8.1.A, 3gc8.2.A, 3gc9.1.A, 3gc9.2.A, 3gcp.1.A, 3gcq.1.A, 3gcu.1.B, 3gcv.1.A, 3geq.1.A, 3gfe.1.A, 3ggf.2.A, 3gi3.1.A, 3gni.1.B, 3gok.1.A, 3gop.1.A, 3gp0.1.A, 3gqi.1.A, 3gql.1.A, 3gql.2.A, 3gql.3.A, 3gt8.1.A, 3gt8.1.B, 3gt8.2.A, 3gt8.2.B, 3gu4.1.A, 3gu8.1.A, 3gub.1.A, 3gvu.2.A, 3h0y.2.A, 3h0z.1.A, 3h0z.2.A, 3h0z.3.A, 3h10.1.A, 3h10.3.A, 3h30.1.A, 3h3c.1.A, 3h4j.1.A, 3h4j.2.A, 3h9o.1.A, 3ha6.1.A, 3ha8.1.A, 3ham.1.A, 3ham.1.B, 3hav.1.A, 3hav.2.A, 3hav.3.A, 3hdn.1.A, 3hec.1.A, 3heg.1.A, 3hgk.1.A, 3hko.1.A, 3hll.1.A, 3hmi.1.A, 3hmn.1.A, 3hng.1.A,

3hnw.1.A, 3hnw.1.B, 3hp2.1.A, 3hub.1.A, 3huc.1.A, 3hv4.1.A, 3hv6.1.A, 3hvc.1.A, 3hx4.1.A, 3hyh.1.A, 3hyh.2.A, 3hzt.1.A, 3i0o.1.A, 3i0q.1.A, 3i1a.1.A, 3i1a.2.A, 3i4b.1.A, 3i4b.1.B, 3i5z.1.A, 3i6u.1.A, 3i6u.1.B, 3i6w.1.A, 3i6w.1.B, 3i6w.2.A, 3i6w.2.B, 3i6w.3.A, 3i6w.3.B, 3i79.1.A, 3i7b.1.A, 3i81.1.A, 3idb.1.A, 3idp.1.A, 3iec.1.A, 3igo.1.A, 3ii5.1.A, 3ik3.1.A, 3ik3.2.A, 3ika.1.A, 3ika.3.B, 3iph.1.A, 3is5.1.A, 3is5.1.F, 3iw4.1.A, 3iw4.2.A, 3iw6.1.A, 3j4r.1.D, 3jr1.1.A, 3js2.2.A, 3juh.1.A, 3jvr.1.A, 3jy0.1.A, 3jy9.1.A, 3jya.1.A, 3k3i.1.A, 3k3j.1.A, 3k54.1.A, 3k5u.1.A, 3ka0.1.A, 3kb7.1.A, 3kc3.1.A, 3kc3.1.B, 3kc3.1.C, 3kc3.2.A, 3kc3.2.C, 3kc3.3.B, 3kc3.3.C, 3kc3.4.A, 3kc3.4.B, 3kc3.4.C, 3kcf.3.A, 3kcf.4.A, 3kcf.5.A, 3kck.1.A, 3kex.1.A, 3kf4.2.A, 3kfa.2.A, 3kk8.1.A, 3kk9.1.A, 3kl8.1.A, 3kmm.1.A, 3kmu.1.A, 3kn5.1.A, 3kn5.2.A, 3kq7.1.A, 3krj.1.A, 3krl.1.A, 3krr.1.A, 3krw.1.A, 3ku2.1.A, 3kul.1.A, 3kvx.1.A, 3kxn.1.A, 3kxx.1.A, 3kxx.3.A, 3kxx.4.A, 3kxz.1.A, 3ky2.1.A, 3ky2.2.A, 3l39.1.A, 3l8p.1.A, 3l8s.1.A, 3l9l.1.A, 3l9m.1.A, 3l9p.1.A, 3lcd.1.A, 3lck.1.A, 3lco.1.A, 3lct.1.A, 3lfb.1.A, 3lfd.1.A, 3lff.1.A, 3lfs.1.A, 3lij.1.A, 3lj1.1.B, 3llt.1.A, 3lmg.1.A, 3lok.1.A, 3lpb.1.A, 3lq3.1.B, 3lq5.1.A, 3lq8.1.A, 3lvp.1.A, 3lvp.2.A, 3lvp.3.A, 3lvp.4.A, 3lw0.1.A, 3lxk.1.A, 3lxn.1.A, 3lxp.1.A, 3lzb.1.A, 3m1s.1.B, 3m2w.1.A, 3ma6.1.A, 3ma6.2.A, 3mbl.1.A, 3mdy.1.A, 3mes.1.A, 3mfr.1.A, 3mh0.1.A, 3mh1.1.A, 3mh2.1.A, 3mh3.1.A, 3mi9.1.A, 3mj1.1.A, 3mn3.1.A, 3mpa.1.A, 3mpm.1.A, 3mpt.1.A, 3ms9.1.A, 3mtl.2.B, 3mv5.1.A, 3mvj.1.A, 3mvj.2.A, 3mvj.3.A, 3mvl.1.A, 3mvl.2.A, 3mvm.1.A, 3mvm.2.A, 3mw1.1.A, 3my0.1.A, 3myg.1.A, 3n4t.1.A, 3n4u.1.A, 3n4v.1.A, 3n9x.1.A, 3nax.1.A, 3nay.1.A, 3ncg.1.A, 3ncz.1.A, 3ncz.1.B, 3ncz.2.A, 3ncz.2.B, 3ndm.1.A, 3new.1.A, 3nga.1.A, 3nie.1.A, 3niz.1.A, 3nlb.1.A, 3nnu.1.A, 3nnx.1.A, 3npc.1.A, 3nr9.1.B, 3nrm.1.A, 3nsz.1.A, 3nun.1.A, 3nus.1.A, 3nw5.1.A, 3nw6.1.A, 3nw7.1.A, 3nx8.1.A, 3nyn.1.A, 3nyx.1.A, 3nz0.1.A, 3o0g.1.A, 3o0g.2.A, 3o17.1.A, 3o17.2.A, 3o23.1.A, 3o2m.1.A, 3o50.1.A, 3o50.2.A, 3o51.1.A, 3o71.1.A, 3o7l.1.A, 3o7l.1.B, 3o8p.1.A, 3o8t.1.A, 3o8u.1.A, 3o96.1.A, 3obg.1.A, 3obj.1.A, 3oc1.1.A, 3ocb.1.A, 3ocb.2.A, 3ocg.1.A, 3ocs.1.A, 3oct.1.A, 3od6.1.A, 3ody.1.A, 3odz.1.A, 3oef.1.A, 3oez.1.A, 3oez.2.A, 3of0.1.A, 3ofm.1.A, 3og7.1.A, 3og7.2.A, 3oht.1.A, 3omv.1.A, 3oog.1.A, 3op5.2.A, 3op5.3.A, 3ori.1.A, 3ork.1.A, 3orl.1.A, 3orm.1.A, 3orn.2.A, 3oro.1.A, 3orx.1.A, 3orx.5.A, 3orz.1.A, 3os3.1.A, 3otu.1.A, 3otv.1.A, 3otv.3.A, 3otv.4.A, 3ouk.1.A, 3oun.1.B, 3ovv.1.A, 3owj.1.A, 3oxz.1.A, 3oy3.1.A, 3oz6.1.A, 3oz6.1.B, 3p08.3.A, 3p23.1.A, 3p23.1.B, 3p4k.1.A, 3p7a.1.A, 3p7c.1.A, 3p86.1.A, 3p86.2.A, 3p9j.1.A, 3pa4.1.A, 3pe2.1.A, 3pfq.1.A, 3pg1.1.A, 3pix.1.A, 3piy.1.A, 3piz.1.A, 3pj2.1.A, 3pj8.1.A, 3pjc.1.A, 3pls.1.A, 3poz.1.A, 3pp0.1.A, 3pp0.2.A, 3ppj.1.A, 3ppz.1.A, 3psc.1.A, 3pvb.1.A, 3pvg.1.A, 3pwy.1.A, 3pxk.1.B, 3pxk.2.A, 3pxq.1.A, 3pxr.1.A, 3py0.1.A, 3py1.1.A, 3py3.1.A, 3pyy.1.A, 3pyy.2.A, 3pze.1.A, 3q04.1.A, 3q2j.1.A, 3q2j.1.B, 3q2m.2.B, 3q32.1.A, 3q32.2.A, 3q3b.1.A, 3q3b.2.A, 3q4c.1.A, 3q4t.1.A, 3q4t.2.A, 3q4u.1.A, 3q4u.1.B, 3q4u.3.A, 3q4z.1.A, 3q4z.1.B, 3q52.1.A, 3q5i.1.A, 3q5z.1.A, 3q60.1.A, 3q6u.1.A, 3q6w.1.A, 3q96.1.A, 3q9w.1.A, 3q9x.2.A, 3q9z.1.A, 3qa0.1.A, 3qa8.1.A, 3qa8.1.B, 3qa8.1.D, 3qa8.2.B, 3qal.1.A, 3qam.1.A, 3qbn.1.A, 3qc4.1.A, 3qc4.2.A, 3qc9.1.A, 3qd2.1.A, 3qd3.1.A, 3qfv.1.A, 3qgw.1.A, 3qgw.2.A, 3qgy.2.A, 3qhr.1.A, 3qkk.1.A, 3qkl.1.A, 3qqj.1.A, 3qqu.1.A, 3qqu.3.A, 3qrj.1.A, 3qrk.1.A, 3qru.1.A, 3qti.1.A, 3qtr.1.A, 3qtu.1.A, 3qtw.1.A, 3que.1.A, 3qup.1.A, 3qwj.1.A, 3qwk.1.A, 3qxp.1.A, 3qyw.1.A, 3qzg.1.A, 3r21.1.A, 3r22.1.A, 3r2b.1.A, 3r2y.1.A, 3r63.1.A, 3r78.1.A, 3r78.2.A, 3r83.1.A, 3r8m.1.A, 3r8v.1.A, 3r8z.1.A, 3r9d.1.A, 3rai.1.A, 3raw.1.A, 3rcd.1.A, 3rcd.4.A, 3rcj.1.A, 3re4.1.A, 3rgf.1.A, 3rhk.1.A, 3rhx.1.A, 3ri1.1.A, 3rin.1.A, 3rkb.1.A, 3rm6.1.A, 3rni.1.A, 3rny.1.A, 3rp0.1.A, 3rp0.2.A, 3rp9.1.A, 3rps.1.A, 3rps.2.A, 3rvg.1.A, 3rwp.1.A, 3rwq.1.A, 3rzf.1.A, 3s0o.1.A, 3s3i.1.A, 3s4q.1.A, 3say.1.A, 3sd0.1.A, 3sd0.1.B, 3sdj.1.A, 3sdm.1.A, 3sg8.1.A, 3sg9.1.A, 3sgc.1.A, 3she.1.A, 3sls.1.A, 3soa.1.A, 3soc.1.A, 3sqq.1.A, 3srv.1.A, 3sv0.1.A, 3svv.1.A, 3sxr.1.A, 3sxs.1.A, 3t8o.1.A, 3t9i.1.A, 3t9t.1.A, 3tac.1.A, 3tdv.1.A, 3tdw.1.A, 3tei.1.A, 3tg1.1.A, 3thb.1.A, 3ti1.1.A, 3tiy.1.A, 3tjd.1.A, 3tjd.2.A, 3tku.1.A, 3tl8.1.A, 3tl8.2.A, 3tl8.3.A, 3tm0.1.A, 3tni.1.A, 3tnp.2.A, 3tnq.1.A, 3tt0.1.A, 3tt0.2.A, 3ttj.1.A, 3tub.1.A, 3tuc.1.A, 3tud.1.A, 3tv7.1.A, 3tv7.1.B, 3tv7.2.A, 3twj.1.A, 3twj.1.B, 3twj.2.A, 3twj.2.B, 3txo.1.A, 3tyk.1.A, 3tz7.2.A, 3tz9.2.A, 3tzm.1.A, 3u4u.1.A, 3u4w.1.A, 3u51.1.A, 3u6j.1.A, 3u9n.1.A, 3ubd.1.A, 3uc3.1.A, 3uc4.1.A, 3uc4.2.A, 3udb.1.A, 3udb.2.A, 3udb.3.A, 3udb.4.A, 3udb.5.A, 3udb.6.A, 3ue4.1.A, 3ue4.2.A, 3ug1.1.B, 3ugc.1.A, 3uib.1.A, 3uim.1.A, 3uiu.1.A, 3ujg.1.A, 3unj.1.A, 3unk.1.A, 3unz.3.A, 3uo4.1.A, 3uo5.1.A, 3uod.1.A, 3uoj.2.A, 3uok.1.A, 3uol.3.B, 3up2.1.A, 3up7.1.A, 3uqc.1.A, 3uqc.1.B, 3uqc.2.B, 3uqf.1.A, 3uqf.2.A, 3uto.1.A, 3uto.2.A, 3uvp.1.A, 3uvr.1.A, 3uys.2.A, 3uyt.2.A, 3uyt.5.A, 3uyt.5.B, 3uyt.6.A, 3uzp.1.A, 3uzp.2.A, 3uzr.1.A, 3uzs.1.A, 3uzt.1.A, 3v1a.1.A, 3v1b.1.A, 3v1b.1.B, 3v1c.1.A, 3v1c.1.B, 3v1d.1.A, 3v1d.1.B, 3v1e.1.A, 3v1e.1.B, 3v1f.1.A, 3v1f.1.B, 3v3v.1.A, 3v5j.1.A, 3v5q.1.A, 3v5t.1.A, 3v5w.1.A, 3v6s.1.A, 3v6s.2.A, 3v8s.1.A, 3vap.1.A, 3vby.1.A, 3vhe.1.A, 3vhk.1.A, 3vid.1.A, 3vjo.1.A, 3vn9.1.A, 3vnt.1.A, 3vqu.1.A, 3vry.2.A, 3vs0.2.A, 3vs3.2.A, 3vs6.1.A, 3vs7.1.A, 3vs7.2.A, 3vud.1.A, 3vug.1.A, 3vuh.1.A, 3vui.1.A, 3vuk.1.A, 3vul.1.A, 3vum.1.A, 3vut.1.A, 3vut.2.A, 3vvh.1.A, 3vvh.2.A, 3vw6.1.A, 3vw6.1.B, 3vw8.1.A, 3vwa.1.A, 3w0m.1.A, 3w0n.1.A, 3w0p.1.A, 3w0q.1.A, 3w0r.1.A, 3w0s.1.A, 3w10.1.A, 3w16.1.A, 3w18.1.A, 3w18.2.A, 3w1f.1.A, 3w2c.1.A, 3w2o.1.A, 3w2r.1.A, 3w2s.1.A, 3w32.1.A, 3w55.1.A, 3w8l.1.A, 3w8l.2.A, 3w8q.1.A, 3we8.1.A, 3wf5.1.A, 3wf7.1.A, 3wf8.1.A, 3wi6.1.B, 3wik.1.A, 3wil.1.A, 3wow.1.A, 3wyx.1.A, 3wzd.1.A, 3wze.1.A, 3wzu.1.A, 3x2u.1.A, 3zc6.1.A, 3zc6.2.A, 3zdi.1.A, 3zdu.1.A, 3zep.1.A, 3zep.2.A, 3zep.3.A, 3zew.1.A, 3zfm.1.A, 3zfx.1.A, 3zfy.1.A, 3zh8.1.A, 3zhp.1.B, 3zls.1.A, 3zlw.1.A, 3zly.1.A, 3zmm.1.A, 3zon.1.A, 3zos.2.A, 3zrk.1.A, 3zsh.1.A, 3zsi.1.A, 3ztx.1.A, 3ztx.2.A, 3zut.1.A, 3zuu.1.A, 3zuv.1.A, 3zxt.1.A, 3zxt.1.B, 3zxt.2.A, 3zya.1.A, 3zzw.1.A, 3zzw.1.B, 4a4x.1.A, 4a7c.1.A, 4aaa.1.A, 4acc.1.A, 4acd.1.B, 4ach.1.A, 4ad8.1.A, 4ae6.1.A, 4ae6.2.A, 4af3.1.A, 4afe.1.A, 4afj.1.A, 4agc.1.A, 4agu.1.A, 4agw.1.A, 4akv.1.A, 4akv.1.B, 4alu.1.A, 4alv.1.A, 4an2.1.A, 4anl.1.A, 4anm.1.A, 4ans.1.A, 4aoj.1.A, 4aot.1.A, 4aot.2.A, 4ap7.1.A, 4apc.1.A, 4app.1.A, 4aqc.1.A, 4aqc.2.A, 4ark.1.B, 4asd.1.A, 4asz.1.A, 4at3.1.A, 4at4.1.A, 4at5.1.A, 4au8.1.A, 4aw2.1.A, 4aw5.1.A, 4awi.1.A, 4ax8.1.A, 4azf.1.A, 4azt.1.A, 4azw.1.A, 4b0g.1.A, 4b4l.1.A, 4b6l.1.A, 4b7t.1.A, 4b8l.1.A, 4b8m.1.A, 4b8m.2.A, 4b99.1.A, 4bb4.1.A, 4bbe.1.A, 4bbf.3.A, 4bbm.1.A, 4bc6.1.A, 4bcf.1.A, 4bcg.1.A, 4bci.1.A, 4bcj.1.A, 4bck.1.A, 4bck.2.A, 4bcm.1.A, 4bcn.1.A, 4bco.1.A, 4bcq.1.A, 4bcq.2.A, 4bfm.1.A, 4bgq.1.A, 4bib.1.A, 4bib.2.A, 4bkj.2.A, 4bn1.1.A, 4br3.1.B, 4brx.1.A, 4btf.1.A, 4btj.1.A, 4btj.2.A, 4btk.1.A, 4bvu.1.A, 4bwk.1.A, 4bwk.1.B, 4bwp.1.A, 4bwp.1.B, 4bwx.1.A, 4bwx.1.B, 4byi.1.A, 4byj.1.A, 4bzo.1.A, 4c02.1.A, 4c0t.1.A, 4c2v.1.A, 4c2v.2.A, 4c2w.1.A, 4c2w.2.A, 4c33.1.A, 4c34.1.A, 4c35.1.A, 4c3p.1.A, 4c3p.1.C, 4c3r.1.A, 4c57.2.A, 4c8b.1.A, 4cfe.1.A, 4cfh.1.A, 4cfn.1.A, 4cfu.1.A, 4cfu.2.A, 4cfw.1.A, 4cfx.1.A, 4cfx.2.A, 4cg4.1.A, 4cg4.1.B, 4cg4.2.A, 4cg4.2.B, 4cg4.3.A, 4cg4.3.B, 4cgk.1.A, 4cgk.1.B, 4cki.1.A, 4ckr.1.A, 4cnh.2.A, 4cqe.1.A, 4cqe.2.A, 4cqg.1.A, 4crs.1.A, 4csv.1.A, 4ct1.1.A, 4cxa.1.A, 4cxa.2.A, 4cyi.1.A, 4cyi.1.B, 4cyj.1.A, 4cyj.1.B, 4czt.1.A, 4czt.1.B, 4czt.2.A, 4czt.2.B, 4czu.1.A, 4czu.1.B, 4czu.2.A, 4czu.2.B, 4czy.1.B, 4czy.1.D, 4d1s.1.A, 4d28.1.A, 4d28.1.B, 4d28.2.A, 4d28.2.B, 4d2p.1.A, 4d2p.1.B, 4d2p.2.A, 4d2p.2.B, 4d2r.1.A, 4d2s.1.A, 4d2t.2.A, 4d2t.4.A, 4d2w.1.B, 4d4r.1.A, 4d4r.2.A, 4d4v.1.A, 4d4v.2.A, 4d55.1.A, 4d9t.1.A, 4da5.1.A, 4dbn.1.A, 4dbn.1.B, 4dbx.1.A, 4dc2.1.A, 4dca.1.A, 4dce.1.A, 4de4.2.A, 4deb.1.A, 4dee.1.A, 4deg.1.A, 4dfb.1.A, 4dfb.2.A, 4dfl.1.A, 4dfu.1.A, 4dfu.2.A, 4dfy.1.A, 4dfz.1.A, 4dg0.1.A, 4dg2.1.A, 4dg3.1.A, 4dgg.1.A, 4dgl.1.C, 4dgl.1.D, 4dgm.1.A, 4dgn.1.A, 4dh7.1.A, 4dhf.1.A, 4din.1.A, 4dit.1.A, 4dn5.3.A, 4dt9.1.A, 4dta.1.A, 4dtb.1.A, 4dtk.1.A, 4dym.1.A, 4e1z.1.A, 4e20.1.A, 4e26.1.B, 4e3c.1.A, 4e3c.1.B,

4e3c.2.A, 4e3c.2.B, 4e3c.3.A, 4e3c.3.B, 4e4l.1.A, 4e4m.1.A, 4e4x.1.A, 4e5a.1.A, 4e5b.1.A, 4e6d.1.A, 4e6d.2.A, 4e6q.1.A, 4e73.1.A, 4e7w.1.A, 4e93.1.A, 4ebw.1.A, 4ec9.1.A, 4eev.1.A, 4efo.1.A, 4eh2.1.A, 4eh5.1.A, 4eh9.1.A, 4ehg.1.B, 4ehv.1.A, 4ejn.1.A, 4eoi.1.A, 4eoi.2.A, 4eoj.1.A, 4eoj.2.A, 4eok.1.A, 4eok.2.A, 4eom.1.A, 4eom.2.A, 4eon.1.A, 4eon.2.A, 4eoo.1.A, 4eoo.2.A, 4eop.1.A, 4eop.2.A, 4eoq.1.A, 4eoq.2.A, 4eor.1.A, 4eor.2.A, 4eos.1.A, 4eos.2.A, 4eqc.1.A, 4eqm.1.A, 4eqm.4.A, 4equ.1.A, 4erk.1.A, 4erw.1.A, 4eut.1.A, 4eut.1.B, 4euu.1.A, 4ewh.1.A, 4ewq.1.A, 4eyj.1.A, 4eym.1.A, 4ez3.1.A, 4ez5.1.A, 4ez7.1.A, 4f08.2.A, 4f09.1.A, 4f0f.1.A, 4f0g.1.A, 4f0i.1.A, 4f0i.2.A, 4f1m.1.A, 4f1o.1.A, 4f1t.1.A, 4f4p.1.A, 4f65.1.A, 4f6s.1.A, 4f6u.1.A, 4f6w.1.A, 4f7l.1.A, 4f9a.1.A, 4fbx.1.A, 4feu.2.A, 4feu.3.A, 4fev.1.A, 4fev.2.A, 4fev.3.A, 4fev.4.A, 4fev.6.A, 4fex.3.A, 4fex.4.A, 4fex.5.A, 4ff8.1.A, 4fg7.1.A, 4fg8.1.A, 4fg9.1.A, 4fgb.1.A, 4fie.1.A, 4fif.2.A, 4fk3.1.A, 4fk3.1.B, 4fl1.1.A, 4fl2.1.A, 4fl3.1.A, 4fmq.1.A, 4fnw.1.A, 4fnx.1.A, 4fny.1.A, 4fnz.1.A, 4fob.1.A, 4fr4.1.A, 4fr4.4.A, 4fsn.1.A, 4fst.1.A, 4fsu.1.A, 4ft3.1.A, 4fv3.1.A, 4fv7.1.A, 4fv8.1.A, 4fx3.2.A, 4fza.1.B, 4fzd.1.B, 4fzf.1.B, 4g1w.1.A, 4g31.1.A, 4g3c.1.A, 4g3c.2.A, 4g3d.1.A, 4g3d.2.A, 4g3f.1.A, 4g3g.1.A, 4g5j.1.A, 4g5p.1.A, 4g5p.2.A, 4g9c.1.A, 4g9c.2.A, 4g9r.1.A, 4gcj.1.A, 4geo.1.A, 4gfm.1.A, 4gfo.1.A, 4gg5.1.A, 4gg7.1.A, 4gii.1.A, 4gl9.1.A, 4gmy.1.A, 4gs6.1.A, 4gt3.1.A, 4gt4.1.A, 4gt5.1.A, 4gu6.1.B, 4gu6.2.A, 4gub.1.A, 4gv1.1.A, 4gvj.1.A, 4gyg.1.A, 4h05.1.A, 4h05.2.A, 4h1j.1.A, 4h3b.1.A, 4h3p.1.A, 4h3q.1.A, 4h58.1.A, 4h58.2.A, 4h58.3.A, 4hct.1.A, 4hgs.1.A, 4hgt.2.A, 4hjo.1.A, 4hnf.1.A, 4hnf.2.A, 4hni.1.A, 4hok.1.A, 4hok.11.A, 4hok.12.A, 4hok.6.A, 4hok.9.A, 4hvg.1.A, 4hvs.2.B, 4hw7.1.A, 4hyh.1.A, 4hyi.1.A, 4hzr.1.A, 4hzs.1.A, 4i1z.1.A, 4i20.1.A, 4i21.1.A, 4i21.2.A, 4i22.1.A, 4i23.1.A, 4i24.1.A, 4i3z.1.A, 4i41.1.A, 4i4f.1.A, 4i5h.1.A, 4i5m.1.A, 4i6h.1.A, 4i92.1.A, 4i93.1.A, 4i93.2.A, 4i94.1.A, 4i94.2.A, 4iaa.1.A, 4ian.1.B, 4iay.1.A, 4ib5.1.A, 4ib5.2.A, 4ib5.3.A, 4ibm.1.A, 4ibm.2.A, 4ic7.1.A, 4ic7.2.A, 4ic8.1.A, 4id7.1.A, 4idt.1.A, 4idt.1.B, 4idv.1.A, 4ieb.1.A, 4ifc.1.A, 4ifc.2.A, 4ifg.1.A, 4ii5.1.A, 4ijp.1.A, 4im0.1.A, 4im3.1.A, 4imy.1.A, 4imy.3.A, 4iq6.1.A, 4ith.1.A, 4ith.2.A, 4iti.1.A, 4iti.2.A, 4itj.1.A, 4itj.2.A, 4iva.1.A, 4iw0.1.A, 4iwd.1.A, 4iwo.1.A, 4iwp.1.A, 4ix3.1.A, 4ix3.1.B, 4ixp.1.A, 4iz5.1.A, 4iz7.1.A, 4iz7.2.A, 4iza.1.A, 4j1r.2.A, 4j52.1.A, 4j7b.1.A, 4j8m.1.A, 4j8n.3.A, 4j95.5.A, 4j96.1.A, 4j96.2.A, 4j97.1.A, 4j98.1.A, 4j98.2.A, 4j99.1.A, 4jai.1.A, 4jaj.1.A, 4jbo.1.A, 4jbq.1.A, 4jdj.1.A, 4jg6.1.A, 4jg7.1.A, 4jg8.1.A, 4ji9.1.A, 4ji9.2.A, 4jia.1.A, 4jjr.1.A, 4jjr.2.A, 4jl9.1.A, 4jlc.1.B, 4jnw.1.A, 4jq7.1.A, 4jr7.1.A, 4jrn.1.A, 4js8.1.A, 4jt3.1.A, 4jvg.1.A, 4jvg.2.A, 4jx3.1.A, 4jxf.1.A, 4k0y.1.A, 4k11.1.A, 4k18.1.A, 4k1p.1.A, 4k2r.1.A, 4k33.1.A, 4k6z.1.A, 4k9y.1.A, 4kab.1.A, 4kao.1.A, 4kao.2.A, 4kb8.1.A, 4kbc.3.A, 4kbc.3.B, 4kik.1.A, 4kik.1.B, 4kin.1.A, 4kin.4.A, 4kio.2.A, 4kip.1.A, 4kiq.1.A, 4kiq.4.A, 4knb.1.A, 4knb.4.A, 4krc.1.A, 4ks7.1.A, 4ks8.1.A, 4ksp.1.B, 4kwp.1.A, 4l00.1.A, 4l00.2.A, 4l01.1.A, 4l01.2.A, 4l3j.1.A, 4l3p.1.A, 4l42.1.A, 4l43.1.A, 4l44.1.A, 4l45.1.A, 4l46.1.A, 4l52.1.A, 4l67.1.A, 4l68.1.A, 4l68.2.A, 4l6q.1.A, 4l7f.1.A, 4l8m.1.A, 4l9i.1.A, 4lfi.1.B, 4lg4.1.A, 4lg4.2.A, 4lg4.3.A, 4lg4.4.A, 4lg4.5.A, 4lg4.6.A, 4lgd.1.A, 4lgd.2.A, 4lgd.3.A, 4lgd.4.A, 4lgg.1.A, 4lgh.1.A, 4lgh.2.A, 4li5.1.A, 4ll0.1.A, 4loo.1.A, 4lop.1.A, 4lop.3.A, 4loq.4.A, 4lqm.1.A, 4lqp.1.A, 4lqq.1.A, 4lqq.2.A, 4lqs.1.A, 4lrj.1.A, 4lrk.1.A, 4lrk.2.A, 4lrk.3.A, 4lrm.1.A, 4lrm.2.A, 4lrm.3.A, 4lrm.4.A, 4lrm.5.A, 4lv5.2.A, 4lv8.1.A, 4m0y.1.A, 4m66.1.A, 4m67.1.A, 4m68.1.A, 4m69.1.A, 4m69.1.B, 4m8t.1.A, 4m97.1.A, 4mao.1.A, 4mbi.1.A, 4mbj.1.A, 4mcv.1.A, 4md7.1.C, 4md8.1.C, 4md9.1.C, 4mf0.1.B, 4mf1.1.B, 4mh7.2.A, 4mk0.1.A, 4mne.1.B, 4mne.1.C, 4mne.2.A, 4mne.2.D, 4mnf.1.A, 4mnf.1.B, 4mta.1.A, 4mvf.1.A, 4mwi.1.A, 4mxa.1.A, 4mxc.1.A, 4mxo.1.A, 4mxx.1.A, 4mxx.2.A, 4mxy.1.A, 4mxy.2.A, 4myg.1.A, 4myg.2.A, 4n4s.1.A, 4n57.2.A, 4nct.3.A, 4nct.4.A, 4neu.1.A, 4neu.2.A, 4nfm.1.A, 4nfn.1.A, 4nh1.1.A, 4nif.1.A, 4nif.1.D, 4nj3.1.A, 4njd.1.A, 4nm0.1.A, 4nm3.1.A, 4nm5.1.A, 4nst.3.A, 4nt4.1.A, 4nts.1.A, 4nts.2.A, 4ntt.1.A, 4ntt.2.A, 4nus.1.A, 4nw6.1.A, 4nwm.1.A, 4nzw.1.B, 4o0r.1.A, 4o0r.2.A, 4o0s.1.A, 4o0t.1.A, 4o0t.2.A, 4o0u.1.A, 4o0v.1.A, 4o0w.1.A, 4o1o.2.A, 4o21.1.A, 4o27.1.B, 4o2p.1.A, 4o2z.1.A, 4o38.1.A, 4o6e.1.A, 4o6l.2.A, 4o7o.1.A, 4o7o.1.B, 4o7p.1.B, 4o91.2.C, 4o96.1.A, 4o96.2.A, 4oau.1.A, 4oav.1.A, 4oav.1.B, 4obq.1.A, 4obq.1.B, 4ocj.1.A, 4ocu.1.A, 4ogr.1.A, 4oh4.1.A, 4oh4.2.A, 4oli.1.A, 4ons.1.A, 4ons.2.A, 4or5.1.A, 4or5.2.A, 4ork.1.A, 4ork.3.A, 4ork.4.A, 4otd.1.A, 4otf.1.A, 4oth.1.A, 4otp.1.A, 4ow8.1.A, 4p2k.1.A, 4p2w.3.B, 4p4c.1.A, 4p90.1.A, 4p90.2.A, 4p9t.1.A, 4pdo.1.B, 4pdp.1.A, 4pdy.1.A, 4ped.1.A, 4pf4.1.A, 4pl3.1.A, 4pl3.1.B, 4pl4.1.A, 4pl4.1.B, 4pl4.2.A, 4pl4.2.B, 4pl5.1.A, 4pl5.1.B, 4pl5.2.A, 4pl5.2.B, 4pmm.1.A, 4pmt.1.A, 4pni.1.A, 4pnk.1.A, 4pp7.1.A, 4pp9.1.A, 4ppa.3.A, 4ppb.3.B, 4ppc.3.A, 4ppc.3.B, 4pqn.1.A, 4prj.1.A, 4pte.1.A, 4pv0.1.A, 4pwn.1.A, 4px6.1.A, 4py1.1.A, 4q2a.1.A, 4q5e.1.A, 4q5j.1.A, 4q5j.2.A, 4q9z.1.A, 4q9z.1.B, 4qd6.1.A, 4qd6.2.A, 4qfg.1.A, 4qml.1.A, 4qmm.1.A, 4qmn.1.A, 4qmt.1.A, 4qny.1.A, 4qo9.1.A, 4qo9.2.A, 4qox.1.A, 4qp1.1.A, 4qp1.2.A, 4qp2.2.A, 4qp3.1.A, 4qp3.2.A, 4qp4.1.A, 4qp4.2.A, 4qpa.1.A, 4qpm.1.A, 4qps.1.A, 4qps.2.A, 4qq5.1.A, 4qqc.1.A, 4qqj.1.A, 4qqt.1.A, 4qta.1.A, 4qtd.1.A, 4qte.2.B, 4qye.1.A, 4r1v.1.A, 4r1y.1.A, 4r3c.1.A, 4r3p.1.A, 4r3r.1.A, 4r5s.1.A, 4r5y.1.A, 4r77.1.A, 4r78.1.A, 4r7b.1.A, 4r8q.1.A, 4ra4.1.A, 4red.1.A, 4red.1.B, 4rer.1.A, 4rew.1.A, 4rfm.1.A, 4rgj.1.A, 4rio.1.A, 4riw.1.A, 4riw.1.B, 4rix.1.A, 4rix.1.B, 4rix.2.B, 4riy.1.A, 4riy.1.B, 4riy.2.B, 4rlo.1.A, 4rlo.2.A, 4rlp.1.A, 4rmz.1.A, 4rpv.1.A, 4rss.1.A, 4rt7.1.A, 4rvt.1.A, 4rvt.1.B, 4rwi.1.A, 4rwi.2.A, 4rwl.2.A, 4rx5.1.A, 4rx9.1.A, 4rz7.1.B, 4rzv.1.A, 4rzv.2.A, 4rzw.1.A, 4rzw.1.B, 4s2z.1.A, 4s30.1.A, 4s31.1.A, 4s33.1.A, 4tks.1.A, 4tl0.1.A, 4tn6.1.A, 4tn6.2.A, 4tnb.1.A, 4tpt.1.A, 4tt7.1.A, 4tth.1.B, 4tw9.1.A, 4tw9.1.B, 4twn.1.A, 4twp.1.A, 4twp.2.A, 4txc.1.A, 4tye.1.A, 4tyg.1.A, 4tyh.1.A, 4tyh.1.B, 4tyi.1.A, 4tyj.1.A, 4u0i.1.A, 4u3y.1.A, 4u3y.1.B, 4u3z.1.A, 4u3z.1.B, 4u40.2.A, 4u41.2.A, 4u42.1.A, 4u42.1.B, 4u43.1.A, 4u43.3.A, 4u44.2.A, 4u45.2.A, 4u45.3.A, 4u5j.2.A, 4u6r.1.A, 4u79.1.A, 4u7z.1.A, 4u94.1.A, 4u9a.1.A, 4u9a.1.B, 4uak.1.A, 4ub7.1.A, 4uba.1.A, 4uba.2.A, 4ueu.1.A, 4ump.1.A, 4ump.1.B, 4umq.1.A, 4umt.1.A, 4un0.1.B, 4usd.1.A, 4use.2.A, 4usf.1.A, 4usf.2.A, 4uv0.1.A, 4uw0.1.A, 4uwy.1.A, 4uwy.2.A, 4ux9.1.A, 4ux9.4.A, 4uxl.1.A, 4uxq.1.A, 4uy9.1.A, 4uya.1.A, 4uyn.1.A, 4uzd.1.A, 4uzh.1.A, 4v01.2.A, 4v0g.1.A, 4v0g.2.A, 4w7p.1.A, 4w8d.1.A, 4w8e.1.A, 4w9w.1.A, 4wa9.1.A, 4wa9.2.A, 4wb5.1.A, 4wb6.1.A, 4wb7.1.A, 4wb7.2.A, 4wb8.1.A, 4wbb.1.B, 4wbo.1.A, 4wd5.1.A, 4wh1.1.A, 4whz.1.A, 4wih.1.A, 4wnk.1.A, 4wnm.1.A, 4wnp.1.A, 4wnp.2.A, 4wnp.4.A, 4wo5.1.A, 4wo5.2.A, 4wot.1.A, 4wov.1.A, 4wsq.1.A, 4wsq.2.A, 4wsy.1.A, 4wua.1.A, 4wun.1.A, 4ww5.1.A, 4x0m.1.A, 4x3f.1.A, 4x3j.1.A, 4x6r.1.A, 4x7l.1.A, 4x7q.1.A, 4x7q.2.A, 4xbr.1.A, 4xbu.1.A, 4xcu.1.A, 4xey.1.A, 4xey.2.A, 4xg3.2.A, 4xh0.1.A, 4xh6.1.A, 4xhk.1.A, 4xhl.1.A, 4xi2.1.A, 4xli.1.A, 4xlv.1.A, 4xne.1.A, 4xoy.1.A, 4xr7.1.B, 4xr7.1.C, 4xr7.2.A, 4xr7.2.B, 4xr7.4.A, 4xr7.4.B, 4xrl.1.A, 4xuf.1.A, 4xuf.2.A, 4xv1.1.A, 4xv1.1.B, 4xv9.1.A, 4xx9.1.A, 4y0x.1.A, 4y5q.1.A, 4y66.1.B, 4y66.2.B, 4y66.3.B, 4y73.1.A, 4y73.2.A, 4y73.4.A, 4y83.3.A, 4y85.2.A, 4y8d.2.A, 4y93.1.A, 4y95.1.A, 4y95.2.A, 4ybk.1.A, 4yc3.1.A, 4yc6.1.A, 4yc8.1.A, 4yff.1.A, 4yfi.1.A, 4yfi.1.B, 4yga.1.A, 4yga.4.A, 4yhf.1.A, 4yhf.2.A, 4yhj.1.A, 4yhj.1.B, 4yht.1.A, 4yjo.1.A, 4yjq.1.A, 4yjt.1.A, 4yju.1.A, 4ymj.1.A, 4yne.1.A, 4ynz.1.A, 4ynz.1.B, 4yom.1.A, 4yp8.4.A, 4yr8.1.A, 4yr8.3.A, 4ysj.1.A, 4ysm.2.A, 4ytc.1.A, 4yur.1.A, 4yz9.1.A, 4yz9.2.A, 4yz9.3.A, 4yzb.2.A, 4yzc.1.A, 4yzd.1.A, 4yzd.3.A, 4yzm.1.A, 4yzm.2.A, 4yzn.1.A, 4z16.1.A, 4z3v.1.A, 4z55.1.A, 4z7g.1.A, 4z7h.2.A, 4z83.1.A, 4z84.1.A, 4zeg.1.A, 4zhx.1.A, 4zhx.2.A, 4zji.1.A, 4zji.3.A, 4zjv.2.A, 4zlo.1.A, 4zlo.1.B, 4zly.1.A, 4zog.1.A, 4zog.2.A, 4zop.1.B, 4zp5.1.A, 4zp5.2.A, 4zs0.1.A, 4zsa.1.A, 4zse.2.A, 4zsg.1.A, 4zsj.1.A, 4zsl.1.A, 4ztr.1.A, 4zts.1.A, 4zxt.1.A, 4zy4.1.A, 4zy4.2.A, 4zy6.1.A, 4zy6.2.A, 4zzm.1.A, 5a14.1.A, 5a46.1.A, 5a4c.1.A, 5a4e.3.A, 5a6n.1.A, 5a6n.1.B, 5a9u.1.A, 5aa8.1.A, 5aa9.1.A, 5aad.1.A, 5aae.1.A, 5acb.1.B, 5acb.2.B, 5aep.1.A,

5afv.1.B, 5aik.2.A, 5air.1.A, 5air.1.B, 5ajq.1.A, 5ajq.2.A, 5am6.2.A, 5am7.1.A, 5am7.2.A, 5amn.1.A, 5ane.1.A, 5ap0.1.A, 5ap1.1.A, 5ap2.1.A, 5ap3.1.A, 5ap6.1.A, 5ap7.1.A, 5ar2.1.A, 5ar4.1.B, 5ar7.1.A, 5ar7.1.B, 5ar8.1.A, 5av4.1.A, 5awm.1.A, 5ax3.1.A, 5ax9.1.A, 5ax9.2.A, 5ax9.3.A, 5b0x.1.A, 5b2k.1.A, 5b2l.1.A, 5b2m.1.A, 5b7v.2.A, 5bmm.1.A, 5bms.1.A, 5bnj.1.A, 5bpy.1.A, 5bpy.2.A, 5bq0.1.A, 5bue.1.A, 5bvf.1.A, 5bvk.1.A, 5bvo.1.A, 5byl.3.A, 5byl.4.A, 5byy.1.A, 5byz.1.A, 5c01.1.A, 5c03.2.A, 5c4k.2.A, 5c4l.1.A, 5c4l.2.A, 5cas.1.A, 5cav.1.A, 5ce3.1.B, 5cei.1.A, 5cek.1.A, 5cem.1.A, 5cen.1.A, 5ceo.1.A, 5cf5.1.A, 5cf5.2.A, 5ci6.1.A, 5ci6.2.A, 5ckw.1.A, 5ckw.1.B, 5clr.1.A, 5clr.1.B, 5cnn.1.A, 5cno.2.A, 5cqu.1.A, 5cs6.1.A, 5csh.2.A, 5csp.1.A, 5csw.1.A, 5csx.1.A, 5ct7.1.A, 5cu6.1.A, 5cvf.1.A, 5cvg.1.A, 5cws.1.E, 5cws.2.E, 5cwz.1.A, 5cwz.2.A, 5cwz.3.A, 5cxh.1.A, 5cxz.1.A, 5cy3.1.A, 5cyi.1.A, 5cyz.1.A, 5czh.1.A, 5czo.1.A, 5d10.1.A, 5d10.2.A, 5d11.1.A, 5d11.2.A, 5d12.1.A, 5d12.2.A, 5d1j.1.A, 5d41.1.A, 5d41.1.B, 5d7a.1.A, 5d7a.3.A, 5d7v.1.A, 5d9h.1.A, 5d9k.1.A, 5d9l.1.A, 5da3.1.A, 5dbx.1.A, 5dbx.1.B, 5de2.1.A, 5de2.2.A, 5dew.2.A, 5dey.1.A, 5dey.2.A, 5dfp.1.A, 5dfz.1.C, 5dg5.1.B, 5dgz.1.A, 5dh3.1.A, 5di1.1.A, 5di1.2.A, 5dls.1.A, 5dmz.1.A, 5dmz.2.A, 5dnr.1.A, 5dos.1.A, 5drb.1.A, 5dt0.1.A, 5dt4.1.A, 5dvr.1.A, 5dyk.2.A, 5e1e.2.A, 5e1s.1.A, 5e7r.1.A, 5e8u.1.A, 5e8x.1.A, 5eak.1.A, 5ebz.1.F, 5ebz.2.E, 5ebz.2.F, 5ed9.1.A, 5ed9.1.B, 5ed9.1.C, 5edp.1.A, 5edr.1.A, 5efq.1.A, 5eg3.1.A, 5ei6.1.A, 5ek7.1.A, 5eob.1.A, 5eqe.1.A, 5es1.1.A, 5eta.1.A, 5eta.2.A, 5etc.1.A, 5etf.1.A, 5eti.1.A, 5ew3.1.A, 5ew3.2.A, 5ew9.1.A, 5eyk.1.A, 5eym.1.A, 5ezr.1.A, 5ezv.1.A, 5ezv.2.A, 5f1z.1.A, 5f4n.1.A, 5f94.1.A, 5f9e.1.A, 5f9e.2.A, 5fbo.1.A, 5fd2.1.A, 5fd2.1.B, 5fdp.1.A, 5fdx.1.A, 5fee.1.A, 5fg8.1.A, 5fgk.1.A, 5fi4.1.B, 5flf.1.A, 5flf.2.A, 5flf.3.A, 5flf.5.A, 5fm2.1.A, 5fqd.1.C, 5fri.1.A, 5ftg.1.B, 5fwl.1.D, 5fxq.1.A, 5fxr.1.A, 5fxs.1.A, 5g15.1.A, 5g1x.1.A, 5g6v.1.A, 5g6v.2.A, 5ghe.1.A, 5ghv.1.A, 5gjd.1.A, 5gjg.1.A, 5gjr.4.A, 5gmp.1.A, 5gnk.1.A, 5grn.1.A, 5gty.1.B, 5gty.1.C, 5gty.1.D, 5gz8.1.A, 5gza.1.A, 5h09.1.A, 5h0e.1.A, 5h2u.1.A, 5h3q.1.A, 5h8g.1.A, 5hbe.1.A, 5hbh.1.A, 5hd7.1.A, 5he1.1.A, 5he2.1.A, 5he3.1.A, 5hes.1.A, 5hez.1.A, 5hez.2.A, 5hg9.1.A, 5hgi.1.A, 5hhw.1.A, 5hi2.1.A, 5hie.1.A, 5hie.3.A, 5hie.4.A, 5hln.1.A, 5hln.1.B, 5hlp.2.A, 5hlw.1.A, 5hnb.1.A, 5hng.1.A, 5hni.1.A, 5hnv.1.A, 5hoa.1.A, 5hor.1.A, 5hti.1.A, 5hu3.1.A, 5hu9.1.A, 5hvj.1.A, 5hvj.2.A, 5hvk.1.A, 5hvk.2.A, 5hvy.1.A, 5hx6.1.A, 5hx6.2.A, 5hze.1.A, 5hzn.7.A, 5hzn.8.A, 5i35.1.A, 5i3o.1.A, 5i3o.2.A, 5i4n.1.A, 5i5z.1.A, 5i8a.1.A, 5i9v.1.A, 5i9w.1.A, 5i9y.1.A, 5ia0.2.A, 5ia0.3.A, 5ia1.1.A, 5ia4.1.A, 5idn.1.A, 5idp.1.A, 5if1.1.A, 5if1.2.A, 5ig1.1.A, 5ig1.2.A, 5igh.1.A, 5igr.1.A, 5igv.1.A, 5igy.1.A, 5igz.1.A, 5ih0.1.A, 5ih1.1.A, 5ih4.1.A, 5iha.1.A, 5ijn.1.G, 5ikw.1.A, 5ime.1.A, 5ime.2.A, 5imx.1.A, 5iqc.1.A, 5iqh.1.A, 5iqh.4.A, 5iqi.1.A, 5iqi.4.A, 5iso.1.A, 5iso.2.A, 5ita.1.A, 5ita.2.A, 5itd.1.B, 5iu2.2.A, 5iuh.1.A, 5iwu.1.A, 5izj.1.A, 5izj.2.A, 5j0a.1.A, 5j0a.1.B, 5j1v.2.A, 5j1v.3.A, 5j5t.1.A, 5j79.1.A, 5j7s.1.A, 5j87.1.A, 5j87.1.D, 5j8i.1.A, 5j95.1.A, 5j95.2.A, 5j9l.1.A, 5j9y.1.A, 5j9z.1.A, 5jeb.1.A, 5jfs.1.A, 5jga.1.A, 5jh6.1.A, 5jk3.1.A, 5jkg.1.A, 5jn2.1.A, 5jr7.1.A, 5jr7.2.A, 5jrq.1.A, 5jsm.2.A, 5jsm.2.B, 5jy7.1.E, 5jy7.1.F, 5jy7.1.H, 5jzj.1.A, 5jzj.2.A, 5jzn.1.A, 5k00.1.A, 5k0k.1.A, 5k0x.1.A, 5k0x.2.A, 5k3y.1.A, 5k3y.2.A, 5k4i.1.A, 5k4j.1.A, 5k5n.1.A, 5k5x.1.A, 5k72.1.A, 5k75.3.A, 5k7i.1.A, 5k9i.2.A, 5kbq.1.A, 5kbq.2.A, 5kbr.1.A, 5kc2.1.B, 5kcv.1.A, 5kcx.1.A, 5khw.2.A, 5khx.1.A, 5kkr.1.A, 5kkr.1.B, 5kmi.1.A, 5kmk.1.A, 5kml.1.A, 5knj.1.A, 5ko1.1.A, 5kpk.2.A, 5kpm.1.A, 5ku8.1.A, 5kvt.1.A, 5kx7.2.A, 5kx8.1.A, 5kx8.2.A, 5kx8.3.A, 5kx8.4.A, 5kz8.1.A, 5kz8.2.A, 5l1z.2.A, 5l2i.1.A, 5l2q.1.A, 5l2q.3.A, 5l2q.4.A, 5l2s.1.A, 5l2t.1.A, 5l2w.1.A, 5l3a.1.A, 5l4g.1.6, 5l4q.1.A, 5l6w.1.A, 5l8k.1.A, 5l8l.1.A, 5lar.1.A, 5lcj.1.A, 5lck.1.A, 5lcq.1.A, 5lcr.1.A, 5li1.1.A, 5li9.1.A, 5lih.1.A, 5ljj.1.A, 5lma.1.A, 5lmk.2.A, 5ln3.1.U, 5loh.1.A, 5lpw.1.A, 5lpz.1.A, 5lqf.1.A, 5lvp.1.A, 5lw1.2.B, 5lwm.1.A, 5lxc.1.A, 5lxd.1.A, 5lxm.1.A, 5m06.1.A, 5m07.1.A, 5m08.1.A, 5m08.2.A, 5m09.1.A, 5m09.2.A, 5m0l.1.A, 5m32.1.6, 5m44.1.A, 5m4c.1.A, 5m4f.1.A, 5m4i.1.A, 5m4u.1.A, 5m51.1.A, 5m53.1.A, 5m56.1.A, 5m56.2.A, 5m57.1.A, 5mag.1.A, 5mah.1.A, 5mai.1.A, 5mhq.1.A, 5mja.1.A, 5mjb.1.A, 5ml5.1.A, 5mo4.1.A, 5mov.1.A, 5mow.2.A, 5mqf.1.K, 5mqv.1.A, 5mqv.5.A, 5mrb.1.A, 5mrd.1.A, 5mty.1.A, 5mxx.1.A, 5my8.1.A, 5myv.2.A, 5myv.3.A, 5myv.4.A, 5mz3.1.A, 5mzl.1.A, 5n1g.1.A, 5n1v.1.A, 5n23.1.A, 5n4v.1.A, 5n63.1.A, 5n65.1.A, 5n7v.1.A, 5n84.1.A, 5n93.1.A, 5na0.1.A, 5nad.1.A, 5ncl.1.A, 5nev.1.A, 5ng0.1.A, 5ng2.1.A, 5ng3.1.A, 5ngu.1.A, 5nhh.1.A, 5nk0.1.A, 5nk3.1.A, 5nk5.1.A, 5nk7.1.A, 5nka.1.A, 5nqc.1.A, 5ntj.1.A, 5ntt.1.A, 5nud.1.A, 5nud.2.A, 5nwz.1.A, 5nwz.2.A, 5nxc.1.A, 5nxd.1.A, 5nxd.2.A, 5nzz.1.B, 5nzz.2.B, 5o0y.1.A, 5o11.1.A, 5o1s.1.A, 5o1v.1.A, 5o1v.1.B, 5o21.1.A, 5o23.1.A, 5o23.2.A, 5o26.1.A, 5o2b.1.A, 5o2b.2.A, 5o2c.1.A, 5o7i.1.A, 5o8u.1.A, 5o8v.1.A, 5o90.1.A, 5oat.1.A, 5obj.1.A, 5obr.1.A, 5odt.1.A, 5okt.2.A, 5okt.4.A, 5omg.1.A, 5omy.1.A, 5one.1.A, 5oni.1.A, 5oo0.1.A, 5oo1.1.A, 5ooi.1.A, 5oop.1.A, 5op2.1.A, 5opu.1.A, 5opv.1.A, 5oq5.1.A, 5oq6.1.A, 5oq7.1.A, 5oq8.1.A, 5orl.1.A, 5os5.1.A, 5os7.1.A, 5osd.1.A, 5osl.1.A, 5osm.1.A, 5osz.1.A, 5otf.1.A, 5otq.1.A, 5owl.1.A, 5owq.3.B, 5owr.1.A, 5oy4.1.A, 5oy6.3.A, 5oy6.4.A, 5p9f.1.A, 5p9k.1.A, 5p9m.1.A, 5qik.1.A, 5qin.1.A, 5r8z.1.A, 5r9d.1.A, 5s7g.1.A, 5s86.1.B, 5sav.1.A, 5saw.1.A, 5sax.1.A, 5say.1.A, 5say.2.A, 5sb1.1.A, 5sb2.1.A, 5swh.1.A, 5sys.1.A, 5t0g.1.S, 5t0h.1.F, 5t0i.1.F, 5t0j.1.T, 5t0p.1.A, 5t31.1.A, 5t3q.1.B, 5t68.1.A, 5t8o.1.A, 5t8p.1.A, 5t8q.1.A, 5t8q.2.A, 5ta6.1.A, 5tbe.1.A, 5tc0.1.A, 5tc0.2.A, 5tco.1.A, 5td2.1.A, 5td2.2.A, 5te0.1.A, 5teh.1.A, 5teh.2.A, 5tex.1.A, 5tf9.1.A, 5tiu.1.A, 5tkd.1.A, 5to8.1.A, 5tob.1.A, 5toe.1.A, 5tos.1.A, 5tq3.1.A, 5tq4.1.A, 5tq5.1.A, 5tq6.1.A, 5tq7.1.A, 5tqw.1.A, 5tqx.1.A, 5tqy.1.A, 5tr6.1.A, 5ts8.1.A, 5tts.1.A, 5tur.1.A, 5tvt.1.A, 5twl.1.A, 5twy.1.A, 5twy.2.A, 5twz.1.A, 5tx3.2.A, 5u6b.1.A, 5u6b.2.A, 5u6c.1.A, 5u6c.1.B, 5u6y.1.A, 5u6y.1.B, 5u6y.1.C, 5u6y.1.D, 5u6y.1.E, 5u6y.1.F, 5u6y.1.G, 5u6y.1.H, 5u6y.1.I, 5u6y.1.J, 5u6y.1.K, 5u6y.1.L, 5u7q.1.A, 5u7q.2.A, 5u7r.1.A, 5u7r.1.B, 5u7r.2.B, 5u94.1.A, 5u9d.1.A, 5uab.1.A, 5ufu.1.A, 5ugc.1.A, 5ugl.1.A, 5ugx.1.A, 5uhn.1.A, 5ui0.1.A, 5ui0.2.A, 5uiq.1.A, 5uir.1.A, 5uit.1.A, 5uiu.2.A, 5uk8.1.B, 5ukf.1.A, 5ukf.2.A, 5ukk.1.A, 5ukl.1.A, 5ukm.1.A, 5ul1.1.B, 5umo.1.A, 5unp.1.A, 5uor.1.A, 5uor.2.A, 5uox.2.A, 5up3.1.A, 5upk.1.B, 5upl.1.A, 5uq1.1.A, 5uq2.1.A, 5uq3.1.A, 5ur1.2.A, 5usq.1.A, 5usy.1.A, 5uu1.1.A, 5uuu.1.A, 5uv4.1.A, 5uvc.1.A, 5uwd.1.A, 5uxa.2.A, 5uxb.1.A, 5uxb.2.A, 5uxc.1.A, 5uxd.1.A, 5uxd.2.A, 5uy6.1.A, 5v5n.1.A, 5v5y.1.A, 5v60.1.A, 5v61.1.A, 5v62.1.A, 5val.2.A, 5vam.1.A, 5vc3.1.A, 5vc5.1.A, 5vc6.1.A, 5vcv.1.A, 5vcw.1.A, 5vcw.2.A, 5vcx.1.A, 5vcy.1.A, 5vd0.1.A, 5vd1.1.A, 5vd2.1.A, 5vd3.1.A, 5vd4.1.A, 5vd5.1.A, 5vd7.1.A, 5vd8.1.A, 5vd9.1.A, 5vda.1.A, 5vdk.1.A, 5ve6.1.A, 5ved.1.A, 5vee.1.A, 5vef.1.A, 5vfs.1.g, 5vib.1.A, 5vio.1.A, 5vio.2.A, 5vio.3.A, 5vio.4.A, 5vja.1.B, 5vja.1.C, 5vlo.1.A, 5vlo.2.A, 5vnd.1.A, 5vnd.2.A, 5vt1.1.A, 5vub.1.A, 5w4w.2.A, 5w5j.1.A, 5w5j.1.B, 5w5o.1.A, 5w5v.1.A, 5w7t.1.A, 5w7t.2.A, 5w84.1.A, 5w86.1.A, 5w86.3.A, 5wax.1.A, 5wax.2.A, 5wdy.1.A, 5wdy.2.A, 5we8.1.A, 5we8.2.A, 5wev.1.A, 5wg3.1.A, 5wg5.1.A, 5wij.1.A, 5wjj.1.A, 5wne.1.B, 5wne.2.A, 5wng.1.A, 5wng.1.B, 5wnh.2.A, 5wnh.2.B, 5wni.1.A, 5wno.1.A, 5wp1.1.A, 5wr7.1.A, 5wvd.1.A, 5x02.1.A, 5x17.1.A, 5x17.2.A, 5x18.1.A, 5x28.1.A, 5x2a.1.A, 5x2a.1.B, 5x2f.1.A, 5x2f.2.A, 5x2f.2.B, 5x2k.1.A, 5x3f.1.B, 5x5o.1.A, 5x8i.1.A, 5xd6.1.A, 5xd6.2.A, 5xdl.1.A, 5xff.1.A, 5xfj.1.A, 5xgm.1.A, 5xgn.1.A, 5xgn.1.B, 5xka.1.A, 5xp5.1.A, 5xp7.2.A, 5xqx.1.A, 5xv7.1.A, 5xvf.1.A, 5xvu.1.A, 5xy1.1.A, 5xyx.1.A, 5xyz.1.A, 5xzv.1.A, 5xzv.2.A, 5y25.1.A, 5y5t.1.A, 5y5u.1.A, 5y5u.2.A, 5y7z.1.A, 5y80.1.A, 5y86.1.A, 5y8u.1.A, 5y90.1.A, 5y9m.2.A, 5y9t.1.A, 5ya5.1.A, 5yj9.1.A, 5yjz.1.A, 5yks.1.A, 5yt3.1.A, 5yt3.1.B, 5yt3.2.A, 5yu9.3.A, 5yu9.4.A, 5yva.1.A, 5ywm.1.A, 5yzg.1.K, 5z1d.1.A, 5z1e.1.A, 5z33.1.A, 5zan.1.A, 5zj6.1.A, 5zjw.1.A, 5zn0.1.A, 5zn2.1.A, 5zn3.1.A, 5zn4.1.A, 5zn5.1.A, 5zto.1.A, 5zv2.1.A, 5zv2.2.A, 5zwj.1.A, 5zxb.1.A, 5zxb.1.B, 5zz4.1.A, 6a1c.1.A, 6a1f.1.A, 6a1g.1.A, 6a32.1.A, 6aaj.1.A, 6aaj.1.B, 6aak.1.B, 6aak.2.A, 6aam.1.A,

6ac9.1.A, 6ac9.3.A, 6ae3.1.A, 6ae3.2.A, 6agx.1.A, 6agx.1.B, 6ao5.1.A, 6ate.1.A, 6ath.1.A, 6aua.1.A, 6aub.1.A, 6ayd.1.A, 6b1u.1.A, 6b1u.2.A, 6b2e.1.A, 6b2p.1.A, 6b2q.1.A, 6b3e.1.A, 6b4w.1.A, 6b5j.4.A, 6b8j.1.A, 6bab.1.A, 6bab.2.A, 6bbu.1.A, 6bbv.1.A, 6bdl.1.A, 6bdn.1.A, 6bfa.1.A, 6bfn.1.A, 6bfn.2.A, 6bg2.1.A, 6bg2.2.A, 6bg2.3.A, 6bhc.1.A, 6bik.1.A, 6bl8.1.A, 6bl8.2.A, 6ble.1.A, 6bny.1.A, 6bod.1.A, 6boe.1.A, 6bql.1.A, 6bqq.1.A, 6brj.1.A, 6bsd.1.A, 6buu.1.A, 6bwk.1.A, 6bx6.1.A, 6bxi.1.A, 6byr.2.A, 6byr.3.A, 6bys.1.C, 6c0u.1.A, 6c2t.1.B, 6c2y.1.A, 6c3e.1.A, 6c3e.1.B, 6c4d.1.A, 6c4d.2.A, 6c4d.3.A, 6c4d.4.A, 6c7y.1.A, 6c83.1.B, 6c9d.1.A, 6c9f.1.A, 6cad.1.A, 6cad.2.A, 6ccf.1.A, 6ccy.1.A, 6cd6.1.A, 6cd7.1.A, 6cd7.2.A, 6cdt.1.A, 6ch4.1.A, 6cjw.1.A, 6ckx.2.A, 6cmj.1.A, 6cn9.1.A, 6cn9.2.A, 6cnh.1.A, 6cpe.1.A, 6cpf.1.A, 6cpg.1.A, 6cpy.1.A, 6cpy.2.A, 6cqd.1.A, 6cqd.1.B, 6cqe.1.A, 6cqf.1.A, 6cth.1.A, 6ctz.1.A, 6cz3.1.A, 6cz4.1.A, 6d1y.1.A, 6d22.1.A, 6d2i.1.A, 6d3k.1.A, 6d3k.1.B, 6d3k.2.B, 6d3l.1.A, 6d5y.1.A, 6d8e.1.A, 6dc0.1.A, 6dc0.1.B, 6dd4.2.A, 6dfl.1.A, 6di5.1.A, 6di9.1.A, 6dkb.1.A, 6dkg.1.A, 6dkw.1.A, 6dkw.2.A, 6dmg.1.A, 6dtl.1.A, 6dtl.2.A, 6duk.1.A, 6duk.3.B, 6e0r.1.A, 6e2o.1.A, 6e2o.1.B, 6e4f.1.A, 6e6e.2.A, 6e6e.4.A, 6e6e.6.A, 6e6e.7.A, 6e9w.1.A, 6e9w.1.B, 6eac.1.A, 6eas.1.A, 6ed6.1.A, 6ed6.2.A, 6edl.1.A, 6ef6.1.A, 6eg9.1.A, 6eg9.1.B, 6ega.1.B, 6egd.1.A, 6egf.1.A, 6ehu.1.A, 6ehu.2.A, 6eif.2.A, 6eij.4.A, 6eil.2.A, 6eim.1.A, 6eim.1.B, 6eiq.1.A, 6eiv.2.A, 6eiv.3.A, 6eix.1.A, 6ej4.1.A, 6ej4.2.A, 6ej4.3.A, 6ej4.4.A, 6ekd.1.A, 6elr.1.A, 6emh.4.A, 6eml.1.4, 6ep9.1.A, 6eq9.1.A, 6eq9.2.A, 6eqi.1.C, 6es0.1.A, 6es0.2.A, 6esa.1.A, 6ewx.1.A, 6ewx.1.B, 6f14.1.A, 6f1w.1.A, 6f1w.2.A, 6f26.1.A, 6f26.2.A, 6f3d.1.A, 6f3e.2.A, 6f3f.1.A, 6f3i.1.A, 6f5e.1.B, 6f7b.1.A, 6fad.1.A, 6fad.3.A, 6fai.1.J, 6fcf.1.A, 6fd3.1.A, 6fdm.1.A, 6fdm.1.B, 6fdn.1.A, 6fdn.1.B, 6fdo.1.A, 6fdo.2.A, 6fdy.1.A, 6fdz.1.A, 6fek.1.A, 6fer.1.A, 6few.1.A, 6fha.1.A, 6fhb.1.A, 6fil.1.A, 6fio.1.A, 6fiq.1.A, 6fjz.1.A, 6fnf.1.A, 6fng.1.A, 6fnh.1.A, 6fnh.2.A, 6fnh.3.A, 6fni.1.A, 6fnj.1.A, 6fnl.1.A, 6frx.1.A, 6ft7.1.A, 6ft9.3.A, 6fu5.1.B, 6fuc.1.A, 6fvf.1.A, 6fyi.1.A, 6fyk.1.A, 6fyk.2.A, 6fyk.3.A, 6fyl.1.A, 6fyo.1.A, 6fyp.1.A, 6fyr.1.A, 6fyv.1.A, 6g18.1.7, 6g38.1.A, 6g39.1.A, 6g3a.1.A, 6g3c.1.A, 6g4j.1.A, 6g51.1.W, 6g54.1.A, 6g76.1.A, 6g78.1.A, 6g9a.1.A, 6g9n.1.A, 6gcr.1.A, 6gcw.1.A, 6gcw.1.B, 6gcx.1.A, 6ges.1.A, 6ggh.1.A, 6gi6.1.A, 6gin.1.A, 6gjo.2.A, 6gn1.1.A, 6gqj.1.A, 6gqk.1.A, 6gqo.1.A, 6gr8.1.A, 6gr9.1.A, 6gra.1.A, 6gro.1.A, 6gtt.1.A, 6gu2.1.A, 6gu6.1.A, 6gu7.2.A, 6gub.1.A, 6gub.2.A, 6guc.1.A, 6guc.2.A, 6gue.1.A, 6gue.2.A, 6guf.1.A, 6guf.2.A, 6guh.1.A, 6guk.1.A, 6gva.1.A, 6gvj.1.A, 6gvx.1.A, 6gwr.1.A, 6gwr.1.B, 6gzd.1.A, 6gzh.1.A, 6gzm.1.A, 6gzm.1.B, 6h0u.1.B, 6h3k.1.A, 6h7w.1.C, 6h7w.1.D, 6h7w.1.J, 6hbn.2.A, 6hd6.1.A, 6hd6.2.A, 6hdp.1.A, 6hdr.1.A, 6hh1.1.A, 6hhf.1.A, 6hhg.1.A, 6hhh.1.A, 6hho.1.A, 6hho.2.A, 6hjj.1.A, 6hjk.1.A, 6hk3.1.A, 6hk3.2.A, 6hk4.2.A, 6hk6.1.A, 6hk6.2.A, 6hk7.1.A, 6hkm.1.A, 6hkn.1.A, 6hm6.1.A, 6hmd.1.A, 6hme.1.A, 6hmp.1.A, 6hmq.1.A, 6hmr.2.A, 6hmx.1.A, 6hp9.1.A, 6hp9.2.A, 6hv0.1.A, 6hvd.1.A, 6hvf.2.A, 6hwj.1.A, 6hwj.2.A, 6hwk.1.A, 6hwk.1.D, 6hwu.1.A, 6hwv.1.A, 6hx1.1.A, 6hxf.1.A, 6hxf.3.A, 6hzu.2.A, 6hzv.1.A, 6i1s.1.A, 6i2d.1.A, 6i2p.1.A, 6i2u.1.A, 6i2y.1.A, 6i2y.1.B, 6i5i.1.A, 6i82.1.A, 6i82.1.B, 6i83.1.A, 6i8z.1.A, 6i99.1.A, 6i99.2.A, 6ib0.1.A, 6ib2.1.A, 6id0.1.4, 6id1.1.3, 6ig8.1.A, 6iii.1.A, 6il3.1.A, 6ilz.1.A, 6ilz.2.A, 6ilz.3.A, 6ilz.4.A, 6in0.1.A, 6in4.1.A, 6iqn.1.A, 6iqn.1.B, 6ise.1.A, 6itj.2.A, 6itt.1.A, 6itt.1.B, 6itv.1.A, 6iuo.1.A, 6iup.1.A, 6iy9.1.A, 6iy9.2.A, 6j5l.1.A, 6j5t.1.G, 6j5t.1.N, 6j5u.1.B, 6j5u.1.C, 6j5v.1.B, 6j5v.1.C, 6j5w.1.A, 6j6i.1.A, 6j6i.1.B, 6j6m.1.A, 6jgm.1.A, 6jkk.1.A, 6jlr.1.A, 6jmf.1.A, 6joi.1.A, 6joj.1.A, 6jol.1.A, 6jpe.1.A, 6jpj.1.A, 6jqr.1.A, 6jrj.1.A, 6jrk.1.A, 6jrx.1.A, 6jut.1.A, 6jux.1.A, 6jwl.1.A, 6jx0.1.A, 6jx4.1.A, 6jxt.1.A, 6jz0.1.A, 6k0j.1.A, 6k20.1.A, 6k3l.1.A, 6ka4.1.A, 6khd.1.A, 6khd.2.A, 6khd.3.A, 6khe.1.A, 6khf.1.A, 6kla.1.B, 6kmh.1.A, 6kmh.2.A, 6kyq.1.A, 6kyr.1.A, 6kzc.1.A, 6l20.1.A, 6l20.2.A, 6l20.3.A, 6l20.4.A, 6l21.1.A, 6l22.1.A, 6l23.1.A, 6l24.1.A, 6l8l.1.A, 6l8l.2.A, 6l8l.3.A, 6l8l.4.A, 6lba.1.A, 6lk5.1.A, 6lk6.1.A, 6ln1.1.A, 6ln1.2.A, 6lnm.1.A, 6lub.1.A, 6lvk.1.A, 6lvm.1.A, 6lxy.1.A, 6m0u.1.A, 6m13.1.A, 6m7z.1.A, 6m7z.3.A, 6m7z.4.A, 6m7z.5.A, 6m7z.6.A, 6m95.1.A, 6m9h.1.A, 6m9l.1.A, 6mcq.1.A, 6mcq.2.A, 6mep.1.A, 6mep.1.B, 6mib.1.A, 6mm5.1.A, 6mm6.1.A, 6mnh.1.A, 6mny.1.A, 6mny.2.A, 6mob.1.A, 6mom.1.A, 6msb.1.R, 6msd.1.R, 6mse.1.R, 6msg.1.R, 6msh.1.R, 6msj.1.R, 6msk.1.R, 6mwe.2.A, 6mx8.1.A, 6myn.1.A, 6mzw.1.A, 6n0p.1.A, 6n3l.1.A, 6n3n.1.A, 6n3o.1.A, 6n6o.1.A, 6n7a.1.A, 6n7c.2.A, 6n8g.1.A, 6n8g.3.A, 6nbs.1.A, 6ncg.1.A, 6ncg.2.A, 6ne7.1.A, 6nfi.1.A, 6nfy.1.A, 6nfz.1.A, 6nfz.1.B, 6ng0.1.A, 6ng0.1.B, 6no7.1.A, 6no7.2.A, 6no7.2.C, 6no8.1.A, 6npt.1.A, 6npy.1.B, 6npz.1.A, 6npz.2.A, 6nsp.1.A, 6nss.1.A, 6nt9.1.A, 6nvk.1.A, 6nvl.1.A, 6nvl.2.A, 6nvl.3.A, 6nw2.1.A, 6nw2.2.A, 6nyb.1.A, 6nyb.1.B, 6nyh.1.A, 6nyh.2.A, 6nyk.1.A, 6nzm.1.A, 6nzr.1.A, 6o5z.1.A, 6o5z.2.A, 6o6q.1.A, 6o6q.1.B, 6o8b.1.D, 6o8c.1.A, 6o8c.1.B, 6o8i.1.A, 6o8u.1.A, 6o8u.2.A, 6o8u.3.A, 6o94.1.A, 6o94.2.A, 6o94.3.A, 6o9d.2.A, 6o9l.1.3, 6ocq.1.A, 6ocq.2.A, 6ohd.1.A, 6oid.1.A, 6oko.1.A, 6ol2.1.A, 6op9.1.A, 6opg.1.A, 6opi.1.A, 6opk.1.A, 6oqi.1.A, 6oqo.1.A, 6ot6.1.A, 6ots.1.A, 6ova.1.A, 6oyt.1.A, 6oyt.1.B, 6oyw.1.A, 6oyw.1.B, 6p1d.1.A, 6p1d.1.B, 6p1d.2.A, 6p1d.2.B, 6p1l.1.B, 6p1l.2.A, 6p3d.1.A, 6p5m.1.A, 6p5m.3.A, 6p5p.4.A, 6p5s.1.A, 6p68.3.A, 6p69.1.A, 6p7g.1.A, 6p7g.1.B, 6p7g.2.B, 6p8e.1.B, 6p8f.1.B, 6p8g.1.B, 6p8h.1.B, 6p8q.1.A, 6p8q.2.A, 6paw.1.A, 6paw.2.A, 6paw.4.A, 6pdj.1.A, 6pjj.1.C, 6pjx.1.A, 6pk6.1.A, 6pl2.1.A, 6pl3.1.A, 6pma.1.A, 6pme.1.B, 6pme.1.D, 6pme.1.L, 6pnx.1.A, 6pnx.1.B, 6pp9.1.A, 6pp9.1.B, 6pwd.1.A, 6pwg.2.A, 6pxn.1.A, 6pxn.2.A, 6pxo.3.A, 6pxo.3.B, 6pxp.1.A, 6pxp.2.A, 6q0j.1.A, 6q0k.1.A, 6q0k.1.B, 6q0t.1.A, 6q0t.1.B, 6q0t.1.C, 6q2a.1.A, 6q2a.15.A, 6q2a.2.A, 6q2a.7.A, 6q38.1.A, 6q3b.1.A, 6q48.1.A, 6q4d.1.A, 6q4g.1.A, 6q4h.1.A, 6q4i.1.A, 6q4k.1.A, 6q4q.1.A, 6q8k.1.A, 6qaq.1.A, 6qas.1.A, 6qas.2.A, 6qat.1.A, 6qat.2.A, 6qau.1.A, 6qau.2.A, 6qav.1.A, 6qav.2.A, 6qav.2.B, 6qdv.1.I, 6qfl.1.A, 6qfr.1.A, 6qmo.1.A, 6qn4.1.A, 6qp5.1.A, 6qtg.1.A, 6qx9.56.A, 6qxk.1.A, 6qy7.1.A, 6qy9.1.A, 6qyx.1.A, 6r3d.1.A, 6r3s.1.A, 6r5f.2.A, 6r5f.3.A, 6r5f.4.A, 6r5k.1.F, 6r5k.1.G, 6ra5.1.A, 6ra7.1.A, 6rbd.1.W, 6rcg.1.A, 6rch.1.A, 6rch.1.B, 6rct.2.A, 6rfi.2.A, 6rfo.1.A, 6rfp.1.A, 6rln.1.A, 6rsb.1.A, 6rsr.1.A, 6rst.1.A, 6rsu.1.A, 6ru8.2.A, 6ru8.3.A, 6ruu.1.A, 6ruu.1.B, 6ruu.2.A, 6s11.1.A, 6s11.2.A, 6s1f.1.B, 6s1i.3.A, 6s1i.4.A, 6s1j.1.A, 6s73.1.A, 6s73.2.A, 6s73.3.A, 6s73.4.A, 6s75.1.A, 6s75.2.A, 6s75.3.A, 6s75.4.A, 6s76.1.A, 6s76.2.A, 6s76.3.A, 6s76.4.A, 6s89.1.A, 6s90.1.A, 6s90.2.A, 6s9b.1.A, 6s9c.1.A, 6s9d.1.A, 6s9x.1.A, 6sd9.1.A, 6sdc.1.A, 6sdd.1.A, 6sde.1.A, 6seq.1.A, 6sfk.1.A, 6sfo.1.A, 6sg4.1.A, 6sg4.1.C, 6sgd.1.A, 6so4.1.A, 6soi.1.A, 6sov.1.A, 6spw.1.A, 6srh.1.A, 6srh.2.A, 6sui.1.A, 6sul.1.A, 6sum.1.A, 6sun.1.A, 6syt.1.A, 6t28.1.A, 6t2w.1.A, 6t41.1.A, 6t6a.2.A, 6t6d.3.A, 6t6d.4.A, 6t6f.1.A, 6t6f.2.A, 6t8x.1.A, 6t8x.1.D, 6t8x.1.F, 6tca.1.A, 6tca.1.B, 6tca.2.A, 6tca.2.B, 6tca.3.A, 6tca.3.B, 6tca.4.A, 6tcu.1.A, 6td3.1.B, 6tew.1.A, 6tfp.1.A, 6tfp.2.A, 6tfp.4.A, 6tfu.1.A, 6tfv.2.A, 6tgu.1.A, 6thw.1.A, 6thx.2.A, 6ti8.1.A, 6ti8.4.A, 6tia.1.A, 6tia.2.A, 6tll.1.A, 6tlo.1.A, 6tn9.1.A, 6tnb.1.A, 6tnc.1.A, 6tnd.1.A, 6tpa.1.A, 6tpd.1.A, 6tpe.1.A, 6tpf.1.A, 6tpi.1.A, 6tsz.1.A, 6tu9.1.A, 6tua.1.A, 6ty3.1.A, 6ty4.1.A, 6u0k.1.A, 6u2g.1.A, 6u2g.1.B, 6u2h.1.C, 6u2h.1.D, 6u42.439.A, 6u42.440.A, 6u42.441.A, 6u42.442.A, 6u5l.1.A, 6u69.1.A, 6u6a.1.A, 6uan.1.C, 6uan.1.D, 6uip.1.A, 6uip.3.A, 6ul8.1.A, 6umw.1.A, 6una.1.A, 6una.2.A, 6unp.1.A, 6unq.1.A, 6unr.1.A, 6urc.2.A, 6uuo.2.A, 6uuo.2.B, 6v2u.1.A, 6v2u.2.A, 6v2w.1.A, 6v2w.1.B, 6v34.1.A, 6v34.2.A, 6v5n.1.A, 6v5n.1.B, 6v5n.2.A, 6v5p.2.A, 6v66.1.A, 6v66.2.A, 6v6a.1.A, 6v6k.1.B, 6v6o.1.B, 6v6o.3.A, 6v6o.3.B, 6v6o.4.B, 6v6q.1.A, 6vbz.1.A, 6vbz.2.A, 6vc0.1.A, 6vc0.2.A, 6vc0.3.A, 6vg3.1.A, 6vg3.2.A, 6vg3.3.A, 6vgl.1.A, 6vgl.2.A, 6vhg.1.A, 6vnc.1.A, 6vnc.2.A, 6vne.1.A, 6vne.2.A, 6vng.2.A, 6vno.1.A, 6vov.1.A, 6vp6.1.B, 6vp8.1.A, 6vp8.1.B, 6vpg.1.A, 6vph.1.B, 6vpi.1.A, 6vpj.1.A, 6vpl.1.A, 6vpm.1.B, 6vqm.1.A, 6vre.1.A, 6vrf.1.A, 6vru.1.A, 6vvc.1.A, 6vvd.1.A, 6vvd.2.A, 6vvd.3.A, 6vve.1.A, 6vvg.1.A,

6vvg.1.B, 6vxr.1.A, 6vzk.1.A, 6w39.1.B, 6w3a.1.A, 6w3a.1.B, 6w3b.1.A, 6w3c.1.A, 6w3c.2.A, 6w3e.1.A, 6w3k.1.A, 6w4o.1.M, 6w7o.1.A, 6w8i.1.A, 6w8i.2.A, 6w8i.3.A, 6w9e.1.A, 6wa2.1.B, 6wa2.2.A, 6wa2.2.B, 6wak.1.A, 6wak.2.A, 6wak.2.B, 6whp.1.A, 6wjd.1.R, 6wjf.1.A, 6wjf.1.B, 6wpp.1.A, 6wpp.2.A, 6wqx.1.B, 6wqx.2.B, 6wtn.1.A, 6wxj.1.A, 6wxn.3.A, 6x5g.1.A, 6x8e.2.A, 6x8g.1.A, 6xag.1.C, 6xbz.1.C, 6xd3.1.C, 6xdb.1.A, 6xdd.1.B, 6xdd.2.A, 6xdf.1.A, 6xdf.1.B, 6xf1.1.A, 6xf1.2.A, 6xfp.1.A, 6xi8.1.B, 6xih.1.A, 6xih.2.A, 6xka.1.A, 6xl4.1.A, 6xl4.2.A, 6xl4.3.A, 6xl4.4.A, 6xlo.1.A, 6xr6.1.A, 6xr7.1.A, 6xrg.1.A, 6xv9.2.A, 6xx6.1.A, 6xx8.1.A, 6y23.1.A, 6y23.2.A, 6y4x.1.A, 6y6f.1.A, 6y6v.1.A, 6y7c.1.6, 6y7z.1.A, 6y9r.1.A, 6y9s.2.A, 6ya6.1.A, 6ya7.1.A, 6ya8.1.A, 6yat.1.A, 6yfz.1.A, 6yg0.1.A, 6yg1.1.A, 6yg2.1.A, 6yg3.1.A, 6yg4.1.A, 6yg6.1.A, 6yg6.2.A, 6yg7.1.A, 6ygn.1.A, 6ygn.2.A, 6yi8.1.A, 6yi8.2.A, 6yid.1.B, 6yid.2.A, 6ykd.1.A, 6yky.1.A, 6yky.2.A, 6yky.3.A, 6yky.4.A, 6yl1.1.A, 6ylc.1.A, 6ylk.1.A, 6yll.1.A, 6yll.2.A, 6yoj.1.A, 6yph.1.A, 6yq1.1.A, 6yr9.3.A, 6yt6.1.A, 6yt6.2.A, 6ytd.1.A, 6yty.1.A, 6yul.2.A, 6yvs.2.A, 6yvs.3.A, 6yvs.4.A, 6yvu.1.A, 6yvy.1.A, 6yvy.4.A, 6yxs.1.A, 6yxt.1.A, 6yxv.4.A, 6yzh.1.A, 6z19.1.A, 6z1c.1.A, 6z1q.1.A, 6z3r.1.A, 6z3u.1.B, 6z45.1.A, 6z4b.1.A, 6z4b.2.A, 6z4y.1.A, 6z50.1.A, 6z53.1.A, 6z55.1.A, 6z59.1.A, 6z83.1.A, 6z84.2.A, 6zgc.3.A, 6ziw.1.A, 6zjf.1.A, 6znm.1.H, 6znm.1.J, 6zqs.1.A, 6zr5.1.A, 6zr5.2.A, 6zv6.1.8, 6zxd.1.9, 6zxe.1.a, 6zxf.1.8, 6zxg.1.a, 7a04.1.A, 7a06.1.B, 7a2a.2.A, 7a49.2.A, 7a4b.1.A, 7a4o.1.A, 7a5b.2.A, 7a5p.1.L, 7a6i.1.A, 7aax.1.A, 7aay.1.A, 7aaz.1.A, 7ab1.1.A, 7aem.1.A, 7aj2.1.A, 7ajm.1.A, 7ajm.2.A, 7ajv.1.A, 7ajy.2.A, 7akm.1.A, 7akm.2.A, 7ako.1.A, 7ako.2.A, 7ank.1.A, 7apf.1.A, 7apg.2.A, 7apj.1.A, 7aqb.1.A, 7aqb.2.A, 7at5.2.A, 7at9.1.A, 7ats.1.A, 7atu.1.A, 7atv.1.A, 7avx.1.A, 7avx.2.A, 7avy.1.A, 7aw4.2.A, 7ax4.1.A, 7ayi.1.A, 7aym.1.A, 7b30.1.A, 7b36.1.A, 7b3m.1.A, 7b3q.1.A, 7b3w.1.A, 7b55.1.A, 7b5l.1.J, 7b5q.1.C, 7b6f.1.A, 7b7s.1.A, 7b85.1.A, 7b8h.1.A, 7bcm.1.A, 7be6.1.A, 7bl1.1.C, 7bmk.1.A, 7bmk.1.B, 7btt.1.A, 7bu4.1.A, 7byk.1.A, 7c2v.1.A, 7c2v.1.B, 7c2w.1.A, 7c2w.1.B, 7c2w.1.C, 7c2w.1.D, 7c3n.1.A, 7cbx.1.A, 7cc2.1.A, 7cc2.2.A, 7cga.1.A, 7cga.2.A, 7cga.3.A, 7cga.4.A, 7chm.1.A, 7chn.1.A, 7cht.1.A, 7cja.1.A, 7clh.1.A, 7cmb.1.A, 7cml.1.A, 7cp3.1.A, 7cp4.1.A, 7cqe.1.A, 7ctv.1.A, 7ctv.2.A, 7ctx.1.A, 7cy2.1.A, 7cyr.1.A, 7cz2.1.A, 7d5o.1.A, 7d6d.1.A, 7d6e.1.A, 7dd1.1.A, 7dg4.1.A, 7dh3.1.A, 7dhv.1.A, 7dsy.1.A, 7dt2.1.A, 7dts.1.A, 7dts.1.B, 7dtz.1.A, 7du8.1.B, 7du9.1.A, 7du9.1.B, 7dua.1.A, 7dv6.1.A, 7dxl.1.A, 7e0z.1.A, 7e11.1.A, 7e34.1.A, 7e73.1.A, 7e75.1.A, 7eec.1.A, 7eed.1.A, 7eef.1.A, 7egc.1.I, 7ejv.1.A, 7ejv.1.B, 7elw.1.B, 7emf.1.C, 7ena.2.A, 7ena.56.A, 7enc.24.A, 7enc.3.A, 7er2.1.A, 7f0a.1.A, 7f0b.1.A, 7f2x.1.A, 7f3g.1.A, 7f3g.1.B, 7f3m.1.A, 7f7w.1.A, 7f7w.2.A, 7fcz.1.A, 7fcz.1.B, 7fd0.1.A, 7feh.1.A, 7fhs.1.A, 7fht.4.A, 7fic.1.A, 7jhg.1.A, 7jhh.1.A, 7jij.1.B, 7jnt.1.A, 7jou.1.A, 7jov.4.B, 7ju6.1.B, 7juq.1.B, 7jur.1.B, 7jus.1.B, 7jut.1.A, 7jut.1.B, 7juv.1.A, 7juv.1.B, 7juw.1.A, 7jux.1.A, 7juy.1.B, 7jv1.1.B, 7jv7.1.A, 7jw7.1.A, 7jxh.1.A, 7jxh.5.A, 7jxk.1.A, 7jxl.1.A, 7jxm.1.A, 7jxu.2.A, 7jxx.1.A, 7jxy.1.A, 7jxy.2.A, 7k0v.1.A, 7k0v.1.B, 7k0v.1.C, 7k0v.1.D, 7k1h.1.A, 7k1h.1.B, 7k1h.2.A, 7k1h.2.B, 7k1h.3.B, 7k1i.1.A, 7k5b.1.C, 7k7l.1.A, 7k7z.1.A, 7kac.1.A, 7kac.1.B, 7khj.1.A, 7khj.2.A, 7khk.1.A, 7khk.2.A, 7kia.1.A, 7kja.1.A, 7kja.2.A, 7kjb.1.A, 7kjc.1.A, 7kjc.2.A, 7kjs.1.A, 7kl1.1.A, 7kp6.1.A, 7kp6.2.A, 7kpl.1.A, 7kpv.1.A, 7ksj.1.A, 7kue.1.B, 7kx6.1.A, 7kx6.2.A, 7kx8.1.A, 7kx8.2.A, 7kxl.1.A, 7kxq.1.A, 7kxw.1.A, 7kxw.2.A, 7kxz.1.A, 7kzm.1.6, 7kzm.1.9, 7l24.1.A, 7l24.2.A, 7l25.1.A, 7l26.1.A, 7l26.4.A, 7l5p.1.A, 7l5p.2.A, 7lbm.1.8, 7lbm.1.m, 7lg8.1.A, 7lg8.2.A, 7lg8.3.A, 7lg8.4.A, 7lgs.2.A, 7lht.1.A, 7lhw.1.A, 7li3.1.A, 7ll4.1.A, 7ll5.1.A, 7lqd.1.A, 7lty.1.A, 7lv3.1.A, 7lv3.1.B, 7lvh.1.A, 7lvh.2.A, 7m0k.1.A, 7m0l.1.A, 7m0m.1.A, 7m0m.1.B, 7m5z.1.A, 7m5z.2.A, 7m74.1.A, 7mf0.1.A, 7mfd.1.A, 7mfd.1.B, 7mfe.1.A, 7mff.1.C, 7mff.1.D, 7mgj.1.A, 7mgk.4.A, 7mon.1.A, 7mon.1.B, 7mp8.1.A, 7mt8.1.A, 7mt9.1.A, 7mta.1.A, 7mtb.1.A, 7mu7.1.A, 7mu7.2.A, 7mx3.1.A, 7mx3.2.A, 7mx3.3.A, 7mx3.4.A, 7mxb.1.A, 7mxb.2.A, 7mxj.1.A, 7mxk.1.A, 7myj.2.A, 7n3u.1.A, 7n4r.1.A, 7n8t.1.A, 7n91.1.A, 7n91.2.A, 7n93.2.A, 7n9g.1.A, 7n9g.2.A, 7naa.1.A, 7nb1.1.A, 7ncf.1.A, 7ng7.1.A, 7nj0.1.B, 7nqw.1.A, 7nry.1.B, 7nti.1.A, 7nur.1.A, 7nvq.1.A, 7nvr.1.e, 7nwk.1.A, 7nxj.1.A, 7nxj.2.A, 7nxk.1.A, 7nxk.2.A, 7o2v.1.A, 7o7i.1.A, 7o7j.1.A, 7o7k.1.A, 7o7k.2.A, 7oai.1.A, 7oam.1.A, 7oam.2.A, 7ogt.1.B, 7oow.1.A, 7oox.1.A, 7opm.1.A, 7opo.1.A, 7opo.4.A, 7opo.5.A, 7ore.1.A, 7orf.1.A, 7ote.1.A, 7ote.2.A, 7ovi.1.A, 7ovj.1.A, 7ovm.1.A, 7oxb.1.A, 7oy5.1.A, 7oy6.1.A, 7ozy.1.A, 7ozy.2.A, 7p1l.1.A, 7p3v.1.A, 7p3v.1.B, 7p5z.1.M, 7p7f.1.A, 7p7g.1.A, 7p7g.2.A, 7p7h.1.A, 7p7h.2.A, 7pcd.1.A, 7pi4.1.D, 7pqv.1.A, 7psu.1.A, 7pt6.1.O, 7pt7.1.G, 7pue.1.A, 7pvu.1.A, 7pvu.2.A, 7pwd.1.A, 7q4a.1.A, 7q4a.1.B, 7q52.1.A, 7q6h.1.A, 7q7i.1.A, 7q7l.1.A, 7q8v.1.A, 7q8y.1.A, 7q8y.2.A, 7qfm.1.A, 7qg2.1.A, 7qg2.4.A, 7qg3.1.A, 7qg5.2.A, 7qgp.1.A, 7qhw.1.A, 7qhw.2.A, 7qq6.1.A, 7qq6.1.B, 7qq6.2.A, 7qq6.2.B, 7qr9.3.A, 7que.1.A, 7que.2.A, 7quf.1.A, 7qux.1.A, 7qwk.1.A, 7qwk.5.A, 7qwk.6.A, 7qy7.1.R, 7qya.1.R, 7r5k.49.A, 7r9l.1.B, 7r9n.1.B, 7r9p.1.A, 7r9p.1.B, 7r9t.1.A, 7r9t.1.B, 7ra5.1.A, 7rj8.1.A, 7rn6.1.A, 7run.1.A, 7rwf.1.A, 7s25.1.A, 7s25.1.B, 7s25.2.A, 7s25.2.B, 7s26.1.A, 7s26.1.B, 7s26.2.A, 7s26.2.B, 7s3l.1.A, 7s46.1.A, 7s47.1.A, 7s48.1.A, 7s4t.1.A, 7s6u.1.A, 7s85.1.A, 7sa0.1.A, 7shv.1.A, 7siu.1.A, 7siu.1.B, 7sj3.1.A, 7sqk.1.B, 7sqm.1.A, 7sxf.1.A, 7sxg.1.A, 7sxh.1.A, 7sxj.1.A, 7sza.1.A, 7szc.1.A, 7szd.1.A, 7szr.1.A, 7szr.2.A, 7t0p.1.A, 7t3x.1.A, 7t4i.1.A, 7t4j.1.A, 7t4j.1.B, 7t4k.1.A, 7t4k.1.B, 7t4l.1.B, 7t4m.1.A, 7t4m.1.B, 7t4t.1.A, 7t4t.2.A, 7t4u.1.A, 7t4u.2.A, 7t4v.1.A, 7t4v.2.A, 7t6f.1.A, 7tbi.16.A, 7tbi.24.A, 7tdz.1.5, 7teu.1.A, 7tnh.1.A, 7tvd.1.A, 7tz7.1.B, 7u2z.1.A, 7u2z.2.A, 7u31.1.A, 7u98.3.A, 7u9a.1.A, 7udp.1.A, 7udq.1.A, 7udq.2.A, 7ug1.1.A, 7ugb.1.A, 7uiq.2.A, 7ujr.1.A, 7ujs.1.A, 7ujt.1.A, 7ukv.1.A, 7ukw.1.A, 7ukw.1.B, 7ukw.2.A, 7ukw.2.B, 7ukz.1.A, 7uos.1.A, 7up4.1.A, 7up4.2.A, 7up6.1.A, 7upm.1.A, 7uxi.1.A, 7uxk.1.A, 7uy0.1.A, 7uy0.2.A, 7uy3.1.A, 7uyr.1.A, 7uyv.1.A, 7uyv.3.A, 7uyw.1.A, 7v3r.1.A, 7v3v.1.M, 7vbt.1.A, 7vdp.1.A, 7vdp.2.A, 7vdq.1.A, 7vjl.1.A, 7vjl.2.A, 7vkm.1.A, 7vkn.1.A, 7vko.1.A, 7vnx.1.A, 7vop.1.2, 7vop.1.5, 7w15.2.A, 7w19.1.A, 7w19.2.A, 7w1a.1.A, 7w1a.2.A, 7w38.1.j, 7w39.1.F, 7w3a.1.F, 7w3b.1.F, 7w3c.1.E, 7w3f.1.F, 7w3g.1.F, 7w3h.1.F, 7w3i.1.G, 7w3j.1.B, 7w3k.1.D, 7w3m.1.F, 7w59.1.n, 7w5c.1.A, 7w5o.1.A, 7w5o.2.A, 7w7x.1.A, 7w7x.1.B, 7w7y.1.A, 7w7y.1.B, 7wcl.1.A, 7wcl.2.A, 7wcw.1.A, 7wcx.1.A, 7wf5.2.A, 7wj6.1.A, 7wj6.1.B, 7wkk.1.G, 7wkk.1.J, 7wkk.1.U, 7wkk.1.X, 7wtt.1.6, 7wu0.1.8, 7x4u.1.A, 7xaf.1.A, 7xbi.1.A, 7xbr.1.A, 7xbr.1.B, 7xbr.1.C, 7xbr.1.D, 7xbr.2.A, 7xbr.2.B, 7xbr.2.C, 7xbr.2.D, 7xdv.1.A, 7xdv.2.A, 7xdx.1.A, 7xdy.1.A, 7xdy.2.A, 7xmk.1.A, 7xmk.1.B, 7xqk.1.A, 7xsv.1.A, 7xyh.2.A, 7xzq.1.A, 7xzr.1.A, 7xzr.2.A, 7y1g.1.A, 7y1g.2.A, 7y4t.1.A, 7yaw.1.A, 7yaw.2.A, 7yaw.3.A, 7yaz.1.A, 7ybo.1.A, 7yc9.1.A, 7ydx.1.B, 7yl1.1.A, 7z1f.1.A, 7z1g.1.A, 7z37.1.C, 7z38.1.C, 7z4v.1.A, 7z5n.1.A, 7z5n.2.A, 7z5n.7.A, 7z5n.8.A, 7z5w.1.A, 7z6i.1.A, 7z9t.1.A, 7z9t.1.B, 7zh8.1.A, 7zh8.3.A, 7zks.1.A, 7zkx.1.A, 7zr0.1.D, 7zr5.1.D, 7zr6.1.D, 7ztl.1.A, 7zvs.1.A, 7zvs.2.A, 7zvs.3.A, 7zw8.1.A, 7zwe.1.A, 7zyn.1.A, 8a27.1.A, 8a2a.1.A, 8a2b.1.A, 8a2d.1.A, 8a5j.1.A, 8a66.1.A, 8a8m.1.A, 8a8m.1.B, 8aau.1.A, 8acm.1.A, 8afr.1.A, 8agy.1.A, 8ahd.1.A, 8ahg.1.A, 8ahp.1.A, 8ak2.1.A, 8am0.1.B, 8an8.1.A, 8ans.1.A, 8aof.1.A, 8arj.1.A, 8atl.1.A, 8atl.2.A, 8atn.1.A, 8atn.2.A, 8au3.1.A, 8au3.2.A, 8au5.1.A, 8auc.1.A, 8auc.2.A, 8av1.1.A, 8aw1.1.A, 8aza.1.B, 8aza.1.C, 8b8n.1.A, 8ba2.1.A, 8ba3.1.A, 8ba4.1.A, 8bab.1.A, 8bem.1.A, 8bi5.1.A, 8bjt.1.A, 8bju.1.A, 8bm2.1.A, 8bm2.2.A, 8boc.1.A, 8bok.1.A, 8bpv.1.A, 8bpw.1.A, 8bu1.1.B, 8bu1.2.B, 8bu2.1.B, 8bu3.3.B, 8bu4.3.B, 8bu5.1.B, 8bu5.2.B, 8bu5.3.B, 8bu6.1.B, 8bu6.2.B, 8bu6.3.B, 8bu7.1.B, 8bua.1.B, 8bua.2.B, 8bub.1.B, 8bub.2.C, 8bub.3.C, 8bug.3.B, 8bui.1.B, 8bui.2.B, 8buj.3.B, 8bum.1.B, 8buo.1.B, 8bus.1.B, 8bvw.1.I, 8bx6.1.A, 8bxc.1.A, 8bya.1.A, 8byq.1.I, 8bzo.1.A, 8bzp.1.A, 8c01.1.3, 8c6j.52.A, 8c7x.1.A, 8c7x.1.B, 8c7y.1.A, 8c7y.1.B, 8car.1.A, 8cav.1.A, 8cav.1.B,

8cbj.1.J, 8cdw.1.A, 8cdw.1.B, 8cgc.1.A, 8ch6.1.Z, 8cie.1.A, 8cij.1.A, 8cur.1.A, 8cvt.1.F, 8d6e.1.A, 8d73.1.A, 8d7m.1.A, 8d7n.2.A, 8d7o.2.A, 8d7p.1.A, 8deg.1.A, 8dgs.1.A, 8dgs.1.B, 8dks.1.A, 8dp5.1.A, 8dp5.1.B, 8ds6.1.A, 8ds6.1.B, 8dso.1.A, 8dsw.1.A, 8dwn.1.A, 8e04.1.A, 8e05.1.A, 8e06.1.A, 8e0l.1.A, 8e1x.1.A, 8e1x.1.B, 8e4t.1.A, 8edh.1.A, 8edh.2.A, 8efj.1.A, 8ej4.1.K, 8ejb.1.A, 8elc.1.A, 8eme.1.B, 8enj.1.A, 8eq5.1.A, 8eq9.1.A, 8eqd.1.A, 8erd.1.A, 8ewy.1.B, 8ex2.1.A, 8f1w.4.A, 8f1z.1.A, 8f7o.1.A, 8f7o.1.B, 8f7p.1.A, 8f7p.1.B, 8fac.1.A, 8fd9.1.A, 8fe2.1.A, 8fe2.2.A, 8fe5.2.A, 8fe9.1.A, 8fec.1.A, 8fec.2.A, 8ff0.1.A, 8fh4.1.A, 8fh4.2.A, 8fh4.3.A, 8fh4.4.A, 8fjz.1.A, 8fjz.1.B, 8fko.2.A, 8fko.3.A, 8flg.1.A, 8fll.1.A, 8fln.1.A, 8fow.1.A, 8fp1.1.A, 8fp3.1.A, 8fx4.1.D, 8g6z.1.A, 8g8o.1.A, 8gae.1.D, 8gds.2.A, 8gft.1.D, 8glv.195.A, 8glv.218.A, 8glv.346.A, 8glv.347.A, 8glv.359.A, 8glv.360.A, 8glv.953.A, 8glv.959.A, 8glv.960.A, 8gmb.1.A, 8gmc.1.A, 8gmc.2.A, 8gu7.1.A, 8gu7.2.A, 8guw.1.A, 8guw.1.B, 8guw.1.C, 8gw3.1.A, 8gw3.3.A, 8gxq.48.A, 8h4r.1.A, 8h59.1.A, 8h6p.1.A, 8h75.1.A, 8h75.1.B, 8h75.1.C, 8h75.1.D, 8h7f.1.A, 8h7f.2.A, 8haq.1.A, 8haq.2.A, 8hd0.1.E, 8hlt.1.A, 8hlt.2.A, 8hmt.1.A, 8hmt.2.A, 8ho6.1.A, 8hoa.1.A, 8hod.1.A, 8hoe.1.A, 8hv2.1.A, 8hv4.1.A, 8hv5.1.A, 8hy7.1.A, 8iah.1.5, 8iah.1.6, 8iah.1.7, 8iai.1.2, 8idc.1.C, 8idd.1.D, 8igq.1.A, 8iyj.505.A, 8iyj.506.A, 8iyj.507.A, 8j07.859.A, 8j07.860.A, 8j5w.1.A, 8j5x.1.A, 8j61.1.A, 8j63.1.A, 8jia.1.C, 8jmx.1.A, 8jpb.1.A, 8k5r.1.A, 8or0.1.G, 8or4.1.G, 8orm.1.C, 8otz.14.A, 8otz.15.A, 8otz.573.A, 8ouv.1.A, 8ov7.1.A, 8ow3.1.A, 8owg.3.A, 8oy2.1.A, 8p08.1.A, 8p4z.1.A, 8p4z.2.A, 8p6v.1.C, 8p7j.1.A, 8p7j.2.A, 8p81.1.A, 8pvp.1.A, 8pyi.1.A, 8pyj.1.A, 8pyk.1.A, 8pyl.1.A, 8q1z.1.A, 8q61.1.A, 8q77.1.A, 8qbu.1.A, 8qcg.1.A, 8qcg.2.A, 8qel.1.B, 8qf1.1.B, 8qlq.1.A, 8qlr.1.A, 8qqy.1.A, 8s93.1.A, 8s99.3.A, 8s9f.1.A, 8s9f.1.B, 8sam.1.A, 8sam.2.A, 8sao.1.B, 8sao.2.B, 8sap.1.A, 8sap.2.A, 8sbc.1.B, 8six.1.A, 8slz.1.B, 8soi.1.A, 8soi.1.B, 8sor.1.D, 8sqz.1.A, 8sqz.1.B, 8srm.1.A, 8srm.1.B, 8srq.1.E, 8ssn.1.A, 8ssn.2.A, 8sso.1.A, 8ssp.1.A, 8stg.1.A, 8stg.2.A, 8tb5.1.A, 8tdu.1.B, 8tdu.2.B, 8tgd.1.B, 8ts7.1.B, 8ts9.1.B, 8tsc.1.B, 8txz.1.A, 8tyq.1.A, 8tzb.1.A, 8tzc.1.A, 8tze.1.A, 8tzf.1.A, 8tzg.1.A, 8tzh.1.B, 8u2o.1.A, 8u95.1.A, 8u95.1.B, 8uap.1.A, 8uaq.1.A, 8w6j.1.E, 8wd4.1.A, 8wf4.1.A, 8wf4.2.A, 8wgf.1.A, 8wjy.1.A, 8wsw.4.A, 8wtf.1.A, 8x88.1.A, 8x88.2.A
