# Supplementary material for: Structural and functional characterization of peste des petits ruminants virus coded hemagglutinin protein using various in-silico approaches
Source: Front Microbiol. 2024 Jun 20;15:1427606. doi: 10.3389/fmicb.2024.1427606 (PMC11222573; doi:10.3389/fmicb.2024.1427606)
Supplement: Supplementary file 9 [file Data_Sheet_9.ZIP › Ikk-beta.pdf]

SWISS-MODEL Homology Modelling Report

Model Building Report

This document lists the results for the homology modelling project "Untitled Project" submitted to SWISS-MODEL workspace on Jan. 10, 2024, 8:11 a.m..The submitted primary amino acid sequence is given in Table T1.

If you use any results in your research, please cite the relevant publications:

- Waterhouse, A., Bertoni, M., Bienert, S., Studer, G., Tauriello, G., Gumienny, R., Heer, F.T., de Beer, T.A.P., Rempfer, C., Bordoli, L., Lepore, R., Schwede, T. SWISS-MODEL: homology modelling of protein structures and complexes. Nucleic Acids Res. 46(W1), W296-W303 (2018).
- Bienert, S., Waterhouse, A., de Beer, T.A.P., Tauriello, G., Studer, G., Bordoli, L., Schwede, T. The SWISS-MODEL Repository - new features and functionality. Nucleic Acids Res. 45, D313-D319 (2017).
- Studer, G., Tauriello, G., Bienert, S., Biasini, M., Johner, N., Schwede, T. ProMod3 - A versatile homology modelling toolbox. PLOS Comp. Biol. 17(1), e1008667 (2021).
- Studer, G., Rempfer, C., Waterhouse, A.M., Gumienny, G., Haas, J., Schwede, T. QMEANDisCo - distance constraints applied on model quality estimation. Bioinformatics 36, 1765-1771 (2020).
- Bertoni, M., Kiefer, F., Biasini, M., Bordoli, L., Schwede, T. Modeling protein quaternary structure of homo- and hetero-oligomers beyond binary interactions by homology. Scientific Reports 7 (2017).

Results

The SWISS-MODEL template library (SMTL version 2023-12-24, PDB release 2023-12-15) was searched with for evolutionary related structures matching the target sequence in Table T1. For details on the template search, see Materials and Methods. Overall 6675 templates were found (Table T2).

Models

The following model was built (see Materials and Methods "Model Building"):

Model #01	File	Built with	Oligo-State	Ligands	GMQE	QMEANDisCo Global
	PDB	ProMod3 3.4.0	homo-dimer (matching prediction)	2 x KSA: K-252A;	0.71	0.74 ± 0.05

Template	Seq Identity	Oligo-state	QSQE	Found by	Method	Resolution	Seq Similarity	Range	Coverage	Description
4kik.1.A	95.48	homo-dimer	0.66	HHblits	X-ray	2.83Å	0.60	9 - 663	0.88	Inhibitor of nuclear factor kappa-B kinase subunit beta

Included Ligands

Ligand	Description
2 x KSA	K-252A

```
Target     MSWSPSLPTQTCGAWEMKERLGTGGFGNVIRWHNQETGEQIAIKQCRQELSPRNRERWCLEIQIMRRLNHPNVVAARDVP
4kik.1.A   -SWSPSLTTQTCGAWEMKERLGTGGFGNVIRWHNQETGEQIAIKQCRQELSPRNRERWCLEIQIMRRLTHPNVVAARDVP

Target     EGMQSLAPNDLPLLAMEYCQGGDLRKYLNQFENCCGLREGAILTLLSDIASALRYLHENRIIHRDLKPENIVLQQGEQRL
4kik.1.A   EGMQNLAPNDLPLLAMEYCQGGDLRKYLNQFENCCGLREGAILTLLSDIASALRYLHENRIIHRDLKPENIVLQQGEQRL

Target     IHKIIDLGYAKELDQGSLCTSFVGTLQYLAPELLEQQKYTVTVDYWSFGTLAFECITGFRPFLPNWQPVQWHSKVRQKSE
4kik.1.A   IHKIIDLGYAKELDQGSLCTSFVGTLQYLAPELLEQQKYTVTVDYWSFGTLAFECITGFRPFLPNWQPVQWHSKVRQKSE

Target     MDIVVSEDLNGAVKFSSSLPHPNNLNSVLAQRLEKWLQLMLMWHPRQRGTDPVYGPNGCFKALDDILNLKLLHVLNMVTG
4kik.1.A   VDIVVSEDLNGTVKFSSSLPYPNNLNSVLAERLEKWLQLMLMWHPRQRGTDPTYGPNGCFKALDDILNLKLVHILNMVTG

Target     TLHTYPVTEDESLQSLKARIRQDTGILEEDQELLQEAGLALIPDKPAAQCLSDGKLNEGRTLDMDLVFLFDNSRVTYESQ
4kik.1.A   TIHTYPVTEDESLQSLKARIQQDTGIPEEDQELLQEAGLALIPDKPATQCISDGKLNEGHTLDMDLVFLFDNSKITYETQ
```

```
Target    VSPQPQPESVSCILQEPKRNLPFFQLRKVWGQVWHSIQALKEDCSRLQQGQRAAMMNLLRNNSCLSKMKNSMASMSQQLK
4kik.1.A  ISPRPQPESVSCILQEPKRNLAFFQLRKVWGQVWHSIQTLKEDCNRLQQGQRAAMMNLLRNNSCLSKMKNSMASMSQQLK

Target    AKLDFFKTSIQIDLEKYREQTEFGITSDKLLLAWREMEQAVELCGRENEVKHLVERMMALQTDIVDLQRSPMGRKQGGTL
4kik.1.A  AKLDFFKTSIQIDLEKYSEQTEFGITSDKLLLAWREMEQAVELCGRENEVKLLVERMMALQTDIVDLQRSPMGRKQGGTL

Target    DDLEEQARELYRRLREKPRDQRTDGDSQEMVRLLLQAIQGFEKKVRVIYTQLSKTVVCKQKALELLPKVEEVVSLMSEDE
4kik.1.A  DDLEEQARELYRRLREKPRDQRTEGDSQEMVRLLLQAIQSFEKKVRVIYTQLSKTVVCKQKALELLPKVEEVVSLMNEDE

Target    KMVVRLQEKRQKELWNLLKIACSKVRGPVSGSPDSMNASRLSHPCQLMSQPCTAPDSLPEAAEKSEDLVAEAHTLCTQLE
4kik.1.A  KTVVRLQEKRQKELWNLLKIACSK--------------------------------------------------------

Target    NALQDTMKEQDQSLRSLDWSWLQSEEEEQQSLERAS
4kik.1.A  ------------------------------------


Target    MSWSPSLPTQTCGAWEMKERLGTGGFGNVIRWHNQETGEQIAIKQCRQELSPRNRERWCLEIQIMRRLNHPNVVAARDVP
4kik.1.B  -SWSPSLTTQTCGAWEMKERLGTGGFGNVIRWHNQETGEQIAIKQCRQELSPRNRERWCLEIQIMRRLTHPNVVAARDVP

Target    EGMQSLAPNDLPLLAMEYCQGGDLRKYLNQFENCCGLREGAILTLLSDIASALRYLHENRIIHRDLKPENIVLQQGEQRL
4kik.1.B  EGMQNLAPNDLPLLAMEYCQGGDLRKYLNQFENCCGLREGAILTLLSDIASALRYLHENRIIHRDLKPENIVLQQGEQRL

Target    IHKIIDLGYAKELDQGSLCTSFVGTLQYLAPELLEQQKYTVTVDYWSFGTLAFECITGFRPFLPNWQPVQWHSKVRQKSE
4kik.1.B  IHKIIDLGYAKELDQGSLCTSFVGTLQYLAPELLEQQKYTVTVDYWSFGTLAFECITGFRPFLPNWQPVQWHSKVRQKSE

Target    MDIVVSEDLNGAVKFSSSLPHPNNLNSVLAQRLEKWLQLMLMWHPRQRGTDPVYGPNGCFKALDDILNLKLLHVLNMVTG
4kik.1.B  VDIVVSEDLNGTVKFSSSLPYPNNLNSVLAERLEKWLQLMLMWHPRQRGTDPTYGPNGCFKALDDILNLKLVHILNMVTG

Target    TLHTYPVTEDESLQSLKARIRQDTGILEEDQELLQEAGLALIPDKPAAQCLSDGKLNEGRTLDMDLVFLFDNSRVTYESQ
4kik.1.B  TIHTYPVTEDESLQSLKARIQQDTGIPEEDQELLQEAGLALIPDKPATQCISDGKLNEGHTLDMDLVFLFDNSKITYETQ

Target    VSPQPQPESVSCILQEPKRNLPFFQLRKVWGQVWHSIQALKEDCSRLQQGQRAAMMNLLRNNSCLSKMKNSMASMSQQLK
4kik.1.B  ISPRPQPESVSCILQEPKRNLAFFQLRKVWGQVWHSIQTLKEDCNRLQQGQRAAMMNLLRNNSCLSKMKNSMASMSQQLK

Target    AKLDFFKTSIQIDLEKYREQTEFGITSDKLLLAWREMEQAVELCGRENEVKHLVERMMALQTDIVDLQRSPMGRKQGGTL
4kik.1.B  AKLDFFKTSIQIDLEKYSEQTEFGITSDKLLLAWREMEQAVELCGRENEVKLLVERMMALQTDIVDLQRSPMGRKQGGTL

Target    DDLEEQARELYRRLREKPRDQRTDGDSQEMVRLLLQAIQGFEKKVRVIYTQLSKTVVCKQKALELLPKVEEVVSLMSEDE
4kik.1.B  DDLEEQARELYRRLREKPRDQRTEGDSQEMVRLLLQAIQSFEKKVRVIYTQLSKTVVCKQKALELLPKVEEVVSLMNEDE

Target    KMVVRLQEKRQKELWNLLKIACSKVRGPVSGSPDSMNASRLSHPCQLMSQPCTAPDSLPEAAEKSEDLVAEAHTLCTQLE
4kik.1.B  KTVVRLQEKRQKELWNLLKIACSK--------------------------------------------------------

Target    NALQDTMKEQDQSLRSLDWSWLQSEEEEQQSLERAS
4kik.1.B  ------------------------------------
```

Materials and Methods

Template Search

Template search with has been performed against the SWISS-MODEL template library (SMTL, last update: 2023-12-24, last included PDB release: 2023-12-15).

Model Building

Models are built based on the target-template alignment using ProMod3 (Studer et al.). Coordinates which are conserved between the target and the template are copied from the template to the model. Insertions and deletions are remodelled using a fragment library. Side chains are then rebuilt. Finally, the geometry of the resulting model is regularized by using a force field.

Model Quality Estimation

The global and per-residue model quality has been assessed using the QMEAN scoring function (Studer et al.).

Ligand Modelling

Ligands present in the template structure are transferred by homology to the model when the following criteria are met: (a) The ligands are annotated as biologically relevant in the template library, (b) the ligand is in contact with the model, (c) the ligand is not clashing with the protein, (d) the residues in contact with the ligand are conserved between the target and the template. If any of these four criteria is not satisfied, a certain ligand will not be included in the model. The model summary includes information on why and which ligand has not been included.

Oligomeric State Conservation

The quaternary structure annotation of the template is used to model the target sequence in its oligomeric form. The method (Bertoni et al.) is based on a supervised machine learning algorithm, Support Vector Machines (SVM), which combines interface conservation, structural clustering, and other template features to provide a quaternary structure quality estimate (QSQE). The QSQE score is a number between 0 and 1, reflecting the expected accuracy of the interchain contacts for a model built based a given alignment and template. Higher numbers indicate higher reliability. This complements the GMQE score which estimates the accuracy of the tertiary structure of the resulting model.

References

- **BLAST**
  Camacho, C., Coulouris, G., Avagyan, V., Ma, N., Papadopoulos, J., Bealer, K., Madden, T.L. BLAST+: architecture and applications. BMC Bioinformatics 10, 421-430 (2009).
- **HHblits**
  Steinegger, M., Meier, M., Mirdita, M., Vöhringer, H., Haunsberger, S. J., Söding, J. HH-suite3 for fast remote homology detection and deep protein annotation. BMC Bioinformatics 20, 473 (2019).

Table T1:

Primary amino acid sequence for which templates were searched and models were built.

```
MSWSPSLPTQTCGAWEMKERLGTGGFGNVIRWHNQETGEQIAIKQCRQELSPRNRERWCLEIQIMRRLNHPNVVAARDVPEGMQSLAPNDLPLLAMEYCQ
GGDLRKYLNQFENCCGLREGAILTLLSDIASALRYLHENRIIHRDLKPENIVLQQGEQRLIHKIIDLGYAKELDQGSLCTSFVGTLQYLAPELLEQQKYT
VTVDYWSFGTLAFECITGFRPFLPNWQPVQWHSKVRQKSEMDIVVSEDLNGAVKFSSSLPHPNNLNSVLAQRLEKWLQLMLMWHPRQRGTDPVYGPNGCF
KALDDILNLKLLHVLNMVTGTLHTYPVTEDESLQSLKARIRQDTGILEEDQELLQEAGLALIPDKPAAQCLSDGKLNEGRTLDMDLVFLFDNSRVTYESQ
VSPQPQPESVSCILQEPKRNLPFFQLRKVWGQVWHSIQALKEDCSRLQQGQRAAMMNLLRNNSCLSKMKNSMASMSQQLKAKLDFFKTSIQIDLEKYREQ
TEFGITSDKLLLAWREMEQAVELCGRENEVKHLVERMMALQTDIVDLQRSPMGRKQGGTLDDLEEQARELYRRLREKPRDQRTDGDSQEMVRLLLQAIQG
FEKKVRVIYTQLSKTVVCKQKALELLPKVEEVVSLMSEDEKMVVRLQEKRQKELWNLLKIACSKVRGPVSGSPDSMNASRLSHPCQLMSQPCTAPDSLPE
AAEKSEDLVAEAHTLCTQLENALQDTMKEQDQSLRSLDWSWLQSEEEEQQSLERAS
```

Table T2:

Template	Seq Identity	Oligo-state	QSQE	Found by	Method	Resolution	Seq Similarity	Coverage	Description
4kik.1.A	95.48	homo-dimer	0.66	HHblits	X-ray	2.83Å	0.60	0.88	Inhibitor of nuclear factor kappa-B kinase subunit beta
A0A673SRE9.1.A	94.84	monomer	-	AFDB search	AlphaFold v2	NA	0.60	1.00	IkappaB kinase
4kik.1.B	95.48	homo-dimer	0.68	HHblits	X-ray	2.83Å	0.60	0.88	Inhibitor of nuclear factor kappa-B kinase subunit beta
4e3c.3.A	95.30	homo-dimer	0.56	HHblits	X-ray	3.98Å	0.60	0.87	Inhibitor of nuclear factor kappa-B kinase subunit beta
3qa8.1.B	75.87	homo-tetramer	0.59	BLAST	X-ray	3.60Å	0.53	0.87	MGC80376 protein
3qa8.1.D	75.87	homo-tetramer	0.59	BLAST	X-ray	3.60Å	0.53	0.87	MGC80376 protein
3qa8.1.A	75.87	homo-tetramer	0.59	BLAST	X-ray	3.60Å	0.53	0.87	MGC80376 protein
3qa8.1.B	75.87	homo-tetramer	0.59	HHblits	X-ray	3.60Å	0.53	0.87	MGC80376 protein
3qa8.1.D	75.87	homo-tetramer	0.59	HHblits	X-ray	3.60Å	0.53	0.87	MGC80376 protein

Template	Seq Identity	Oligo-state	QSQE	Found by	Method	Resolution	Seq Similarity	Coverage	Description
3qa8.1.A	75.87	homo-tetramer	0.59	HHblits	X-ray	3.60Å	0.53	0.87	MGC80376 protein
4e3c.2.A	95.30	homo-dimer	0.54	HHblits	X-ray	3.98Å	0.60	0.87	Inhibitor of nuclear factor kappa-B kinase subunit beta
4e3c.3.B	95.30	homo-dimer	0.56	HHblits	X-ray	3.98Å	0.60	0.87	Inhibitor of nuclear factor kappa-B kinase subunit beta
4e3c.2.B	95.30	homo-dimer	0.54	HHblits	X-ray	3.98Å	0.60	0.87	Inhibitor of nuclear factor kappa-B kinase subunit beta
4e3c.1.A	95.30	homo-dimer	0.53	HHblits	X-ray	3.98Å	0.60	0.87	Inhibitor of nuclear factor kappa-B kinase subunit beta
4e3c.1.B	95.30	homo-dimer	0.53	HHblits	X-ray	3.98Å	0.60	0.87	Inhibitor of nuclear factor kappa-B kinase subunit beta
3qa8.2.B	75.87	homo-tetramer	0.54	HHblits	X-ray	3.60Å	0.53	0.87	MGC80376 protein
3rzf.1.A	75.87	monomer	-	HHblits	X-ray	4.00Å	0.53	0.87	MGC80376 protein
4im0.1.A	28.61	homo-dimer	0.28	HHblits	X-ray	2.40Å	0.34	0.47	Serine/threonine-protein kinase TBK1
6rst.1.A	28.98	homo-dimer	0.22	HHblits	X-ray	3.29Å	0.34	0.47	Serine/threonine-protein kinase TBK1
6rsr.1.A	28.98	homo-dimer	0.17	HHblits	X-ray	3.15Å	0.34	0.47	Serine/threonine-protein kinase TBK1
4im0.1.A	29.06	homo-dimer	0.25	BLAST	X-ray	2.40Å	0.35	0.46	Serine/threonine-protein kinase TBK1
4im3.1.A	28.61	homo-dimer	0.18	HHblits	X-ray	3.34Å	0.34	0.47	Serine/threonine-protein kinase TBK1
6rst.1.A	30.03	homo-dimer	0.19	BLAST	X-ray	3.29Å	0.35	0.45	Serine/threonine-protein kinase TBK1
4im3.1.A	29.06	homo-dimer	0.17	BLAST	X-ray	3.34Å	0.35	0.46	Serine/threonine-protein kinase TBK1
6rsr.1.A	30.03	homo-dimer	0.16	BLAST	X-ray	3.15Å	0.35	0.45	Serine/threonine-protein kinase TBK1
5w5v.1.A	28.90	homo-dimer	0.11	HHblits	X-ray	3.64Å	0.34	0.47	Serine/threonine-protein kinase TBK1
5w5v.1.A	29.34	homo-dimer	0.08	BLAST	X-ray	3.64Å	0.35	0.46	Serine/threonine-protein kinase TBK1
6bny.1.A	28.90	monomer	-	HHblits	X-ray	3.34Å	0.34	0.47	Serine/threonine-protein kinase TBK1
6bod.1.A	28.90	monomer	-	HHblits	X-ray	3.20Å	0.34	0.47	Serine/threonine-protein kinase TBK1
6boe.1.A	28.90	monomer	-	HHblits	X-ray	3.60Å	0.34	0.47	Serine/threonine-protein kinase TBK1
6bny.1.A	29.34	monomer	-	BLAST	X-ray	3.34Å	0.35	0.46	Serine/threonine-protein kinase TBK1
6bod.1.A	29.34	monomer	-	BLAST	X-ray	3.20Å	0.35	0.46	Serine/threonine-protein kinase TBK1
6boe.1.A	29.34	monomer	-	BLAST	X-ray	3.60Å	0.35	0.46	Serine/threonine-protein kinase TBK1
6ckx.2.A	24.57	monomer	-	HHblits	X-ray	2.80Å	0.31	0.39	Cyclin-dependent kinase 12
7nxk.2.A	24.57	monomer	-	HHblits	X-ray	3.00Å	0.31	0.39	Cyclin-dependent kinase 12
4nst.3.A	24.57	monomer	-	HHblits	X-ray	2.20Å	0.31	0.39	Cyclin-dependent kinase 12

Template	Seq Identity	Oligo-state	QSQE	Found by	Method	Resolution	Seq Similarity	Coverage	Description
4n4s.1.A	22.34	monomer	-	HHblits	X-ray	2.20Å	0.31	0.38	Mitogen-activated protein kinase 1
6zr5.1.A	24.14	monomer	-	HHblits	X-ray	2.70Å	0.32	0.38	Mitogen-activated protein kinase 8
8p81.1.A	24.57	monomer	-	HHblits	X-ray	2.68Å	0.31	0.39	Cyclin-dependent kinase 12
7nxk.1.A	24.57	monomer	-	HHblits	X-ray	3.00Å	0.31	0.39	Cyclin-dependent kinase 12
3vum.1.A	24.22	monomer	-	HHblits	X-ray	2.69Å	0.32	0.38	Mitogen-activated protein kinase 8
4i5h.1.A	22.34	monomer	-	HHblits	X-ray	1.90Å	0.31	0.38	Mitogen-activated protein kinase 1
5acb.2.B	23.81	monomer	-	HHblits	X-ray	2.70Å	0.31	0.39	CYCLIN-DEPENDENT KINASE 12
3vuh.1.A	24.91	monomer	-	HHblits	X-ray	2.70Å	0.32	0.38	Mitogen-activated protein kinase 8
4qtd.1.A	23.71	monomer	-	HHblits	X-ray	1.50Å	0.31	0.38	Mitogen-activated protein kinase 8
6zr5.2.A	24.14	monomer	-	HHblits	X-ray	2.70Å	0.32	0.38	Mitogen-activated protein kinase 8
4cxa.1.A	23.81	monomer	-	HHblits	X-ray	3.15Å	0.31	0.39	CYCLIN-DEPENDENT KINASE 12
5acb.1.B	23.81	monomer	-	HHblits	X-ray	2.70Å	0.31	0.39	CYCLIN-DEPENDENT KINASE 12
3vug.1.A	24.57	monomer	-	HHblits	X-ray	3.24Å	0.32	0.38	Mitogen-activated protein kinase 8
3vuk.1.A	24.57	monomer	-	HHblits	X-ray	2.95Å	0.32	0.38	Mitogen-activated protein kinase 8

The table above shows the top 50 filtered templates. A further 5,594 templates were found which were considered to be less suitable for modelling than the filtered list.
1a06.1.A, 1a9u.1.A, 1ad5.1.A, 1apm.1.A, 1aq1.1.A, 1b39.1.A, 1b6c.5.D, 1bi7.1.A, 1bi8.1.A, 1bkx.1.A, 1bl7.1.A, 1blx.1.A, 1bmk.1.A, 1buh.1.A, 1bx6.1.A, 1byg.1.A, 1cdk.1.A, 1ckj.1.A, 1ckj.1.B, 1cm8.1.A, 1cmk.1.A, 1csn.1.A, 1ctp.1.A, 1di8.1.A, 1di9.1.A, 1dow.1.A, 1ds5.1.A, 1e9h.1.A, 1eh4.1.A, 1eh4.2.A, 1eq1.1.A, 1f0q.1.A, 1f3m.1.D, 1f5q.1.A, 1f5q.2.A, 1fgi.1.A, 1fin.1.A, 1fin.2.A, 1fmk.1.A, 1fot.1.A, 1fpu.1.A, 1fq1.1.B, 1fvr.1.A, 1fvv.1.A, 1g3n.4.C, 1g5s.1.A, 1gih.1.A, 1gij.1.A, 1gjo.1.A, 1gng.1.A, 1gol.1.A, 1gzk.1.A, 1gzn.1.A, 1h01.1.A, 1h1p.2.A, 1h1q.1.A, 1h1q.2.A, 1h1w.1.A, 1h25.2.A, 1h27.1.A, 1h28.2.A, 1h4l.1.A, 1h8f.1.A, 1hcl.1.A, 1how.1.A, 1i09.1.A, 1i44.1.A, 1ia8.1.A, 1ias.1.A, 1ias.1.B, 1ir3.1.C, 1irk.1.A, 1j1b.1.A, 1j1b.1.B, 1j3h.1.A, 1j7i.1.A, 1j7u.1.A, 1j7u.1.B, 1j91.1.A, 1j91.2.A, 1jbp.1.A, 1jks.1.A, 1jkt.1.A, 1jkt.2.A, 1jnk.1.A, 1jow.1.B, 1jpa.1.A, 1jqh.2.A, 1jst.1.A, 1jst.1.C, 1jsu.1.A, 1jvp.1.A, 1jwh.1.A, 1jwh.1.B, 1k2p.1.A, 1k2p.1.B, 1k3a.1.A, 1k9a.1.A, 1k9a.2.A, 1k9a.3.A, 1k9a.5.A, 1k9a.6.A, 1koa.1.A, 1ksw.1.A, 1kv1.1.A, 1kwp.3.B, 1kwp.3.C, 1l8t.1.A, 1lew.1.A, 1lp4.1.A, 1lq7.1.A, 1luf.1.A, 1m14.1.A, 1m17.1.A, 1m2p.1.A, 1m2q.1.A, 1m2r.1.A, 1m52.1.A, 1m7n.1.A, 1mp8.1.A, 1mq4.1.A, 1mqb.1.A, 1mqb.2.A, 1mru.1.A, 1mru.2.A, 1mrv.1.A, 1muo.1.A, 1na7.1.A, 1nd4.1.A, 1nw1.1.A, 1nxk.1.A, 1ny3.2.C, 1o6k.1.A, 1o6l.1.A, 1o6y.1.A, 1o9u.1.A, 1oi9.2.A, 1oit.1.A, 1okv.2.A, 1oky.1.A, 1ol2.1.A, 1ol5.1.A, 1ol6.1.A, 1ol7.1.A, 1omw.1.A, 1opk.1.A, 1opl.1.A, 1opl.2.A, 1ouk.1.A, 1oz1.1.A, 1p14.1.A, 1p2a.1.A, 1p4f.1.A, 1p4o.1.A, 1p5e.1.A, 1pf8.1.A, 1phk.1.A, 1pjk.1.A, 1pkg.1.A, 1pkg.2.A, 1pme.1.A, 1pmn.1.A, 1pmu.1.A, 1pmv.1.A, 1pxo.1.A, 1py5.1.A, 1pyx.1.A, 1pyx.1.B, 1q24.1.A, 1q5k.1.A, 1q5k.1.B, 1q8w.1.A, 1q8y.1.A, 1q8y.2.A, 1qcf.1.A, 1ql6.1.A, 1qmz.1.A, 1qpd.1.A, 1qpe.1.A, 1r0e.1.A, 1r0p.1.A, 1r39.1.A, 1r3c.1.A, 1r78.1.A, 1rdq.1.A, 1re8.1.A, 1rek.1.A, 1rjb.1.A, 1rkc.1.A, 1rke.1.A, 1rqq.1.A, 1rw8.1.A, 1s9i.1.A, 1s9i.1.B, 1s9j.1.A, 1sm2.1.A, 1sm2.2.A, 1smh.1.A, 1snu.2.A, 1stc.1.A, 1syk.1.A, 1syq.1.A, 1szm.1.A, 1szm.2.A, 1t01.1.A, 1t45.1.A, 1t46.1.A, 1tki.1.A, 1tqi.1.A, 1tvo.1.A, 1u46.1.A, 1u46.2.A, 1u54.1.A, 1u54.2.A, 1u59.1.A, 1u5q.1.A, 1u5q.2.A, 1u5r.2.A, 1u5r.3.B, 1ua2.1.A, 1ua2.2.A, 1ua2.3.A, 1ua2.4.A, 1ukh.1.A, 1uki.1.A, 1ung.2.A, 1unh.1.A, 1uu7.1.A, 1uu9.1.A, 1uv5.1.A, 1uwh.1.A, 1uwj.1.A, 1v0b.1.A, 1v0o.1.A, 1v0o.2.A, 1v0p.1.A, 1v0p.2.A, 1v1k.1.A, 1vjy.1.A, 1vr2.1.A, 1vyw.1.A, 1vyw.2.A, 1vzo.1.A, 1w7h.1.A, 1w82.1.A, 1w98.1.A, 1wak.1.A, 1wbo.1.A, 1wbv.1.A, 1wbw.1.A, 1wmk.1.A, 1wvw.1.A, 1wvx.1.A, 1wvy.1.A, 1wzy.1.A, 1x8b.1.A, 1xh4.1.A, 1xh6.1.A, 1xh9.1.A, 1xjd.1.A, 1xkk.1.A, 1xr1.1.A, 1xwj.1.A, 1xws.1.A, 1y57.1.A, 1y6a.1.A, 1y8g.1.A, 1ydi.1.A, 1ydt.1.A, 1yhv.1.A, 1yhw.1.A, 1yi3.1.A, 1yi4.1.A, 1yi6.1.A, 1yi6.2.A, 1ym7.2.A, 1yoj.1.A, 1yol.1.A, 1yol.2.A, 1yom.2.A, 1yqj.1.A, 1yrp.1.A, 1yvj.1.A, 1yw2.1.A, 1ywn.1.A, 1ywr.1.A, 1yxs.1.A, 1yxu.1.A, 1yzm.1.A, 1z0k.1.B, 1z0k.2.B, 1z9x.1.A, 1zlt.1.A, 1zmu.1.A, 1zmv.1.A, 1zoe.1.A, 1zog.1.A, 1zp9.1.A, 1zp9.3.A, 1zp9.4.A, 1zrz.1.A, 1ztf.1.A, 1zvz.1.A, 1zw2.1.A, 1zw3.1.A, 1zws.1.A, 1zxe.1.A, 1zxe.1.B, 1zxe.3.A, 1zxe.3.B, 1zy5.1.A, 1zy5.1.B, 1zyc.1.A, 1zyc.1.B, 1zyc.2.A, 1zyc.2.B, 1zyd.1.A, 1zyj.1.A, 1zyl.1.A, 1zys.1.A, 1zz2.1.A, 2a0c.1.A, 2a19.1.B, 2a19.1.C, 2a1a.1.B, 2a27.1.A, 2a2a.1.A, 2ac3.1.A, 2ac5.1.A, 2acx.1.A, 2auh.1.A, 2ayp.1.A, 2b0q.1.A, 2b1p.1.A, 2b4s.1.B, 2b52.1.A, 2b53.1.A, 2b54.1.A, 2b7a.1.A,

2b7a.2.A, 2b9f.1.A, 2b9h.1.A, 2baj.1.A, 2bak.1.A, 2bal.1.A, 2baq.1.A, 2bcj.1.A, 2bdf.1.A, 2bdw.1.A, 2bdw.1.B, 2bfy.1.A, 2bfy.2.A, 2biy.1.A, 2bkk.1.A, 2bkk.2.A, 2bmc.1.A, 2bts.1.A, 2bva.1.A, 2bva.2.A, 2c1b.1.A, 2c30.1.A, 2c3j.1.A, 2c47.1.A, 2c6d.1.A, 2c6e.1.A, 2c6e.2.A, 2cch.1.A, 2cdz.1.A, 2cgw.1.A, 2chl.1.A, 2cjm.2.A, 2cko.1.A, 2ckp.1.A, 2ckp.1.B, 2ckq.1.A, 2clq.1.A, 2cmw.1.A, 2cn5.1.A, 2cn8.1.B, 2cpk.1.A, 2csn.1.A, 2dq7.1.A, 2dwb.1.A, 2dyl.1.A, 2dzm.1.A, 2e2b.1.A, 2e9p.1.A, 2e9v.1.A, 2eb2.1.A, 2eb3.1.A, 2efr.1.A, 2efr.1.B, 2efs.1.A, 2efs.1.B, 2erk.1.A, 2erz.1.A, 2etm.1.A, 2etm.2.A, 2eu9.1.A, 2euf.1.B, 2eva.1.A, 2ewa.1.A, 2exc.1.A, 2exe.1.A, 2exm.1.A, 2f2c.1.B, 2f2u.1.A, 2f2u.2.A, 2f49.3.A, 2f4j.1.A, 2f57.1.A, 2f57.2.A, 2fb8.1.A, 2fh9.1.A, 2fo0.1.A, 2fsl.1.A, 2fso.1.A, 2fst.1.A, 2fum.1.A, 2fum.3.A, 2fvd.1.A, 2fys.1.A, 2fys.2.A, 2g01.1.A, 2g01.2.A, 2g15.1.A, 2g1t.4.A, 2g2f.1.A, 2g2f.2.A, 2g2h.1.A, 2g2h.2.A, 2g2i.1.A, 2g2i.2.A, 2gcd.1.A, 2gcd.2.A, 2gdo.1.A, 2gfc.1.A, 2gfs.1.A, 2ghl.1.A, 2ghm.1.A, 2gmx.1.A, 2gmx.2.A, 2gnf.1.A, 2gnj.1.A, 2gph.1.A, 2gqg.1.A, 2gqg.2.A, 2gs6.1.C, 2gs7.1.A, 2gsf.1.A, 2gtm.1.A, 2gu8.1.A, 2gww.1.A, 2h34.1.A, 2h34.2.A, 2h6d.1.A, 2h8h.1.A, 2h96.1.A, 2h96.2.A, 2h9v.1.A, 2hak.1.A, 2hak.2.A, 2hak.3.A, 2hak.6.A, 2hak.7.A, 2hck.1.B, 2hel.1.A, 2hen.1.A, 2hiw.1.A, 2hiw.2.A, 2hk5.1.A, 2hog.1.A, 2hsq.1.A, 2hw6.1.A, 2hw6.2.A, 2hw7.1.A, 2hwo.1.A, 2hwp.2.A, 2hxq.1.A, 2hyy.3.A, 2hz0.1.A, 2hz0.2.A, 2hz4.1.A, 2hz4.2.A, 2hz4.3.A, 2hz8.1.A, 2hzi.1.A, 2hzi.2.A, 2hzn.1.A, 2i0e.1.A, 2i0v.1.A, 2i0y.1.A, 2i1m.1.A, 2i40.2.A, 2i7q.1.A, 2ibf.1.A, 2ig7.1.A, 2iiu.1.A, 2ijm.1.A, 2in6.1.A, 2itn.1.A, 2itp.1.A, 2itx.1.A, 2itz.1.A, 2ivu.1.A, 2ivv.1.A, 2iw6.1.A, 2iw8.1.A, 2iwi.1.A, 2izs.1.A, 2j0i.1.A, 2j0j.1.A, 2j0k.1.A, 2j0k.2.A, 2j0l.1.A, 2j0m.1.B, 2j2i.1.A, 2j4z.1.A, 2j4z.2.A, 2j50.1.A, 2j5e.1.A, 2j5f.1.A, 2j90.1.A, 2j90.1.B, 2j9m.1.A, 2jam.1.A, 2jav.1.A, 2jbo.1.A, 2jbp.1.A, 2jbp.10.A, 2jc6.1.A, 2jed.1.A, 2jed.2.A, 2jfm.1.B, 2jgz.1.A, 2jii.1.A, 2jit.1.A, 2jit.2.A, 2jiu.1.A, 2jiu.2.A, 2jiv.1.A, 2jiv.2.A, 2jkm.1.A, 2jko.1.A, 2jkq.1.A, 2kty.1.A, 2kul.1.A, 2lav.1.A, 2lgc.1.A, 2lxy.1.A, 2mi7.1.A, 2no3.2.A, 2np8.1.A, 2npq.1.A, 2nru.1.A, 2nru.2.A, 2nru.3.A, 2nru.4.A, 2nry.1.A, 2nry.2.A, 2nry.3.A, 2nry.4.A, 2o5k.1.A, 2o8y.1.A, 2ofu.1.A, 2ofv.1.A, 2ofv.1.B, 2og8.1.A, 2ogv.1.A, 2oh4.1.A, 2oib.1.A, 2oib.2.A, 2oib.3.A, 2oic.2.A, 2oic.3.A, 2oid.1.A, 2oid.2.A, 2oid.3.A, 2oid.4.A, 2oiq.1.A, 2oj9.1.A, 2ojf.1.A, 2ojg.1.A, 2ojj.1.A, 2ok1.1.A, 2onl.1.B, 2onl.2.A, 2onl.2.B, 2ow3.1.A, 2owb.2.B, 2oxy.1.A, 2oza.1.A, 2oza.1.B, 2ozo.1.A, 2p2h.1.A, 2p2i.1.A, 2p3g.1.A, 2p4i.1.A, 2p4i.2.A, 2p55.1.B, 2pe2.1.A, 2phk.1.A, 2pih.1.A, 2pk9.2.A, 2pl0.1.A, 2pml.1.A, 2pmo.1.A, 2ppq.1.A, 2psq.1.A, 2ptk.1.A, 2pup.1.A, 2puu.1.A, 2pvf.1.A, 2pvj.1.A, 2pvy.1.B, 2pwl.1.A, 2py3.1.A, 2pyw.1.A, 2pz5.1.A, 2pzi.1.A, 2pzi.2.A, 2pzp.1.A, 2pzr.1.A, 2pzy.1.A, 2pzy.2.A, 2pzy.3.A, 2pzy.4.A, 2q0b.1.A, 2q0n.1.A, 2q83.1.A, 2qd9.1.A, 2qg5.1.A, 2qg5.2.A, 2qg7.1.A, 2qg7.1.B, 2qg7.2.A, 2qg7.2.B, 2qhm.1.A, 2qi8.1.A, 2qkr.1.A, 2qkw.1.B, 2qlq.1.A, 2qlq.2.A, 2qlu.1.A, 2qnj.1.A, 2qnj.1.B, 2qoc.1.A, 2qod.1.A, 2qof.1.A, 2qoh.1.A, 2qoi.1.A, 2qok.1.A, 2qol.1.A, 2qon.1.A, 2qoo.1.A, 2qu5.1.A, 2qu6.1.A, 2qur.1.A, 2qvs.1.A, 2r0i.1.A, 2r2p.1.A, 2r3n.1.A, 2r4b.1.A, 2r64.1.A, 2r7i.1.A, 2r9s.1.A, 2rei.1.A, 2rf9.1.A, 2rf9.2.A, 2rfd.1.A, 2rfd.2.A, 2rfe.2.A, 2rfe.4.A, 2rfn.1.A, 2rfs.1.A, 2rg5.1.A, 2rg6.1.A, 2rgp.1.A, 2rio.1.A, 2rku.1.A, 2rl5.1.A, 2rsv.1.A, 2src.1.A, 2uzb.2.A, 2uzd.1.A, 2uzl.1.A, 2uzv.1.B, 2v55.1.A, 2v5q.1.A, 2v5q.2.A, 2v62.1.A, 2v71.1.A, 2v71.1.B, 2v7a.1.A, 2vd5.1.A, 2vn9.1.A, 2vo6.1.A, 2vrx.1.A, 2vrx.2.A, 2vth.1.A, 2vtn.1.A, 2vtt.1.A, 2vuw.1.A, 2vwb.1.A, 2vwb.2.A, 2vwi.1.A, 2vwi.1.B, 2vwi.1.C, 2vwi.1.D, 2vwx.1.A, 2vx3.4.A, 2vz6.1.A, 2w1g.1.A, 2w1i.1.A, 2w1z.1.A, 2w4j.1.A, 2w4k.1.A, 2w4o.1.A, 2w5a.1.A, 2w5h.1.A, 2w7x.1.B, 2w96.1.B, 2w99.1.B, 2w9f.1.B, 2w9z.1.B, 2wb8.1.A, 2wd1.1.A, 2wei.1.A, 2wel.1.A, 2wma.1.A, 2wma.2.A, 2wmb.2.A, 2wnt.1.A, 2wo6.2.A, 2wot.1.A, 2wqb.1.A, 2wqe.1.A, 2wqn.1.A, 2wqo.1.A, 2wtk.1.B, 2wtk.1.C, 2wtk.2.C, 2wtv.1.A, 2wtv.2.A, 2wtw.1.A, 2wu6.1.A, 2wzj.1.A, 2x0g.1.A, 2x39.1.A, 2x4f.1.A, 2x4z.1.A, 2x6e.1.A, 2x7f.1.A, 2x7o.3.A, 2x8e.1.A, 2x8i.1.A, 2x9e.1.A, 2xa4.1.A, 2xba.1.A, 2xck.1.A, 2xik.1.A, 2xir.1.A, 2xj0.1.A, 2xk9.1.A, 2xm8.1.B, 2xne.1.A, 2xng.1.A, 2xnm.1.A, 2xru.1.A, 2xrw.1.A, 2xs0.1.A, 2xuu.1.A, 2xyn.3.A, 2xyu.1.A, 2xzs.1.A, 2y0a.1.A, 2y4i.1.A, 2y4i.1.B, 2y4p.1.B, 2y4p.2.A, 2y6m.1.A, 2y7j.1.A, 2y7j.1.B, 2y8o.1.A, 2ya9.1.A, 2yak.1.A, 2ycf.1.A, 2ycr.1.A, 2yex.1.A, 2yfx.1.A, 2yhv.1.A, 2yiw.1.A, 2yix.1.A, 2yiy.1.A, 2yjr.1.A, 2yjs.1.A, 2yn8.1.A, 2ywp.1.A, 2yza.1.A, 2z2w.2.A, 2z60.1.A, 2z7l.1.A, 2z7q.1.A, 2z8c.1.A, 2zb1.1.A, 2zdt.1.A, 2zdu.1.A, 2zjw.1.A, 2zm1.1.A, 2zm3.2.A, 2zm3.3.A, 2zmc.1.A, 2zmd.1.A, 2zoq.1.A, 2zoq.2.A, 2zv7.1.A, 3a2c.1.D, 3a2c.1.F, 3a2c.1.G, 3a2c.1.H, 3a2c.1.I, 3a2c.1.L, 3a4o.1.A, 3a4p.1.A, 3a60.1.A, 3a60.1.B, 3a61.1.A, 3a62.1.A, 3a7f.1.A, 3a7g.1.A, 3a7g.2.A, 3a7i.1.A, 3a8w.1.A, 3a8x.1.A, 3a99.1.A, 3ag9.1.A, 3ag9.3.B, 3agl.1.A, 3aln.1.A, 3aln.2.A, 3aln.3.A, 3alo.1.A, 3ama.1.A, 3amy.1.A, 3anr.1.A, 3aox.1.A, 3aqv.1.A, 3at4.1.A, 3ats.1.A, 3axw.1.A, 3b2t.1.A, 3b2w.1.A, 3b8r.1.A, 3b8r.2.A, 3bbt.1.A, 3bce.1.A, 3be2.1.A, 3bea.1.A, 3beg.1.A, 3bgq.1.A, 3bhh.1.A, 3bhh.3.A, 3bht.1.A, 3bht.2.A, 3bhy.1.A, 3bi6.1.A, 3bkb.1.A, 3blq.1.A, 3blr.1.A, 3bpr.4.A, 3bqr.1.B, 3brb.1.A, 3brb.2.A, 3brt.1.A, 3brt.1.C, 3brv.1.A, 3brv.1.C, 3bu3.1.A, 3bv2.1.A, 3bv3.1.A, 3bx5.1.A, 3bym.1.A, 3bys.1.A, 3byv.1.A, 3bz3.1.A, 3c0g.1.A, 3c0i.1.A, 3c1x.1.A, 3c4c.1.A, 3c4c.2.A, 3c4d.1.A, 3c4d.2.A, 3c4e.1.A, 3c4f.1.A, 3c4x.1.A, 3c4y.1.A, 3c4y.1.B, 3c4z.1.A, 3c50.1.B, 3c51.1.A, 3c51.1.B, 3c5i.1.A, 3c5u.1.A, 3c7q.1.A, 3c9w.1.A, 3cbl.1.A, 3cc6.1.A, 3ce3.1.A, 3cik.1.A, 3cjf.1.A, 3cjg.1.A, 3ckw.1.A, 3cly.1.A, 3coh.1.A, 3coi.1.A, 3cok.1.A, 3com.1.A, 3com.2.A, 3cp9.2.A, 3cqu.1.A, 3cs9.1.A, 3cs9.4.A, 3csv.1.A, 3cth.1.A, 3ctj.1.A, 3ctq.1.A, 3cxw.1.A, 3d0e.1.A, 3d14.1.A, 3d2k.1.A, 3d5u.1.A, 3d5v.1.A, 3d5x.1.A, 3d7t.1.A, 3d7t.2.B, 3d7u.1.A, 3d7u.1.B, 3d94.1.A, 3d9v.1.A, 3d9v.1.B, 3da6.1.A, 3dae.1.A, 3daj.1.A, 3dak.1.A, 3dak.1.C, 3dbq.1.A, 3dcv.1.A, 3ddp.1.A, 3ddq.1.A, 3dfa.1.A, 3dfc.1.A, 3dj5.1.A, 3dk3.1.A, 3dk3.2.A, 3dk6.1.A, 3dk6.2.A, 3dk7.1.A, 3dk7.2.A, 3dkc.1.A, 3dkf.1.A, 3dkg.1.A, 3dko.1.A, 3dls.1.A, 3dlz.1.A, 3dnd.1.A, 3dqw.1.A, 3dqw.3.A, 3dqx.1.A, 3dqx.2.A, 3ds6.1.A, 3ds6.2.A, 3ds6.3.A, 3ds6.4.A, 3dt1.1.A, 3dtc.1.A, 3du8.2.A, 3dv3.1.A, 3dxn.1.A, 3dxp.1.A, 3dxq.1.A, 3dy7.1.A, 3dzo.1.A, 3dzq.1.A, 3e3b.2.B, 3e3p.1.A, 3e5a.1.A, 3e64.1.A, 3e7o.1.A, 3e7o.1.B, 3e8c.2.A, 3e92.1.A, 3e93.1.A, 3eb0.1.A, 3efl.2.A, 3ej1.1.A, 3ekk.1.A, 3el7.1.A, 3el8.1.A, 3emg.1.A, 3en7.1.A, 3en9.1.A, 3en9.2.A, 3enh.1.A, 3enm.1.A, 3enm.1.B, 3enm.2.A, 3eqc.1.A, 3eqp.1.A, 3eqr.1.A, 3erk.1.A, 3eta.1.A, 3eyg.1.A, 3ezv.1.A, 3f2a.1.A, 3f3w.1.A, 3f3z.1.A, 3f5p.17.F, 3f5u.1.A, 3f61.1.A, 3f66.1.A, 3f66.2.A, 3f69.1.A, 3f6x.3.A, 3f7w.1.A, 3f7z.1.A, 3f82.1.A, 3f88.1.A, 3faa.1.A, 3faa.3.A, 3fc1.1.A, 3fe3.1.A, 3fe3.1.B, 3feg.1.A, 3fhi.1.A, 3fhr.1.A, 3fi2.1.A, 3fi3.1.A, 3fi8.1.A, 3fko.1.A, 3fl4.1.A, 3fln.1.A, 3fls.1.A, 3fme.1.A, 3fpm.1.A, 3fsk.1.A, 3fv8.1.A, 3fwq.1.A, 3fwq.2.A, 3fy0.1.A, 3fy2.1.A, 3fzo.1.A, 3fzr.1.A, 3fzt.1.A, 3g0e.1.A, 3g0f.1.A, 3g0f.1.B, 3g2f.1.A, 3g2f.2.A, 3g33.1.A, 3g33.1.C, 3g51.1.A, 3g6h.1.A, 3g6h.2.A, 3g90.1.A, 3gb2.1.A, 3gbz.1.A, 3gc7.1.A, 3gc8.1.A, 3gc8.2.A, 3gc9.1.A, 3gc9.2.A, 3gcp.1.A, 3gcq.1.A, 3gcu.1.B, 3gcv.1.A, 3geq.1.A, 3gfe.1.A, 3ggf.2.A, 3gi3.1.A, 3gni.1.B, 3gok.1.A, 3gop.1.A, 3gp0.1.A, 3gqi.1.A, 3gql.1.A, 3gql.2.A, 3gql.3.A, 3gt8.1.A, 3gt8.1.B, 3gt8.2.A, 3gt8.2.B, 3gu4.1.A, 3gu8.1.A, 3gub.1.A, 3gvu.2.A, 3h0y.2.A, 3h0z.1.A, 3h0z.2.A, 3h0z.3.A, 3h10.1.A, 3h10.3.A, 3h30.1.A, 3h3c.1.A, 3h4j.1.A, 3h4j.2.A, 3h9o.1.A, 3ha6.1.A, 3ha8.1.A, 3ham.1.A, 3ham.1.B, 3hav.1.A, 3hav.2.A, 3hav.3.A, 3hdn.1.A, 3hec.1.A, 3heg.1.A, 3hgk.1.A, 3hko.1.A, 3hll.1.A, 3hmi.1.A, 3hmn.1.A, 3hng.1.A, 3hnw.1.A, 3hnw.1.B, 3hp2.1.A, 3hub.1.A, 3huc.1.A, 3hv4.1.A, 3hv6.1.A, 3hvc.1.A, 3hx4.1.A, 3hyh.1.A, 3hyh.2.A, 3hzt.1.A, 3i0o.1.A, 3i0q.1.A, 3i1a.1.A, 3i1a.2.A, 3i4b.1.A, 3i4b.1.B, 3i5z.1.A, 3i6u.1.A, 3i6u.1.B, 3i6w.1.A, 3i6w.1.B, 3i6w.2.A, 3i6w.2.B, 3i6w.3.A, 3i6w.3.B, 3i79.1.A, 3i7b.1.A, 3i81.1.A, 3idb.1.A, 3idp.1.A, 3iec.1.A, 3igo.1.A, 3ii5.1.A, 3ik3.1.A, 3ik3.2.A, 3ika.1.A, 3ika.3.B, 3iph.1.A, 3is5.1.A, 3is5.1.F, 3iw4.1.A, 3iw4.2.A, 3iw6.1.A, 3j4r.1.D, 3jr1.1.A, 3js2.2.A, 3juh.1.A,

3jvr.1.A, 3jy0.1.A, 3jy9.1.A, 3jya.1.A, 3k3i.1.A, 3k3j.1.A, 3k54.1.A, 3k5u.1.A, 3ka0.1.A, 3kb7.1.A, 3kc3.1.A, 3kc3.1.B, 3kc3.1.C, 3kc3.2.A, 3kc3.2.C, 3kc3.3.B, 3kc3.3.C, 3kc3.4.A, 3kc3.4.B, 3kc3.4.C, 3kcf.3.A, 3kcf.4.A, 3kcf.5.A, 3kck.1.A, 3kex.1.A, 3kf4.2.A, 3kfa.2.A, 3kk8.1.A, 3kk9.1.A, 3kl8.1.A, 3kmm.1.A, 3kmu.1.A, 3kn5.1.A, 3kn5.2.A, 3kq7.1.A, 3krj.1.A, 3krl.1.A, 3krr.1.A, 3krw.1.A, 3ku2.1.A, 3kul.1.A, 3kvx.1.A, 3kxn.1.A, 3kxx.1.A, 3kxx.3.A, 3kxx.4.A, 3kxz.1.A, 3ky2.1.A, 3ky2.2.A, 3l39.1.A, 3l8p.1.A, 3l8s.1.A, 3l9l.1.A, 3l9m.1.A, 3l9p.1.A, 3lcd.1.A, 3lck.1.A, 3lco.1.A, 3lct.1.A, 3lfb.1.A, 3lfd.1.A, 3lff.1.A, 3lfs.1.A, 3lij.1.A, 3lj1.1.B, 3llt.1.A, 3lmg.1.A, 3lok.1.A, 3lpb.1.A, 3lq3.1.B, 3lq5.1.A, 3lq8.1.A, 3lvp.1.A, 3lvp.2.A, 3lvp.3.A, 3lvp.4.A, 3lw0.1.A, 3lxk.1.A, 3lxn.1.A, 3lxp.1.A, 3lzb.1.A, 3m1s.1.B, 3m2w.1.A, 3ma6.1.A, 3ma6.2.A, 3mbl.1.A, 3mdy.1.A, 3mes.1.A, 3mfr.1.A, 3mh0.1.A, 3mh1.1.A, 3mh2.1.A, 3mh3.1.A, 3mi9.1.A, 3mj1.1.A, 3mn3.1.A, 3mpa.1.A, 3mpm.1.A, 3mpt.1.A, 3ms9.1.A, 3mtl.2.B, 3mv5.1.A, 3mvj.1.A, 3mvj.2.A, 3mvj.3.A, 3mvl.1.A, 3mvl.2.A, 3mvm.1.A, 3mvm.2.A, 3mw1.1.A, 3my0.1.A, 3myg.1.A, 3n4t.1.A, 3n4u.1.A, 3n4v.1.A, 3n9x.1.A, 3nax.1.A, 3nay.1.A, 3ncg.1.A, 3ncz.1.A, 3ncz.1.B, 3ncz.2.A, 3ncz.2.B, 3ndm.1.A, 3new.1.A, 3nga.1.A, 3nie.1.A, 3niz.1.A, 3nlb.1.A, 3nnu.1.A, 3nnx.1.A, 3npc.1.A, 3nr9.1.B, 3nrm.1.A, 3nsz.1.A, 3nun.1.A, 3nus.1.A, 3nw5.1.A, 3nw6.1.A, 3nw7.1.A, 3nx8.1.A, 3nyn.1.A, 3nyx.1.A, 3nz0.1.A, 3o0g.1.A, 3o0g.2.A, 3o17.1.A, 3o17.2.A, 3o23.1.A, 3o2m.1.A, 3o50.1.A, 3o50.2.A, 3o51.1.A, 3o71.1.A, 3o7l.1.A, 3o7l.1.B, 3o8p.1.A, 3o8t.1.A, 3o8u.1.A, 3o96.1.A, 3obg.1.A, 3obj.1.A, 3oc1.1.A, 3ocb.1.A, 3ocb.2.A, 3ocg.1.A, 3ocs.1.A, 3oct.1.A, 3od6.1.A, 3ody.1.A, 3odz.1.A, 3oef.1.A, 3oez.1.A, 3oez.2.A, 3of0.1.A, 3ofm.1.A, 3og7.1.A, 3og7.2.A, 3oht.1.A, 3omv.1.A, 3oog.1.A, 3op5.2.A, 3op5.3.A, 3ori.1.A, 3ork.1.A, 3orl.1.A, 3orm.1.A, 3orn.2.A, 3oro.1.A, 3orx.1.A, 3orx.5.A, 3orz.1.A, 3os3.1.A, 3otu.1.A, 3otv.1.A, 3otv.3.A, 3otv.4.A, 3ouk.1.A, 3oun.1.B, 3ovv.1.A, 3owj.1.A, 3oxi.1.A, 3oxz.1.A, 3oy1.1.A, 3oy3.1.A, 3oz6.1.A, 3oz6.1.B, 3p08.3.A, 3p23.1.A, 3p23.1.B, 3p4k.1.A, 3p7a.1.A, 3p7c.1.A, 3p86.1.A, 3p86.2.A, 3p9j.1.A, 3pa4.1.A, 3pe2.1.A, 3pfq.1.A, 3pg1.1.A, 3pix.1.A, 3piy.1.A, 3piz.1.A, 3pj2.1.A, 3pj8.1.A, 3pjc.1.A, 3pls.1.A, 3plt.1.A, 3plt.1.B, 3plt.2.A, 3poz.1.A, 3pp0.1.A, 3pp0.2.A, 3ppj.1.A, 3ppz.1.A, 3psc.1.A, 3ptg.1.A, 3pvb.1.A, 3pvg.1.A, 3pwy.1.A, 3pxk.1.B, 3pxk.2.A, 3pxq.1.A, 3pxr.1.A, 3py0.1.A, 3py1.1.A, 3py3.1.A, 3pyy.1.A, 3pyy.2.A, 3pze.1.A, 3q04.1.A, 3q2j.1.A, 3q2j.1.B, 3q2m.2.B, 3q32.1.A, 3q32.2.A, 3q3b.1.A, 3q3b.2.A, 3q4c.1.A, 3q4t.1.A, 3q4t.2.A, 3q4u.1.A, 3q4u.1.B, 3q4u.3.A, 3q4z.1.A, 3q4z.1.B, 3q52.1.A, 3q5i.1.A, 3q5z.1.A, 3q60.1.A, 3q6u.1.A, 3q6w.1.A, 3q96.1.A, 3q9w.1.A, 3q9x.2.A, 3q9z.1.A, 3qa0.1.A, 3qa8.2.B, 3qal.1.A, 3qam.1.A, 3qbn.1.A, 3qc4.1.A, 3qc4.2.A, 3qc9.1.A, 3qd2.1.A, 3qd3.1.A, 3qfv.1.A, 3qgw.1.A, 3qgw.2.A, 3qgy.2.A, 3qhr.1.A, 3qkk.1.A, 3qkl.1.A, 3qqj.1.A, 3qqu.1.A, 3qqu.3.A, 3qrj.1.A, 3qrk.1.A, 3qru.1.A, 3qti.1.A, 3qtr.1.A, 3qtu.1.A, 3qtw.1.A, 3que.1.A, 3qup.1.A, 3qwj.1.A, 3qwk.1.A, 3qxp.1.A, 3qyw.1.A, 3qzg.1.A, 3r21.1.A, 3r22.1.A, 3r2b.1.A, 3r2y.1.A, 3r63.1.A, 3r78.1.A, 3r78.2.A, 3r83.1.A, 3r8m.1.A, 3r8v.1.A, 3r8z.1.A, 3r9d.1.A, 3rai.1.A, 3raw.1.A, 3rcd.1.A, 3rcd.4.A, 3rcj.1.A, 3re4.1.A, 3rf3.3.B, 3rgf.1.A, 3rhk.1.A, 3rhx.1.A, 3ri1.1.A, 3rin.1.A, 3rkb.1.A, 3rm6.1.A, 3rni.1.A, 3rny.1.A, 3rp0.1.A, 3rp0.2.A, 3rp9.1.A, 3rps.1.A, 3rps.2.A, 3rtp.1.A, 3rvg.1.A, 3rwp.1.A, 3rwq.1.A, 3rzf.1.A, 3s0o.1.A, 3s3i.1.A, 3s4q.1.A, 3s90.1.A, 3s90.2.A, 3say.1.A, 3sd0.1.A, 3sd0.1.B, 3sdj.1.A, 3sdm.1.A, 3sg8.1.A, 3sg9.1.A, 3sgc.1.A, 3she.1.A, 3sls.1.A, 3soa.1.A, 3soc.1.A, 3sqq.1.A, 3srv.1.A, 3sv0.1.A, 3svv.1.A, 3sxr.1.A, 3sxs.1.A, 3t8o.1.A, 3t9i.1.A, 3t9t.1.A, 3tac.1.A, 3tdv.1.A, 3tdw.1.A, 3tei.1.A, 3tg1.1.A, 3thb.1.A, 3ti1.1.A, 3tiy.1.A, 3tj5.1.A, 3tj6.1.A, 3tjd.1.A, 3tjd.2.A, 3tku.1.A, 3tl8.1.A, 3tl8.2.A, 3tl8.3.A, 3tm0.1.A, 3tni.1.A, 3tnp.2.A, 3tnq.1.A, 3tt0.1.A, 3tt0.2.A, 3ttj.1.A, 3tub.1.A, 3tuc.1.A, 3tud.1.A, 3tv7.1.A, 3tv7.1.B, 3tv7.2.A, 3twj.1.A, 3twj.1.B, 3twj.2.A, 3twj.2.B, 3txo.1.A, 3tyk.1.A, 3tz7.2.A, 3tz9.2.A, 3tzm.1.A, 3u4u.1.A, 3u4w.1.A, 3u51.1.A, 3u6j.1.A, 3u9n.1.A, 3ubd.1.A, 3uc3.1.A, 3uc4.1.A, 3uc4.2.A, 3udb.1.A, 3udb.2.A, 3udb.3.A, 3udb.4.A, 3udb.5.A, 3udb.6.A, 3ue4.1.A, 3ue4.2.A, 3ug1.1.B, 3ugc.1.A, 3uib.1.A, 3uim.1.A, 3uiu.1.A, 3ujg.1.A, 3unj.1.A, 3unk.1.A, 3unz.3.A, 3uo4.1.A, 3uo5.1.A, 3uod.1.A, 3uoj.2.A, 3uok.1.A, 3uol.3.B, 3up2.1.A, 3up7.1.A, 3uqc.1.A, 3uqc.1.B, 3uqc.2.B, 3uqf.1.A, 3uqf.2.A, 3uto.1.A, 3uto.2.A, 3uvp.1.A, 3uvr.1.A, 3uys.2.A, 3uyt.2.A, 3uyt.5.A, 3uyt.5.B, 3uyt.6.A, 3uzp.1.A, 3uzp.2.A, 3uzr.1.A, 3uzs.1.A, 3uzt.1.A, 3v3v.1.A, 3v5j.1.A, 3v5q.1.A, 3v5t.1.A, 3v5w.1.A, 3v6s.1.A, 3v6s.2.A, 3v8s.1.A, 3vap.1.A, 3vby.1.A, 3vhe.1.A, 3vhk.1.A, 3vid.1.A, 3vjo.1.A, 3vn9.1.A, 3vnt.1.A, 3vqu.1.A, 3vry.2.A, 3vs0.2.A, 3vs3.2.A, 3vs6.1.A, 3vs7.1.A, 3vs7.2.A, 3vud.1.A, 3vui.1.A, 3vul.1.A, 3vut.1.A, 3vut.2.A, 3vvh.1.A, 3vvh.2.A, 3vw6.1.A, 3vw6.1.B, 3vw8.1.A, 3vwa.1.A, 3w0m.1.A, 3w0n.1.A, 3w0p.1.A, 3w0q.1.A, 3w0r.1.A, 3w0s.1.A, 3w10.1.A, 3w16.1.A, 3w18.1.A, 3w18.2.A, 3w1f.1.A, 3w2c.1.A, 3w2o.1.A, 3w2r.1.A, 3w2s.1.A, 3w32.1.A, 3w55.1.A, 3w8l.1.A, 3w8l.2.A, 3w8q.1.A, 3we8.1.A, 3wf5.1.A, 3wf7.1.A, 3wf8.1.A, 3wi6.1.B, 3wik.1.A, 3wil.1.A, 3wow.1.A, 3wyx.1.A, 3wzd.1.A, 3wze.1.A, 3wzu.1.A, 3x2u.1.A, 3zc6.1.A, 3zc6.2.A, 3zdi.1.A, 3zdl.1.A, 3zdu.1.A, 3zep.1.A, 3zep.2.A, 3zep.3.A, 3zew.1.A, 3zfm.1.A, 3zfx.1.A, 3zfy.1.A, 3zh8.1.A, 3zhp.1.B, 3zls.1.A, 3zlw.1.A, 3zly.1.A, 3zmm.1.A, 3zon.1.A, 3zos.2.A, 3zrk.1.A, 3zsh.1.A, 3zsi.1.A, 3ztx.1.A, 3ztx.2.A, 3zu7.1.A, 3zut.1.A, 3zuu.1.A, 3zuv.1.A, 3zxt.1.A, 3zxt.1.B, 3zxt.2.A, 3zya.1.A, 3zzw.1.A, 3zzw.1.B, 4a4x.1.A, 4a7c.1.A, 4aaa.1.A, 4acc.1.A, 4acd.1.B, 4ach.1.A, 4ae6.1.A, 4ae6.2.A, 4af3.1.A, 4afe.1.A, 4afj.1.A, 4agc.1.A, 4agu.1.A, 4agw.1.A, 4akv.1.A, 4akv.1.B, 4alu.1.A, 4alv.1.A, 4an2.1.A, 4anl.1.A, 4anm.1.A, 4ans.1.A, 4aoj.1.A, 4aot.1.A, 4aot.2.A, 4ap7.1.A, 4apc.1.A, 4app.1.A, 4aqc.1.A, 4aqc.2.A, 4ark.1.B, 4asd.1.A, 4asz.1.A, 4at3.1.A, 4at4.1.A, 4at5.1.A, 4au8.1.A, 4aw2.1.A, 4aw5.1.A, 4awi.1.A, 4ax8.1.A, 4azf.1.A, 4azt.1.A, 4azw.1.A, 4b0g.1.A, 4b4l.1.A, 4b6l.1.A, 4b7t.1.A, 4b8l.1.A, 4b8m.1.A, 4b8m.2.A, 4b99.1.A, 4bb4.1.A, 4bbe.1.A, 4bbf.3.A, 4bbm.1.A, 4bc6.1.A, 4bcf.1.A, 4bcg.1.A, 4bci.1.A, 4bcj.1.A, 4bck.1.A, 4bck.2.A, 4bcm.1.A, 4bcn.1.A, 4bco.1.A, 4bcq.1.A, 4bcq.2.A, 4bfm.1.A, 4bgq.1.A, 4bib.1.A, 4bib.2.A, 4bkj.2.A, 4bn1.1.A, 4br3.1.B, 4brx.1.A, 4btf.1.A, 4btj.1.A, 4btj.2.A, 4btk.1.A, 4bvu.1.A, 4bwk.1.A, 4bwk.1.B, 4bwp.1.A, 4bwp.1.B, 4bwx.1.A, 4bwx.1.B, 4byi.1.A, 4byj.1.A, 4bzo.1.A, 4c02.1.A, 4c0t.1.A, 4c2v.1.A, 4c2v.2.A, 4c2w.1.A, 4c2w.2.A, 4c33.1.A, 4c34.1.A, 4c35.1.A, 4c3p.1.A, 4c3p.1.C, 4c3r.1.A, 4c57.2.A, 4c8b.1.A, 4cfe.1.A, 4cfh.1.A, 4cfn.1.A, 4cfu.1.A, 4cfu.2.A, 4cfw.1.A, 4cfx.1.A, 4cfx.2.A, 4cki.1.A, 4ckr.1.A, 4cnh.2.A, 4cqe.1.A, 4cqe.2.A, 4cqg.1.A, 4crs.1.A, 4csv.1.A, 4ct1.1.A, 4cxa.2.A, 4cyi.1.A, 4cyi.1.B, 4cyj.1.A, 4cyj.1.B, 4czt.1.A, 4czt.1.B, 4czt.2.A, 4czt.2.B, 4czu.1.A, 4czu.1.B, 4czu.2.A, 4czu.2.B, 4czy.1.B, 4czy.1.D, 4d1s.1.A, 4d28.1.A, 4d28.1.B, 4d28.2.A, 4d28.2.B, 4d2p.1.A, 4d2p.1.B, 4d2p.2.A, 4d2p.2.B, 4d2r.1.A, 4d2s.1.A, 4d2t.2.A, 4d2t.4.A, 4d2w.1.B, 4d4r.1.A, 4d4r.2.A, 4d4v.1.A, 4d4v.2.A, 4d55.1.A, 4d9t.1.A, 4da5.1.A, 4dbn.1.A, 4dbn.1.B, 4dbx.1.A, 4dc2.1.A, 4dca.1.A, 4dce.1.A, 4de4.2.A, 4deb.1.A, 4dee.1.A, 4deg.1.A, 4dfb.1.A, 4dfb.2.A, 4dfl.1.A, 4dfu.1.A, 4dfu.2.A, 4dfy.1.A, 4dfz.1.A, 4dg0.1.A, 4dg2.1.A, 4dg3.1.A, 4dgg.1.A, 4dgl.1.C, 4dgl.1.D, 4dgm.1.A, 4dgn.1.A, 4dh7.1.A, 4dhf.1.A, 4din.1.A, 4dit.1.A, 4dj9.1.A, 4dn5.3.A, 4dt9.1.A, 4dta.1.A, 4dtb.1.A, 4dtk.1.A, 4dym.1.A, 4e17.1.A, 4e18.1.A, 4e1z.1.A, 4e20.1.A, 4e26.1.B, 4e3c.1.A, 4e3c.1.B, 4e3c.2.A, 4e3c.2.B, 4e3c.3.A, 4e3c.3.B, 4e4l.1.A, 4e4m.1.A, 4e4x.1.A, 4e5a.1.A, 4e5b.1.A, 4e6d.1.A, 4e6d.2.A, 4e6q.1.A, 4e73.1.A, 4e7w.1.A, 4e93.1.A, 4ebw.1.A, 4ec9.1.A, 4eev.1.A, 4efo.1.A, 4eh2.1.A, 4eh5.1.A, 4eh9.1.A, 4ehg.1.B, 4ehp.1.A, 4ehv.1.A, 4ejn.1.A, 4eoi.1.A, 4eoi.2.A, 4eoj.1.A, 4eoj.2.A, 4eok.1.A, 4eok.2.A, 4eom.1.A, 4eom.2.A, 4eon.1.A, 4eon.2.A, 4eoo.1.A, 4eoo.2.A, 4eop.1.A, 4eop.2.A, 4eoq.1.A, 4eoq.2.A, 4eor.1.A, 4eor.2.A, 4eos.1.A, 4eos.2.A, 4eqc.1.A, 4eqm.1.A, 4eqm.4.A, 4equ.1.A, 4erk.1.A, 4erw.1.A, 4eut.1.A, 4eut.1.B, 4euu.1.A, 4ewh.1.A, 4ewq.1.A, 4eyj.1.A,

4eym.1.A, 4ez3.1.A, 4ez5.1.A, 4ez7.1.A, 4f08.2.A, 4f09.1.A, 4f0f.1.A, 4f0g.1.A, 4f0i.1.A, 4f0i.2.A, 4f1m.1.A, 4f1o.1.A, 4f1t.1.A, 4f4p.1.A, 4f65.1.A, 4f6s.1.A, 4f6u.1.A, 4f6w.1.A, 4f7l.1.A, 4f9a.1.A, 4fbx.1.A, 4feu.2.A, 4feu.3.A, 4fev.1.A, 4fev.2.A, 4fev.3.A, 4fev.4.A, 4fev.6.A, 4fex.3.A, 4fex.4.A, 4fex.5.A, 4ff8.1.A, 4fg7.1.A, 4fg8.1.A, 4fg9.1.A, 4fgb.1.A, 4fie.1.A, 4fif.2.A, 4fk3.1.A, 4fk3.1.B, 4fl1.1.A, 4fl2.1.A, 4fl3.1.A, 4fmq.1.A, 4fnw.1.A, 4fnx.1.A, 4fny.1.A, 4fnz.1.A, 4fob.1.A, 4fr4.1.A, 4fr4.4.A, 4fsn.1.A, 4fst.1.A, 4fsu.1.A, 4ft3.1.A, 4fv3.1.A, 4fv7.1.A, 4fv8.1.A, 4fx3.2.A, 4fza.1.B, 4fzd.1.B, 4fzf.1.B, 4g1w.1.A, 4g31.1.A, 4g3c.1.A, 4g3c.2.A, 4g3d.1.A, 4g3d.2.A, 4g3f.1.A, 4g3g.1.A, 4g5j.1.A, 4g5p.1.A, 4g5p.2.A, 4g9c.1.A, 4g9c.2.A, 4g9r.1.A, 4gcj.1.A, 4geo.1.A, 4gfm.1.A, 4gfo.1.A, 4gg5.1.A, 4gg7.1.A, 4gii.1.A, 4gl9.1.A, 4gmy.1.A, 4gs6.1.A, 4gt3.1.A, 4gt4.1.A, 4gt5.1.A, 4gu6.1.B, 4gu6.2.A, 4gub.1.A, 4gv1.1.A, 4gvj.1.A, 4gyg.1.A, 4h05.1.A, 4h05.2.A, 4h1j.1.A, 4h3b.1.A, 4h3p.1.A, 4h3q.1.A, 4h58.1.A, 4h58.2.A, 4h58.3.A, 4h9n.1.C, 4h9q.1.C, 4h9s.1.C, 4h9s.2.C, 4hct.1.A, 4hga.1.A, 4hgs.1.A, 4hgt.2.A, 4hjo.1.A, 4hnf.1.A, 4hnf.2.A, 4hni.1.A, 4hok.1.A, 4hok.11.A, 4hok.12.A, 4hok.6.A, 4hok.9.A, 4hvg.1.A, 4hvs.2.B, 4hw7.1.A, 4hyh.1.A, 4hyi.1.A, 4hzr.1.A, 4hzs.1.A, 4i1z.1.A, 4i20.1.A, 4i21.1.A, 4i21.2.A, 4i22.1.A, 4i23.1.A, 4i24.1.A, 4i3z.1.A, 4i41.1.A, 4i4f.1.A, 4i5m.1.A, 4i6h.1.A, 4i92.1.A, 4i93.1.A, 4i93.2.A, 4i94.1.A, 4i94.2.A, 4iaa.1.A, 4ian.1.B, 4iay.1.A, 4ib5.1.A, 4ib5.2.A, 4ib5.3.A, 4ibm.1.A, 4ibm.2.A, 4ic7.1.A, 4ic7.2.A, 4ic8.1.A, 4id7.1.A, 4idt.1.A, 4idt.1.B, 4idv.1.A, 4ieb.1.A, 4ifc.1.A, 4ifc.2.A, 4ifg.1.A, 4ii5.1.A, 4ijp.1.A, 4imy.1.A, 4imy.3.A, 4iq6.1.A, 4ith.1.A, 4ith.2.A, 4iti.1.A, 4iti.2.A, 4itj.1.A, 4itj.2.A, 4iva.1.A, 4iw0.1.A, 4iwd.1.A, 4iwo.1.A, 4iwp.1.A, 4ix3.1.A, 4ix3.1.B, 4ixp.1.A, 4iz5.1.A, 4iz7.1.A, 4iz7.2.A, 4iza.1.A, 4j1r.2.A, 4j52.1.A, 4j7b.1.A, 4j8m.1.A, 4j8n.3.A, 4j95.5.A, 4j96.1.A, 4j96.2.A, 4j97.1.A, 4j98.1.A, 4j98.2.A, 4j99.1.A, 4jai.1.A, 4jaj.1.A, 4jbo.1.A, 4jbq.1.A, 4jdj.1.A, 4jg6.1.A, 4jg7.1.A, 4jg8.1.A, 4ji9.1.A, 4ji9.2.A, 4jia.1.A, 4jjr.1.A, 4jjr.2.A, 4jl9.1.A, 4jlc.1.B, 4jnw.1.A, 4jq7.1.A, 4jr7.1.A, 4jrn.1.A, 4js8.1.A, 4jt3.1.A, 4jvg.1.A, 4jvg.2.A, 4jx3.1.A, 4jxf.1.A, 4k0y.1.A, 4k11.1.A, 4k18.1.A, 4k1p.1.A, 4k2r.1.A, 4k33.1.A, 4k6z.1.A, 4k9y.1.A, 4kab.1.A, 4kao.1.A, 4kao.2.A, 4kb8.1.A, 4kbc.3.A, 4kbc.3.B, 4kin.1.A, 4kin.4.A, 4kio.2.A, 4kip.1.A, 4kiq.1.A, 4kiq.4.A, 4knb.1.A, 4knb.4.A, 4krc.1.A, 4ks7.1.A, 4ks8.1.A, 4ksp.1.B, 4kwp.1.A, 4l00.1.A, 4l00.2.A, 4l01.1.A, 4l01.2.A, 4l3j.1.A, 4l3p.1.A, 4l42.1.A, 4l43.1.A, 4l44.1.A, 4l45.1.A, 4l46.1.A, 4l52.1.A, 4l67.1.A, 4l68.1.A, 4l68.2.A, 4l6q.1.A, 4l7f.1.A, 4l8m.1.A, 4l9i.1.A, 4lfi.1.B, 4lg4.1.A, 4lg4.2.A, 4lg4.3.A, 4lg4.4.A, 4lg4.5.A, 4lg4.6.A, 4lgd.1.A, 4lgd.2.A, 4lgd.3.A, 4lgd.4.A, 4lgg.1.A, 4lgh.1.A, 4lgh.2.A, 4li5.1.A, 4ll0.1.A, 4loo.1.A, 4lop.1.A, 4lop.3.A, 4loq.4.A, 4lqm.1.A, 4lqp.1.A, 4lqq.1.A, 4lqq.2.A, 4lqs.1.A, 4lrj.1.A, 4lrk.1.A, 4lrk.2.A, 4lrk.3.A, 4lrm.1.A, 4lrm.2.A, 4lrm.3.A, 4lrm.4.A, 4lrm.5.A, 4lv5.2.A, 4lv8.1.A, 4m0y.1.A, 4m66.1.A, 4m67.1.A, 4m68.1.A, 4m69.1.A, 4m69.1.B, 4m8t.1.A, 4m97.1.A, 4mao.1.A, 4mbi.1.A, 4mbj.1.A, 4mcv.1.A, 4md7.1.C, 4md8.1.C, 4md9.1.C, 4mf0.1.B, 4mf1.1.B, 4mh7.2.A, 4mk0.1.A, 4mne.1.B, 4mne.1.C, 4mne.2.A, 4mne.2.D, 4mnf.1.A, 4mnf.1.B, 4mta.1.A, 4mvf.1.A, 4mwi.1.A, 4mxa.1.A, 4mxc.1.A, 4mxo.1.A, 4mxx.1.A, 4mxx.2.A, 4mxy.1.A, 4mxy.2.A, 4myg.1.A, 4myg.2.A, 4n57.2.A, 4nct.3.A, 4nct.4.A, 4neu.1.A, 4neu.2.A, 4nfm.1.A, 4nfn.1.A, 4nh1.1.A, 4nif.1.A, 4nif.1.D, 4nj3.1.A, 4njd.1.A, 4nm0.1.A, 4nm3.1.A, 4nm5.1.A, 4nt4.1.A, 4nts.1.A, 4nts.2.A, 4ntt.1.A, 4ntt.2.A, 4nus.1.A, 4nw6.1.A, 4nwm.1.A, 4nzw.1.B, 4o0r.1.A, 4o0r.2.A, 4o0s.1.A, 4o0t.1.A, 4o0t.2.A, 4o0u.1.A, 4o0v.1.A, 4o0w.1.A, 4o1o.2.A, 4o21.1.A, 4o27.1.B, 4o2p.1.A, 4o2z.1.A, 4o38.1.A, 4o6e.1.A, 4o6l.2.A, 4o7o.1.A, 4o7o.1.B, 4o7p.1.B, 4o91.2.C, 4o96.1.A, 4o96.2.A, 4oau.1.A, 4oav.1.A, 4oav.1.B, 4obq.1.A, 4obq.1.B, 4ocj.1.A, 4ocu.1.A, 4ogr.1.A, 4oh4.1.A, 4oh4.2.A, 4oli.1.A, 4or5.1.A, 4or5.2.A, 4ork.1.A, 4ork.3.A, 4ork.4.A, 4otd.1.A, 4otf.1.A, 4oth.1.A, 4otp.1.A, 4ow8.1.A, 4p2k.1.A, 4p2w.3.B, 4p4c.1.A, 4p90.1.A, 4p90.2.A, 4pdo.1.B, 4pdp.1.A, 4pdy.1.A, 4ped.1.A, 4pf4.1.A, 4pl3.1.A, 4pl3.1.B, 4pl4.1.A, 4pl4.1.B, 4pl4.2.A, 4pl4.2.B, 4pl5.1.A, 4pl5.1.B, 4pl5.2.A, 4pl5.2.B, 4pmm.1.A, 4pmt.1.A, 4pni.1.A, 4pnk.1.A, 4pp7.1.A, 4pp9.1.A, 4ppa.3.A, 4ppb.3.B, 4ppc.3.A, 4ppc.3.B, 4pqn.1.A, 4prj.1.A, 4pte.1.A, 4pv0.1.A, 4pwn.1.A, 4px6.1.A, 4py1.1.A, 4q2a.1.A, 4q5e.1.A, 4q5j.1.A, 4q5j.2.A, 4q9z.1.A, 4q9z.1.B, 4qd6.1.A, 4qd6.2.A, 4qfg.1.A, 4qml.1.A, 4qmm.1.A, 4qmn.1.A, 4qmt.1.A, 4qny.1.A, 4qo9.1.A, 4qo9.2.A, 4qox.1.A, 4qp1.1.A, 4qp1.2.A, 4qp2.2.A, 4qp3.1.A, 4qp3.2.A, 4qp4.1.A, 4qp4.2.A, 4qpa.1.A, 4qpm.1.A, 4qps.1.A, 4qps.2.A, 4qq5.1.A, 4qqc.1.A, 4qqj.1.A, 4qqt.1.A, 4qta.1.A, 4qte.2.B, 4qye.1.A, 4qyy.1.A, 4r1v.1.A, 4r1y.1.A, 4r3c.1.A, 4r3p.1.A, 4r3r.1.A, 4r5s.1.A, 4r5y.1.A, 4r77.1.A, 4r78.1.A, 4r7b.1.A, 4r8q.1.A, 4ra4.1.A, 4red.1.A, 4red.1.B, 4rer.1.A, 4rew.1.A, 4rfm.1.A, 4rgj.1.A, 4rh7.1.A, 4rio.1.A, 4riw.1.A, 4riw.1.B, 4rix.1.A, 4rix.1.B, 4rix.2.B, 4riy.1.A, 4riy.1.B, 4riy.2.B, 4rlo.1.A, 4rlo.2.A, 4rlp.1.A, 4rmz.1.A, 4rpv.1.A, 4rss.1.A, 4rt7.1.A, 4rvt.1.A, 4rvt.1.B, 4rwi.1.A, 4rwi.2.A, 4rwl.2.A, 4rx5.1.A, 4rx9.1.A, 4rz7.1.B, 4rzv.1.A, 4rzv.2.A, 4rzw.1.A, 4rzw.1.B, 4s2z.1.A, 4s30.1.A, 4s31.1.A, 4s33.1.A, 4tks.1.A, 4tl0.1.A, 4tn6.1.A, 4tn6.2.A, 4tnb.1.A, 4tpt.1.A, 4tt7.1.A, 4tth.1.B, 4tw9.1.A, 4tw9.1.B, 4twn.1.A, 4twp.1.A, 4twp.2.A, 4txc.1.A, 4tye.1.A, 4tyg.1.A, 4tyh.1.A, 4tyh.1.B, 4tyi.1.A, 4tyj.1.A, 4u0i.1.A, 4u3y.1.A, 4u3y.1.B, 4u3z.1.A, 4u3z.1.B, 4u40.2.A, 4u41.2.A, 4u42.1.A, 4u42.1.B, 4u43.1.A, 4u43.3.A, 4u44.2.A, 4u45.2.A, 4u45.3.A, 4u5j.2.A, 4u6r.1.A, 4u79.1.A, 4u7z.1.A, 4u94.1.A, 4u9a.1.A, 4u9a.1.B, 4uak.1.A, 4ub7.1.A, 4uba.1.A, 4uba.2.A, 4ueu.1.A, 4ump.1.A, 4ump.1.B, 4umq.1.A, 4umt.1.A, 4un0.1.B, 4usd.1.A, 4use.2.A, 4usf.1.A, 4usf.2.A, 4uv0.1.A, 4uw0.1.A, 4uwy.1.A, 4uwy.2.A, 4ux9.1.A, 4ux9.4.A, 4uxl.1.A, 4uxq.1.A, 4uxv.1.A, 4uy9.1.A, 4uya.1.A, 4uyn.1.A, 4uzd.1.A, 4uzh.1.A, 4v01.2.A, 4v0g.1.A, 4v0g.2.A, 4w4y.1.A, 4w7p.1.A, 4w8d.1.A, 4w8e.1.A, 4w9w.1.A, 4wa9.1.A, 4wa9.2.A, 4wb5.1.A, 4wb6.1.A, 4wb7.1.A, 4wb7.2.A, 4wb8.1.A, 4wbb.1.B, 4wbo.1.A, 4wd5.1.A, 4wh1.1.A, 4whz.1.A, 4wih.1.A, 4wnk.1.A, 4wnm.1.A, 4wnp.1.A, 4wnp.2.A, 4wnp.4.A, 4wo5.1.A, 4wo5.2.A, 4wot.1.A, 4wov.1.A, 4wsq.1.A, 4wsq.2.A, 4wsy.1.A, 4wua.1.A, 4wun.1.A, 4ww5.1.A, 4x0m.1.A, 4x3f.1.A, 4x3j.1.A, 4x6r.1.A, 4x7l.1.A, 4x7q.1.A, 4x7q.2.A, 4xbr.1.A, 4xbu.1.A, 4xcu.1.A, 4xey.1.A, 4xey.2.A, 4xg3.2.A, 4xh0.1.A, 4xh6.1.A, 4xhk.1.A, 4xhl.1.A, 4xi2.1.A, 4xj0.1.A, 4xj0.2.A, 4xli.1.A, 4xlv.1.A, 4xne.1.A, 4xoy.1.A, 4xr7.1.B, 4xr7.1.C, 4xr7.2.A, 4xr7.2.B, 4xr7.4.A, 4xr7.4.B, 4xrl.1.A, 4xuf.1.A, 4xuf.2.A, 4xv1.1.A, 4xv1.1.B, 4xv9.1.A, 4xx9.1.A, 4y0x.1.A, 4y5h.1.A, 4y5q.1.A, 4y66.1.B, 4y66.2.B, 4y66.3.B, 4y73.1.A, 4y73.2.A, 4y73.4.A, 4y83.3.A, 4y85.2.A, 4y8d.2.A, 4y93.1.A, 4y95.1.A, 4y95.2.A, 4ybk.1.A, 4yc3.1.A, 4yc6.1.A, 4yc8.1.A, 4yff.1.A, 4yfi.1.A, 4yfi.1.B, 4yga.1.A, 4yga.4.A, 4yhf.1.A, 4yhf.2.A, 4yhj.1.A, 4yhj.1.B, 4yht.1.A, 4yjo.1.A, 4yjq.1.A, 4yjt.1.A, 4yju.1.A, 4ymj.1.A, 4yne.1.A, 4ynz.1.A, 4ynz.1.B, 4yom.1.A, 4yp8.4.A, 4yr8.1.A, 4yr8.3.A, 4ysj.1.A, 4ysm.2.A, 4ytc.1.A, 4yur.1.A, 4yz9.1.A, 4yz9.2.A, 4yz9.3.A, 4yzb.2.A, 4yzc.1.A, 4yzd.1.A, 4yzd.3.A, 4yzm.1.A, 4yzm.2.A, 4yzn.1.A, 4z16.1.A, 4z3v.1.A, 4z55.1.A, 4z7g.1.A, 4z7h.2.A, 4z83.1.A, 4z84.1.A, 4zeg.1.A, 4zhx.1.A, 4zhx.2.A, 4zji.1.A, 4zji.3.A, 4zjv.2.A, 4zlo.1.A, 4zlo.1.B, 4zly.1.A, 4zog.1.A, 4zog.2.A, 4zop.1.B, 4zp5.1.A, 4zp5.2.A, 4zs0.1.A, 4zsa.1.A, 4zse.2.A, 4zsg.1.A, 4zsj.1.A, 4zsl.1.A, 4ztr.1.A, 4zts.1.A, 4zxt.1.A, 4zy4.1.A, 4zy4.2.A, 4zy6.1.A, 4zy6.2.A, 4zzm.1.A, 5a14.1.A, 5a46.1.A, 5a4c.1.A, 5a4e.3.A, 5a6n.1.A, 5a6n.1.B, 5a9u.1.A, 5aa8.1.A, 5aa9.1.A, 5aad.1.A, 5aae.1.A, 5aep.1.A, 5afv.1.B, 5aik.2.A, 5air.1.A, 5air.1.B, 5ajq.1.A, 5ajq.2.A, 5am6.2.A, 5am7.1.A, 5am7.2.A, 5amn.1.A, 5ane.1.A, 5ap0.1.A, 5ap1.1.A, 5ap2.1.A, 5ap3.1.A, 5ap6.1.A, 5ap7.1.A, 5apt.1.A, 5apt.1.B, 5apt.1.C, 5apu.1.A, 5apu.1.B, 5apu.1.C, 5apv.1.A, 5apv.1.B, 5apv.1.C, 5ar2.1.A, 5ar4.1.B, 5ar7.1.A, 5ar7.1.B, 5ar8.1.A, 5av4.1.A, 5awm.1.A, 5ax3.1.A, 5ax9.1.A, 5ax9.2.A, 5ax9.3.A, 5b0x.1.A, 5b2k.1.A, 5b2l.1.A, 5b2m.1.A, 5b7v.2.A, 5bmm.1.A, 5bms.1.A, 5bnj.1.A, 5bpy.1.A, 5bpy.2.A, 5bq0.1.A, 5bue.1.A, 5buo.1.A, 5bvf.1.A, 5bvk.1.A, 5bvo.1.A, 5byl.3.A, 5byl.4.A, 5byy.1.A, 5byz.1.A, 5c01.1.A, 5c03.2.A,

5c3l.1.A, 5c4k.2.A, 5c4l.1.A, 5c4l.2.A, 5cas.1.A, 5cav.1.A, 5ce3.1.B, 5cei.1.A, 5cek.1.A, 5cem.1.A, 5cen.1.A, 5ceo.1.A, 5cf5.1.A, 5cf5.2.A, 5ci6.1.A, 5ci6.2.A, 5ckw.1.A, 5ckw.1.B, 5clr.1.A, 5clr.1.B, 5cnn.1.A, 5cno.2.A, 5cqu.1.A, 5cs6.1.A, 5csh.2.A, 5csp.1.A, 5csw.1.A, 5csx.1.A, 5ct7.1.A, 5cu6.1.A, 5cvf.1.A, 5cvg.1.A, 5cwz.1.A, 5cwz.2.A, 5cwz.3.A, 5cxh.1.A, 5cxz.1.A, 5cy3.1.A, 5cyi.1.A, 5cyz.1.A, 5czh.1.A, 5czo.1.A, 5czp.1.s, 5d10.1.A, 5d10.2.A, 5d11.1.A, 5d11.2.A, 5d12.1.A, 5d12.2.A, 5d1j.1.A, 5d41.1.A, 5d41.1.B, 5d7a.1.A, 5d7a.3.A, 5d7v.1.A, 5d9h.1.A, 5d9k.1.A, 5d9l.1.A, 5da3.1.A, 5dbx.1.A, 5dbx.1.B, 5de2.1.A, 5de2.2.A, 5dew.2.A, 5dey.1.A, 5dey.2.A, 5dfe.1.C, 5dfe.2.C, 5dfp.1.A, 5dfz.1.C, 5dg5.1.B, 5dgz.1.A, 5dh3.1.A, 5di1.1.A, 5di1.2.A, 5dls.1.A, 5dmz.1.A, 5dmz.2.A, 5dnr.1.A, 5dos.1.A, 5drb.1.A, 5dt0.1.A, 5dt4.1.A, 5dvr.1.A, 5dyk.2.A, 5e1e.2.A, 5e1s.1.A, 5e7r.1.A, 5e8u.1.A, 5e8x.1.A, 5eak.1.A, 5ebz.1.A, 5ebz.1.F, 5ebz.2.E, 5ebz.2.F, 5ed9.1.A, 5ed9.1.B, 5ed9.1.C, 5edp.1.A, 5edr.1.A, 5efq.1.A, 5eg3.1.A, 5ei6.1.A, 5ek7.1.A, 5eob.1.A, 5eqe.1.A, 5es1.1.A, 5eta.1.A, 5eta.2.A, 5etc.1.A, 5etf.1.A, 5eti.1.A, 5ew3.1.A, 5ew3.2.A, 5ew9.1.A, 5eyk.1.A, 5eym.1.A, 5ezr.1.A, 5ezv.1.A, 5ezv.2.A, 5f1z.1.A, 5f4n.1.A, 5f94.1.A, 5f9e.1.A, 5f9e.2.A, 5fbo.1.A, 5fd2.1.A, 5fd2.1.B, 5fdp.1.A, 5fdx.1.A, 5fee.1.A, 5fg8.1.A, 5fgk.1.A, 5fi4.1.B, 5flf.1.A, 5flf.2.A, 5flf.3.A, 5flf.5.A, 5fm2.1.A, 5fqd.1.C, 5fri.1.A, 5ftg.1.B, 5fwl.1.D, 5fxq.1.A, 5fxr.1.A, 5fxs.1.A, 5g15.1.A, 5g1x.1.A, 5g6v.1.A, 5g6v.2.A, 5ghe.1.A, 5ghv.1.A, 5gjd.1.A, 5gjg.1.A, 5gmp.1.A, 5gnk.1.A, 5grn.1.A, 5gty.1.B, 5gty.1.C, 5gty.1.D, 5gz8.1.A, 5gza.1.A, 5h09.1.A, 5h0e.1.A, 5h2u.1.A, 5h3q.1.A, 5h8g.1.A, 5hbe.1.A, 5hbh.1.A, 5hd7.1.A, 5he1.1.A, 5he2.1.A, 5he3.1.A, 5hes.1.A, 5hez.1.A, 5hez.2.A, 5hg9.1.A, 5hgi.1.A, 5hhw.1.A, 5hi2.1.A, 5hie.1.A, 5hie.3.A, 5hie.4.A, 5hln.1.A, 5hln.1.B, 5hlp.2.A, 5hlw.1.A, 5hnb.1.A, 5hng.1.A, 5hni.1.A, 5hnv.1.A, 5hoa.1.A, 5hor.1.A, 5hti.1.A, 5hu3.1.A, 5hu9.1.A, 5hvj.1.A, 5hvj.2.A, 5hvk.1.A, 5hvk.2.A, 5hvy.1.A, 5hx6.1.A, 5hx6.2.A, 5hze.1.A, 5hzn.7.A, 5hzn.8.A, 5i35.1.A, 5i3o.1.A, 5i3o.2.A, 5i4n.1.A, 5i5z.1.A, 5i8a.1.A, 5i9v.1.A, 5i9w.1.A, 5i9y.1.A, 5ia0.2.A, 5ia0.3.A, 5ia1.1.A, 5ia4.1.A, 5idn.1.A, 5idp.1.A, 5if1.1.A, 5if1.2.A, 5ig1.1.A, 5ig1.2.A, 5igh.1.A, 5igr.1.A, 5igv.1.A, 5igy.1.A, 5igz.1.A, 5ih0.1.A, 5ih1.1.A, 5ih4.1.A, 5iha.1.A, 5ijn.1.F, 5ijn.1.G, 5ijn.1.H, 5ijn.1.L, 5ijn.1.R, 5ikw.1.A, 5ime.1.A, 5ime.2.A, 5imx.1.A, 5iqc.1.A, 5iqh.1.A, 5iqh.4.A, 5iqi.1.A, 5iqi.4.A, 5iso.1.A, 5iso.2.A, 5ita.1.A, 5ita.2.A, 5itd.1.B, 5iu2.2.A, 5iuh.1.A, 5iwu.1.A, 5izj.1.A, 5izj.2.A, 5j0a.1.A, 5j0a.1.B, 5j0k.1.A, 5j0k.1.B, 5j1g.1.A, 5j1v.2.A, 5j1v.3.A, 5j5t.1.A, 5j79.1.A, 5j7s.1.A, 5j87.1.A, 5j87.1.D, 5j8i.1.A, 5j95.1.A, 5j95.2.A, 5j9l.1.A, 5j9y.1.A, 5j9z.1.A, 5jeb.1.A, 5jfs.1.A, 5jga.1.A, 5jh6.1.A, 5jk3.1.A, 5jkg.1.A, 5jn2.1.A, 5jr7.1.A, 5jr7.2.A, 5jrq.1.A, 5jsm.2.A, 5jsm.2.B, 5jy7.1.E, 5jy7.1.F, 5jy7.1.H, 5jzj.1.A, 5jzj.2.A, 5jzn.1.A, 5k00.1.A, 5k0k.1.A, 5k0x.1.A, 5k0x.2.A, 5k3y.1.A, 5k3y.2.A, 5k4i.1.A, 5k4j.1.A, 5k5n.1.A, 5k5x.1.A, 5k72.1.A, 5k75.3.A, 5k7i.1.A, 5k9i.2.A, 5kbq.1.A, 5kbq.2.A, 5kbr.1.A, 5kc2.1.B, 5kcv.1.A, 5kcx.1.A, 5kdm.1.C, 5ke0.1.A, 5khw.2.A, 5khx.1.A, 5kkr.1.A, 5kkr.1.B, 5kmi.1.A, 5kmk.1.A, 5kml.1.A, 5knj.1.A, 5ko1.1.A, 5kpk.2.A, 5kpm.1.A, 5ku8.1.A, 5kud.1.A, 5kvt.1.A, 5kx7.2.A, 5kx8.1.A, 5kx8.2.A, 5kx8.3.A, 5kx8.4.A, 5kz8.1.A, 5kz8.2.A, 5l1z.2.A, 5l2i.1.A, 5l2q.1.A, 5l2q.3.A, 5l2q.4.A, 5l2s.1.A, 5l2t.1.A, 5l2w.1.A, 5l3a.1.A, 5l4q.1.A, 5l6w.1.A, 5l8k.1.A, 5l8l.1.A, 5lar.1.A, 5lcj.1.A, 5lck.1.A, 5lcq.1.A, 5lcr.1.A, 5li1.1.A, 5li9.1.A, 5lih.1.A, 5ljj.1.A, 5lm1.1.A, 5lm2.1.A, 5lm2.2.A, 5lma.1.A, 5lmk.2.A, 5loh.1.A, 5lpw.1.A, 5lpz.1.A, 5lqf.1.A, 5lvp.1.A, 5lw1.2.B, 5lwm.1.A, 5lxc.1.A, 5lxd.1.A, 5lxm.1.A, 5m06.1.A, 5m07.1.A, 5m08.1.A, 5m08.2.A, 5m09.1.A, 5m09.2.A, 5m0l.1.A, 5m44.1.A, 5m4c.1.A, 5m4f.1.A, 5m4i.1.A, 5m4u.1.A, 5m51.1.A, 5m53.1.A, 5m56.1.A, 5m56.2.A, 5m57.1.A, 5mag.1.A, 5mah.1.A, 5mai.1.A, 5mg8.1.B, 5mg8.2.B, 5mhq.1.A, 5mja.1.A, 5mjb.1.A, 5ml5.1.A, 5mo4.1.A, 5mov.1.A, 5mow.2.A, 5mqv.1.A, 5mqv.5.A, 5mrb.1.A, 5mrd.1.A, 5mty.1.A, 5mxx.1.A, 5my8.1.A, 5myv.2.A, 5myv.3.A, 5myv.4.A, 5mz3.1.A, 5mzl.1.A, 5n1g.1.A, 5n1v.1.A, 5n23.1.A, 5n4v.1.A, 5n63.1.A, 5n65.1.A, 5n7v.1.A, 5n84.1.A, 5n93.1.A, 5na0.1.A, 5nad.1.A, 5ncl.1.A, 5nev.1.A, 5ng0.1.A, 5ng2.1.A, 5ng3.1.A, 5ngu.1.A, 5nhh.1.A, 5nk0.1.A, 5nk3.1.A, 5nk5.1.A, 5nk7.1.A, 5nka.1.A, 5nqc.1.A, 5ntj.1.A, 5ntt.1.A, 5nud.1.A, 5nud.2.A, 5nwz.1.A, 5nwz.2.A, 5nxc.1.A, 5nxd.1.A, 5nxd.2.A, 5nzz.1.B, 5nzz.2.B, 5o0y.1.A, 5o11.1.A, 5o1s.1.A, 5o1v.1.A, 5o1v.1.B, 5o21.1.A, 5o23.1.A, 5o23.2.A, 5o26.1.A, 5o2b.1.A, 5o2b.2.A, 5o2c.1.A, 5o7i.1.A, 5o8u.1.A, 5o8v.1.A, 5o90.1.A, 5oat.1.A, 5obj.1.A, 5obr.1.A, 5odt.1.A, 5okt.2.A, 5okt.4.A, 5omg.1.A, 5omy.1.A, 5one.1.A, 5oni.1.A, 5oo0.1.A, 5oo1.1.A, 5ooi.1.A, 5oop.1.A, 5op2.1.A, 5opu.1.A, 5opv.1.A, 5oq5.1.A, 5oq6.1.A, 5oq7.1.A, 5oq8.1.A, 5orl.1.A, 5os5.1.A, 5os7.1.A, 5osd.1.A, 5osl.1.A, 5osm.1.A, 5osz.1.A, 5otf.1.A, 5otq.1.A, 5owl.1.A, 5owq.3.B, 5owr.1.A, 5oy4.1.A, 5oy6.3.A, 5oy6.4.A, 5p9f.1.A, 5p9k.1.A, 5p9m.1.A, 5qik.1.A, 5qin.1.A, 5r8z.1.A, 5r9d.1.A, 5s7g.1.A, 5s86.1.B, 5sav.1.A, 5saw.1.A, 5sax.1.A, 5say.1.A, 5say.2.A, 5sb1.1.A, 5sb2.1.A, 5swh.1.A, 5sys.1.A, 5t0p.1.A, 5t31.1.A, 5t3q.1.B, 5t68.1.A, 5t8o.1.A, 5t8p.1.A, 5t8q.1.A, 5t8q.2.A, 5ta6.1.A, 5tbe.1.A, 5tc0.1.A, 5tc0.2.A, 5tco.1.A, 5td2.1.A, 5td2.2.A, 5te0.1.A, 5teh.1.A, 5teh.2.A, 5tex.1.A, 5tf9.1.A, 5tiu.1.A, 5tkd.1.A, 5to8.1.A, 5tob.1.A, 5toe.1.A, 5tos.1.A, 5tq3.1.A, 5tq4.1.A, 5tq5.1.A, 5tq6.1.A, 5tq7.1.A, 5tqw.1.A, 5tqx.1.A, 5tqy.1.A, 5tr6.1.A, 5ts8.1.A, 5tts.1.A, 5tur.1.A, 5tvt.1.A, 5twl.1.A, 5twy.1.A, 5twy.2.A, 5twz.1.A, 5tx3.2.A, 5u6b.1.A, 5u6b.2.A, 5u6c.1.A, 5u6c.1.B, 5u6y.1.A, 5u6y.1.B, 5u6y.1.C, 5u6y.1.D, 5u6y.1.E, 5u6y.1.F, 5u6y.1.G, 5u6y.1.H, 5u6y.1.I, 5u6y.1.J, 5u6y.1.K, 5u6y.1.L, 5u7q.1.A, 5u7q.2.A, 5u7r.1.A, 5u7r.1.B, 5u7r.2.B, 5u94.1.A, 5u9d.1.A, 5uab.1.A, 5ufu.1.A, 5ugc.1.A, 5ugl.1.A, 5ugx.1.A, 5uhn.1.A, 5ui0.1.A, 5ui0.2.A, 5uiq.1.A, 5uir.1.A, 5uit.1.A, 5uiu.2.A, 5uk8.1.B, 5ukf.1.A, 5ukf.2.A, 5ukk.1.A, 5ukl.1.A, 5ukm.1.A, 5ul1.1.B, 5umo.1.A, 5unp.1.A, 5uor.1.A, 5uor.2.A, 5uox.2.A, 5up3.1.A, 5upk.1.B, 5upl.1.A, 5uq1.1.A, 5uq2.1.A, 5uq3.1.A, 5ur1.2.A, 5usq.1.A, 5usy.1.A, 5uu1.1.A, 5uuu.1.A, 5uv4.1.A, 5uvc.1.A, 5uwd.1.A, 5uxa.2.A, 5uxb.1.A, 5uxb.2.A, 5uxc.1.A, 5uxd.1.A, 5uxd.2.A, 5uy6.1.A, 5v5n.1.A, 5v5y.1.A, 5v60.1.A, 5v61.1.A, 5v62.1.A, 5val.2.A, 5vam.1.A, 5vc3.1.A, 5vc5.1.A, 5vc6.1.A, 5vcv.1.A, 5vcw.1.A, 5vcw.2.A, 5vcx.1.A, 5vcy.1.A, 5vd0.1.A, 5vd1.1.A, 5vd2.1.A, 5vd3.1.A, 5vd4.1.A, 5vd5.1.A, 5vd7.1.A, 5vd8.1.A, 5vd9.1.A, 5vda.1.A, 5vdk.1.A, 5ve6.1.A, 5ved.1.A, 5vee.1.A, 5vef.1.A, 5vib.1.A, 5vio.1.A, 5vio.2.A, 5vio.3.A, 5vio.4.A, 5vja.1.B, 5vja.1.C, 5vlo.1.A, 5vlo.2.A, 5vnd.1.A, 5vnd.2.A, 5vt1.1.A, 5vub.1.A, 5w4w.2.A, 5w5j.1.A, 5w5j.1.B, 5w5o.1.A, 5w7t.1.A, 5w7t.2.A, 5w84.1.A, 5w86.1.A, 5w86.3.A, 5wax.1.A, 5wax.2.A, 5wdy.1.A, 5wdy.2.A, 5we8.1.A, 5we8.2.A, 5wev.1.A, 5wg3.1.A, 5wg5.1.A, 5wij.1.A, 5wjj.1.A, 5wne.1.B, 5wne.2.A, 5wng.1.A, 5wng.1.B, 5wnh.2.A, 5wnh.2.B, 5wni.1.A, 5wno.1.A, 5wp1.1.A, 5wr7.1.A, 5wvd.1.A, 5x02.1.A, 5x17.1.A, 5x17.2.A, 5x18.1.A, 5x28.1.A, 5x2a.1.A, 5x2a.1.B, 5x2f.1.A, 5x2f.2.A, 5x2f.2.B, 5x2k.1.A, 5x3f.1.B, 5x5o.1.A, 5x8i.1.A, 5xd6.1.A, 5xd6.2.A, 5xdl.1.A, 5xff.1.A, 5xfj.1.A, 5xgm.1.A, 5xgn.1.A, 5xgn.1.B, 5xka.1.A, 5xp5.1.A, 5xp7.2.A, 5xqx.1.A, 5xv7.1.A, 5xvf.1.A, 5xvu.1.A, 5xy1.1.A, 5xyx.1.A, 5xyz.1.A, 5xzv.1.A, 5xzv.2.A, 5y04.1.A, 5y25.1.A, 5y5t.1.A, 5y5u.1.A, 5y5u.2.A, 5y7z.1.A, 5y80.1.A, 5y86.1.A, 5y8u.1.A, 5y90.1.A, 5y9m.2.A, 5y9t.1.A, 5ya5.1.A, 5yj9.1.A, 5yjz.1.A, 5yks.1.A, 5yt3.1.A, 5yt3.1.B, 5yt3.2.A, 5yu9.3.A, 5yu9.4.A, 5yva.1.A, 5ywm.1.A, 5z1d.1.A, 5z1e.1.A, 5z33.1.A, 5zan.1.A, 5zj6.1.A, 5zjw.1.A, 5zn0.1.A, 5zn2.1.A, 5zn3.1.A, 5zn4.1.A, 5zn5.1.A, 5zto.1.A, 5zv2.1.A, 5zv2.2.A, 5zwj.1.A, 5zxb.1.A, 5zxb.1.B, 5zz4.1.A, 6a1c.1.A, 6a1f.1.A, 6a1g.1.A, 6a32.1.A, 6aaj.1.A, 6aaj.1.B, 6aak.1.B, 6aak.2.A, 6aam.1.A, 6ac9.1.A, 6ac9.3.A, 6ae3.1.A, 6ae3.2.A, 6agx.1.A, 6agx.1.B, 6ao5.1.A, 6ate.1.A, 6ath.1.A, 6aua.1.A, 6aub.1.A, 6ayd.1.A, 6b1u.1.A, 6b1u.2.A, 6b2e.1.A, 6b2p.1.A, 6b2q.1.A, 6b3e.1.A, 6b4w.1.A, 6b5j.4.A, 6b8j.1.A, 6bab.1.A, 6bab.2.A, 6bbu.1.A, 6bbv.1.A, 6bdl.1.A, 6bdn.1.A, 6bfa.1.A, 6bfn.1.A, 6bfn.2.A, 6bg2.1.A, 6bg2.2.A, 6bg2.3.A, 6bhc.1.A, 6bik.1.A, 6bl8.1.A, 6bl8.2.A, 6ble.1.A, 6bql.1.A, 6bqq.1.A,

6brj.1.A, 6bsd.1.A, 6buu.1.A, 6bwk.1.A, 6bx6.1.A, 6bxi.1.A, 6byr.2.A, 6byr.3.A, 6bys.1.C, 6c0u.1.A, 6c2t.1.B, 6c2y.1.A, 6c3e.1.A, 6c3e.1.B, 6c4d.1.A, 6c4d.2.A, 6c4d.3.A, 6c4d.4.A, 6c7y.1.A, 6c83.1.B, 6c9d.1.A, 6c9f.1.A, 6cad.1.A, 6cad.2.A, 6ccf.1.A, 6ccy.1.A, 6cd6.1.A, 6cd7.1.A, 6cd7.2.A, 6cdt.1.A, 6ch4.1.A, 6cjw.1.A, 6cmj.1.A, 6cn9.1.A, 6cn9.2.A, 6cnh.1.A, 6cpe.1.A, 6cpf.1.A, 6cpg.1.A, 6cpw.1.A, 6cpy.1.A, 6cpy.2.A, 6cqd.1.A, 6cqd.1.B, 6cqe.1.A, 6cqf.1.A, 6cth.1.A, 6ctz.1.A, 6cz3.1.A, 6cz4.1.A, 6d1y.1.A, 6d22.1.A, 6d2i.1.A, 6d3k.1.A, 6d3k.1.B, 6d3k.2.B, 6d3l.1.A, 6d5y.1.A, 6d8e.1.A, 6dc0.1.A, 6dc0.1.B, 6dd4.2.A, 6dfl.1.A, 6dfp.1.A, 6di5.1.A, 6di9.1.A, 6dkb.1.A, 6dkg.1.A, 6dkw.1.A, 6dkw.2.A, 6dmg.1.A, 6dtl.1.A, 6dtl.2.A, 6duk.1.A, 6duk.3.B, 6e0r.1.A, 6e2o.1.A, 6e2o.1.B, 6e4f.1.A, 6e6e.2.A, 6e6e.4.A, 6e6e.6.A, 6e6e.7.A, 6e9w.1.A, 6e9w.1.B, 6eac.1.A, 6eas.1.A, 6ed6.1.A, 6ed6.2.A, 6edl.1.A, 6ef6.1.A, 6eg9.1.A, 6eg9.1.B, 6ega.1.B, 6egd.1.A, 6egf.1.A, 6ehu.1.A, 6ehu.2.A, 6eif.2.A, 6eij.4.A, 6eil.2.A, 6eim.1.A, 6eim.1.B, 6eiq.1.A, 6eiv.2.A, 6eiv.3.A, 6eix.1.A, 6ej4.1.A, 6ej4.2.A, 6ej4.3.A, 6ej4.4.A, 6ek7.1.A, 6ekd.1.A, 6el1.1.S, 6elr.1.A, 6emh.4.A, 6eml.1.4, 6ep9.1.A, 6eq9.1.A, 6eq9.2.A, 6eqi.1.C, 6es0.1.A, 6es0.2.A, 6esa.1.A, 6ewx.1.A, 6ewx.1.B, 6f14.1.A, 6f1w.1.A, 6f1w.2.A, 6f26.1.A, 6f26.2.A, 6f3d.1.A, 6f3e.2.A, 6f3f.1.A, 6f3i.1.A, 6f5e.1.B, 6f7b.1.A, 6fad.1.A, 6fad.3.A, 6fai.1.J, 6fcf.1.A, 6fd3.1.A, 6fdm.1.A, 6fdm.1.B, 6fdn.1.A, 6fdn.1.B, 6fdo.1.A, 6fdo.2.A, 6fdy.1.A, 6fdz.1.A, 6fek.1.A, 6fer.1.A, 6few.1.A, 6fha.1.A, 6fhb.1.A, 6fil.1.A, 6fio.1.A, 6fiq.1.A, 6fjz.1.A, 6fnf.1.A, 6fng.1.A, 6fnh.1.A, 6fnh.2.A, 6fnh.3.A, 6fni.1.A, 6fnj.1.A, 6fnl.1.A, 6fq4.1.A, 6frx.1.A, 6ft7.1.A, 6ft9.3.A, 6fu5.1.B, 6fuc.1.A, 6fvf.1.A, 6fyi.1.A, 6fyk.1.A, 6fyk.2.A, 6fyk.3.A, 6fyl.1.A, 6fyo.1.A, 6fyp.1.A, 6fyr.1.A, 6fyv.1.A, 6g18.1.7, 6g38.1.A, 6g39.1.A, 6g3a.1.A, 6g3c.1.A, 6g4j.1.A, 6g51.1.W, 6g54.1.A, 6g76.1.A, 6g78.1.A, 6g9a.1.A, 6g9n.1.A, 6gcr.1.A, 6gcw.1.A, 6gcw.1.B, 6gcx.1.A, 6ges.1.A, 6ggh.1.A, 6gi6.1.A, 6gin.1.A, 6gjo.2.A, 6gn1.1.A, 6gqj.1.A, 6gqk.1.A, 6gqo.1.A, 6gr8.1.A, 6gr9.1.A, 6gra.1.A, 6gro.1.A, 6gtt.1.A, 6gu2.1.A, 6gu6.1.A, 6gu7.2.A, 6gub.1.A, 6gub.2.A, 6guc.1.A, 6guc.2.A, 6gue.1.A, 6gue.2.A, 6guf.1.A, 6guf.2.A, 6guh.1.A, 6guk.1.A, 6gva.1.A, 6gvj.1.A, 6gvx.1.A, 6gwr.1.A, 6gwr.1.B, 6gzd.1.A, 6gzh.1.A, 6gzm.1.A, 6gzm.1.B, 6h0u.1.B, 6h3k.1.A, 6hbn.2.A, 6hd6.1.A, 6hd6.2.A, 6hdp.1.A, 6hdr.1.A, 6hh1.1.A, 6hhf.1.A, 6hhg.1.A, 6hhh.1.A, 6hho.1.A, 6hho.2.A, 6hjj.1.A, 6hjk.1.A, 6hk3.1.A, 6hk3.2.A, 6hk4.2.A, 6hk6.1.A, 6hk6.2.A, 6hk7.1.A, 6hkm.1.A, 6hkn.1.A, 6hm6.1.A, 6hmd.1.A, 6hme.1.A, 6hmp.1.A, 6hmq.1.A, 6hmr.2.A, 6hmx.1.A, 6hp9.1.A, 6hp9.2.A, 6hv0.1.A, 6hvd.1.A, 6hvf.2.A, 6hwj.1.A, 6hwj.2.A, 6hwk.1.A, 6hwk.1.D, 6hwu.1.A, 6hwv.1.A, 6hx1.1.A, 6hxf.1.A, 6hxf.3.A, 6hzu.2.A, 6hzv.1.A, 6i1s.1.A, 6i2d.1.A, 6i2p.1.A, 6i2u.1.A, 6i2y.1.A, 6i2y.1.B, 6i5i.1.A, 6i82.1.A, 6i82.1.B, 6i83.1.A, 6i8z.1.A, 6i99.1.A, 6i99.2.A, 6ib0.1.A, 6ib2.1.A, 6ig8.1.A, 6iii.1.A, 6il3.1.A, 6ilz.1.A, 6ilz.2.A, 6ilz.3.A, 6ilz.4.A, 6in0.1.A, 6in4.1.A, 6iqn.1.A, 6iqn.1.B, 6ise.1.A, 6itj.2.A, 6itt.1.A, 6itt.1.B, 6itv.1.A, 6iuo.1.A, 6iup.1.A, 6iy9.1.A, 6iy9.2.A, 6j5l.1.A, 6j5t.1.G, 6j5t.1.N, 6j5u.1.B, 6j5u.1.C, 6j5v.1.B, 6j5v.1.C, 6j5w.1.A, 6j6i.1.A, 6j6i.1.B, 6j6m.1.A, 6jgm.1.A, 6jkk.1.A, 6jlr.1.A, 6jmf.1.A, 6joi.1.A, 6joj.1.A, 6jol.1.A, 6jpe.1.A, 6jpj.1.A, 6jqr.1.A, 6jrj.1.A, 6jrk.1.A, 6jrx.1.A, 6jut.1.A, 6jux.1.A, 6jwl.1.A, 6jx0.1.A, 6jx4.1.A, 6jxt.1.A, 6jz0.1.A, 6k0j.1.A, 6k20.1.A, 6k3l.1.A, 6ka4.1.A, 6khd.1.A, 6khd.2.A, 6khd.3.A, 6khe.1.A, 6khf.1.A, 6kla.1.B, 6kmh.1.A, 6kmh.2.A, 6kyq.1.A, 6kyr.1.A, 6kzc.1.A, 6l20.1.A, 6l20.2.A, 6l20.3.A, 6l20.4.A, 6l21.1.A, 6l22.1.A, 6l23.1.A, 6l24.1.A, 6l8l.1.A, 6l8l.2.A, 6l8l.3.A, 6l8l.4.A, 6lba.1.A, 6lk5.1.A, 6lk6.1.A, 6ln1.1.A, 6ln1.2.A, 6lnm.1.A, 6lub.1.A, 6lvk.1.A, 6lvm.1.A, 6lxy.1.A, 6m0u.1.A, 6m13.1.A, 6m7z.1.A, 6m7z.3.A, 6m7z.4.A, 6m7z.5.A, 6m7z.6.A, 6m95.1.A, 6m9h.1.A, 6m9l.1.A, 6mcq.1.A, 6mcq.2.A, 6mep.1.A, 6mep.1.B, 6mib.1.A, 6mm5.1.A, 6mm6.1.A, 6mnh.1.A, 6mny.1.A, 6mny.2.A, 6mob.1.A, 6mom.1.A, 6mwe.2.A, 6mx8.1.A, 6myn.1.A, 6mzw.1.A, 6n0p.1.A, 6n3l.1.A, 6n3n.1.A, 6n3o.1.A, 6n6o.1.A, 6n7a.1.A, 6n7c.2.A, 6n8g.1.A, 6n8g.3.A, 6nbs.1.A, 6ncg.1.A, 6ncg.2.A, 6ne7.1.A, 6nfi.1.A, 6nfy.1.A, 6nfz.1.A, 6nfz.1.B, 6ng0.1.A, 6ng0.1.B, 6no7.1.A, 6no7.2.A, 6no7.2.C, 6no8.1.A, 6npt.1.A, 6npy.1.B, 6npz.1.A, 6npz.2.A, 6nsp.1.A, 6nss.1.A, 6nt9.1.A, 6nvk.1.A, 6nvl.1.A, 6nvl.2.A, 6nvl.3.A, 6nw2.1.A, 6nw2.2.A, 6nyb.1.A, 6nyb.1.B, 6nyh.1.A, 6nyh.2.A, 6nzm.1.A, 6nzr.1.A, 6o5z.1.A, 6o5z.2.A, 6o6q.1.A, 6o6q.1.B, 6o8b.1.C, 6o8b.1.D, 6o8c.1.A, 6o8c.1.B, 6o8i.1.A, 6o8u.1.A, 6o8u.2.A, 6o8u.3.A, 6o94.1.A, 6o94.2.A, 6o94.3.A, 6o9d.2.A, 6o9l.1.3, 6ocq.1.A, 6ocq.2.A, 6ohd.1.A, 6oid.1.A, 6oko.1.A, 6ol2.1.A, 6op9.1.A, 6opg.1.A, 6opi.1.A, 6opk.1.A, 6oqi.1.A, 6oqo.1.A, 6ot6.1.A, 6ots.1.A, 6ova.1.A, 6oyt.1.A, 6oyt.1.B, 6oyw.1.A, 6oyw.1.B, 6p1d.1.A, 6p1d.1.B, 6p1d.2.A, 6p1d.2.B, 6p1l.1.B, 6p1l.2.A, 6p3d.1.A, 6p5m.1.A, 6p5m.3.A, 6p5p.4.A, 6p5s.1.A, 6p68.3.A, 6p69.1.A, 6p7g.1.A, 6p7g.1.B, 6p7g.2.B, 6p8e.1.B, 6p8f.1.B, 6p8g.1.B, 6p8h.1.B, 6p8q.1.A, 6p8q.2.A, 6paw.1.A, 6paw.2.A, 6paw.4.A, 6pdj.1.A, 6pjj.1.C, 6pjx.1.A, 6pk6.1.A, 6pl2.1.A, 6pl3.1.A, 6pma.1.A, 6pme.1.B, 6pme.1.D, 6pme.1.L, 6pnx.1.A, 6pnx.1.B, 6pp9.1.A, 6pp9.1.B, 6pwd.1.A, 6pwg.2.A, 6pxn.1.A, 6pxn.2.A, 6pxo.3.A, 6pxo.3.B, 6pxp.1.A, 6pxp.2.A, 6q0j.1.A, 6q0k.1.A, 6q0k.1.B, 6q0t.1.A, 6q0t.1.B, 6q0t.1.C, 6q2a.1.A, 6q2a.15.A, 6q2a.2.A, 6q2a.7.A, 6q38.1.A, 6q3b.1.A, 6q48.1.A, 6q4d.1.A, 6q4g.1.A, 6q4h.1.A, 6q4i.1.A, 6q4k.1.A, 6q4q.1.A, 6q8k.1.A, 6qaq.1.A, 6qas.1.A, 6qas.2.A, 6qat.1.A, 6qat.2.A, 6qau.1.A, 6qau.2.A, 6qav.1.A, 6qav.2.A, 6qav.2.B, 6qfl.1.A, 6qfr.1.A, 6qmo.1.A, 6qn4.1.A, 6qp5.1.A, 6qtg.1.A, 6qx9.56.A, 6qxk.1.A, 6qy7.1.A, 6qy9.1.A, 6qyx.1.A, 6r3d.1.A, 6r3s.1.A, 6r5f.2.A, 6r5f.3.A, 6r5f.4.A, 6r5k.1.F, 6r5k.1.G, 6ra5.1.A, 6ra7.1.A, 6rbd.1.W, 6rcg.1.A, 6rch.1.A, 6rch.1.B, 6rct.2.A, 6rfi.2.A, 6rfo.1.A, 6rfp.1.A, 6rln.1.A, 6rsb.1.A, 6rsu.1.A, 6ru8.2.A, 6ru8.3.A, 6ruu.1.A, 6ruu.1.B, 6ruu.2.A, 6s11.1.A, 6s11.2.A, 6s1f.1.B, 6s1i.3.A, 6s1i.4.A, 6s1j.1.A, 6s73.1.A, 6s73.2.A, 6s73.3.A, 6s73.4.A, 6s75.1.A, 6s75.2.A, 6s75.3.A, 6s75.4.A, 6s76.1.A, 6s76.2.A, 6s76.3.A, 6s76.4.A, 6s89.1.A, 6s90.1.A, 6s90.2.A, 6s9b.1.A, 6s9c.1.A, 6s9d.1.A, 6s9x.1.A, 6sd9.1.A, 6sdc.1.A, 6sdd.1.A, 6sde.1.A, 6seq.1.A, 6sfk.1.A, 6sfo.1.A, 6sg4.1.A, 6sg4.1.C, 6sgd.1.A, 6so4.1.A, 6soi.1.A, 6sov.1.A, 6spw.1.A, 6srh.1.A, 6srh.2.A, 6sui.1.A, 6sul.1.A, 6sum.1.A, 6sun.1.A, 6t28.1.A, 6t2w.1.A, 6t41.1.A, 6t6a.2.A, 6t6d.3.A, 6t6d.4.A, 6t6f.1.A, 6t6f.2.A, 6t8x.1.A, 6t8x.1.D, 6t8x.1.F, 6tca.1.A, 6tca.1.B, 6tca.2.A, 6tca.2.B, 6tca.3.A, 6tca.3.B, 6tca.4.A, 6tcu.1.A, 6td3.1.B, 6tew.1.A, 6tfp.1.A, 6tfp.2.A, 6tfp.4.A, 6tfu.1.A, 6tfv.2.A, 6tgu.1.A, 6thw.1.A, 6thx.2.A, 6ti8.1.A, 6ti8.4.A, 6tia.1.A, 6tia.2.A, 6tll.1.A, 6tlo.1.A, 6tn9.1.A, 6tnb.1.A, 6tnc.1.A, 6tnd.1.A, 6tpa.1.A, 6tpd.1.A, 6tpe.1.A, 6tpf.1.A, 6tsz.1.A, 6tu9.1.A, 6tua.1.A, 6ty3.1.A, 6ty4.1.A, 6u0k.1.A, 6u2g.1.A, 6u2g.1.B, 6u2h.1.C, 6u2h.1.D, 6u42.439.A, 6u42.440.A, 6u42.441.A, 6u42.442.A, 6u5l.1.A, 6u69.1.A, 6u6a.1.A, 6uan.1.C, 6uan.1.D, 6uip.1.A, 6uip.3.A, 6ul8.1.A, 6umw.1.A, 6una.1.A, 6una.2.A, 6unp.1.A, 6unq.1.A, 6unr.1.A, 6urc.2.A, 6uuo.2.A, 6uuo.2.B, 6v2u.1.A, 6v2u.2.A, 6v2w.1.A, 6v2w.1.B, 6v34.1.A, 6v34.2.A, 6v5n.1.A, 6v5n.1.B, 6v5n.2.A, 6v5p.2.A, 6v66.1.A, 6v66.2.A, 6v6a.1.A, 6v6k.1.B, 6v6o.1.B, 6v6o.3.A, 6v6o.3.B, 6v6o.4.B, 6v6q.1.A, 6vbz.1.A, 6vbz.2.A, 6vc0.1.A, 6vc0.2.A, 6vc0.3.A, 6vg3.1.A, 6vg3.2.A, 6vg3.3.A, 6vgl.1.A, 6vgl.2.A, 6vhg.1.A, 6vnc.1.A, 6vnc.2.A, 6vne.1.A, 6vne.2.A, 6vng.2.A, 6vno.1.A, 6vov.1.A, 6vp6.1.B, 6vp8.1.A, 6vp8.1.B, 6vpg.1.A, 6vph.1.B, 6vpi.1.A, 6vpj.1.A, 6vpl.1.A, 6vpm.1.B, 6vqm.1.A, 6vre.1.A, 6vrf.1.A, 6vru.1.A, 6vvc.1.A, 6vvd.1.A, 6vvd.2.A, 6vvd.3.A, 6vve.1.A, 6vvg.1.A, 6vvg.1.B, 6vxr.1.A, 6vzk.1.A, 6w39.1.B, 6w3a.1.A, 6w3a.1.B, 6w3b.1.A, 6w3c.1.A, 6w3c.2.A, 6w3e.1.A, 6w3k.1.A, 6w4o.1.M, 6w7o.1.A, 6w8i.1.A, 6w8i.2.A, 6w8i.3.A, 6w9e.1.A, 6wa2.1.B, 6wa2.2.A, 6wa2.2.B, 6wak.1.A, 6wak.2.A, 6wak.2.B, 6whp.1.A, 6wjf.1.A, 6wjf.1.B, 6wpp.1.A, 6wpp.2.A, 6wqx.1.B, 6wqx.2.B, 6wtn.1.A, 6wxj.1.A, 6wxn.3.A, 6x5g.1.A, 6x8e.2.A, 6x8g.1.A, 6xag.1.C, 6xbz.1.C, 6xd3.1.C, 6xdb.1.A, 6xdd.1.B, 6xdd.2.A, 6xdf.1.A, 6xdf.1.B, 6xfp.1.A, 6xi8.1.B, 6xih.1.A, 6xih.2.A, 6xka.1.A, 6xl4.1.A, 6xl4.2.A, 6xl4.3.A, 6xl4.4.A, 6xlo.1.A, 6xm1.1.B,

6xr6.1.A, 6xr7.1.A, 6xrg.1.A, 6xv9.2.A, 6xx6.1.A, 6xx8.1.A, 6y23.1.A, 6y23.2.A, 6y4x.1.A, 6y6f.1.A, 6y6v.1.A, 6y7c.1.6, 6y7z.1.A, 6y9r.1.A, 6y9s.2.A, 6ya6.1.A, 6ya7.1.A, 6ya8.1.A, 6yat.1.A, 6yfz.1.A, 6yg0.1.A, 6yg1.1.A, 6yg2.1.A, 6yg3.1.A, 6yg4.1.A, 6yg6.1.A, 6yg6.2.A, 6yg7.1.A, 6ygn.1.A, 6ygn.2.A, 6yi8.1.A, 6yi8.2.A, 6yid.1.B, 6yid.2.A, 6ykd.1.A, 6yky.1.A, 6yky.2.A, 6yky.3.A, 6yky.4.A, 6yl1.1.A, 6ylc.1.A, 6ylk.1.A, 6yll.1.A, 6yll.2.A, 6yoj.1.A, 6yph.1.A, 6yq1.1.A, 6yr9.3.A, 6yt6.1.A, 6yt6.2.A, 6ytd.1.A, 6yty.1.A, 6yul.2.A, 6yvs.2.A, 6yvs.3.A, 6yvs.4.A, 6yvy.1.A, 6yvy.4.A, 6yxs.1.A, 6yxt.1.A, 6yxv.4.A, 6yzh.1.A, 6z19.1.A, 6z1c.1.A, 6z1q.1.A, 6z3u.1.B, 6z45.1.A, 6z4b.1.A, 6z4b.2.A, 6z4y.1.A, 6z50.1.A, 6z53.1.A, 6z55.1.A, 6z59.1.A, 6z6f.1.D, 6z6h.1.C, 6z6o.1.C, 6z6p.1.D, 6z83.1.A, 6z84.2.A, 6zgc.3.A, 6ziw.1.A, 6zjf.1.A, 6znm.1.H, 6znm.1.J, 6zqs.1.A, 6zv6.1.8, 6zxd.1.9, 6zxe.1.a, 6zxf.1.8, 6zxg.1.a, 6zyw.1.A, 7a04.1.A, 7a06.1.B, 7a2a.2.A, 7a49.2.A, 7a4b.1.A, 7a4o.1.A, 7a5b.2.A, 7a6i.1.A, 7aax.1.A, 7aay.1.A, 7aaz.1.A, 7ab1.1.A, 7aem.1.A, 7aj2.1.A, 7ajm.1.A, 7ajm.2.A, 7ajv.1.A, 7ajy.2.A, 7akm.1.A, 7akm.2.A, 7ako.1.A, 7ako.2.A, 7apf.1.A, 7apg.2.A, 7apj.1.A, 7aqb.1.A, 7aqb.2.A, 7at5.2.A, 7at9.1.A, 7ats.1.A, 7atu.1.A, 7atv.1.A, 7avx.1.A, 7avx.2.A, 7avy.1.A, 7aw4.2.A, 7ax4.1.A, 7ayi.1.A, 7aym.1.A, 7b30.1.A, 7b36.1.A, 7b3m.1.A, 7b3q.1.A, 7b3w.1.A, 7b55.1.A, 7b5l.1.J, 7b5q.1.C, 7b6f.1.A, 7b7s.1.A, 7b85.1.A, 7b8h.1.A, 7bcm.1.A, 7be6.1.A, 7bl1.1.C, 7bmk.1.A, 7bmk.1.B, 7btt.1.A, 7bu4.1.A, 7byk.1.A, 7c2v.1.A, 7c2v.1.B, 7c2w.1.A, 7c2w.1.B, 7c2w.1.C, 7c2w.1.D, 7c3n.1.A, 7cbx.1.A, 7cc2.1.A, 7cc2.2.A, 7cga.1.A, 7cga.2.A, 7cga.3.A, 7cga.4.A, 7chm.1.A, 7chn.1.A, 7cht.1.A, 7cja.1.A, 7clh.1.A, 7cmb.1.A, 7cml.1.A, 7cp3.1.A, 7cp4.1.A, 7cqe.1.A, 7ctv.1.A, 7ctv.2.A, 7ctx.1.A, 7cy2.1.A, 7cyr.1.A, 7cz2.1.A, 7d5o.1.A, 7dd1.1.A, 7dg4.1.A, 7dh3.1.A, 7dhv.1.A, 7dl2.1.A, 7dl2.1.D, 7dsy.1.A, 7dt2.1.A, 7dts.1.A, 7dts.1.B, 7dtz.1.A, 7du8.1.B, 7du9.1.A, 7du9.1.B, 7dua.1.A, 7dv6.1.A, 7dxl.1.A, 7e0z.1.A, 7e11.1.A, 7e34.1.A, 7e73.1.A, 7e75.1.A, 7eec.1.A, 7eed.1.A, 7eef.1.A, 7egc.1.I, 7ejv.1.A, 7ejv.1.B, 7elw.1.B, 7emf.1.C, 7ena.2.A, 7ena.56.A, 7enc.24.A, 7enc.3.A, 7er2.1.A, 7f0a.1.A, 7f0b.1.A, 7f2x.1.A, 7f3g.1.A, 7f3g.1.B, 7f3m.1.A, 7f7w.1.A, 7f7w.2.A, 7fcz.1.A, 7fcz.1.B, 7fd0.1.A, 7feh.1.A, 7fhs.1.A, 7fht.4.A, 7fic.1.A, 7jhg.1.A, 7jhh.1.A, 7jij.1.B, 7jnt.1.A, 7jou.1.A, 7jov.4.B, 7ju6.1.B, 7juq.1.B, 7jur.1.B, 7jus.1.B, 7jut.1.A, 7jut.1.B, 7juv.1.A, 7juv.1.B, 7juw.1.A, 7jux.1.A, 7juy.1.B, 7jv1.1.B, 7jv7.1.A, 7jw7.1.A, 7jxh.1.A, 7jxh.5.A, 7jxk.1.A, 7jxl.1.A, 7jxm.1.A, 7jxu.2.A, 7jxx.1.A, 7jxy.1.A, 7jxy.2.A, 7k0v.1.A, 7k0v.1.B, 7k0v.1.C, 7k0v.1.D, 7k1h.1.A, 7k1h.1.B, 7k1h.2.A, 7k1h.2.B, 7k1h.3.B, 7k1i.1.A, 7k5b.1.C, 7k7l.1.A, 7k7z.1.A, 7kac.1.A, 7kac.1.B, 7kek.1.B, 7khj.1.A, 7khj.2.A, 7khk.1.A, 7khk.2.A, 7kia.1.A, 7kja.1.A, 7kja.2.A, 7kjb.1.A, 7kjc.1.A, 7kjc.2.A, 7kjs.1.A, 7kl1.1.A, 7kog.1.A, 7kog.1.B, 7kp6.1.A, 7kp6.2.A, 7kpl.1.A, 7kpv.1.A, 7ksj.1.A, 7kue.1.B, 7kx6.1.A, 7kx6.2.A, 7kx8.1.A, 7kx8.2.A, 7kxl.1.A, 7kxq.1.A, 7kxw.1.A, 7kxw.2.A, 7kxz.1.A, 7kzm.1.6, 7kzm.1.9, 7l24.1.A, 7l24.2.A, 7l25.1.A, 7l26.1.A, 7l26.4.A, 7l5p.1.A, 7l5p.2.A, 7lbm.1.8, 7lbm.1.m, 7lg8.1.A, 7lg8.2.A, 7lg8.3.A, 7lg8.4.A, 7lgs.2.A, 7lht.1.A, 7lhw.1.A, 7li3.1.A, 7ll4.1.A, 7ll5.1.A, 7lqd.1.A, 7lty.1.A, 7lv3.1.A, 7lv3.1.B, 7lvh.1.A, 7lvh.2.A, 7m0k.1.A, 7m0l.1.A, 7m0m.1.A, 7m0m.1.B, 7m5z.1.A, 7m5z.2.A, 7m74.1.A, 7mf0.1.A, 7mfd.1.A, 7mfd.1.B, 7mfe.1.A, 7mff.1.C, 7mff.1.D, 7mgj.1.A, 7mgk.4.A, 7mon.1.A, 7mon.1.B, 7mp8.1.A, 7mt8.1.A, 7mt9.1.A, 7mta.1.A, 7mtb.1.A, 7mu7.1.A, 7mu7.2.A, 7mx3.1.A, 7mx3.2.A, 7mx3.3.A, 7mx3.4.A, 7mxb.1.A, 7mxb.2.A, 7mxj.1.A, 7mxk.1.A, 7myj.2.A, 7n3u.1.A, 7n4r.1.A, 7n85.1.F, 7n85.1.I, 7n85.1.L, 7n85.1.O, 7n8t.1.A, 7n91.1.A, 7n91.2.A, 7n93.2.A, 7n9f.1.F, 7n9f.1.I, 7n9f.1.L, 7n9f.1.O, 7n9g.1.A, 7n9g.2.A, 7naa.1.A, 7nb1.1.A, 7ncf.1.A, 7ng7.1.A, 7nj0.1.B, 7nqw.1.A, 7nry.1.B, 7nti.1.A, 7nur.1.A, 7nvq.1.A, 7nvr.1.e, 7nwk.1.A, 7nxj.1.A, 7nxj.2.A, 7nyx.1.A, 7nyx.1.B, 7nyy.1.A, 7nyy.1.B, 7nyz.1.B, 7nz0.1.A, 7nz0.1.B, 7nz3.1.A, 7o2v.1.A, 7o7i.1.A, 7o7j.1.A, 7o7k.1.A, 7o7k.2.A, 7oai.1.A, 7oam.1.A, 7oam.2.A, 7ogt.1.B, 7oow.1.A, 7oox.1.A, 7opm.1.A, 7opo.1.A, 7opo.4.A, 7opo.5.A, 7ore.1.A, 7orf.1.A, 7ote.1.A, 7ote.2.A, 7ovi.1.A, 7ovj.1.A, 7ovm.1.A, 7oxb.1.A, 7oy5.1.A, 7oy6.1.A, 7ozy.1.A, 7ozy.2.A, 7p1l.1.A, 7p3r.1.A, 7p3r.1.B, 7p3r.1.C, 7p3r.1.D, 7p3v.1.A, 7p3v.1.B, 7p5z.1.M, 7p7f.1.A, 7p7g.1.A, 7p7g.2.A, 7p7h.1.A, 7p7h.2.A, 7pcd.1.A, 7per.1.G, 7per.1.J, 7pi4.1.D, 7pkn.1.A, 7pqv.1.A, 7psu.1.A, 7pt6.1.O, 7pt7.1.G, 7pue.1.A, 7pvu.1.A, 7pvu.2.A, 7pwd.1.A, 7q4a.1.A, 7q4a.1.B, 7q52.1.A, 7q6h.1.A, 7q7i.1.A, 7q7l.1.A, 7q8v.1.A, 7q8y.1.A, 7q8y.2.A, 7qcd.1.A, 7qfm.1.A, 7qg2.1.A, 7qg2.4.A, 7qg3.1.A, 7qg5.2.A, 7qgp.1.A, 7qhw.1.A, 7qhw.2.A, 7qoo.1.B, 7qq6.1.A, 7qq6.1.B, 7qq6.2.A, 7qq6.2.B, 7qr9.3.A, 7que.1.A, 7que.2.A, 7quf.1.A, 7qux.1.A, 7qwk.1.A, 7qwk.5.A, 7qwk.6.A, 7r5k.43.A, 7r5k.49.A, 7r5k.53.A, 7r5k.54.A, 7r5v.1.A, 7r9l.1.B, 7r9n.1.B, 7r9p.1.A, 7r9p.1.B, 7r9t.1.A, 7r9t.1.B, 7ra5.1.A, 7rj8.1.A, 7rn6.1.A, 7run.1.A, 7rwf.1.A, 7s25.1.A, 7s25.1.B, 7s25.2.A, 7s25.2.B, 7s26.1.A, 7s26.1.B, 7s26.2.A, 7s26.2.B, 7s3l.1.A, 7s46.1.A, 7s47.1.A, 7s48.1.A, 7s4t.1.A, 7s6u.1.A, 7s85.1.A, 7sa0.1.A, 7shv.1.A, 7siu.1.A, 7siu.1.B, 7sj3.1.A, 7sqk.1.A, 7sqm.1.A, 7sxf.1.A, 7sxg.1.A, 7sxh.1.A, 7sxj.1.A, 7sza.1.A, 7szc.1.A, 7szd.1.A, 7szr.1.A, 7szr.2.A, 7t0p.1.A, 7t3x.1.A, 7t4i.1.A, 7t4j.1.A, 7t4j.1.B, 7t4k.1.A, 7t4k.1.B, 7t4l.1.B, 7t4m.1.A, 7t4m.1.B, 7t4t.1.A, 7t4t.2.A, 7t4u.1.A, 7t4u.2.A, 7t4v.1.A, 7t4v.2.A, 7t6f.1.A, 7tdz.1.5, 7teu.1.A, 7tnh.1.A, 7tvd.1.A, 7tz7.1.B, 7u2z.1.A, 7u2z.2.A, 7u31.1.A, 7u98.3.A, 7u9a.1.A, 7udp.1.A, 7udq.1.A, 7udq.2.A, 7ug1.1.A, 7ugb.1.A, 7uiq.2.A, 7ujr.1.A, 7ujs.1.A, 7ujt.1.A, 7ukv.1.A, 7ukw.1.A, 7ukw.1.B, 7ukw.2.A, 7ukw.2.B, 7ukz.1.A, 7uos.1.A, 7up4.1.A, 7up4.2.A, 7up6.1.A, 7upm.1.A, 7uxi.1.A, 7uxk.1.A, 7uy0.1.A, 7uy0.2.A, 7uy3.1.A, 7uyr.1.A, 7uyv.1.A, 7uyv.3.A, 7uyw.1.A, 7v3r.1.A, 7v3v.1.M, 7vbt.1.A, 7vdp.1.A, 7vdp.2.A, 7vdq.1.A, 7vf2.1.C, 7vf2.1.D, 7vf5.1.B, 7vf5.1.C, 7vjl.1.A, 7vjl.2.A, 7vkm.1.A, 7vkn.1.A, 7vko.1.A, 7vnx.1.A, 7vop.1.2, 7vop.1.5, 7w15.2.A, 7w19.1.A, 7w19.2.A, 7w1a.1.A, 7w1a.2.A, 7w5c.1.A, 7w5o.1.A, 7w5o.2.A, 7w7x.1.A, 7w7x.1.B, 7w7y.1.A, 7w7y.1.B, 7wcl.1.A, 7wcl.2.A, 7wcw.1.A, 7wcx.1.A, 7wf5.2.A, 7wj6.1.A, 7wj6.1.B, 7wkk.1.G, 7wkk.1.J, 7wkk.1.U, 7wkk.1.X, 7wot.1.J, 7wot.1.S, 7wot.1.V, 7wtt.1.6, 7wu0.1.8, 7x4u.1.A, 7xaf.1.A, 7xbi.1.A, 7xbr.1.A, 7xbr.1.B, 7xbr.1.C, 7xbr.1.D, 7xbr.2.A, 7xbr.2.B, 7xbr.2.C, 7xbr.2.D, 7xdv.1.A, 7xdv.2.A, 7xdx.1.A, 7xdy.1.A, 7xdy.2.A, 7xhn.1.B, 7xho.1.B, 7xmk.1.A, 7xmk.1.B, 7xqk.1.A, 7xsv.1.A, 7xyh.2.A, 7xzq.1.A, 7xzr.1.A, 7xzr.2.A, 7y1g.1.A, 7y1g.2.A, 7y4t.1.A, 7yaw.1.A, 7yaw.2.A, 7yaw.3.A, 7yaz.1.A, 7ybo.1.A, 7yc9.1.A, 7ydx.1.B, 7yl1.1.A, 7ywx.1.A, 7yyh.1.H, 7z1f.1.A, 7z1g.1.A, 7z37.1.C, 7z38.1.C, 7z4v.1.A, 7z5n.1.A, 7z5n.2.A, 7z5n.7.A, 7z5n.8.A, 7z5w.1.A, 7z6i.1.A, 7z9t.1.A, 7z9t.1.B, 7zh8.1.A, 7zh8.3.A, 7zks.1.A, 7zkx.1.A, 7zr0.1.D, 7zr5.1.D, 7zr6.1.D, 7ztl.1.A, 7zvs.1.A, 7zvs.2.A, 7zvs.3.A, 7zw8.1.A, 7zwe.1.A, 7zyn.1.A, 8a27.1.A, 8a2a.1.A, 8a2b.1.A, 8a2d.1.A, 8a5j.1.A, 8a66.1.A, 8a8m.1.A, 8a8m.1.B, 8aau.1.A, 8acm.1.A, 8afr.1.A, 8agy.1.A, 8ahd.1.A, 8ahg.1.A, 8ahp.1.A, 8ak2.1.A, 8am0.1.B, 8an8.1.A, 8ans.1.A, 8aof.1.A, 8arj.1.A, 8atl.1.A, 8atl.2.A, 8atn.1.A, 8atn.2.A, 8au3.1.A, 8au3.2.A, 8au5.1.A, 8auc.1.A, 8auc.2.A, 8av1.1.A, 8aw1.1.A, 8ax8.1.A, 8aza.1.B, 8aza.1.C, 8b8n.1.A, 8ba2.1.A, 8ba3.1.A, 8ba4.1.A, 8bab.1.A, 8bem.1.A, 8bi5.1.A, 8bjt.1.A, 8bju.1.A, 8bm2.1.A, 8bm2.2.A, 8boc.1.A, 8bok.1.A, 8bpv.1.A, 8bpw.1.A, 8bu1.1.B, 8bu1.2.B, 8bu2.1.B, 8bu3.3.B, 8bu4.3.B, 8bu5.1.B, 8bu5.2.B, 8bu5.3.B, 8bu6.1.B, 8bu6.2.B, 8bu6.3.B, 8bu7.1.B, 8bua.1.B, 8bua.2.B, 8bub.1.B, 8bub.2.C, 8bub.3.C, 8bug.3.B, 8bui.1.B, 8bui.2.B, 8buj.3.B, 8bum.1.B, 8buo.1.B, 8bus.1.B, 8bvw.1.I, 8bwy.1.A, 8bx6.1.A, 8bx8.1.C, 8bxc.1.A, 8bya.1.A, 8byq.1.I, 8bzo.1.A, 8bzp.1.A, 8c01.1.3, 8c7x.1.A, 8c7x.1.B, 8c7y.1.A, 8c7y.1.B, 8car.1.A, 8cav.1.A, 8cav.1.B, 8cbj.1.J, 8cdw.1.A, 8cdw.1.B, 8cdy.1.A, 8cgc.1.A, 8cie.1.A, 8cij.1.A, 8cur.1.A, 8d6e.1.A, 8d73.1.A, 8d7m.1.A, 8d7n.2.A, 8d7o.2.A, 8d7p.1.A, 8deg.1.A, 8dgs.1.A, 8dgs.1.B, 8dks.1.A, 8dp5.1.A, 8dp5.1.B, 8ds6.1.A, 8ds6.1.B, 8dso.1.A, 8dsw.1.A, 8dwn.1.A, 8e04.1.A, 8e05.1.A, 8e06.1.A, 8e1x.1.A,

8e1x.1.B, 8e4t.1.A, 8edh.1.A, 8edh.2.A, 8efj.1.A, 8ej4.1.K, 8ejb.1.A, 8ek4.1.A, 8ek4.1.B, 8elc.1.A, 8eme.1.B, 8enj.1.A, 8eq5.1.A, 8eq9.1.A, 8eqd.1.A, 8erd.1.A, 8ewy.1.B, 8ex2.1.A, 8f1w.4.A, 8f1z.1.A, 8f7o.1.A, 8f7o.1.B, 8f7p.1.A, 8f7p.1.B, 8fac.1.A, 8fd9.1.A, 8fe2.1.A, 8fe2.2.A, 8fe5.2.A, 8fe9.1.A, 8fec.1.A, 8fec.2.A, 8ff0.1.A, 8fh4.1.A, 8fh4.2.A, 8fh4.3.A, 8fh4.4.A, 8fjz.1.A, 8fjz.1.B, 8fko.2.A, 8fko.3.A, 8flg.1.A, 8fll.1.A, 8fln.1.A, 8fow.1.A, 8fp1.1.A, 8fp3.1.A, 8fx4.1.D, 8g6z.1.A, 8g8o.1.A, 8gae.1.D, 8gds.2.A, 8gft.1.D, 8gl3.1.A, 8glv.195.A, 8glv.218.A, 8glv.346.A, 8glv.347.A, 8glv.359.A, 8glv.360.A, 8glv.953.A, 8glv.959.A, 8glv.960.A, 8gmb.1.A, 8gmc.1.A, 8gmc.2.A, 8gu7.1.A, 8gu7.2.A, 8guw.1.A, 8guw.1.B, 8guw.1.C, 8gw3.1.A, 8gw3.3.A, 8gxq.48.A, 8h4r.1.A, 8h59.1.A, 8h6p.1.A, 8h75.1.A, 8h75.1.B, 8h75.1.C, 8h75.1.D, 8h7f.1.A, 8h7f.2.A, 8haq.1.A, 8haq.2.A, 8hd0.1.E, 8hlt.1.A, 8hlt.2.A, 8hmt.1.A, 8hmt.2.A, 8ho6.1.A, 8hoa.1.A, 8hod.1.A, 8hoe.1.A, 8hv2.1.A, 8hv4.1.A, 8hv5.1.A, 8hy7.1.A, 8i6o.1.E, 8i7o.48.A, 8i7o.49.A, 8i7o.56.A, 8i7o.59.A, 8i7o.92.A, 8i7r.107.A, 8i7r.108.A, 8i7r.110.A, 8i7r.111.A, 8i7r.113.A, 8i7r.114.A, 8i7r.129.A, 8i7r.132.A, 8i7r.133.A, 8i7r.134.A, 8i7r.136.A, 8i7r.137.A, 8i7r.138.A, 8i7r.139.A, 8i7r.140.A, 8i7r.214.A, 8i7r.215.A, 8i7r.216.A, 8idc.1.C, 8idd.1.D, 8igq.1.A, 8iyj.424.A, 8iyj.425.A, 8iyj.426.A, 8iyj.427.A, 8iyj.428.A, 8iyj.429.A, 8iyj.430.A, 8iyj.431.A, 8iyj.432.A, 8iyj.450.A, 8iyj.451.A, 8iyj.452.A, 8iyj.453.A, 8iyj.454.A, 8iyj.455.A, 8iyj.456.A, 8iyj.457.A, 8iyj.458.A, 8iyj.476.A, 8iyj.477.A, 8iyj.478.A, 8iyj.479.A, 8iyj.480.A, 8iyj.481.A, 8iyj.505.A, 8iyj.506.A, 8iyj.507.A, 8iyj.545.A, 8iyj.546.A, 8iyj.547.A, 8iyj.548.A, 8j07.844.A, 8j07.859.A, 8j07.860.A, 8j07.894.A, 8j07.935.A, 8j07.954.A, 8j07.963.A, 8j5w.1.A, 8j5x.1.A, 8j61.1.A, 8j63.1.A, 8jia.1.C, 8jmx.1.A, 8jpb.1.A, 8k5r.1.A, 8or0.1.G, 8or4.1.G, 8orm.1.C, 8otz.14.A, 8otz.15.A, 8otz.573.A, 8otz.92.A, 8otz.93.A, 8otz.94.A, 8otz.95.A, 8ouv.1.A, 8ov7.1.A, 8ow3.1.A, 8owg.3.A, 8oy2.1.A, 8p08.1.A, 8p4z.1.A, 8p4z.2.A, 8p6v.1.C, 8p7j.1.A, 8p7j.2.A, 8pvp.1.A, 8pyi.1.A, 8pyj.1.A, 8pyk.1.A, 8pyl.1.A, 8q1z.1.A, 8q61.1.A, 8q77.1.A, 8qbu.1.A, 8qcg.1.A, 8qcg.2.A, 8qel.1.B, 8qf1.1.B, 8qlq.1.A, 8qlr.1.A, 8qqy.1.A, 8s93.1.A, 8s99.3.A, 8s9f.1.A, 8s9f.1.B, 8sam.1.A, 8sam.2.A, 8sao.1.B, 8sao.2.B, 8sap.1.A, 8sap.2.A, 8sbc.1.B, 8six.1.A, 8slz.1.B, 8snb.3.A, 8snb.5.A, 8snb.6.A, 8soi.1.A, 8soi.1.B, 8sor.1.D, 8sqz.1.A, 8sqz.1.B, 8srm.1.A, 8srm.1.B, 8srq.1.E, 8ssn.1.A, 8ssn.2.A, 8sso.1.A, 8ssp.1.A, 8stg.1.A, 8stg.2.A, 8t8f.1.C, 8tb5.1.A, 8tdu.1.B, 8tdu.2.B, 8tgd.1.B, 8tj5.1.V, 8tj5.1.Y, 8tj5.1.c, 8to0.398.A, 8to0.402.A, 8to0.415.A, 8to0.460.A, 8to0.461.A, 8ts7.1.B, 8ts9.1.B, 8tsc.1.B, 8txz.1.A, 8tyq.1.A, 8tzb.1.A, 8tzc.1.A, 8tze.1.A, 8tzf.1.A, 8tzg.1.A, 8tzh.1.B, 8u2o.1.A, 8u95.1.A, 8u95.1.B, 8uap.1.A, 8uaq.1.A, 8w6j.1.E, 8wd4.1.A, 8wf4.1.A, 8wf4.2.A, 8wgf.1.A, 8wjy.1.A, 8wsw.4.A, 8wtf.1.A, 8x88.1.A, 8x88.2.A
