# Supplementary material for: Structural and functional characterization of peste des petits ruminants virus coded hemagglutinin protein using various in-silico approaches
Source: Front Microbiol. 2024 Jun 20;15:1427606. doi: 10.3389/fmicb.2024.1427606 (PMC11222573; doi:10.3389/fmicb.2024.1427606)
Supplement: Supplementary file 9 [file Data_Sheet_9.ZIP › IRAK-4.pdf]

# SWISS-MODEL Homology Modelling Report

## Model Building Report

This document lists the results for the homology modelling project "Untitled Project" submitted to SWISS-MODEL workspace on Jan. 8, 2024, 8:30 a.m..The submitted primary amino acid sequence is given in Table T1.

If you use any results in your research, please cite the relevant publications:


- Waterhouse, A., Bertoni, M., Bienert, S., Studer, G., Tauriello, G., Gumienny, R., Heer, F.T., de Beer, T.A.P., Rempfer, C., Bordoli, L., Lepore, R., Schwede, T. SWISS-MODEL: homology modelling of protein structures and complexes. Nucleic Acids Res. 46(W1), W296-W303 (2018).
- Bienert, S., Waterhouse, A., de Beer, T.A.P., Tauriello, G., Studer, G., Bordoli, L., Schwede, T. The SWISS-MODEL Repository - new features and functionality. Nucleic Acids Res. 45, D313-D319 (2017).
- Studer, G., Tauriello, G., Bienert, S., Biasini, M., Johner, N., Schwede, T. ProMod3 - A versatile homology modelling toolbox. PLOS Comp. Biol. 17(1), e1008667 (2021).
- Studer, G., Rempfer, C., Waterhouse, A.M., Gumienny, G., Haas, J., Schwede, T. QMEANDisCo - distance constraints applied on model quality estimation. Bioinformatics 36, 1765-1771 (2020).
- Bertoni, M., Kiefer, F., Biasini, M., Bordoli, L., Schwede, T. Modeling protein quaternary structure of homo- and hetero-oligomers beyond binary interactions by homology. Scientific Reports 7 (2017).


## Results

The SWISS-MODEL template library (SMTL version 2023-12-24, PDB release 2023-12-15) was searched with for evolutionary related structures matching the target sequence in Table T1. For details on the template search, see Materials and Methods. Overall 6706 templates were found (Table T2).

## Models

The following model was built (see Materials and Methods "Model Building"):

| Model #01 | File | Built with | Oligo-State | Ligands | GMQE |
|---|---|---|---|---|---|
| | PDB | ProMod3 3.3.0 | monomer | None | 0.84 |

| Template | Seq Identity | Oligo-state | QSQE | Found by | Method | Resolution | Seq Similarity | Range | Coverage | Description |
|---|---|---|---|---|---|---|---|---|---|---|
| Q9NWZ3.1.A | 90.22 | monomer | - | AFDB search | AlphaFold v2 | - | 0.58 | 1 - 461 | 1.00 | Interleukin-1 receptor-associated kinase 4 |

**The template contained no ligands.**

```
Target     MNKPITASTYVRCLSLGLIRKLSDFIDPQEGWKKLAVAIKKPSGDDRYNQFHIRRFEALLQIGKSPTCELLFDWGTTNCT
Q9NWZ3.1.AMNKPITPSTYVRCLNVGLIRKLSDFIDPQEGWKKLAVAIKKPSGDDRYNQFHIRRFEALLQTGKSPTSELLFDWGTTNCT

Target     VGDLVDILVQNEFFAPASLLLPDAVPKNVNTLPSKVTVVAVQQKPKPLCGKDRTSVISDENPEQNYVLPDSSSPENTSLE
Q9NWZ3.1.AVGDLVDLLIQNEFFAPASLLLPDAVPKTANTLPSK-EAITVQQKQMPFCDKDRTLMTPVQNLEQSYMPPDSSSPENKSLE

Target     FSDTRFHSFSFFELKDVTNNFDERPISVGGNKMGEGGFGVVYKGYVNNRTVAVKKLAAMVDISTEELKQQFDQEIKVMAK
Q9NWZ3.1.AVSDTRFHSFSFYELKNVTNNFDERPISVGGNKMGEGGFGVVYKGYVNNTTVAVKKLAAMVDITTEELKQQFDQEIKVMAK

Target     CQHENLVELLGFSSDGDDLCLVYVYMPNGSLLDRLSCLDGTPPLSWNMRCKIAQGAANGLSYLHENHHIHRDIKSANILL
Q9NWZ3.1.ACQHENLVELLGFSSDGDDLCLVYVYMPNGSLLDRLSCLDGTPPLSWHMRCKIAQGAANGINFLHENHHIHRDIKSANILL

Target     DEDFTAKISDFGLARASEKFAQTVMTSRIVGTTAYMAPEALRGEITPKSDIYSFGVVLLEIITGLPAVDEHREPQLLLDI
Q9NWZ3.1.ADEAFTAKISDFGLARASEKFAQTVMTSRIVGTTAYMAPEALRGEITPKSDIYSFGVVLLEIITGLPAVDEHREPQLLLDI

Target     KEEIEDEEKTIEDYVDRKMNDIDSTSIETMYSVASQCLHEKKNKRPDIKKVQQLLEEMTGS
```

```
Q9NWZ3.1.AKEEIEDEEKTIEDYIDKKMNDADSTSVEAMYSVASQCLHEKKNKRPDIKKVQQLLQEMTAS
```

---

## Materials and Methods

### Template Search

Template search with has been performed against the SWISS-MODEL template library (SMTL, last update: 2023-12-24, last included PDB release: 2023-12-15).

### Model Building

Models are built based on the target-template alignment using ProMod3 (Studer et al.). Coordinates which are conserved between the target and the template are copied from the template to the model. Insertions and deletions are remodelled using a fragment library. Side chains are then rebuilt. Finally, the geometry of the resulting model is regularized by using a force field.

### Model Quality Estimation

The global and per-residue model quality has been assessed using the QMEAN scoring function (Studer et al.).

### Ligand Modelling

Ligands present in the template structure are transferred by homology to the model when the following criteria are met: (a) The ligands are annotated as biologically relevant in the template library, (b) the ligand is in contact with the model, (c) the ligand is not clashing with the protein, (d) the residues in contact with the ligand are conserved between the target and the template. If any of these four criteria is not satisfied, a certain ligand will not be included in the model. The model summary includes information on why and which ligand has not been included.

### Oligomeric State Conservation

The quaternary structure annotation of the template is used to model the target sequence in its oligomeric form. The method (Bertoni et al.) is based on a supervised machine learning algorithm, Support Vector Machines (SVM), which combines interface conservation, structural clustering, and other template features to provide a quaternary structure quality estimate (QSQE). The QSQE score is a number between 0 and 1, reflecting the expected accuracy of the interchain contacts for a model built based a given alignment and template. Higher numbers indicate higher reliability. This complements the GMQE score which estimates the accuracy of the tertiary structure of the resulting model.

### References


- **BLAST**
  Camacho, C., Coulouris, G., Avagyan, V., Ma, N., Papadopoulos, J., Bealer, K., Madden, T.L. BLAST+: architecture and applications. BMC Bioinformatics 10, 421-430 (2009).
- **HHblits**
  Steinegger, M., Meier, M., Mirdita, M., Vöhringer, H., Haunsberger, S. J., Söding, J. HH-suite3 for fast remote homology detection and deep protein annotation. BMC Bioinformatics 20, 473 (2019).


### Table T1:

Primary amino acid sequence for which templates were searched and models were built.

```
MNKPITASTYVRCLSLGLIRKLSDFIDPQEGWKKLAVAIKKPSGDDRYNQFHIRRFEALLQIGKSPTCELLFDWGTTNCTVGDLVDILVQNEFFAPASLL
LPDAVPKNVNTLPSKVTVVAVQQKPKPLCGKDRTSVISDENPEQNYVLPDSSSPENTSLEFSDTRFHSFSFFELKDVTNNFDERPISVGGNKMGEGGFGV
VYKGYVNNRTVAVKKLAAMVDISTEELKQQFDQEIKVMAKCQHENLVELLGFSSDGDDLCLVYVYMPNGSLLDRLSCLDGTPPLSWNMRCKIAQGAANGL
SYLHENHHIHRDIKSANILLDEDFTAKISDFGLARASEKFAQTVMTSRIVGTTAYMAPEALRGEITPKSDIYSFGVVLLEIITGLPAVDEHREPQLLLDI
KEEIEDEEKTIEDYVDRKMNDIDSTSIETMYSVASQCLHEKKNKRPDIKKVQQLLEEMTGS
```

### Table T2:

| Template | Seq Identity | Oligo-state | QSQE | Found by | Method | Resolution | Seq Similarity | Coverage | Description |
|---|---|---|---|---|---|---|---|---|---|
| Q9NWZ3.1.A | 90.22 | monomer | - | AFDB search | AlphaFold v2 | NA | 0.58 | 1.00 | Interleukin-1 receptor-associated kinase 4 |

| Template | Seq Identity | Oligo-state | QSQE | Found by | Method | Resolution | Seq Similarity | Coverage | Description |
|---|---|---|---|---|---|---|---|---|---|
| 8dks.1.A | 90.22 | monomer | - | BLAST | X-ray | 2.45Å | 0.58 | 1.00 | Interleukin-1 receptor-associated kinase 4 |
| 8dks.1.A | 90.22 | monomer | - | HHblits | X-ray | 2.45Å | 0.58 | 1.00 | Interleukin-1 receptor-associated kinase 4 |
| 5uiu.2.A | 94.14 | monomer | - | HHblits | X-ray | 2.02Å | 0.59 | 0.67 | Interleukin-1 receptor-associated kinase 4 |
| 5uit.1.A | 94.14 | monomer | - | HHblits | X-ray | 1.84Å | 0.59 | 0.67 | Interleukin-1 receptor-associated kinase 4 |
| 6thx.2.A | 94.14 | monomer | - | HHblits | X-ray | 1.99Å | 0.59 | 0.67 | Interleukin-1 receptor-associated kinase 4 |
| 6thw.1.A | 94.14 | monomer | - | HHblits | X-ray | 2.44Å | 0.59 | 0.67 | Interleukin-1 receptor-associated kinase 4 |
| 5uiq.1.A | 94.14 | monomer | - | HHblits | X-ray | 2.64Å | 0.59 | 0.67 | Interleukin-1 receptor-associated kinase 4 |
| 5uir.1.A | 94.14 | monomer | - | HHblits | X-ray | 2.64Å | 0.59 | 0.67 | Interleukin-1 receptor-associated kinase 4 |
| 6d8e.1.A | 22.31 | monomer | - | HHblits | X-ray | 2.54Å | 0.31 | 0.56 | Epidermal growth factor receptor |
| 7zyn.1.A | 23.08 | monomer | - | HHblits | X-ray | 2.30Å | 0.32 | 0.56 | Epidermal growth factor receptor |
| 3bht.2.A | 27.03 | monomer | - | HHblits | X-ray | 2.00Å | 0.33 | 0.56 | Cell division protein kinase 2 |
| 5cav.1.A | 22.31 | monomer | - | HHblits | X-ray | 2.73Å | 0.31 | 0.56 | Epidermal growth factor receptor |
| 3lff.1.A | 23.19 | monomer | - | HHblits | X-ray | 1.50Å | 0.32 | 0.57 | Mitogen-activated protein kinase 14 |
| 3ika.1.A | 22.31 | monomer | - | HHblits | X-ray | 2.90Å | 0.31 | 0.56 | Epidermal growth factor receptor |
| 6s89.1.A | 23.08 | monomer | - | HHblits | X-ray | 2.70Å | 0.32 | 0.56 | Epidermal growth factor receptor |
| 7ztl.1.A | 25.79 | monomer | - | HHblits | X-ray | 1.90Å | 0.33 | 0.55 | Aurora kinase A |
| 3gcq.1.A | 23.19 | monomer | - | HHblits | X-ray | 2.00Å | 0.32 | 0.57 | Mitogen-activated protein kinase 14 |
| 4bcn.1.A | 27.03 | monomer | - | HHblits | X-ray | 2.10Å | 0.33 | 0.56 | CYCLIN-DEPENDENT KINASE 2 |
| 3bht.1.A | 27.03 | monomer | - | HHblits | X-ray | 2.00Å | 0.33 | 0.56 | Cell division protein kinase 2 |
| 3gcv.1.A | 23.19 | monomer | - | HHblits | X-ray | 2.30Å | 0.32 | 0.57 | Mitogen-activated protein kinase 14 |
| 4wd5.1.A | 22.31 | monomer | - | HHblits | X-ray | 3.30Å | 0.31 | 0.56 | Epidermal growth factor receptor |
| 3gcp.1.A | 23.19 | monomer | - | HHblits | X-ray | 2.25Å | 0.32 | 0.57 | Mitogen-activated protein kinase 14 |
| 3ika.3.B | 22.31 | homo-dimer | - | HHblits | X-ray | 2.90Å | 0.31 | 0.56 | Epidermal growth factor receptor |
| 6i2u.1.A | 25.79 | monomer | - | HHblits | X-ray | 2.50Å | 0.33 | 0.55 | Aurora kinase A |
| 3hub.1.A | 23.19 | monomer | - | HHblits | X-ray | 2.25Å | 0.32 | 0.57 | Mitogen-activated protein kinase 14 |
| 3lfb.1.A | 23.19 | monomer | - | HHblits | X-ray | 2.60Å | 0.32 | 0.57 | Mitogen-activated protein kinase 14 |
| 3l8s.1.A | 23.19 | monomer | - | HHblits | X-ray | 2.35Å | 0.32 | 0.57 | Mitogen-activated protein kinase 14 |
| 2x6e.1.A | 25.79 | monomer | - | HHblits | X-ray | 3.35Å | 0.33 | 0.55 | SERINE/THREONINE-PROTEIN KINASE 6 |
| 7nvq.1.A | 27.27 | monomer | - | HHblits | X-ray | 2.05Å | 0.33 | 0.57 | Cyclin-dependent kinase 2 |

| Template | Seq Identity | Oligo-state | QSQE | Found by | Method | Resolution | Seq Similarity | Coverage | Description |
|---|---|---|---|---|---|---|---|---|---|
| 5k4j.1.A | 27.27 | monomer | - | HHblits | X-ray | 1.60Å | 0.33 | 0.57 | Cyclin-dependent kinase 2 |
| 3poz.1.A | 22.31 | monomer | - | HHblits | X-ray | 1.50Å | 0.31 | 0.56 | Epidermal growth factor receptor |
| 5one.1.A | 25.79 | monomer | - | HHblits | X-ray | 2.60Å | 0.33 | 0.55 | Aurora kinase A |
| 7ra5.1.A | 27.27 | monomer | - | HHblits | X-ray | 1.67Å | 0.33 | 0.57 | Cyclin-dependent kinase 2 |
| 3lfd.1.A | 23.19 | monomer | - | HHblits | X-ray | 3.40Å | 0.32 | 0.57 | Mitogen-activated protein kinase 14 |
| 4zts.1.A | 25.79 | monomer | - | HHblits | X-ray | 2.90Å | 0.33 | 0.55 | Aurora kinase A |
| 6gra.1.A | 25.79 | monomer | - | HHblits | X-ray | 2.60Å | 0.33 | 0.55 | Aurora kinase A |
| 4ztr.1.A | 25.79 | monomer | - | HHblits | X-ray | 2.85Å | 0.33 | 0.55 | Aurora kinase A |
| 8oy2.1.A | 27.27 | monomer | - | HHblits | X-ray | 2.62Å | 0.33 | 0.57 | Cyclin-dependent kinase 2 |
| 3ezv.1.A | 27.03 | monomer | - | HHblits | X-ray | 1.99Å | 0.33 | 0.56 | Cell division protein kinase 2 |
| 6z4y.1.A | 25.79 | monomer | - | HHblits | X-ray | 2.25Å | 0.33 | 0.55 | Aurora kinase A |
| 4eok.1.A | 37.91 | monomer | - | BLAST | X-ray | 2.57Å | 0.39 | 0.39 | Cyclin-dependent kinase 2 |
| 4eoj.2.A | 37.91 | monomer | - | BLAST | X-ray | 1.65Å | 0.39 | 0.39 | Cyclin-dependent kinase 2 |
| 4eoj.1.A | 37.91 | monomer | - | BLAST | X-ray | 1.65Å | 0.39 | 0.39 | Cyclin-dependent kinase 2 |
| 4eoi.1.A | 37.91 | monomer | - | BLAST | X-ray | 2.00Å | 0.39 | 0.39 | Cyclin-dependent kinase 2 |
| 4eoi.2.A | 37.91 | monomer | - | BLAST | X-ray | 2.00Å | 0.39 | 0.39 | Cyclin-dependent kinase 2 |
| 4eok.2.A | 37.91 | monomer | - | BLAST | X-ray | 2.57Å | 0.39 | 0.39 | Cyclin-dependent kinase 2 |
| 4eom.1.A | 37.91 | monomer | - | BLAST | X-ray | 2.10Å | 0.39 | 0.39 | Cyclin-dependent kinase 2 |
| 4eom.2.A | 37.91 | monomer | - | BLAST | X-ray | 2.10Å | 0.39 | 0.39 | Cyclin-dependent kinase 2 |
| 1wh4.1.A | 84.96 | monomer | - | BLAST | NMR | NA | 0.57 | 0.25 | interleukin-1 receptor-associated kinase 4 |

The table above shows the top 50 filtered templates. A further 5,518 templates were found which were considered to be less suitable for modelling than the filtered list.
1a06.1.A, 1a9u.1.A, 1ad5.1.A, 1apm.1.A, 1aq1.1.A, 1b39.1.A, 1b6c.5.D, 1bi7.1.A, 1bi8.1.A, 1bkx.1.A, 1bl7.1.A, 1blx.1.A, 1bmk.1.A, 1buh.1.A, 1bx6.1.A, 1byg.1.A, 1cdk.1.A, 1ckj.1.A, 1ckj.1.B, 1cm8.1.A, 1cmk.1.A, 1csn.1.A, 1ctp.1.A, 1d2z.1.A, 1d2z.1.B, 1d2z.2.A, 1d2z.2.B, 1di8.1.A, 1di9.1.A, 1ds5.1.A, 1e7u.1.A, 1e8w.1.A, 1e8x.1.A, 1e8y.1.A, 1e9h.1.A, 1eh4.1.A, 1eh4.2.A, 1f0q.1.A, 1f3m.1.D, 1f5q.1.A, 1f5q.2.A, 1fgi.1.A, 1fin.1.A, 1fin.2.A, 1fmk.1.A, 1fot.1.A, 1fpu.1.A, 1fq1.1.B, 1fvr.1.A, 1fvv.1.A, 1g3n.4.C, 1g5s.1.A, 1gih.1.A, 1gij.1.A, 1gjo.1.A, 1gng.1.A, 1gol.1.A, 1gzk.1.A, 1gzn.1.A, 1h01.1.A, 1h1p.2.A, 1h1q.1.A, 1h1q.2.A, 1h1w.1.A, 1h25.2.A, 1h27.1.A, 1h28.2.A, 1h4l.1.A, 1h8f.1.A, 1hcl.1.A, 1he8.1.A, 1how.1.A, 1i09.1.A, 1i44.1.A, 1ia8.1.A, 1ias.1.A, 1ias.1.B, 1ik7.1.A, 1ik7.2.A, 1ir3.1.C, 1irk.1.A, 1j1b.1.A, 1j1b.1.B, 1j3h.1.A, 1j7i.1.A, 1j7u.1.A, 1j7u.1.B, 1j91.1.A, 1j91.2.A, 1jbp.1.A, 1jks.1.A, 1jkt.1.A, 1jkt.2.A, 1jnk.1.A, 1jow.1.B, 1jpa.1.A, 1jqh.2.A, 1jst.1.A, 1jst.1.C, 1jsu.1.A, 1jvp.1.A, 1jwh.1.A, 1jwh.1.B, 1k2p.1.A, 1k2p.1.B, 1k3a.1.A, 1k9a.1.A, 1k9a.2.A, 1k9a.3.A, 1k9a.5.A, 1k9a.6.A, 1koa.1.A, 1ksw.1.A, 1kv1.1.A, 1kwp.3.B, 1kwp.3.C, 1l8t.1.A, 1lew.1.A, 1lp4.1.A, 1luf.1.A, 1m14.1.A, 1m17.1.A, 1m2p.1.A, 1m2q.1.A, 1m2r.1.A, 1m52.1.A, 1m7n.1.A, 1mp8.1.A, 1mq4.1.A, 1mqb.1.A, 1mqb.2.A, 1mru.1.A, 1mru.2.A, 1mrv.1.A, 1muo.1.A, 1na7.1.A, 1nd4.1.A, 1ngr.1.A, 1nw1.1.A, 1nxk.1.A, 1ny3.2.C, 1o6k.1.A, 1o6l.1.A, 1o6y.1.A, 1o9u.1.A, 1oi9.2.A, 1oit.1.A, 1okv.2.A, 1oky.1.A, 1ol2.1.A, 1ol5.1.A, 1ol6.1.A, 1ol7.1.A, 1omw.1.A, 1opk.1.A, 1opl.1.A, 1opl.2.A, 1ouk.1.A, 1oz1.1.A, 1p14.1.A, 1p2a.1.A, 1p4f.1.A, 1p4o.1.A, 1p5e.1.A, 1pf8.1.A, 1phk.1.A, 1pjk.1.A, 1pkg.1.A, 1pkg.2.A, 1pme.1.A, 1pmn.1.A, 1pmu.1.A, 1pmv.1.A, 1pxo.1.A, 1py5.1.A, 1pyx.1.A, 1pyx.1.B, 1q24.1.A, 1q5k.1.A, 1q5k.1.B, 1q8w.1.A, 1q8y.1.A, 1q8y.2.A, 1qcf.1.A, 1ql6.1.A, 1qmz.1.A, 1qpd.1.A, 1qpe.1.A, 1r0e.1.A, 1r0p.1.A, 1r39.1.A, 1r3c.1.A, 1r78.1.A, 1rdq.1.A, 1re8.1.A, 1rek.1.A, 1rjb.1.A, 1rqq.1.A, 1rw8.1.A, 1s9i.1.A, 1s9i.1.B, 1s9j.1.A, 1sm2.1.A, 1sm2.2.A, 1smh.1.A, 1snu.2.A, 1stc.1.A, 1syk.1.A, 1szm.1.A, 1szm.2.A, 1t45.1.A, 1t46.1.A, 1tki.1.A, 1tqi.1.A, 1tvo.1.A, 1u46.1.A, 1u46.2.A, 1u54.1.A, 1u54.2.A, 1u59.1.A, 1u5q.1.A, 1u5q.2.A, 1u5r.2.A, 1u5r.3.B, 1ua2.1.A, 1ua2.2.A, 1ua2.3.A, 1ua2.4.A, 1ukh.1.A, 1uki.1.A, 1ung.2.A, 1unh.1.A, 1uu7.1.A, 1uu9.1.A, 1uv5.1.A, 1uwh.1.A, 1uwj.1.A, 1v0b.1.A, 1v0o.1.A, 1v0o.2.A, 1v0p.1.A, 1v0p.2.A, 1v1k.1.A, 1vjy.1.A, 1vr2.1.A, 1vyw.1.A, 1vyw.2.A, 1vzo.1.A, 1w7h.1.A, 1w82.1.A, 1w98.1.A, 1wak.1.A, 1wbo.1.A, 1wbv.1.A, 1wbw.1.A, 1wh4.1.A, 1wmg.1.A, 1wmg.1.B, 1wmg.1.C, 1wmg.1.D, 1wmg.1.E, 1wmg.1.F, 1wmk.1.A, 1wvw.1.A, 1wvx.1.A,

1wvy.1.A, 1wxp.1.A, 1wzy.1.A, 1x8b.1.A, 1xh4.1.A, 1xh6.1.A, 1xh9.1.A, 1xjd.1.A, 1xkk.1.A, 1xr1.1.A, 1xws.1.A, 1y57.1.A, 1y6a.1.A, 1y8g.1.A, 1ydt.1.A, 1ygo.1.A, 1yhv.1.A, 1yhw.1.A, 1yi3.1.A, 1yi4.1.A, 1yi6.1.A, 1yi6.2.A, 1ym7.2.A, 1yoj.1.A, 1yol.1.A, 1yol.2.A, 1yom.2.A, 1yqj.1.A, 1yrp.1.A, 1yvj.1.A, 1yw2.1.A, 1ywn.1.A, 1ywr.1.A, 1yxs.1.A, 1yxu.1.A, 1z9x.1.A, 1zlt.1.A, 1zmu.1.A, 1zmv.1.A, 1zoe.1.A, 1zog.1.A, 1zp9.1.A, 1zp9.3.A, 1zp9.4.A, 1zrz.1.A, 1ztf.1.A, 1zws.1.A, 1zxe.1.A, 1zxe.1.B, 1zxe.3.A, 1zxe.3.B, 1zy5.1.A, 1zy5.1.B, 1zyc.1.A, 1zyc.1.B, 1zyc.2.A, 1zyc.2.B, 1zyd.1.A, 1zyj.1.A, 1zyl.1.A, 1zys.1.A, 1zz2.1.A, 2a0c.1.A, 2a19.1.B, 2a19.1.C, 2a1a.1.B, 2a27.1.A, 2a2a.1.A, 2a4z.1.A, 2a5u.1.A, 2a9i.1.A, 2ac3.1.A, 2ac5.1.A, 2acx.1.A, 2auh.1.A, 2ayp.1.A, 2b0q.1.A, 2b1p.1.A, 2b4s.1.B, 2b52.1.A, 2b53.1.A, 2b54.1.A, 2b7a.1.A, 2b7a.2.A, 2b9f.1.A, 2b9h.1.A, 2baj.1.A, 2bak.1.A, 2bal.1.A, 2baq.1.A, 2bcj.1.A, 2bdf.1.A, 2bdw.1.A, 2bdw.1.B, 2bfy.1.A, 2bfy.2.A, 2biy.1.A, 2bkk.1.A, 2bkk.2.A, 2bmc.1.A, 2bts.1.A, 2bva.1.A, 2bva.2.A, 2c1b.1.A, 2c30.1.A, 2c3j.1.A, 2c47.1.A, 2c6d.1.A, 2c6e.1.A, 2c6e.2.A, 2cch.1.A, 2cdz.1.A, 2cgw.1.A, 2chl.1.A, 2chw.1.A, 2chx.1.A, 2cjm.2.A, 2cko.1.A, 2ckp.1.A, 2ckp.1.B, 2ckq.1.A, 2clq.1.A, 2cmw.1.A, 2cn5.1.A, 2cn8.1.B, 2cpk.1.A, 2csn.1.A, 2d96.1.A, 2dbf.1.A, 2dq7.1.A, 2dwb.1.A, 2dyl.1.A, 2e2b.1.A, 2e9p.1.A, 2e9v.1.A, 2eb2.1.A, 2eb3.1.A, 2erk.1.A, 2erz.1.A, 2etm.1.A, 2etm.2.A, 2eu9.1.A, 2euf.1.B, 2eva.1.A, 2ewa.1.A, 2exc.1.A, 2exe.1.A, 2exm.1.A, 2f2c.1.B, 2f2u.1.A, 2f2u.2.A, 2f49.3.A, 2f4j.1.A, 2f57.1.A, 2f57.2.A, 2fb8.1.A, 2fh9.1.A, 2fo0.1.A, 2fsl.1.A, 2fso.1.A, 2fst.1.A, 2fum.1.A, 2fum.3.A, 2fvd.1.A, 2fys.1.A, 2fys.2.A, 2g01.1.A, 2g01.2.A, 2g15.1.A, 2g1t.4.A, 2g2f.1.A, 2g2f.2.A, 2g2h.1.A, 2g2h.2.A, 2g2i.1.A, 2g2i.2.A, 2g7r.1.A, 2gcd.1.A, 2gcd.2.A, 2gdo.1.A, 2gfc.1.A, 2gfs.1.A, 2ghl.1.A, 2ghm.1.A, 2gmx.1.A, 2gmx.2.A, 2gnf.1.A, 2gnj.1.A, 2gph.1.A, 2gqg.1.A, 2gqg.2.A, 2gs6.1.C, 2gs7.1.A, 2gsf.1.A, 2gtm.1.A, 2gu8.1.A, 2h34.1.A, 2h34.2.A, 2h6d.1.A, 2h8h.1.A, 2h96.1.A, 2h96.2.A, 2h9v.1.A, 2hak.1.A, 2hak.2.A, 2hak.3.A, 2hak.6.A, 2hak.7.A, 2hck.1.B, 2hel.1.A, 2hen.1.A, 2hiw.1.A, 2hiw.2.A, 2hk5.1.A, 2hog.1.A, 2hw6.1.A, 2hw6.2.A, 2hw7.1.A, 2hwo.1.A, 2hwp.2.A, 2hxq.1.A, 2hyy.3.A, 2hz0.1.A, 2hz0.2.A, 2hz4.1.A, 2hz4.2.A, 2hz4.3.A, 2hzi.1.A, 2hzi.2.A, 2hzn.1.A, 2i0e.1.A, 2i0v.1.A, 2i0y.1.A, 2i1m.1.A, 2i40.2.A, 2i7q.1.A, 2ib1.1.A, 2ig7.1.A, 2ijm.1.A, 2in6.1.A, 2itn.1.A, 2itp.1.A, 2itx.1.A, 2itz.1.A, 2ivu.1.A, 2ivv.1.A, 2iw6.1.A, 2iw8.1.A, 2iwi.1.A, 2izs.1.A, 2j0i.1.A, 2j0j.1.A, 2j0k.1.A, 2j0k.2.A, 2j0l.1.A, 2j0m.1.B, 2j2i.1.A, 2j4z.1.A, 2j4z.2.A, 2j50.1.A, 2j5e.1.A, 2j5f.1.A, 2j90.1.A, 2j90.1.B, 2j9m.1.A, 2jam.1.A, 2jav.1.A, 2jbo.1.A, 2jbp.1.A, 2jbp.10.A, 2jc6.1.A, 2jed.1.A, 2jed.2.A, 2jfm.1.B, 2jgz.1.A, 2jii.1.A, 2jit.1.A, 2jit.2.A, 2jiu.1.A, 2jiu.2.A, 2jiv.1.A, 2jiv.2.A, 2jkm.1.A, 2jko.1.A, 2jkq.1.A, 2kty.1.A, 2kul.1.A, 2lav.1.A, 2lgc.1.A, 2n80.1.A, 2n83.1.A, 2n97.1.A, 2n97.1.B, 2no3.2.A, 2np8.1.A, 2npq.1.A, 2nru.1.A, 2nru.2.A, 2nru.3.A, 2nru.4.A, 2nry.1.A, 2nry.2.A, 2nry.3.A, 2nry.4.A, 2o5k.1.A, 2o8y.1.A, 2ofu.1.A, 2ofv.1.A, 2ofv.1.B, 2og8.1.A, 2ogv.1.A, 2oh4.1.A, 2oib.1.A, 2oib.2.A, 2oib.3.A, 2oic.2.A, 2oic.3.A, 2oid.1.A, 2oid.2.A, 2oid.3.A, 2oid.4.A, 2oiq.1.A, 2oj9.1.A, 2ojf.1.A, 2ojg.1.A, 2ojj.1.A, 2ok1.1.A, 2onl.1.B, 2onl.2.A, 2onl.2.B, 2ow3.1.A, 2owb.2.B, 2oxy.1.A, 2oza.1.A, 2oza.1.B, 2ozo.1.A, 2p2h.1.A, 2p2i.1.A, 2p3g.1.A, 2p4i.1.A, 2p4i.2.A, 2p55.1.B, 2pe2.1.A, 2phk.1.A, 2pk9.2.A, 2pl0.1.A, 2pml.1.A, 2pmo.1.A, 2ppq.1.A, 2psq.1.A, 2ptk.1.A, 2pup.1.A, 2puu.1.A, 2pvf.1.A, 2pvj.1.A, 2pvy.1.B, 2pwl.1.A, 2py3.1.A, 2pyw.1.A, 2pz5.1.A, 2pzi.1.A, 2pzi.2.A, 2pzp.1.A, 2pzr.1.A, 2pzy.1.A, 2pzy.2.A, 2pzy.3.A, 2pzy.4.A, 2q0b.1.A, 2q0n.1.A, 2q83.1.A, 2qd9.1.A, 2qg5.1.A, 2qg5.2.A, 2qg7.1.A, 2qg7.1.B, 2qg7.2.A, 2qg7.2.B, 2qhm.1.A, 2qi8.1.A, 2qkr.1.A, 2qkw.1.B, 2qlq.1.A, 2qlq.2.A, 2qlu.1.A, 2qnj.1.A, 2qnj.1.B, 2qoc.1.A, 2qod.1.A, 2qof.1.A, 2qoh.1.A, 2qoi.1.A, 2qok.1.A, 2qol.1.A, 2qon.1.A, 2qoo.1.A, 2qu5.1.A, 2qu6.1.A, 2qur.1.A, 2qvs.1.A, 2r0i.1.A, 2r2p.1.A, 2r3n.1.A, 2r4b.1.A, 2r64.1.A, 2r7i.1.A, 2r9s.1.A, 2rd0.1.A, 2rei.1.A, 2rf9.1.A, 2rf9.2.A, 2rfd.1.A, 2rfd.2.A, 2rfe.2.A, 2rfe.4.A, 2rfn.1.A, 2rfs.1.A, 2rg5.1.A, 2rg6.1.A, 2rgp.1.A, 2rio.1.A, 2rku.1.A, 2rl5.1.A, 2rsv.1.A, 2src.1.A, 2uzb.2.A, 2uzd.1.A, 2uzl.1.A, 2uzv.1.B, 2v55.1.A, 2v5q.1.A, 2v5q.2.A, 2v62.1.A, 2v7a.1.A, 2vd5.1.A, 2vn9.1.A, 2vo6.1.A, 2vrx.1.A, 2vrx.2.A, 2vth.1.A, 2vtn.1.A, 2vtt.1.A, 2vuw.1.A, 2vwb.1.A, 2vwb.2.A, 2vwi.1.A, 2vwi.1.B, 2vwi.1.C, 2vwi.1.D, 2vwx.1.A, 2vx3.4.A, 2vz6.1.A, 2w1g.1.A, 2w1i.1.A, 2w1z.1.A, 2w4j.1.A, 2w4k.1.A, 2w4o.1.A, 2w5a.1.A, 2w5h.1.A, 2w7x.1.B, 2w96.1.B, 2w99.1.B, 2w9f.1.B, 2w9z.1.B, 2wb8.1.A, 2wd1.1.A, 2wei.1.A, 2wel.1.A, 2wma.1.A, 2wma.2.A, 2wmb.2.A, 2wnt.1.A, 2wo6.2.A, 2wot.1.A, 2wqb.1.A, 2wqe.1.A, 2wqn.1.A, 2wqo.1.A, 2wtk.1.B, 2wtk.1.C, 2wtk.2.C, 2wtv.1.A, 2wtv.2.A, 2wtw.1.A, 2wu6.1.A, 2wxg.1.A, 2wzj.1.A, 2x0g.1.A, 2x39.1.A, 2x4f.1.A, 2x4z.1.A, 2x6f.1.A, 2x6f.1.B, 2x6j.1.B, 2x7f.1.A, 2x7o.3.A, 2x8e.1.A, 2x8i.1.A, 2x9e.1.A, 2xa4.1.A, 2xba.1.A, 2xck.1.A, 2xik.1.A, 2xir.1.A, 2xj0.1.A, 2xk9.1.A, 2xm8.1.B, 2xne.1.A, 2xng.1.A, 2xnm.1.A, 2xru.1.A, 2xrw.1.A, 2xs0.1.A, 2xuu.1.A, 2xyn.3.A, 2xyu.1.A, 2xzs.1.A, 2y0a.1.A, 2y3a.1.A, 2y4i.1.A, 2y4i.1.B, 2y4p.1.B, 2y4p.2.A, 2y6m.1.A, 2y7j.1.A, 2y7j.1.B, 2y8o.1.A, 2ya9.1.A, 2yak.1.A, 2ycf.1.A, 2ycr.1.A, 2yex.1.A, 2yfx.1.A, 2yhv.1.A, 2yiw.1.A, 2yix.1.A, 2yiy.1.A, 2yjr.1.A, 2yjs.1.A, 2yn8.1.A, 2ywp.1.A, 2yza.1.A, 2z2w.2.A, 2z60.1.A, 2z7l.1.A, 2z7q.1.A, 2z8c.1.A, 2zb1.1.A, 2zdt.1.A, 2zdu.1.A, 2zjw.1.A, 2zm1.1.A, 2zm3.2.A, 2zm3.3.A, 2zmc.1.A, 2zmd.1.A, 2zoq.1.A, 2zoq.2.A, 2zv7.1.A, 3a2c.1.D, 3a2c.1.F, 3a2c.1.G, 3a2c.1.H, 3a2c.1.I, 3a2c.1.L, 3a4o.1.A, 3a4p.1.A, 3a60.1.A, 3a60.1.B, 3a61.1.A, 3a62.1.A, 3a7f.1.A, 3a7g.1.A, 3a7g.2.A, 3a7i.1.A, 3a8w.1.A, 3a8x.1.A, 3a99.1.A, 3ag9.1.A, 3ag9.3.B, 3agl.1.A, 3aln.1.A, 3aln.2.A, 3aln.3.A, 3alo.1.A, 3ama.1.A, 3amy.1.A, 3anr.1.A, 3aox.1.A, 3apc.1.A, 3aqv.1.A, 3at4.1.A, 3ats.1.A, 3axw.1.A, 3b2t.1.A, 3b2w.1.A, 3b8r.1.A, 3b8r.2.A, 3bbt.1.A, 3bce.1.A, 3be2.1.A, 3bea.1.A, 3beg.1.A, 3bgq.1.A, 3bhh.1.A, 3bhh.3.A, 3bht.1.A, 3bht.2.A, 3bhy.1.A, 3bi6.1.A, 3bkb.1.A, 3blq.1.A, 3blr.1.A, 3bpr.4.A, 3bqr.1.B, 3brb.1.A, 3brb.2.A, 3bu3.1.A, 3bv2.1.A, 3bv3.1.A, 3bx5.1.A, 3bym.1.A, 3bys.1.A, 3byv.1.A, 3bz3.1.A, 3c0g.1.A, 3c0i.1.A, 3c1x.1.A, 3c4c.1.A, 3c4c.2.A, 3c4d.1.A, 3c4d.2.A, 3c4e.1.A, 3c4f.1.A, 3c4x.1.A, 3c4y.1.A, 3c4y.1.B, 3c4z.1.A, 3c50.1.B, 3c51.1.A, 3c51.1.B, 3c5i.1.A, 3c5u.1.A, 3c7q.1.A, 3c9w.1.A, 3cbl.1.A, 3cc6.1.A, 3ce3.1.A, 3cik.1.A, 3cjf.1.A, 3cjg.1.A, 3ckw.1.A, 3cly.1.A, 3coh.1.A, 3coi.1.A, 3cok.1.A, 3com.1.A, 3com.2.A, 3cp9.2.A, 3cqu.1.A, 3cs9.1.A, 3cs9.4.A, 3csf.1.A, 3cst.1.A, 3csv.1.A, 3cth.1.A, 3ctj.1.A, 3ctq.1.A, 3cxw.1.A, 3d0e.1.A, 3d14.1.A, 3d2k.1.A, 3d5u.1.A, 3d5v.1.A, 3d5x.1.A, 3d7t.1.A, 3d7t.2.B, 3d7u.1.A, 3d7u.1.B, 3d94.1.A, 3d9v.1.A, 3d9v.1.B, 3da6.1.A, 3dae.1.A, 3daj.1.A, 3dak.1.A, 3dak.1.C, 3dbq.1.A, 3dcv.1.A, 3ddp.1.A, 3ddq.1.A, 3dfa.1.A, 3dfc.1.A, 3dj5.1.A, 3dk3.1.A, 3dk3.2.A, 3dk6.1.A, 3dk6.2.A, 3dk7.1.A, 3dk7.2.A, 3dkc.1.A, 3dkf.1.A, 3dkg.1.A, 3dko.1.A, 3dls.1.A, 3dlz.1.A, 3dnd.1.A, 3dpd.1.A, 3dqw.1.A, 3dqw.3.A, 3dqx.1.A, 3dqx.2.A, 3ds6.1.A, 3ds6.2.A, 3ds6.3.A, 3ds6.4.A, 3dt1.1.A, 3dtc.1.A, 3du8.2.A, 3dv3.1.A, 3dxn.1.A, 3dxp.1.A, 3dxq.1.A, 3dy7.1.A, 3dzo.1.A, 3dzq.1.A, 3e3b.2.B, 3e3p.1.A, 3e5a.1.A, 3e64.1.A, 3e7o.1.A, 3e7o.1.B, 3e8c.2.A, 3e92.1.A, 3e93.1.A, 3eb0.1.A, 3efl.2.A, 3ej1.1.A, 3ekk.1.A, 3el7.1.A, 3el8.1.A, 3emg.1.A, 3en7.1.A, 3en9.1.A, 3en9.2.A, 3ene.1.A, 3enh.1.A, 3enm.1.A, 3enm.1.B, 3enm.2.A, 3eqc.1.A, 3eqp.1.A, 3eqr.1.A, 3erk.1.A, 3eta.1.A, 3eyg.1.A, 3ezv.1.A, 3f2a.1.A, 3f3w.1.A, 3f3z.1.A, 3f5p.17.F, 3f5u.1.A, 3f61.1.A, 3f66.1.A, 3f66.2.A, 3f69.1.A, 3f6x.3.A, 3f7w.1.A, 3f7z.1.A, 3f82.1.A, 3f88.1.A, 3faa.1.A, 3faa.3.A, 3fc1.1.A, 3fe3.1.A, 3fe3.1.B, 3feg.1.A, 3fhi.1.A, 3fhr.1.A, 3fi2.1.A, 3fi3.1.A, 3fi8.1.A, 3fko.1.A, 3fl4.1.A, 3fln.1.A, 3fls.1.A, 3fme.1.A, 3fpm.1.A, 3fsk.1.A, 3fv8.1.A, 3fwq.1.A, 3fwq.2.A, 3fy0.1.A, 3fy2.1.A, 3fzo.1.A, 3fzr.1.A, 3fzt.1.A, 3g0e.1.A, 3g0f.1.A, 3g0f.1.B, 3g2f.1.A, 3g2f.2.A, 3g33.1.A, 3g33.1.C, 3g51.1.A, 3g5b.1.A, 3g6h.1.A, 3g6h.2.A, 3g90.1.A, 3gb2.1.A, 3gbz.1.A, 3gc7.1.A, 3gc8.1.A, 3gc8.2.A, 3gc9.1.A, 3gc9.2.A, 3gcu.1.B, 3geq.1.A, 3gfe.1.A, 3ggf.2.A, 3gi3.1.A, 3gni.1.B, 3gok.1.A, 3gop.1.A, 3gp0.1.A, 3gqi.1.A, 3gql.1.A, 3gql.2.A,

3gql.3.A, 3gt8.1.A, 3gt8.1.B, 3gt8.2.A, 3gt8.2.B, 3gu4.1.A, 3gu8.1.A, 3gub.1.A, 3gvu.2.A, 3h0y.2.A, 3h0z.1.A, 3h0z.2.A, 3h0z.3.A, 3h10.1.A, 3h10.3.A, 3h30.1.A, 3h3c.1.A, 3h4j.1.A, 3h4j.2.A, 3h9o.1.A, 3ha6.1.A, 3ha8.1.A, 3ham.1.A, 3ham.1.B, 3hav.1.A, 3hav.2.A, 3hav.3.A, 3hdn.1.A, 3hec.1.A, 3heg.1.A, 3hgk.1.A, 3hhm.1.A, 3hiz.1.A, 3hko.1.A, 3hll.1.A, 3hmi.1.A, 3hmn.1.A, 3hng.1.A, 3hp2.1.A, 3huc.1.A, 3hv4.1.A, 3hv6.1.A, 3hvc.1.A, 3hx4.1.A, 3hyh.1.A, 3hyh.2.A, 3hzt.1.A, 3i0o.1.A, 3i0q.1.A, 3i1a.1.A, 3i1a.2.A, 3i4b.1.A, 3i4b.1.B, 3i5z.1.A, 3i6u.1.A, 3i6u.1.B, 3i6w.1.A, 3i6w.1.B, 3i6w.2.A, 3i6w.2.B, 3i6w.3.A, 3i6w.3.B, 3i79.1.A, 3i7b.1.A, 3i81.1.A, 3idb.1.A, 3idp.1.A, 3iec.1.A, 3igo.1.A, 3ihy.1.A, 3ihy.3.A, 3ihy.4.A, 3ii5.1.A, 3ik3.1.A, 3ik3.2.A, 3iph.1.A, 3is5.1.A, 3is5.1.F, 3iw4.1.A, 3iw4.2.A, 3iw6.1.A, 3j4r.1.D, 3jr1.1.A, 3js2.2.A, 3juh.1.A, 3jvr.1.A, 3jy0.1.A, 3jy9.1.A, 3jya.1.A, 3k3i.1.A, 3k3j.1.A, 3k54.1.A, 3k5u.1.A, 3ka0.1.A, 3kb7.1.A, 3kc3.1.A, 3kc3.1.B, 3kc3.1.C, 3kc3.2.A, 3kc3.2.C, 3kc3.3.B, 3kc3.3.C, 3kc3.4.A, 3kc3.4.B, 3kc3.4.C, 3kcf.3.A, 3kcf.4.A, 3kcf.5.A, 3kck.1.A, 3kex.1.A, 3kf4.2.A, 3kfa.2.A, 3kk8.1.A, 3kk9.1.A, 3kl8.1.A, 3kmm.1.A, 3kmu.1.A, 3kn5.1.A, 3kn5.2.A, 3kq7.1.A, 3krj.1.A, 3krl.1.A, 3krr.1.A, 3krw.1.A, 3ku2.1.A, 3kul.1.A, 3kvx.1.A, 3kxn.1.A, 3kxx.1.A, 3kxx.3.A, 3kxx.4.A, 3kxz.1.A, 3ky2.1.A, 3ky2.2.A, 3l13.1.A, 3l54.1.A, 3l8p.1.A, 3l9l.1.A, 3l9m.1.A, 3l9p.1.A, 3lcd.1.A, 3lck.1.A, 3lco.1.A, 3lct.1.A, 3lfs.1.A, 3lij.1.A, 3lj1.1.B, 3lj3.1.A, 3llt.1.A, 3lmg.1.A, 3lok.1.A, 3lpb.1.A, 3lq3.1.B, 3lq5.1.A, 3lq8.1.A, 3ls8.1.A, 3lvp.1.A, 3lvp.2.A, 3lvp.3.A, 3lvp.4.A, 3lw0.1.A, 3lxk.1.A, 3lxn.1.A, 3lxp.1.A, 3lzb.1.A, 3m1s.1.B, 3m2w.1.A, 3ma6.1.A, 3ma6.2.A, 3mbl.1.A, 3mdy.1.A, 3mes.1.A, 3mfr.1.A, 3mh0.1.A, 3mh1.1.A, 3mh2.1.A, 3mh3.1.A, 3mi9.1.A, 3mj1.1.A, 3mjw.1.A, 3mn3.1.A, 3mop.1.A, 3mop.1.B, 3mop.1.C, 3mop.1.E, 3mop.1.F, 3mop.1.G, 3mop.1.H, 3mop.1.K, 3mop.1.L, 3mop.1.M, 3mop.1.N, 3mpa.1.A, 3mpm.1.A, 3mpt.1.A, 3ms9.1.A, 3mtl.2.B, 3mv5.1.A, 3mvj.1.A, 3mvj.2.A, 3mvj.3.A, 3mvl.1.A, 3mvl.2.A, 3mvm.1.A, 3mvm.2.A, 3mw1.1.A, 3my0.1.A, 3myg.1.A, 3n4t.1.A, 3n4u.1.A, 3n4v.1.A, 3n9x.1.A, 3nax.1.A, 3nay.1.A, 3ncg.1.A, 3ncz.1.A, 3ncz.1.B, 3ncz.2.A, 3ncz.2.B, 3ndm.1.A, 3new.1.A, 3nga.1.A, 3nie.1.A, 3niz.1.A, 3nlb.1.A, 3nnu.1.A, 3nnx.1.A, 3npc.1.A, 3nr9.1.B, 3nrm.1.A, 3nsz.1.A, 3nun.1.A, 3nus.1.A, 3nw5.1.A, 3nw6.1.A, 3nw7.1.A, 3nx8.1.A, 3nyn.1.A, 3nyx.1.A, 3nz0.1.A, 3nzs.1.A, 3o0g.1.A, 3o0g.2.A, 3o17.1.A, 3o17.2.A, 3o23.1.A, 3o2m.1.A, 3o50.1.A, 3o50.2.A, 3o51.1.A, 3o71.1.A, 3o7l.1.A, 3o7l.1.B, 3o8p.1.A, 3o8t.1.A, 3o8u.1.A, 3o96.1.A, 3oaw.1.A, 3obg.1.A, 3obj.1.A, 3oc1.1.A, 3ocb.1.A, 3ocb.2.A, 3ocg.1.A, 3ocs.1.A, 3oct.1.A, 3od6.1.A, 3ody.1.A, 3odz.1.A, 3oef.1.A, 3oez.1.A, 3oez.2.A, 3of0.1.A, 3ofm.1.A, 3og7.1.A, 3og7.2.A, 3oht.1.A, 3omv.1.A, 3oog.1.A, 3op5.2.A, 3op5.3.A, 3ori.1.A, 3ork.1.A, 3orl.1.A, 3orm.1.A, 3orn.2.A, 3oro.1.A, 3orx.1.A, 3orx.5.A, 3orz.1.A, 3os3.1.A, 3otu.1.A, 3otv.1.A, 3otv.3.A, 3otv.4.A, 3ouk.1.A, 3oun.1.B, 3ovv.1.A, 3owj.1.A, 3oxi.1.A, 3oxz.1.A, 3oy1.1.A, 3oy3.1.A, 3oz6.1.A, 3oz6.1.B, 3p08.3.A, 3p23.1.A, 3p23.1.B, 3p2b.1.A, 3p4k.1.A, 3p7a.1.A, 3p7c.1.A, 3p86.1.A, 3p86.2.A, 3p9j.1.A, 3pa4.1.A, 3pe2.1.A, 3pfq.1.A, 3pg1.1.A, 3pix.1.A, 3piy.1.A, 3piz.1.A, 3pj2.1.A, 3pj8.1.A, 3pjc.1.A, 3pls.1.A, 3pp0.1.A, 3pp0.2.A, 3ppj.1.A, 3ppz.1.A, 3psc.1.A, 3ptg.1.A, 3pvb.1.A, 3pvg.1.A, 3pwy.1.A, 3pxk.1.B, 3pxk.2.A, 3pxq.1.A, 3pxr.1.A, 3py0.1.A, 3py1.1.A, 3py3.1.A, 3pyy.1.A, 3pyy.2.A, 3pze.1.A, 3q04.1.A, 3q2j.1.A, 3q2j.1.B, 3q2m.2.B, 3q32.1.A, 3q32.2.A, 3q3b.1.A, 3q3b.2.A, 3q4c.1.A, 3q4t.1.A, 3q4t.2.A, 3q4u.1.A, 3q4u.1.B, 3q4u.3.A, 3q4z.1.A, 3q4z.1.B, 3q52.1.A, 3q5i.1.A, 3q5z.1.A, 3q60.1.A, 3q6u.1.A, 3q6w.1.A, 3q96.1.A, 3q9w.1.A, 3q9x.2.A, 3q9z.1.A, 3qa0.1.A, 3qa8.1.A, 3qa8.1.B, 3qa8.1.D, 3qa8.2.B, 3qal.1.A, 3qam.1.A, 3qbn.1.A, 3qc4.1.A, 3qc4.2.A, 3qc9.1.A, 3qd2.1.A, 3qd3.1.A, 3qfv.1.A, 3qgw.1.A, 3qgw.2.A, 3qgy.2.A, 3qhr.1.A, 3qjz.1.A, 3qkk.1.A, 3qkl.1.A, 3qqj.1.A, 3qqu.1.A, 3qqu.3.A, 3qrj.1.A, 3qrk.1.A, 3qru.1.A, 3qti.1.A, 3qtr.1.A, 3qtu.1.A, 3qtw.1.A, 3que.1.A, 3qup.1.A, 3qwj.1.A, 3qwk.1.A, 3qxp.1.A, 3qyw.1.A, 3qzg.1.A, 3r21.1.A, 3r22.1.A, 3r2b.1.A, 3r2y.1.A, 3r63.1.A, 3r78.1.A, 3r78.2.A, 3r83.1.A, 3r8m.1.A, 3r8v.1.A, 3r8z.1.A, 3r9d.1.A, 3rai.1.A, 3raw.1.A, 3rcd.1.A, 3rcd.4.A, 3rcj.1.A, 3re4.1.A, 3rgf.1.A, 3rhk.1.A, 3rhx.1.A, 3ri1.1.A, 3rin.1.A, 3rkb.1.A, 3rm6.1.A, 3rni.1.A, 3rny.1.A, 3rp0.1.A, 3rp0.2.A, 3rp9.1.A, 3rps.1.A, 3rps.2.A, 3rtp.1.A, 3rvg.1.A, 3rwp.1.A, 3rwq.1.A, 3rzf.1.A, 3s0o.1.A, 3s3i.1.A, 3s4q.1.A, 3say.1.A, 3sd0.1.A, 3sd0.1.B, 3sdj.1.A, 3sdm.1.A, 3sg8.1.A, 3sg9.1.A, 3sgc.1.A, 3she.1.A, 3sls.1.A, 3soa.1.A, 3soc.1.A, 3sqq.1.A, 3srv.1.A, 3sv0.1.A, 3svv.1.A, 3sxr.1.A, 3sxs.1.A, 3t8m.1.A, 3t8o.1.A, 3t9i.1.A, 3t9t.1.A, 3tac.1.A, 3tdv.1.A, 3tdw.1.A, 3tei.1.A, 3tg1.1.A, 3thb.1.A, 3ti1.1.A, 3tiy.1.A, 3tjd.1.A, 3tjd.2.A, 3tku.1.A, 3tl5.1.A, 3tl8.1.A, 3tl8.2.A, 3tl8.3.A, 3tm0.1.A, 3tni.1.A, 3tnp.2.A, 3tnq.1.A, 3tt0.1.A, 3tt0.2.A, 3ttj.1.A, 3tub.1.A, 3tuc.1.A, 3tud.1.A, 3tv7.1.A, 3tv7.1.B, 3tv7.2.A, 3twj.1.A, 3twj.1.B, 3twj.2.A, 3twj.2.B, 3txo.1.A, 3tyk.1.A, 3tz7.2.A, 3tz9.2.A, 3tzm.1.A, 3u4u.1.A, 3u4w.1.A, 3u51.1.A, 3u6j.1.A, 3u9n.1.A, 3ubd.1.A, 3uc3.1.A, 3uc4.1.A, 3uc4.2.A, 3udb.1.A, 3udb.2.A, 3udb.3.A, 3udb.4.A, 3udb.5.A, 3udb.6.A, 3ue4.1.A, 3ue4.2.A, 3ug1.1.B, 3ugc.1.A, 3uib.1.A, 3uim.1.A, 3uiu.1.A, 3ujg.1.A, 3unj.1.A, 3unk.1.A, 3unz.3.A, 3uo4.1.A, 3uo5.1.A, 3uod.1.A, 3uoj.2.A, 3uok.1.A, 3uol.3.B, 3up2.1.A, 3up7.1.A, 3uqc.1.A, 3uqc.1.B, 3uqc.2.B, 3uqf.1.A, 3uqf.2.A, 3uto.1.A, 3uto.2.A, 3uvp.1.A, 3uvr.1.A, 3uys.2.A, 3uyt.2.A, 3uyt.5.A, 3uyt.5.B, 3uyt.6.A, 3uzp.1.A, 3uzp.2.A, 3uzr.1.A, 3uzs.1.A, 3uzt.1.A, 3v3v.1.A, 3v5j.1.A, 3v5q.1.A, 3v5t.1.A, 3v5w.1.A, 3v6s.1.A, 3v6s.2.A, 3v8s.1.A, 3vap.1.A, 3vby.1.A, 3vhe.1.A, 3vhk.1.A, 3vid.1.A, 3vjo.1.A, 3vn9.1.A, 3vnt.1.A, 3vqu.1.A, 3vry.2.A, 3vs0.2.A, 3vs3.2.A, 3vs6.1.A, 3vs7.1.A, 3vs7.2.A, 3vud.1.A, 3vug.1.A, 3vuh.1.A, 3vui.1.A, 3vuk.1.A, 3vul.1.A, 3vum.1.A, 3vut.1.A, 3vut.2.A, 3vvh.1.A, 3vvh.2.A, 3vw6.1.A, 3vw6.1.B, 3vw8.1.A, 3vwa.1.A, 3w0m.1.A, 3w0n.1.A, 3w0p.1.A, 3w0q.1.A, 3w0r.1.A, 3w0s.1.A, 3w10.1.A, 3w16.1.A, 3w18.1.A, 3w18.2.A, 3w1f.1.A, 3w2c.1.A, 3w2o.1.A, 3w2r.1.A, 3w2s.1.A, 3w32.1.A, 3w55.1.A, 3w8l.1.A, 3w8l.2.A, 3w8q.1.A, 3we8.1.A, 3wf5.1.A, 3wf7.1.A, 3wf8.1.A, 3wi6.1.B, 3wik.1.A, 3wil.1.A, 3wow.1.A, 3wyx.1.A, 3wzd.1.A, 3wze.1.A, 3wzu.1.A, 3x2u.1.A, 3zc6.1.A, 3zc6.2.A, 3zdi.1.A, 3zdu.1.A, 3zep.1.A, 3zep.2.A, 3zep.3.A, 3zew.1.A, 3zfm.1.A, 3zfx.1.A, 3zfy.1.A, 3zh8.1.A, 3zhp.1.B, 3zim.1.A, 3zls.1.A, 3zlw.1.A, 3zly.1.A, 3zmm.1.A, 3zon.1.A, 3zos.2.A, 3zrk.1.A, 3zsh.1.A, 3zsi.1.A, 3ztx.1.A, 3ztx.2.A, 3zu7.1.A, 3zut.1.A, 3zuu.1.A, 3zuv.1.A, 3zw3.1.A, 3zxt.1.A, 3zxt.1.B, 3zxt.2.A, 3zya.1.A, 3zzw.1.A, 3zzw.1.B, 4a4x.1.A, 4a55.1.A, 4a7c.1.A, 4aaa.1.A, 4acc.1.A, 4acd.1.B, 4ach.1.A, 4ae6.1.A, 4ae6.2.A, 4af3.1.A, 4afe.1.A, 4afj.1.A, 4agc.1.A, 4agu.1.A, 4agw.1.A, 4ajw.1.A, 4alu.1.A, 4alv.1.A, 4an2.1.A, 4anl.1.A, 4anm.1.A, 4ann.1.A, 4ano.1.A, 4ans.1.A, 4anu.1.A, 4anv.1.A, 4anx.1.A, 4aof.1.A, 4aoj.1.A, 4aot.1.A, 4aot.2.A, 4ap7.1.A, 4apc.1.A, 4app.1.A, 4aqc.1.A, 4aqc.2.A, 4ark.1.B, 4asd.1.A, 4asz.1.A, 4at3.1.A, 4at4.1.A, 4at5.1.A, 4au8.1.A, 4aw2.1.A, 4aw5.1.A, 4awi.1.A, 4ax8.1.A, 4azf.1.A, 4azt.1.A, 4azw.1.A, 4b0g.1.A, 4b4l.1.A, 4b6l.1.A, 4b7t.1.A, 4b8l.1.A, 4b8m.1.A, 4b8m.2.A, 4b99.1.A, 4bb4.1.A, 4bbe.1.A, 4bbf.3.A, 4bbm.1.A, 4bc6.1.A, 4bcf.1.A, 4bcg.1.A, 4bci.1.A, 4bcj.1.A, 4bck.1.A, 4bck.2.A, 4bcm.1.A, 4bcn.1.A, 4bco.1.A, 4bcq.1.A, 4bcq.2.A, 4bfm.1.A, 4bfr.1.A, 4bfr.2.A, 4bgq.1.A, 4bib.1.A, 4bib.2.A, 4bkj.2.A, 4bn1.1.A, 4br3.1.B, 4brx.1.A, 4btf.1.A, 4btj.1.A, 4btj.2.A, 4btk.1.A, 4bvu.1.A, 4bwk.1.A, 4bwk.1.B, 4bwp.1.A, 4bwp.1.B, 4bwx.1.A, 4bwx.1.B, 4byi.1.A, 4byj.1.A, 4bzo.1.A, 4c02.1.A, 4c0t.1.A, 4c2v.1.A, 4c2v.2.A, 4c2w.1.A, 4c2w.2.A, 4c33.1.A, 4c34.1.A, 4c35.1.A, 4c3p.1.A, 4c3p.1.C, 4c3r.1.A, 4c57.2.A, 4c8b.1.A, 4cfe.1.A, 4cfh.1.A, 4cfn.1.A, 4cfu.1.A, 4cfu.2.A, 4cfw.1.A, 4cfx.1.A, 4cfx.2.A, 4cki.1.A, 4ckr.1.A, 4cnh.2.A, 4cqe.1.A, 4cqe.2.A, 4cqg.1.A, 4crs.1.A, 4csv.1.A, 4ct1.1.A, 4cxa.1.A, 4cxa.2.A, 4cyi.1.A, 4cyi.1.B, 4cyj.1.A, 4cyj.1.B, 4czt.1.A, 4czt.1.B, 4czt.2.A, 4czt.2.B, 4czu.1.A, 4czu.1.B, 4czu.2.A, 4czu.2.B, 4czy.1.B, 4czy.1.D, 4d0l.1.A, 4d0l.3.A, 4d1s.1.A, 4d28.1.A, 4d28.1.B, 4d28.2.A, 4d28.2.B, 4d2p.1.A, 4d2p.1.B, 4d2p.2.A, 4d2p.2.B, 4d2r.1.A, 4d2s.1.A, 4d2t.2.A,

4d2t.4.A, 4d2w.1.B, 4d4r.1.A, 4d4r.2.A, 4d4v.1.A, 4d4v.2.A, 4d55.1.A, 4d9t.1.A, 4da5.1.A, 4dbn.1.A, 4dbn.1.B, 4dbx.1.A, 4dc2.1.A, 4dca.1.A, 4dce.1.A, 4de4.2.A, 4deb.1.A, 4dee.1.A, 4deg.1.A, 4dfb.1.A, 4dfb.2.A, 4dfl.1.A, 4dfu.1.A, 4dfu.2.A, 4dfy.1.A, 4dfz.1.A, 4dg0.1.A, 4dg2.1.A, 4dg3.1.A, 4dgg.1.A, 4dgl.1.C, 4dgl.1.D, 4dgm.1.A, 4dgn.1.A, 4dh7.1.A, 4dhf.1.A, 4din.1.A, 4dit.1.A, 4dk5.1.A, 4dn5.3.A, 4dt9.1.A, 4dta.1.A, 4dtb.1.A, 4dtk.1.A, 4dym.1.A, 4e1z.1.A, 4e20.1.A, 4e26.1.B, 4e3c.1.A, 4e3c.1.B, 4e3c.2.A, 4e3c.2.B, 4e3c.3.A, 4e3c.3.B, 4e4l.1.A, 4e4m.1.A, 4e4x.1.A, 4e5a.1.A, 4e5b.1.A, 4e6d.1.A, 4e6d.2.A, 4e6q.1.A, 4e73.1.A, 4e7w.1.A, 4e93.1.A, 4ebw.1.A, 4ec9.1.A, 4eev.1.A, 4eh2.1.A, 4eh5.1.A, 4eh9.1.A, 4ehg.1.B, 4ehv.1.A, 4ejn.1.A, 4eoi.1.A, 4eoi.2.A, 4eoj.1.A, 4eoj.2.A, 4eok.1.A, 4eok.2.A, 4eom.1.A, 4eom.2.A, 4eon.1.A, 4eon.2.A, 4eoo.1.A, 4eoo.2.A, 4eop.1.A, 4eop.2.A, 4eoq.1.A, 4eoq.2.A, 4eor.1.A, 4eor.2.A, 4eos.1.A, 4eos.2.A, 4eqc.1.A, 4eqm.1.A, 4eqm.4.A, 4equ.1.A, 4erk.1.A, 4erw.1.A, 4eut.1.A, 4eut.1.B, 4euu.1.A, 4ewh.1.A, 4ewq.1.A, 4ey0.1.A, 4eyj.1.A, 4eym.1.A, 4ez3.1.A, 4ez5.1.A, 4ez7.1.A, 4ezk.1.A, 4ezl.1.A, 4f08.2.A, 4f09.1.A, 4f0f.1.A, 4f0g.1.A, 4f0i.1.A, 4f0i.2.A, 4f1m.1.A, 4f1o.1.A, 4f1s.1.A, 4f1t.1.A, 4f42.1.A, 4f4p.1.A, 4f65.1.A, 4f6s.1.A, 4f6u.1.A, 4f6w.1.A, 4f7l.1.A, 4f9a.1.A, 4fa6.1.A, 4fbn.1.A, 4fbx.1.A, 4feu.2.A, 4feu.3.A, 4fev.1.A, 4fev.2.A, 4fev.3.A, 4fev.4.A, 4fev.6.A, 4fex.3.A, 4fex.4.A, 4fex.5.A, 4ff8.1.A, 4fg7.1.A, 4fg8.1.A, 4fg9.1.A, 4fgb.1.A, 4fie.1.A, 4fif.2.A, 4fjy.1.A, 4fk3.1.A, 4fk3.1.B, 4fl1.1.A, 4fl2.1.A, 4fl3.1.A, 4fmq.1.A, 4fnw.1.A, 4fnx.1.A, 4fny.1.A, 4fnz.1.A, 4fob.1.A, 4fr4.1.A, 4fr4.4.A, 4fsn.1.A, 4fst.1.A, 4fsu.1.A, 4ft3.1.A, 4ful.1.A, 4fv3.1.A, 4fv7.1.A, 4fv8.1.A, 4fx3.2.A, 4fza.1.B, 4fzd.1.B, 4fzf.1.B, 4g1w.1.A, 4g31.1.A, 4g3c.1.A, 4g3c.2.A, 4g3d.1.A, 4g3d.2.A, 4g3f.1.A, 4g3g.1.A, 4g5j.1.A, 4g5p.1.A, 4g5p.2.A, 4g9c.1.A, 4g9c.2.A, 4g9r.1.A, 4gb9.1.A, 4gcj.1.A, 4geo.1.A, 4gfm.1.A, 4gfo.1.A, 4gg5.1.A, 4gg7.1.A, 4gii.1.A, 4gl9.1.A, 4gmy.1.A, 4gs6.1.A, 4gt3.1.A, 4gt4.1.A, 4gt5.1.A, 4gu6.1.B, 4gu6.2.A, 4gub.1.A, 4gv1.1.A, 4gvj.1.A, 4gyg.1.A, 4h05.1.A, 4h05.2.A, 4h1j.1.A, 4h3b.1.A, 4h3p.1.A, 4h3q.1.A, 4h58.1.A, 4h58.2.A, 4h58.3.A, 4hct.1.A, 4hgs.1.A, 4hgt.2.A, 4hjo.1.A, 4hle.1.A, 4hnf.1.A, 4hnf.2.A, 4hni.1.A, 4hok.1.A, 4hok.11.A, 4hok.12.A, 4hok.6.A, 4hok.9.A, 4hvb.1.A, 4hvg.1.A, 4hvs.2.B, 4hw7.1.A, 4hyh.1.A, 4hyi.1.A, 4hzr.1.A, 4hzs.1.A, 4i1z.1.A, 4i20.1.A, 4i21.1.A, 4i21.2.A, 4i22.1.A, 4i23.1.A, 4i24.1.A, 4i3z.1.A, 4i41.1.A, 4i4f.1.A, 4i5h.1.A, 4i5m.1.A, 4i6h.1.A, 4i92.1.A, 4i93.1.A, 4i93.2.A, 4i94.1.A, 4i94.2.A, 4iaa.1.A, 4ian.1.B, 4iay.1.A, 4ib5.1.A, 4ib5.2.A, 4ib5.3.A, 4ibm.1.A, 4ibm.2.A, 4ic7.1.A, 4ic7.2.A, 4ic8.1.A, 4id7.1.A, 4idt.1.A, 4idt.1.B, 4idv.1.A, 4ieb.1.A, 4ifc.1.A, 4ifc.2.A, 4ifg.1.A, 4ii5.1.A, 4ijp.1.A, 4im0.1.A, 4im3.1.A, 4imy.1.A, 4imy.3.A, 4iq6.1.A, 4ith.1.A, 4ith.2.A, 4iti.1.A, 4iti.2.A, 4itj.1.A, 4itj.2.A, 4iva.1.A, 4iw0.1.A, 4iwd.1.A, 4iwo.1.A, 4iwp.1.A, 4ix3.1.A, 4ix3.1.B, 4ixp.1.A, 4iz5.1.A, 4iz7.1.A, 4iz7.2.A, 4iza.1.A, 4j1r.2.A, 4j52.1.A, 4j6i.1.A, 4j7b.1.A, 4j8m.1.A, 4j8n.3.A, 4j95.5.A, 4j96.1.A, 4j96.2.A, 4j97.1.A, 4j98.1.A, 4j98.2.A, 4j99.1.A, 4jai.1.A, 4jaj.1.A, 4jbo.1.A, 4jbq.1.A, 4jdj.1.A, 4jg6.1.A, 4jg7.1.A, 4jg8.1.A, 4ji9.1.A, 4ji9.2.A, 4jia.1.A, 4jjr.1.A, 4jjr.2.A, 4jl9.1.A, 4jlc.1.B, 4jnw.1.A, 4jq7.1.A, 4jr7.1.A, 4jrn.1.A, 4js8.1.A, 4jt3.1.A, 4jvg.1.A, 4jvg.2.A, 4jx3.1.A, 4jxf.1.A, 4k0y.1.A, 4k11.1.A, 4k18.1.A, 4k2r.1.A, 4k33.1.A, 4k6z.1.A, 4k9y.1.A, 4kab.1.A, 4kao.1.A, 4kao.2.A, 4kb8.1.A, 4kbc.3.A, 4kbc.3.B, 4kik.1.A, 4kik.1.B, 4kin.1.A, 4kin.4.A, 4kio.2.A, 4kip.1.A, 4kiq.1.A, 4kiq.4.A, 4knb.1.A, 4knb.4.A, 4krc.1.A, 4ks7.1.A, 4ks8.1.A, 4ksp.1.B, 4kwp.1.A, 4l00.1.A, 4l00.2.A, 4l01.1.A, 4l01.2.A, 4l23.1.A, 4l3j.1.A, 4l3p.1.A, 4l42.1.A, 4l43.1.A, 4l44.1.A, 4l45.1.A, 4l46.1.A, 4l52.1.A, 4l67.1.A, 4l68.1.A, 4l68.2.A, 4l6q.1.A, 4l7f.1.A, 4l8m.1.A, 4l9i.1.A, 4lfi.1.B, 4lg4.1.A, 4lg4.2.A, 4lg4.3.A, 4lg4.4.A, 4lg4.5.A, 4lg4.6.A, 4lgd.1.A, 4lgd.2.A, 4lgd.3.A, 4lgd.4.A, 4lgg.1.A, 4lgh.1.A, 4lgh.2.A, 4li5.1.A, 4ll0.1.A, 4loo.1.A, 4lop.1.A, 4lop.3.A, 4loq.4.A, 4lqm.1.A, 4lqp.1.A, 4lqq.1.A, 4lqq.2.A, 4lqs.1.A, 4lrj.1.A, 4lrk.1.A, 4lrk.2.A, 4lrk.3.A, 4lrm.1.A, 4lrm.2.A, 4lrm.3.A, 4lrm.4.A, 4lrm.5.A, 4lv5.2.A, 4lv8.1.A, 4m0y.1.A, 4m66.1.A, 4m67.1.A, 4m68.1.A, 4m69.1.A, 4m69.1.B, 4m8t.1.A, 4m97.1.A, 4mao.1.A, 4mbi.1.A, 4mbj.1.A, 4mcv.1.A, 4md7.1.C, 4md8.1.C, 4md9.1.C, 4mf0.1.B, 4mf1.1.B, 4mh7.2.A, 4mk0.1.A, 4mne.1.B, 4mne.1.C, 4mne.2.A, 4mne.2.D, 4mnf.1.A, 4mnf.1.B, 4mta.1.A, 4mvf.1.A, 4mwi.1.A, 4mxa.1.A, 4mxc.1.A, 4mxo.1.A, 4mxx.1.A, 4mxx.2.A, 4mxy.1.A, 4mxy.2.A, 4myg.1.A, 4myg.2.A, 4n4s.1.A, 4n57.2.A, 4nct.3.A, 4nct.4.A, 4neu.1.A, 4neu.2.A, 4nfm.1.A, 4nfn.1.A, 4nh1.1.A, 4nif.1.A, 4nif.1.D, 4nj3.1.A, 4njd.1.A, 4nm0.1.A, 4nm3.1.A, 4nm5.1.A, 4nst.3.A, 4nt4.1.A, 4nts.1.A, 4nts.2.A, 4ntt.1.A, 4ntt.2.A, 4nus.1.A, 4nw6.1.A, 4nwm.1.A, 4nzw.1.B, 4o0r.1.A, 4o0r.2.A, 4o0s.1.A, 4o0t.1.A, 4o0t.2.A, 4o0u.1.A, 4o0v.1.A, 4o0w.1.A, 4o1o.2.A, 4o21.1.A, 4o27.1.B, 4o2p.1.A, 4o2z.1.A, 4o38.1.A, 4o6e.1.A, 4o6l.2.A, 4o7o.1.A, 4o7o.1.B, 4o7p.1.B, 4o91.2.C, 4o96.1.A, 4o96.2.A, 4oau.1.A, 4oav.1.A, 4oav.1.B, 4obq.1.A, 4obq.1.B, 4ocj.1.A, 4ocu.1.A, 4ogr.1.A, 4oh4.1.A, 4oh4.2.A, 4oli.1.A, 4or5.1.A, 4or5.2.A, 4ork.1.A, 4ork.3.A, 4ork.4.A, 4otd.1.A, 4otf.1.A, 4oth.1.A, 4otp.1.A, 4ovv.1.A, 4ow8.1.A, 4p2k.1.A, 4p2w.3.B, 4p4c.1.A, 4p90.1.A, 4p90.2.A, 4pdo.1.B, 4pdp.1.A, 4pdy.1.A, 4ped.1.A, 4pf4.1.A, 4ph4.1.A, 4pl3.1.A, 4pl3.1.B, 4pl4.1.A, 4pl4.1.B, 4pl4.2.A, 4pl4.2.B, 4pl5.1.A, 4pl5.1.B, 4pl5.2.A, 4pl5.2.B, 4pmm.1.A, 4pmt.1.A, 4pni.1.A, 4pnk.1.A, 4pp7.1.A, 4pp9.1.A, 4ppa.3.A, 4ppb.3.B, 4ppc.3.A, 4ppc.3.B, 4pqn.1.A, 4prj.1.A, 4ps8.1.A, 4pte.1.A, 4pv0.1.A, 4pwn.1.A, 4px6.1.A, 4py1.1.A, 4q2a.1.A, 4q5e.1.A, 4q5j.1.A, 4q5j.2.A, 4q9z.1.A, 4q9z.1.B, 4qd6.1.A, 4qd6.2.A, 4qfg.1.A, 4qml.1.A, 4qmm.1.A, 4qmn.1.A, 4qmt.1.A, 4qny.1.A, 4qo9.1.A, 4qo9.2.A, 4qox.1.A, 4qp1.1.A, 4qp1.2.A, 4qp2.2.A, 4qp3.1.A, 4qp3.2.A, 4qp4.1.A, 4qp4.2.A, 4qpa.1.A, 4qpm.1.A, 4qps.1.A, 4qps.2.A, 4qq5.1.A, 4qqc.1.A, 4qqj.1.A, 4qqt.1.A, 4qta.1.A, 4qtd.1.A, 4qte.2.B, 4qye.1.A, 4qyy.1.A, 4r1v.1.A, 4r1y.1.A, 4r3c.1.A, 4r3p.1.A, 4r3r.1.A, 4r5s.1.A, 4r5y.1.A, 4r77.1.A, 4r78.1.A, 4r7b.1.A, 4r8q.1.A, 4ra4.1.A, 4red.1.A, 4red.1.B, 4rer.1.A, 4rew.1.A, 4rfm.1.A, 4rgj.1.A, 4rio.1.A, 4riw.1.A, 4riw.1.B, 4rix.1.A, 4rix.1.B, 4rix.2.B, 4riy.1.A, 4riy.1.B, 4riy.2.B, 4rlo.1.A, 4rlo.2.A, 4rlp.1.A, 4rmz.1.A, 4rpv.1.A, 4rss.1.A, 4rt7.1.A, 4rvt.1.A, 4rvt.1.B, 4rwi.1.A, 4rwi.2.A, 4rwl.2.A, 4rx5.1.A, 4rx9.1.A, 4rz7.1.B, 4rzv.1.A, 4rzv.2.A, 4rzw.1.A, 4rzw.1.B, 4s2z.1.A, 4s30.1.A, 4s31.1.A, 4s33.1.A, 4tks.1.A, 4tl0.1.A, 4tn6.1.A, 4tn6.2.A, 4tnb.1.A, 4tpt.1.A, 4tt7.1.A, 4tth.1.B, 4tuu.1.A, 4tw9.1.A, 4tw9.1.B, 4twn.1.A, 4twp.1.A, 4twp.2.A, 4txc.1.A, 4tye.1.A, 4tyg.1.A, 4tyh.1.A, 4tyh.1.B, 4tyi.1.A, 4tyj.1.A, 4u0i.1.A, 4u3y.1.A, 4u3y.1.B, 4u3z.1.A, 4u3z.1.B, 4u40.2.A, 4u41.2.A, 4u42.1.A, 4u42.1.B, 4u43.1.A, 4u43.3.A, 4u44.2.A, 4u45.2.A, 4u45.3.A, 4u5j.2.A, 4u6r.1.A, 4u79.1.A, 4u7z.1.A, 4u94.1.A, 4u9a.1.A, 4u9a.1.B, 4uak.1.A, 4ub7.1.A, 4uba.1.A, 4uba.2.A, 4ueu.1.A, 4ump.1.A, 4ump.1.B, 4umq.1.A, 4umt.1.A, 4un0.1.B, 4urk.1.A, 4usd.1.A, 4use.2.A, 4usf.1.A, 4usf.2.A, 4uv0.1.A, 4uw0.1.A, 4uwg.1.A, 4uwy.1.A, 4uwy.2.A, 4ux9.1.A, 4ux9.4.A, 4uxl.1.A, 4uxq.1.A, 4uy9.1.A, 4uya.1.A, 4uyn.1.A, 4uzd.1.A, 4uzh.1.A, 4v01.2.A, 4v0g.1.A, 4v0g.2.A, 4v0i.2.A, 4w4y.1.A, 4w7p.1.A, 4w8d.1.A, 4w8e.1.A, 4w9w.1.A, 4wa9.1.A, 4wa9.2.A, 4wae.1.A, 4waf.1.A, 4wb5.1.A, 4wb6.1.A, 4wb7.1.A, 4wb7.2.A, 4wb8.1.A, 4wbb.1.B, 4wbo.1.A, 4wh1.1.A, 4whz.1.A, 4wih.1.A, 4wnk.1.A, 4wnm.1.A, 4wnp.1.A, 4wnp.2.A, 4wnp.4.A, 4wo5.1.A, 4wo5.2.A, 4wot.1.A, 4wov.1.A, 4wsq.1.A, 4wsq.2.A, 4wsy.1.A, 4wua.1.A, 4wun.1.A, 4ww5.1.A, 4wwn.1.A, 4x0m.1.A, 4x3f.1.A, 4x3j.1.A, 4x6r.1.A, 4x7l.1.A, 4x7q.1.A, 4x7q.2.A, 4xbr.1.A, 4xbu.1.A, 4xcu.1.A, 4xe0.1.A, 4xey.1.A, 4xey.2.A, 4xg3.2.A, 4xh0.1.A, 4xh6.1.A, 4xhk.1.A, 4xhl.1.A, 4xi2.1.A, 4xj0.1.A, 4xj0.2.A, 4xli.1.A, 4xlv.1.A, 4xne.1.A, 4xoy.1.A, 4xr7.1.B, 4xr7.1.C, 4xr7.2.A, 4xr7.2.B, 4xr7.4.A, 4xr7.4.B, 4xrl.1.A, 4xuf.1.A, 4xuf.2.A, 4xv1.1.A, 4xv1.1.B, 4xv9.1.A, 4xx9.1.A, 4y0x.1.A, 4y5h.1.A, 4y5q.1.A, 4y73.1.A, 4y73.2.A, 4y73.4.A, 4y83.3.A, 4y85.2.A, 4y8d.2.A, 4y93.1.A, 4y95.1.A, 4y95.2.A, 4ybk.1.A, 4yc3.1.A, 4yc6.1.A, 4yc8.1.A, 4yff.1.A, 4yfi.1.A, 4yfi.1.B, 4yga.1.A, 4yga.4.A, 4yhf.1.A, 4yhf.2.A, 4yhj.1.A, 4yhj.1.B, 4yht.1.A, 4yjo.1.A, 4yjq.1.A, 4yjt.1.A, 4yju.1.A, 4ymj.1.A, 4yne.1.A, 4ynz.1.A, 4ynz.1.B, 4yom.1.A,

4yp8.4.A, 4yr8.1.A, 4yr8.3.A, 4ysj.1.A, 4ysm.2.A, 4ytc.1.A, 4yur.1.A, 4yz9.1.A, 4yz9.2.A, 4yz9.3.A, 4yzb.2.A, 4yzc.1.A, 4yzd.1.A, 4yzd.3.A, 4yzm.1.A, 4yzm.2.A, 4yzn.1.A, 4z16.1.A, 4z3v.1.A, 4z55.1.A, 4z7g.1.A, 4z7h.2.A, 4z83.1.A, 4z84.1.A, 4zeg.1.A, 4zhx.1.A, 4zhx.2.A, 4zji.1.A, 4zji.3.A, 4zjv.2.A, 4zlo.1.A, 4zlo.1.B, 4zly.1.A, 4zog.1.A, 4zog.2.A, 4zop.1.B, 4zp5.1.A, 4zp5.2.A, 4zs0.1.A, 4zsa.1.A, 4zse.2.A, 4zsg.1.A, 4zsj.1.A, 4zsl.1.A, 4zxt.1.A, 4zy4.1.A, 4zy4.2.A, 4zy6.1.A, 4zy6.2.A, 4zzm.1.A, 5a14.1.A, 5a46.1.A, 5a4c.1.A, 5a4e.3.A, 5a6n.1.A, 5a6n.1.B, 5a9u.1.A, 5aa8.1.A, 5aa9.1.A, 5aad.1.A, 5aae.1.A, 5acb.1.B, 5acb.2.B, 5aep.1.A, 5afv.1.B, 5aik.2.A, 5air.1.A, 5air.1.B, 5ajq.1.A, 5ajq.2.A, 5am6.2.A, 5am7.1.A, 5am7.2.A, 5amn.1.A, 5ane.1.A, 5ap0.1.A, 5ap1.1.A, 5ap2.1.A, 5ap3.1.A, 5ap6.1.A, 5ap7.1.A, 5ar2.1.A, 5ar4.1.B, 5ar7.1.A, 5ar7.1.B, 5ar8.1.A, 5av4.1.A, 5awm.1.A, 5ax3.1.A, 5ax9.1.A, 5ax9.2.A, 5ax9.3.A, 5b0x.1.A, 5b2k.1.A, 5b2l.1.A, 5b2m.1.A, 5b7v.2.A, 5bmm.1.A, 5bms.1.A, 5bnj.1.A, 5bpy.1.A, 5bpy.2.A, 5bq0.1.A, 5bue.1.A, 5bvf.1.A, 5bvk.1.A, 5bvo.1.A, 5byl.3.A, 5byl.4.A, 5byy.1.A, 5byz.1.A, 5c01.1.A, 5c03.2.A, 5c46.1.A, 5c4k.2.A, 5c4l.1.A, 5c4l.2.A, 5cas.1.A, 5ce3.1.B, 5cei.1.A, 5cek.1.A, 5cem.1.A, 5cen.1.A, 5ceo.1.A, 5cf5.1.A, 5cf5.2.A, 5ci6.1.A, 5ci6.2.A, 5ckw.1.A, 5ckw.1.B, 5clr.1.A, 5clr.1.B, 5cnn.1.A, 5cno.2.A, 5cqu.1.A, 5cs6.1.A, 5csh.2.A, 5csp.1.A, 5csw.1.A, 5csx.1.A, 5ct7.1.A, 5cu6.1.A, 5cvf.1.A, 5cvg.1.A, 5cwz.1.A, 5cwz.2.A, 5cwz.3.A, 5cxh.1.A, 5cxz.1.A, 5cy3.1.A, 5cyi.1.A, 5cyz.1.A, 5czh.1.A, 5czo.1.A, 5d10.1.A, 5d10.2.A, 5d11.1.A, 5d11.2.A, 5d12.1.A, 5d12.2.A, 5d1j.1.A, 5d41.1.A, 5d41.1.B, 5d7a.1.A, 5d7a.3.A, 5d7v.1.A, 5d9h.1.A, 5d9k.1.A, 5d9l.1.A, 5da3.1.A, 5dbx.1.A, 5dbx.1.B, 5de2.1.A, 5de2.2.A, 5dew.2.A, 5dey.1.A, 5dey.2.A, 5dfp.1.A, 5dfz.1.B, 5dfz.1.C, 5dg5.1.B, 5dgz.1.A, 5dh3.1.A, 5di1.1.A, 5di1.2.A, 5dls.1.A, 5dmz.1.A, 5dmz.2.A, 5dnr.1.A, 5dos.1.A, 5drb.1.A, 5dt0.1.A, 5dt4.1.A, 5dvr.1.A, 5dxh.1.A, 5dxt.1.A, 5dxu.1.A, 5dyk.2.A, 5e1e.2.A, 5e1s.1.A, 5e7r.1.A, 5e8u.1.A, 5e8x.1.A, 5eak.1.A, 5ebz.1.A, 5ebz.1.F, 5ebz.2.E, 5ebz.2.F, 5edp.1.A, 5edr.1.A, 5efq.1.A, 5eg3.1.A, 5ei6.1.A, 5ek7.1.A, 5eob.1.A, 5eqe.1.A, 5es1.1.A, 5eta.1.A, 5eta.2.A, 5etc.1.A, 5etf.1.A, 5eti.1.A, 5ew3.1.A, 5ew3.2.A, 5ew9.1.A, 5eyk.1.A, 5eym.1.A, 5ezr.1.A, 5ezv.1.A, 5ezv.2.A, 5f1z.1.A, 5f4n.1.A, 5f94.1.A, 5f9e.1.A, 5f9e.2.A, 5fbo.1.A, 5fbw.1.A, 5fd2.1.A, 5fd2.1.B, 5fdp.1.A, 5fdx.1.A, 5fee.1.A, 5fg8.1.A, 5fgk.1.A, 5fi4.1.A, 5fi4.1.B, 5flf.1.A, 5flf.2.A, 5flf.3.A, 5flf.5.A, 5fm2.1.A, 5fqd.1.C, 5fri.1.A, 5ftg.1.B, 5fwl.1.D, 5fxq.1.A, 5fxr.1.A, 5fxs.1.A, 5g15.1.A, 5g1x.1.A, 5g2n.1.A, 5g55.1.A, 5g6v.1.A, 5g6v.2.A, 5ghv.1.A, 5gjd.1.A, 5gjg.1.A, 5gmp.1.A, 5gnk.1.A, 5grn.1.A, 5gty.1.B, 5gty.1.C, 5gty.1.D, 5gz8.1.A, 5gza.1.A, 5h09.1.A, 5h0e.1.A, 5h2u.1.A, 5h3q.1.A, 5h8g.1.A, 5hbe.1.A, 5hbh.1.A, 5hd7.1.A, 5he1.1.A, 5he2.1.A, 5he3.1.A, 5hes.1.A, 5hez.1.A, 5hez.2.A, 5hg9.1.A, 5hgi.1.A, 5hhw.1.A, 5hi2.1.A, 5hie.1.A, 5hie.3.A, 5hie.4.A, 5hln.1.A, 5hln.1.B, 5hlp.2.A, 5hlw.1.A, 5hnb.1.A, 5hng.1.A, 5hni.1.A, 5hnv.1.A, 5hoa.1.A, 5hor.1.A, 5hti.1.A, 5hu3.1.A, 5hu9.1.A, 5hvj.1.A, 5hvj.2.A, 5hvk.1.A, 5hvk.2.A, 5hvy.1.A, 5hx6.1.A, 5hx6.2.A, 5hze.1.A, 5hzn.7.A, 5hzn.8.A, 5i35.1.A, 5i3o.1.A, 5i3o.2.A, 5i4n.1.A, 5i4u.2.A, 5i5z.1.A, 5i6u.1.A, 5i8a.1.A, 5i9v.1.A, 5i9w.1.A, 5i9y.1.A, 5ia0.2.A, 5ia0.3.A, 5ia1.1.A, 5ia4.1.A, 5idn.1.A, 5idp.1.A, 5if1.1.A, 5if1.2.A, 5ig1.1.A, 5ig1.2.A, 5igh.1.A, 5igr.1.A, 5igv.1.A, 5igy.1.A, 5igz.1.A, 5ih0.1.A, 5ih1.1.A, 5ih4.1.A, 5iha.1.A, 5ikw.1.A, 5ime.1.A, 5ime.2.A, 5imx.1.A, 5iqc.1.A, 5iqh.1.A, 5iqh.4.A, 5iqi.1.A, 5iqi.4.A, 5iso.1.A, 5iso.2.A, 5ita.1.A, 5ita.2.A, 5itd.1.B, 5iu2.2.A, 5iuh.1.A, 5iwu.1.A, 5izj.1.A, 5izj.2.A, 5j0a.1.A, 5j0a.1.B, 5j1v.2.A, 5j1v.3.A, 5j5t.1.A, 5j79.1.A, 5j7s.1.A, 5j87.1.A, 5j87.1.D, 5j8i.1.A, 5j95.1.A, 5j95.2.A, 5j9l.1.A, 5j9y.1.A, 5j9z.1.A, 5jeb.1.A, 5jfs.1.A, 5jga.1.A, 5jh6.1.A, 5jha.1.A, 5jhb.1.A, 5jk3.1.A, 5jkg.1.A, 5jn2.1.A, 5jr7.1.A, 5jr7.2.A, 5jrq.1.A, 5jsm.2.A, 5jsm.2.B, 5jy7.1.E, 5jy7.1.F, 5jy7.1.H, 5jzj.1.A, 5jzj.2.A, 5jzn.1.A, 5k00.1.A, 5k0k.1.A, 5k0x.1.A, 5k0x.2.A, 5k3y.1.A, 5k3y.2.A, 5k4i.1.A, 5k4j.1.A, 5k5n.1.A, 5k5x.1.A, 5k72.1.A, 5k75.3.A, 5k7i.1.A, 5k9i.2.A, 5kbq.1.A, 5kbq.2.A, 5kbr.1.A, 5kc2.1.A, 5kc2.1.B, 5kcv.1.A, 5kcx.1.A, 5ke0.1.A, 5khw.2.A, 5khx.1.A, 5kkr.1.A, 5kkr.1.B, 5kmi.1.A, 5kmk.1.A, 5kml.1.A, 5knj.1.A, 5ko1.1.A, 5kpk.2.A, 5kpm.1.A, 5ku8.1.A, 5kvt.1.A, 5kx7.2.A, 5kx8.1.A, 5kx8.2.A, 5kx8.3.A, 5kx8.4.A, 5kz8.1.A, 5kz8.2.A, 5l1z.2.A, 5l2i.1.A, 5l2q.1.A, 5l2q.3.A, 5l2q.4.A, 5l2s.1.A, 5l2t.1.A, 5l2w.1.A, 5l3a.1.A, 5l4q.1.A, 5l6w.1.A, 5l8k.1.A, 5l8l.1.A, 5lar.1.A, 5lcj.1.A, 5lck.1.A, 5lcq.1.A, 5lcr.1.A, 5li1.1.A, 5li9.1.A, 5lih.1.A, 5ljj.1.A, 5lma.1.A, 5lmk.2.A, 5loh.1.A, 5lpw.1.A, 5lpz.1.A, 5lqf.1.A, 5lvp.1.A, 5lw1.2.B, 5lwm.1.A, 5lxc.1.A, 5lxd.1.A, 5lxm.1.A, 5m06.1.A, 5m07.1.A, 5m08.1.A, 5m08.2.A, 5m09.1.A, 5m09.2.A, 5m0l.1.A, 5m44.1.A, 5m4c.1.A, 5m4f.1.A, 5m4i.1.A, 5m4u.1.A, 5m51.1.A, 5m53.1.A, 5m56.1.A, 5m56.2.A, 5m57.1.A, 5m6u.1.A, 5mag.1.A, 5mah.1.A, 5mai.1.A, 5mhq.1.A, 5mja.1.A, 5mjb.1.A, 5ml5.1.A, 5mo4.1.A, 5mov.1.A, 5mow.2.A, 5mqv.1.A, 5mqv.5.A, 5mrb.1.A, 5mrd.1.A, 5mty.1.A, 5mxx.1.A, 5my8.1.A, 5myv.2.A, 5myv.3.A, 5myv.4.A, 5mz3.1.A, 5mzl.1.A, 5n1g.1.A, 5n1v.1.A, 5n23.1.A, 5n4v.1.A, 5n63.1.A, 5n65.1.A, 5n7v.1.A, 5n84.1.A, 5n93.1.A, 5na0.1.A, 5nad.1.A, 5ncl.1.A, 5ncy.1.A, 5ncz.1.A, 5nev.1.A, 5ng0.1.A, 5ng2.1.A, 5ng3.1.A, 5ngb.1.A, 5ngu.1.A, 5nhh.1.A, 5nk0.1.A, 5nk3.1.A, 5nk5.1.A, 5nk7.1.A, 5nka.1.A, 5nqc.1.A, 5ntj.1.A, 5ntt.1.A, 5nud.1.A, 5nud.2.A, 5nwz.1.A, 5nwz.2.A, 5nxc.1.A, 5nxd.1.A, 5nxd.2.A, 5nzz.1.B, 5nzz.2.B, 5o0y.1.A, 5o11.1.A, 5o1s.1.A, 5o1v.1.A, 5o1v.1.B, 5o21.1.A, 5o23.1.A, 5o23.2.A, 5o26.1.A, 5o2b.1.A, 5o2b.2.A, 5o2c.1.A, 5o7i.1.A, 5o83.1.A, 5o8u.1.A, 5o8v.1.A, 5o90.1.A, 5oat.1.A, 5obj.1.A, 5obr.1.A, 5odt.1.A, 5okt.2.A, 5okt.4.A, 5omg.1.A, 5omy.1.A, 5oni.1.A, 5oo0.1.A, 5oo1.1.A, 5ooi.1.A, 5oop.1.A, 5op2.1.A, 5opu.1.A, 5opv.1.A, 5oq4.1.A, 5oq5.1.A, 5oq6.1.A, 5oq7.1.A, 5oq8.1.A, 5orl.1.A, 5os5.1.A, 5os7.1.A, 5osd.1.A, 5osl.1.A, 5osm.1.A, 5osz.1.A, 5otf.1.A, 5otq.1.A, 5owl.1.A, 5owq.3.B, 5owr.1.A, 5oy4.1.A, 5oy6.3.A, 5oy6.4.A, 5p9f.1.A, 5p9k.1.A, 5p9m.1.A, 5qik.1.A, 5qin.1.A, 5r8z.1.A, 5r9d.1.A, 5s7g.1.A, 5s86.1.B, 5sav.1.A, 5saw.1.A, 5sax.1.A, 5say.1.A, 5say.2.A, 5sb1.1.A, 5sb2.1.A, 5sw8.1.A, 5swh.1.A, 5swt.1.A, 5sxj.1.A, 5sys.1.A, 5t0p.1.A, 5t23.1.A, 5t27.1.A, 5t31.1.A, 5t3q.1.B, 5t68.1.A, 5t7f.1.A, 5t8i.1.A, 5t8o.1.A, 5t8p.1.A, 5t8q.1.A, 5t8q.2.A, 5ta6.1.A, 5tbe.1.A, 5tc0.1.A, 5tc0.2.A, 5tco.1.A, 5td2.1.A, 5td2.2.A, 5te0.1.A, 5teh.1.A, 5teh.2.A, 5tex.1.A, 5tf9.1.A, 5tiu.1.A, 5tkd.1.A, 5to8.1.A, 5tob.1.A, 5toe.1.A, 5tos.1.A, 5tq3.1.A, 5tq4.1.A, 5tq5.1.A, 5tq6.1.A, 5tq7.1.A, 5tqw.1.A, 5tqx.1.A, 5tqy.1.A, 5tr6.1.A, 5ts8.1.A, 5tts.1.A, 5tur.1.A, 5tvt.1.A, 5twl.1.A, 5twy.1.A, 5twy.2.A, 5twz.1.A, 5tx3.2.A, 5u6b.1.A, 5u6b.2.A, 5u6c.1.A, 5u6c.1.B, 5u6y.1.A, 5u6y.1.B, 5u6y.1.C, 5u6y.1.D, 5u6y.1.E, 5u6y.1.F, 5u6y.1.G, 5u6y.1.H, 5u6y.1.I, 5u6y.1.J, 5u6y.1.K, 5u6y.1.L, 5u7q.1.A, 5u7q.2.A, 5u7r.1.A, 5u7r.1.B, 5u7r.2.B, 5u94.1.A, 5u9d.1.A, 5uab.1.A, 5ubr.1.A, 5ufu.1.A, 5ugc.1.A, 5ugl.1.A, 5ugx.1.A, 5uhn.1.A, 5ui0.1.A, 5ui0.2.A, 5uk8.1.B, 5uke.1.A, 5ukf.1.A, 5ukf.2.A, 5ukk.1.A, 5ukl.1.A, 5ukm.1.A, 5ul1.1.B, 5umo.1.A, 5unp.1.A, 5uor.1.A, 5uor.2.A, 5uox.2.A, 5up3.1.A, 5upk.1.B, 5upl.1.A, 5uq1.1.A, 5uq2.1.A, 5uq3.1.A, 5ur1.2.A, 5usq.1.A, 5usy.1.A, 5uu1.1.A, 5uuu.1.A, 5uv4.1.A, 5uvc.1.A, 5uwd.1.A, 5uxa.2.A, 5uxb.1.A, 5uxb.2.A, 5uxc.1.A, 5uxd.1.A, 5uxd.2.A, 5uy6.1.A, 5v5n.1.A, 5v5y.1.A, 5v60.1.A, 5v61.1.A, 5v62.1.A, 5val.2.A, 5vam.1.A, 5vc3.1.A, 5vc5.1.A, 5vc6.1.A, 5vcv.1.A, 5vcw.1.A, 5vcw.2.A, 5vcx.1.A, 5vcy.1.A, 5vd0.1.A, 5vd1.1.A, 5vd2.1.A, 5vd3.1.A, 5vd4.1.A, 5vd5.1.A, 5vd7.1.A, 5vd8.1.A, 5vd9.1.A, 5vda.1.A, 5vdk.1.A, 5ve6.1.A, 5ved.1.A, 5vee.1.A, 5vef.1.A, 5vib.1.A, 5vio.1.A, 5vio.2.A, 5vio.3.A, 5vio.4.A, 5vja.1.B, 5vja.1.C, 5vlo.1.A, 5vlo.2.A, 5vnd.1.A, 5vnd.2.A, 5vt1.1.A, 5vub.1.A, 5w4w.2.A, 5w5j.1.A, 5w5j.1.B, 5w5o.1.A, 5w5v.1.A, 5w7t.1.A, 5w7t.2.A, 5w84.1.A, 5w86.1.A, 5w86.3.A, 5wax.1.A, 5wax.2.A, 5wdy.1.A, 5wdy.2.A, 5we8.1.A, 5we8.2.A, 5wev.1.A, 5wg3.1.A, 5wg5.1.A, 5wij.1.A, 5wjj.1.A, 5wne.1.B, 5wne.2.A, 5wng.1.A, 5wng.1.B, 5wnh.2.A, 5wnh.2.B, 5wni.1.A, 5wno.1.A, 5wp1.1.A, 5wr7.1.A, 5wvd.1.A, 5x02.1.A, 5x17.1.A, 5x17.2.A, 5x18.1.A, 5x28.1.A, 5x2a.1.A, 5x2a.1.B, 5x2f.1.A,

5x2f.2.A, 5x2f.2.B, 5x2k.1.A, 5x3f.1.B, 5x5o.1.A, 5x8i.1.A, 5xd6.1.A, 5xd6.2.A, 5xdl.1.A, 5xff.1.A, 5xfj.1.A, 5xgi.1.A, 5xgj.1.A,
5xgm.1.A, 5xgn.1.A, 5xgn.1.B, 5xka.1.A, 5xp5.1.A, 5xp7.2.A, 5xqx.1.A, 5xv7.1.A, 5xvf.1.A, 5xvu.1.A, 5xy1.1.A, 5xyx.1.A,
5xyz.1.A, 5xzv.1.A, 5xzv.2.A, 5y25.1.A, 5y5t.1.A, 5y5u.1.A, 5y5u.2.A, 5y7z.1.A, 5y80.1.A, 5y86.1.A, 5y8u.1.A, 5y90.1.A,
5y9m.2.A, 5y9t.1.A, 5ya5.1.A, 5yj9.1.A, 5yjz.1.A, 5yks.1.A, 5yt3.1.A, 5yt3.1.B, 5yt3.2.A, 5yu9.3.A, 5yu9.4.A, 5yva.1.A,
5ywm.1.A, 5z1d.1.A, 5z1e.1.A, 5z33.1.A, 5zan.1.A, 5zj6.1.A, 5zjw.1.A, 5zn0.1.A, 5zn2.1.A, 5zn3.1.A, 5zn4.1.A, 5zn5.1.A,
5zto.1.A, 5zv2.1.A, 5zv2.2.A, 5zwj.1.A, 5zxb.1.A, 5zxb.1.B, 5zz4.1.A, 6a1c.1.A, 6a1f.1.A, 6a1g.1.A, 6a32.1.A, 6aaj.1.A,
6aaj.1.B, 6aak.1.B, 6aak.2.A, 6aam.1.A, 6ac0.1.A, 6ac9.1.A, 6ac9.3.A, 6ae3.1.A, 6ae3.2.A, 6agx.1.A, 6agx.1.B, 6ao5.1.A,
6ate.1.A, 6ath.1.A, 6aua.1.A, 6aub.1.A, 6aud.1.A, 6ayd.1.A, 6b1u.1.A, 6b1u.2.A, 6b2e.1.A, 6b2p.1.A, 6b2q.1.A, 6b3e.1.A,
6b4w.1.A, 6b5j.4.A, 6b8j.1.A, 6bab.1.A, 6bab.2.A, 6bbu.1.A, 6bbv.1.A, 6bdl.1.A, 6bdn.1.A, 6bfa.1.A, 6bfn.1.A, 6bfn.2.A,
6bg2.1.A, 6bg2.2.A, 6bg2.3.A, 6bhc.1.A, 6bik.1.A, 6bl8.1.A, 6bl8.2.A, 6ble.1.A, 6bny.1.A, 6bod.1.A, 6boe.1.A, 6bql.1.A,
6bqq.1.A, 6brj.1.A, 6bsd.1.A, 6buu.1.A, 6bwk.1.A, 6bx6.1.A, 6bxi.1.A, 6byr.2.A, 6byr.3.A, 6bys.1.C, 6c0u.1.A, 6c1s.1.A,
6c2t.1.B, 6c2y.1.A, 6c3e.1.A, 6c3e.1.B, 6c4d.1.A, 6c4d.2.A, 6c4d.3.A, 6c4d.4.A, 6c7y.1.A, 6c83.1.B, 6c9d.1.A, 6c9f.1.A,
6cad.1.A, 6cad.2.A, 6ccf.1.A, 6ccy.1.A, 6cd6.1.A, 6cd7.1.A, 6cd7.2.A, 6cdt.1.A, 6ch4.1.A, 6cjw.1.A, 6ckx.2.A, 6cmj.1.A,
6cn9.1.A, 6cn9.2.A, 6cnh.1.A, 6cpe.1.A, 6cpf.1.A, 6cpg.1.A, 6cpw.1.A, 6cpy.1.A, 6cpy.2.A, 6cqd.1.A, 6cqd.1.B, 6cqe.1.A,
6cqf.1.A, 6cth.1.A, 6ctz.1.A, 6cz3.1.A, 6cz4.1.A, 6d1y.1.A, 6d22.1.A, 6d2i.1.A, 6d3k.1.A, 6d3k.1.B, 6d3k.2.B, 6d3l.1.A,
6d5y.1.A, 6dc0.1.A, 6dc0.1.B, 6dd4.2.A, 6dfl.1.A, 6dgt.1.A, 6di5.1.A, 6di9.1.A, 6dkb.1.A, 6dkg.1.A, 6dkw.1.A, 6dkw.2.A,
6dmg.1.A, 6dtl.1.A, 6dtl.2.A, 6duk.1.A, 6duk.3.B, 6e0r.1.A, 6e2o.1.A, 6e2o.1.B, 6e4f.1.A, 6e6e.2.A, 6e6e.4.A, 6e6e.6.A,
6e6e.7.A, 6e9w.1.A, 6e9w.1.B, 6eac.1.A, 6eas.1.A, 6ed6.1.A, 6ed6.2.A, 6edl.1.A, 6ef6.1.A, 6eg9.1.A, 6eg9.1.B, 6ega.1.B,
6egd.1.A, 6egf.1.A, 6ehu.1.A, 6ehu.2.A, 6eif.2.A, 6eij.4.A, 6eil.2.A, 6eim.1.A, 6eim.1.B, 6eiq.1.A, 6eiv.2.A, 6eiv.3.A, 6eix.1.A,
6ej4.1.A, 6ej4.2.A, 6ej4.3.A, 6ej4.4.A, 6ekd.1.A, 6elr.1.A, 6emh.4.A, 6eml.1.4, 6ep9.1.A, 6eq9.1.A, 6eq9.2.A, 6eqi.1.C,
6es0.1.A, 6es0.2.A, 6esa.1.A, 6ewx.1.A, 6ewx.1.B, 6eyz.1.A, 6f14.1.A, 6f1w.1.A, 6f1w.2.A, 6f26.1.A, 6f26.2.A, 6f3d.1.A,
6f3e.2.A, 6f3f.1.A, 6f3i.1.A, 6f5e.1.B, 6f7b.1.A, 6fad.1.A, 6fad.3.A, 6fai.1.J, 6fcf.1.A, 6fd3.1.A, 6fdm.1.A, 6fdm.1.B, 6fdn.1.A,
6fdn.1.B, 6fdo.1.A, 6fdo.2.A, 6fdy.1.A, 6fdz.1.A, 6fek.1.A, 6fer.1.A, 6few.1.A, 6fha.1.A, 6fhb.1.A, 6fil.1.A, 6fio.1.A, 6fiq.1.A,
6fjz.1.A, 6fnf.1.A, 6fng.1.A, 6fnh.1.A, 6fnh.2.A, 6fnh.3.A, 6fni.1.A, 6fnj.1.A, 6fnl.1.A, 6frx.1.A, 6ft7.1.A, 6ft9.3.A, 6fu5.1.B,
6fuc.1.A, 6fvf.1.A, 6fyi.1.A, 6fyk.1.A, 6fyk.2.A, 6fyk.3.A, 6fyl.1.A, 6fyo.1.A, 6fyp.1.A, 6fyr.1.A, 6fyv.1.A, 6g18.1.7, 6g38.1.A,
6g39.1.A, 6g3a.1.A, 6g3c.1.A, 6g4j.1.A, 6g51.1.W, 6g54.1.A, 6g6w.1.A, 6g76.1.A, 6g78.1.A, 6g9a.1.A, 6g9n.1.A, 6gcr.1.A,
6gcw.1.A, 6gcw.1.B, 6gcx.1.A, 6ges.1.A, 6ggh.1.A, 6gi6.1.A, 6gin.1.A, 6gjo.2.A, 6gk2.1.B, 6gl3.1.A, 6gl3.2.A, 6gn1.1.A,
6gqj.1.A, 6gqk.1.A, 6gqo.1.A, 6gr8.1.A, 6gr9.1.A, 6gro.1.A, 6gtt.1.A, 6gu2.1.A, 6gu6.1.A, 6gu7.2.A, 6gub.1.A, 6gub.2.A,
6guc.1.A, 6guc.2.A, 6gue.1.A, 6gue.2.A, 6guf.1.A, 6guf.2.A, 6guh.1.A, 6guk.1.A, 6gva.1.A, 6gvf.1.A, 6gvg.1.A, 6gvj.1.A,
6gvx.1.A, 6gwr.1.A, 6gwr.1.B, 6gy0.1.A, 6gzd.1.A, 6gzh.1.A, 6gzm.1.A, 6gzm.1.B, 6h0u.1.B, 6h3k.1.A, 6hbn.2.A, 6hd6.1.A,
6hd6.2.A, 6hdp.1.A, 6hdr.1.A, 6hh1.1.A, 6hhf.1.A, 6hhg.1.A, 6hhh.1.A, 6hho.1.A, 6hho.2.A, 6hi9.1.A, 6hj7.1.A, 6hjj.1.A,
6hjk.1.A, 6hk3.1.A, 6hk3.2.A, 6hk4.2.A, 6hk6.1.A, 6hk6.2.A, 6hk7.1.A, 6hkm.1.A, 6hkn.1.A, 6hm6.1.A, 6hmd.1.A, 6hme.1.A,
6hmp.1.A, 6hmq.1.A, 6hmr.2.A, 6hmx.1.A, 6hp9.1.A, 6hp9.2.A, 6hv0.1.A, 6hvd.1.A, 6hvf.2.A, 6hwj.1.A, 6hwj.2.A, 6hwk.1.A,
6hwk.1.D, 6hwu.1.A, 6hwv.1.A, 6hx1.1.A, 6hxf.1.A, 6hxf.3.A, 6hzu.2.A, 6hzv.1.A, 6i1s.1.A, 6i2d.1.A, 6i2p.1.A, 6i2y.1.A,
6i2y.1.B, 6i3n.1.A, 6i3u.1.A, 6i5i.1.A, 6i82.1.A, 6i82.1.B, 6i83.1.A, 6i8z.1.A, 6i99.1.A, 6i99.2.A, 6ib0.1.A, 6ib2.1.A, 6ig8.1.A,
6iii.1.A, 6il3.1.A, 6ilz.1.A, 6ilz.2.A, 6ilz.3.A, 6ilz.4.A, 6in0.1.A, 6in4.1.A, 6iqn.1.A, 6iqn.1.B, 6ise.1.A, 6itj.2.A, 6itt.1.A, 6itt.1.B,
6itv.1.A, 6iuo.1.A, 6iup.1.A, 6iy9.1.A, 6iy9.2.A, 6j5l.1.A, 6j5t.1.G, 6j5t.1.N, 6j5u.1.B, 6j5u.1.C, 6j5v.1.B, 6j5v.1.C, 6j5w.1.A,
6j6i.1.A, 6j6i.1.B, 6j6m.1.A, 6jgm.1.A, 6jkk.1.A, 6jlr.1.A, 6jmf.1.A, 6joi.1.A, 6joj.1.A, 6jol.1.A, 6jpe.1.A, 6jpj.1.A, 6jqr.1.A,
6jrj.1.A, 6jrk.1.A, 6jrx.1.A, 6jut.1.A, 6jux.1.A, 6jwl.1.A, 6jx0.1.A, 6jx4.1.A, 6jxt.1.A, 6jz0.1.A, 6k0j.1.A, 6k20.1.A, 6k3l.1.A,
6ka4.1.A, 6khd.1.A, 6khd.2.A, 6khd.3.A, 6khe.1.A, 6khf.1.A, 6kla.1.B, 6kmh.1.A, 6kmh.2.A, 6kyq.1.A, 6kyr.1.A, 6kzc.1.A,
6l20.1.A, 6l20.2.A, 6l20.3.A, 6l20.4.A, 6l21.1.A, 6l22.1.A, 6l23.1.A, 6l24.1.A, 6l8l.1.A, 6l8l.2.A, 6l8l.3.A, 6l8l.4.A, 6lba.1.A,
6lk5.1.A, 6lk6.1.A, 6ln1.1.A, 6ln1.2.A, 6lnm.1.A, 6lub.1.A, 6lvk.1.A, 6lvm.1.A, 6lxy.1.A, 6m0u.1.A, 6m13.1.A, 6m7z.1.A,
6m7z.3.A, 6m7z.4.A, 6m7z.5.A, 6m7z.6.A, 6m95.1.A, 6m9h.1.A, 6m9l.1.A, 6mcq.1.A, 6mcq.2.A, 6mep.1.A, 6mep.1.B,
6mib.1.A, 6mm5.1.A, 6mm6.1.A, 6mnh.1.A, 6mny.1.A, 6mny.2.A, 6mob.1.A, 6mom.1.A, 6mul.1.A, 6mum.1.A, 6mwe.2.A,
6mx8.1.A, 6myn.1.A, 6mzw.1.A, 6n0p.1.A, 6n3l.1.A, 6n3n.1.A, 6n3o.1.A, 6n6o.1.A, 6n7a.1.A, 6n7c.2.A, 6n8g.1.A, 6n8g.3.A,
6nbs.1.A, 6ncg.1.A, 6ncg.2.A, 6nct.1.A, 6ne7.1.A, 6nfi.1.A, 6nfy.1.A, 6nfz.1.A, 6nfz.1.B, 6ng0.1.A, 6ng0.1.B, 6no7.1.A,
6no7.2.A, 6no7.2.C, 6no8.1.A, 6npt.1.A, 6npy.1.B, 6npz.1.A, 6npz.2.A, 6nsp.1.A, 6nss.1.A, 6nt9.1.A, 6nvk.1.A, 6nvl.1.A,
6nvl.2.A, 6nvl.3.A, 6nw2.1.A, 6nw2.2.A, 6nyb.1.A, 6nyb.1.B, 6nyh.1.A, 6nyh.2.A, 6nzm.1.A, 6nzr.1.A, 6o5z.1.A, 6o5z.2.A,
6o6q.1.A, 6o6q.1.B, 6o8b.1.C, 6o8b.1.D, 6o8c.1.A, 6o8c.1.B, 6o8i.1.A, 6o8u.1.A, 6o8u.2.A, 6o8u.3.A, 6o94.1.A, 6o94.2.A,
6o94.3.A, 6o9d.2.A, 6o9l.1.3, 6oac.1.A, 6oco.1.A, 6ocq.1.A, 6ocq.2.A, 6ocu.1.A, 6ohd.1.A, 6oid.1.A, 6oko.1.A, 6ol2.1.A,
6op9.1.A, 6opg.1.A, 6opi.1.A, 6opk.1.A, 6oqi.1.A, 6oqo.1.A, 6ot6.1.A, 6ots.1.A, 6ova.1.A, 6oyt.1.A, 6oyt.1.B, 6oyw.1.A,
6oyw.1.B, 6p1d.1.A, 6p1d.1.B, 6p1d.2.A, 6p1d.2.B, 6p1l.1.B, 6p1l.2.A, 6p3d.1.A, 6p5m.1.A, 6p5m.3.A, 6p5p.4.A, 6p5s.1.A,
6p68.3.A, 6p69.1.A, 6p7g.1.A, 6p7g.1.B, 6p7g.2.B, 6p8e.1.B, 6p8f.1.B, 6p8g.1.B, 6p8h.1.B, 6p8q.1.A, 6p8q.2.A, 6paw.1.A,
6paw.2.A, 6paw.4.A, 6pdj.1.A, 6pjj.1.C, 6pjx.1.A, 6pk6.1.A, 6pl2.1.A, 6pl3.1.A, 6pma.1.A, 6pme.1.B, 6pme.1.D, 6pme.1.L,
6pnx.1.A, 6pnx.1.B, 6pp9.1.A, 6pp9.1.B, 6pwd.1.A, 6pwg.2.A, 6pxn.1.A, 6pxn.2.A, 6pxo.3.A, 6pxo.3.B, 6pxp.1.A, 6pxp.2.A,
6pys.1.A, 6q0j.1.A, 6q0k.1.A, 6q0k.1.B, 6q0t.1.A, 6q0t.1.B, 6q0t.1.C, 6q2a.1.A, 6q2a.15.A, 6q2a.2.A, 6q2a.7.A, 6q38.1.A,
6q3b.1.A, 6q48.1.A, 6q4d.1.A, 6q4g.1.A, 6q4h.1.A, 6q4i.1.A, 6q4k.1.A, 6q4q.1.A, 6q8k.1.A, 6qaq.1.A, 6qas.1.A, 6qas.2.A,
6qat.1.A, 6qat.2.A, 6qau.1.A, 6qau.2.A, 6qav.1.A, 6qav.2.A, 6qav.2.B, 6qfl.1.A, 6qfr.1.A, 6qmo.1.A, 6qn4.1.A, 6qp5.1.A,
6qtg.1.A, 6qx9.56.A, 6qxk.1.A, 6qy7.1.A, 6qy9.1.A, 6qyx.1.A, 6r3d.1.A, 6r3s.1.A, 6r5f.2.A, 6r5f.3.A, 6r5f.4.A, 6r5k.1.F,
6r5k.1.G, 6ra5.1.A, 6ra7.1.A, 6rbd.1.W, 6rcg.1.A, 6rch.1.A, 6rch.1.B, 6rct.2.A, 6rfi.2.A, 6rfo.1.A, 6rfp.1.A, 6rln.1.A, 6rsb.1.A,
6rsr.1.A, 6rst.1.A, 6rsu.1.A, 6ru8.2.A, 6ru8.3.A, 6ruu.1.A, 6ruu.1.B, 6ruu.2.A, 6s11.1.A, 6s11.2.A, 6s1f.1.B, 6s1i.3.A, 6s1i.4.A,
6s1j.1.A, 6s73.1.A, 6s73.2.A, 6s73.3.A, 6s73.4.A, 6s75.1.A, 6s75.2.A, 6s75.3.A, 6s75.4.A, 6s76.1.A, 6s76.2.A, 6s76.3.A,
6s76.4.A, 6s90.1.A, 6s90.2.A, 6s9b.1.A, 6s9c.1.A, 6s9d.1.A, 6s9x.1.A, 6sd9.1.A, 6sdc.1.A, 6sdd.1.A, 6sde.1.A, 6seq.1.A,
6sfk.1.A, 6sfo.1.A, 6sg4.1.A, 6sg4.1.C, 6sgd.1.A, 6so4.1.A, 6soi.1.A, 6sov.1.A, 6spw.1.A, 6srh.1.A, 6srh.2.A, 6sui.1.A,
6sul.1.A, 6sum.1.A, 6sun.1.A, 6t28.1.A, 6t2w.1.A, 6t41.1.A, 6t6a.2.A, 6t6d.3.A, 6t6d.4.A, 6t6f.1.A, 6t6f.2.A, 6t8x.1.A, 6t8x.1.D,
6t8x.1.F, 6tca.1.A, 6tca.1.B, 6tca.2.A, 6tca.2.B, 6tca.3.A, 6tca.3.B, 6tca.4.A, 6tcu.1.A, 6td3.1.B, 6tew.1.A, 6tfp.1.A, 6tfp.2.A,
6tfp.4.A, 6tfu.1.A, 6tfv.2.A, 6tgu.1.A, 6ti8.1.A, 6ti8.4.A, 6tia.1.A, 6tia.2.A, 6tll.1.A, 6tlo.1.A, 6tn9.1.A, 6tnb.1.A, 6tnc.1.A,
6tnd.1.A, 6tpa.1.A, 6tpd.1.A, 6tpe.1.A, 6tpf.1.A, 6tsz.1.A, 6tu9.1.A, 6tua.1.A, 6ty3.1.A, 6ty4.1.A, 6u0k.1.A, 6u2g.1.A, 6u2g.1.B,

6u2h.1.C, 6u2h.1.D, 6u5l.1.A, 6u69.1.A, 6u6a.1.A, 6uan.1.C, 6uan.1.D, 6uip.1.A, 6uip.3.A, 6ul8.1.A, 6umw.1.A, 6una.1.A, 6una.2.A, 6unp.1.A, 6unq.1.A, 6unr.1.A, 6urc.2.A, 6uuo.2.A, 6uuo.2.B, 6v2u.1.A, 6v2u.2.A, 6v2w.1.A, 6v2w.1.B, 6v34.1.A, 6v34.2.A, 6v5n.1.A, 6v5n.1.B, 6v5n.2.A, 6v5p.2.A, 6v66.1.A, 6v66.2.A, 6v6a.1.A, 6v6k.1.B, 6v6o.1.B, 6v6o.3.A, 6v6o.3.B, 6v6o.4.B, 6v6q.1.A, 6vbz.1.A, 6vbz.2.A, 6vc0.1.A, 6vc0.2.A, 6vc0.3.A, 6vg3.1.A, 6vg3.2.A, 6vg3.3.A, 6vgl.1.A, 6vgl.2.A, 6vhg.1.A, 6vnc.1.A, 6vnc.2.A, 6vne.1.A, 6vne.2.A, 6vng.2.A, 6vno.1.A, 6vov.1.A, 6vp6.1.B, 6vp8.1.A, 6vp8.1.B, 6vpg.1.A, 6vph.1.B, 6vpi.1.A, 6vpj.1.A, 6vpl.1.A, 6vpm.1.B, 6vqm.1.A, 6vre.1.A, 6vrf.1.A, 6vru.1.A, 6vvc.1.A, 6vvd.1.A, 6vvd.2.A, 6vvd.3.A, 6vve.1.A, 6vvg.1.A, 6vvg.1.B, 6vxr.1.A, 6vzk.1.A, 6w39.1.B, 6w3a.1.A, 6w3a.1.B, 6w3b.1.A, 6w3c.1.A, 6w3c.2.A, 6w3e.1.A, 6w3k.1.A, 6w4o.1.M, 6w7o.1.A, 6w8i.1.A, 6w8i.2.A, 6w8i.3.A, 6w9e.1.A, 6wa2.1.B, 6wa2.2.A, 6wa2.2.B, 6wak.1.A, 6wak.2.A, 6wak.2.B, 6whp.1.A, 6wjf.1.A, 6wjf.1.B, 6wpp.1.A, 6wpp.2.A, 6wqx.1.B, 6wqx.2.B, 6wtn.1.A, 6wxj.1.A, 6wxn.3.A, 6x5g.1.A, 6x8e.2.A, 6x8g.1.A, 6xag.1.C, 6xbz.1.C, 6xd3.1.C, 6xdb.1.A, 6xdd.1.B, 6xdd.2.A, 6xdf.1.A, 6xdf.1.B, 6xfp.1.A, 6xi8.1.B, 6xih.1.A, 6xih.2.A, 6xka.1.A, 6xl4.1.A, 6xl4.2.A, 6xl4.3.A, 6xl4.4.A, 6xlo.1.A, 6xr6.1.A, 6xr7.1.A, 6xrg.1.A, 6xrl.1.A, 6xrm.1.A, 6xrn.1.A, 6xv9.2.A, 6xx6.1.A, 6xx8.1.A, 6y23.1.A, 6y23.2.A, 6y4x.1.A, 6y6f.1.A, 6y6v.1.A, 6y7c.1.6, 6y7z.1.A, 6y9r.1.A, 6y9s.2.A, 6ya6.1.A, 6ya7.1.A, 6ya8.1.A, 6yat.1.A, 6yfz.1.A, 6yg0.1.A, 6yg1.1.A, 6yg2.1.A, 6yg3.1.A, 6yg4.1.A, 6yg6.1.A, 6yg6.2.A, 6yg7.1.A, 6ygn.1.A, 6ygn.2.A, 6yi8.1.A, 6yi8.2.A, 6yid.1.B, 6yid.2.A, 6ykd.1.A, 6ykg.1.A, 6yky.1.A, 6yky.2.A, 6yky.3.A, 6yky.4.A, 6yl1.1.A, 6ylc.1.A, 6ylk.1.A, 6yll.1.A, 6yll.2.A, 6yoj.1.A, 6yph.1.A, 6yq1.1.A, 6yr9.3.A, 6yt6.1.A, 6yt6.2.A, 6ytd.1.A, 6yty.1.A, 6yul.2.A, 6yvs.2.A, 6yvs.3.A, 6yvs.4.A, 6yvy.1.A, 6yvy.4.A, 6yxs.1.A, 6yxt.1.A, 6yxv.4.A, 6yzh.1.A, 6z0f.1.A, 6z0f.1.B, 6z19.1.A, 6z1c.1.A, 6z1q.1.A, 6z3u.1.B, 6z45.1.A, 6z4b.1.A, 6z4b.2.A, 6z50.1.A, 6z53.1.A, 6z55.1.A, 6z59.1.A, 6z83.1.A, 6z84.2.A, 6zgc.3.A, 6ziw.1.A, 6zjf.1.A, 6zqs.1.A, 6zr5.1.A, 6zr5.2.A, 6zv6.1.8, 6zxd.1.9, 6zxe.1.a, 6zxf.1.8, 6zxg.1.a, 7a04.1.A, 7a06.1.B, 7a2a.2.A, 7a49.2.A, 7a4b.1.A, 7a4o.1.A, 7a5b.2.A, 7a6i.1.A, 7aax.1.A, 7aay.1.A, 7aaz.1.A, 7ab1.1.A, 7aem.1.A, 7aj2.1.A, 7ajm.1.A, 7ajm.2.A, 7ajv.1.A, 7ajy.2.A, 7akm.1.A, 7akm.2.A, 7ako.1.A, 7ako.2.A, 7apf.1.A, 7apg.2.A, 7apj.1.A, 7aqb.1.A, 7aqb.2.A, 7at5.2.A, 7at9.1.A, 7ats.1.A, 7atu.1.A, 7atv.1.A, 7avx.1.A, 7avx.2.A, 7avy.1.A, 7aw4.2.A, 7ax4.1.A, 7ayi.1.A, 7aym.1.A, 7b30.1.A, 7b36.1.A, 7b3m.1.A, 7b3q.1.A, 7b3w.1.A, 7b55.1.A, 7b5l.1.J, 7b5q.1.C, 7b6f.1.A, 7b7s.1.A, 7b85.1.A, 7b8h.1.A, 7bcm.1.A, 7be6.1.A, 7bi2.1.A, 7bi4.1.A, 7bi6.1.A, 7bl1.1.B, 7bl1.1.C, 7bmk.1.A, 7bmk.1.B, 7btt.1.A, 7bu4.1.A, 7byk.1.A, 7c2v.1.A, 7c2v.1.B, 7c2w.1.A, 7c2w.1.B, 7c2w.1.C, 7c2w.1.D, 7c3n.1.A, 7cbx.1.A, 7cc2.1.A, 7cc2.2.A, 7cga.1.A, 7cga.2.A, 7cga.3.A, 7cga.4.A, 7chm.1.A, 7chn.1.A, 7cht.1.A, 7cja.1.A, 7clh.1.A, 7cmb.1.A, 7cml.1.A, 7cp3.1.A, 7cp4.1.A, 7cqe.1.A, 7csq.1.A, 7csq.1.B, 7ctv.1.A, 7ctv.2.A, 7ctx.1.A, 7cy2.1.A, 7cyr.1.A, 7cz2.1.A, 7d5o.1.A, 7dd1.1.A, 7dg4.1.A, 7dh3.1.A, 7dhv.1.A, 7dsy.1.A, 7dt2.1.A, 7dts.1.A, 7dts.1.B, 7dtz.1.A, 7du8.1.B, 7du9.1.A, 7du9.1.B, 7dua.1.A, 7dv6.1.A, 7dxl.1.A, 7e0z.1.A, 7e11.1.A, 7e34.1.A, 7e73.1.A, 7e75.1.A, 7eec.1.A, 7eed.1.A, 7eef.1.A, 7egc.1.I, 7ejv.1.A, 7ejv.1.B, 7elw.1.B, 7ena.2.A, 7enc.24.A, 7er2.1.A, 7f0a.1.A, 7f0b.1.A, 7f2x.1.A, 7f3g.1.A, 7f3g.1.B, 7f3m.1.A, 7f7w.1.A, 7f7w.2.A, 7fcz.1.A, 7fcz.1.B, 7fd0.1.A, 7feh.1.A, 7fhs.1.A, 7fht.4.A, 7fic.1.A, 7jhg.1.A, 7jhh.1.A, 7jij.1.B, 7jiu.1.A, 7jnt.1.A, 7jou.1.A, 7jov.4.B, 7ju6.1.B, 7juq.1.B, 7jur.1.B, 7jus.1.B, 7jut.1.A, 7jut.1.B, 7juv.1.A, 7juv.1.B, 7juw.1.A, 7jux.1.A, 7juy.1.B, 7jv1.1.B, 7jv7.1.A, 7jw7.1.A, 7jwz.1.A, 7jx0.1.A, 7jxh.1.A, 7jxh.5.A, 7jxk.1.A, 7jxl.1.A, 7jxm.1.A, 7jxu.2.A, 7jxx.1.A, 7jxy.1.A, 7jxy.2.A, 7k0v.1.A, 7k0v.1.B, 7k0v.1.C, 7k0v.1.D, 7k1h.1.A, 7k1h.1.B, 7k1h.2.A, 7k1h.2.B, 7k1h.3.B, 7k1i.1.A, 7k6m.1.A, 7k7l.1.A, 7k7z.1.A, 7kac.1.A, 7kac.1.B, 7khj.1.A, 7khj.2.A, 7khk.1.A, 7khk.2.A, 7kia.1.A, 7kja.1.A, 7kja.2.A, 7kjb.1.A, 7kjc.1.A, 7kjc.2.A, 7kjs.1.A, 7kke.1.A, 7kl1.1.A, 7kp6.1.A, 7kp6.2.A, 7kpl.1.A, 7kpv.1.A, 7ksj.1.A, 7kue.1.B, 7kx6.1.A, 7kx6.2.A, 7kx8.1.A, 7kx8.2.A, 7kxl.1.A, 7kxq.1.A, 7kxw.1.A, 7kxw.2.A, 7kxz.1.A, 7l24.1.A, 7l24.2.A, 7l25.1.A, 7l26.1.A, 7l26.4.A, 7l5p.1.A, 7l5p.2.A, 7lbm.1.8, 7lg8.1.A, 7lg8.2.A, 7lg8.3.A, 7lg8.4.A, 7lgs.2.A, 7lht.1.A, 7lhw.1.A, 7li3.1.A, 7ll4.1.A, 7ll5.1.A, 7lm2.1.A, 7lqd.1.A, 7lty.1.A, 7lv3.1.A, 7lv3.1.B, 7lvh.1.A, 7lvh.2.A, 7m0k.1.A, 7m0l.1.A, 7m0m.1.A, 7m0m.1.B, 7m5z.1.A, 7m5z.2.A, 7m74.1.A, 7mf0.1.A, 7mfd.1.A, 7mfd.1.B, 7mfe.1.A, 7mff.1.C, 7mff.1.D, 7mgj.1.A, 7mgk.4.A, 7mon.1.A, 7mon.1.B, 7mp8.1.A, 7mt8.1.A, 7mt9.1.A, 7mta.1.A, 7mtb.1.A, 7mu7.1.A, 7mu7.2.A, 7mx3.1.A, 7mx3.2.A, 7mx3.3.A, 7mx3.4.A, 7mxb.1.A, 7mxb.2.A, 7mxj.1.A, 7mxk.1.A, 7myj.2.A, 7myn.1.A, 7myo.1.B, 7n3u.1.A, 7n4r.1.A, 7n8t.1.A, 7n91.1.A, 7n91.2.A, 7n93.2.A, 7n9g.1.A, 7n9g.2.A, 7naa.1.A, 7nb1.1.A, 7ncf.1.A, 7ng7.1.A, 7nj0.1.B, 7nqw.1.A, 7nry.1.B, 7nti.1.A, 7nur.1.A, 7nvq.1.A, 7nwk.1.A, 7nxj.1.A, 7nxj.2.A, 7nxk.1.A, 7nxk.2.A, 7o2v.1.A, 7o7i.1.A, 7o7j.1.A, 7o7k.1.A, 7o7k.2.A, 7oai.1.A, 7oam.1.A, 7oam.2.A, 7oi4.1.A, 7oow.1.A, 7oox.1.A, 7opm.1.A, 7opo.1.A, 7opo.4.A, 7opo.5.A, 7ore.1.A, 7orf.1.A, 7ote.1.A, 7ote.2.A, 7ovi.1.A, 7ovj.1.A, 7ovm.1.A, 7oxb.1.A, 7oy5.1.A, 7oy6.1.A, 7ozy.1.A, 7ozy.2.A, 7p1l.1.A, 7p3v.1.A, 7p3v.1.B, 7p5z.1.M, 7p7f.1.A, 7p7g.1.A, 7p7g.2.A, 7p7h.1.A, 7p7h.2.A, 7pcd.1.A, 7pg5.1.A, 7pi4.1.D, 7pqv.1.A, 7psu.1.A, 7pt6.1.O, 7pt7.1.G, 7pue.1.A, 7pvu.1.A, 7pvu.2.A, 7pwd.1.A, 7q4a.1.A, 7q4a.1.B, 7q52.1.A, 7q6h.1.A, 7q7i.1.A, 7q7l.1.A, 7q8v.1.A, 7q8y.1.A, 7q8y.2.A, 7qfm.1.A, 7qg2.1.A, 7qg2.4.A, 7qg3.1.A, 7qg5.2.A, 7qgp.1.A, 7qhw.1.A, 7qhw.2.A, 7qq6.1.A, 7qq6.1.B, 7qq6.2.A, 7qq6.2.B, 7qr9.3.A, 7que.1.A, 7que.2.A, 7quf.1.A, 7qux.1.A, 7qwk.1.A, 7qwk.5.A, 7qwk.6.A, 7r9l.1.B, 7r9n.1.B, 7r9p.1.A, 7r9p.1.B, 7r9t.1.A, 7r9t.1.B, 7ra5.1.A, 7rj8.1.A, 7rn6.1.A, 7rsj.1.A, 7rsp.1.A, 7run.1.A, 7rwf.1.A, 7s25.1.A, 7s25.1.B, 7s25.2.A, 7s25.2.B, 7s26.1.A, 7s26.1.B, 7s26.2.A, 7s26.2.B, 7s3l.1.A, 7s46.1.A, 7s47.1.A, 7s48.1.A, 7s4t.1.A, 7s6u.1.A, 7s85.1.A, 7sa0.1.A, 7shv.1.A, 7siu.1.A, 7siu.1.B, 7sj3.1.A, 7sqm.1.A, 7sxf.1.A, 7sxg.1.A, 7sxh.1.A, 7sxj.1.A, 7sza.1.A, 7szc.1.A, 7szd.1.A, 7szr.1.A, 7szr.2.A, 7t0p.1.A, 7t3x.1.A, 7t4i.1.A, 7t4j.1.A, 7t4j.1.B, 7t4k.1.A, 7t4k.1.B, 7t4l.1.B, 7t4m.1.A, 7t4m.1.B, 7t4t.1.A, 7t4t.2.A, 7t4u.1.A, 7t4u.2.A, 7t4v.1.A, 7t4v.2.A, 7t6f.1.A, 7teu.1.A, 7tnh.1.A, 7tvd.1.A, 7tz7.1.B, 7u2z.1.A, 7u2z.2.A, 7u31.1.A, 7u98.3.A, 7u9a.1.A, 7udp.1.A, 7udq.1.A, 7udq.2.A, 7ug1.1.A, 7ugb.1.A, 7uiq.2.A, 7ujr.1.A, 7ujs.1.A, 7ujt.1.A, 7ukv.1.A, 7ukw.1.A, 7ukw.1.B, 7ukw.2.A, 7ukw.2.B, 7ukz.1.A, 7uos.1.A, 7up4.1.A, 7up4.2.A, 7up6.1.A, 7upm.1.A, 7uxi.1.A, 7uxk.1.A, 7uy0.1.A, 7uy0.2.A, 7uy3.1.A, 7uyr.1.A, 7uyv.1.A, 7uyv.3.A, 7uyw.1.A, 7v3r.1.A, 7v3v.1.M, 7vbt.1.A, 7vdp.1.A, 7vdp.2.A, 7vdq.1.A, 7vjl.1.A, 7vjl.2.A, 7vkm.1.A, 7vkn.1.A, 7vko.1.A, 7vnx.1.A, 7w15.2.A, 7w19.1.A, 7w19.2.A, 7w1a.1.A, 7w1a.2.A, 7w5c.1.A, 7w5o.1.A, 7w5o.2.A, 7w7x.1.A, 7w7x.1.B, 7w7y.1.A, 7w7y.1.B, 7wcl.1.A, 7wcl.2.A, 7wcw.1.A, 7wcx.1.A, 7wf5.2.A, 7wj6.1.A, 7wj6.1.B, 7wtt.1.6, 7wu0.1.8, 7x4u.1.A, 7xaf.1.A, 7xbi.1.A, 7xbr.1.A, 7xbr.1.B, 7xbr.1.C, 7xbr.1.D, 7xbr.2.A, 7xbr.2.B, 7xbr.2.C, 7xbr.2.D, 7xdv.1.A, 7xdv.2.A, 7xdx.1.A, 7xdy.1.A, 7xdy.2.A, 7xmk.1.A, 7xmk.1.B, 7xqk.1.A, 7xsv.1.A, 7xyh.2.A, 7xzq.1.A, 7xzr.1.A, 7xzr.2.A, 7y1g.1.A, 7y1g.2.A, 7y4t.1.A, 7yaw.1.A, 7yaw.2.A, 7yaw.3.A, 7yaz.1.A, 7ybo.1.A, 7yc9.1.A, 7ydx.1.B, 7yl1.1.A, 7z1f.1.A, 7z1g.1.A, 7z37.1.C, 7z38.1.C, 7z4v.1.A, 7z5n.1.A, 7z5n.2.A, 7z5n.7.A, 7z5n.8.A, 7z5w.1.A, 7z61.1.A, 7z6i.1.A, 7z74.1.A, 7z9t.1.A, 7z9t.1.B, 7zh8.1.A, 7zh8.3.A, 7zks.1.A, 7zkx.1.A, 7zr0.1.D, 7zr5.1.D, 7zr6.1.D, 7zvs.1.A, 7zvs.2.A, 7zvs.3.A, 7zw8.1.A, 7zwe.1.A, 8a27.1.A, 8a2a.1.A, 8a2b.1.A, 8a2d.1.A, 8a5j.1.A, 8a66.1.A, 8a8m.1.A, 8a8m.1.B, 8aau.1.A, 8acm.1.A, 8afr.1.A, 8agy.1.A, 8ahd.1.A, 8ahg.1.A, 8ahp.1.A, 8ak2.1.A, 8am0.1.B, 8an8.1.A, 8ans.1.A, 8aof.1.A, 8arj.1.A, 8atl.1.A, 8atl.2.A, 8atn.1.A, 8atn.2.A, 8au3.1.A, 8au3.2.A, 8au5.1.A, 8av1.1.A, 8aw1.1.A, 8aza.1.B, 8aza.1.C, 8b8n.1.A, 8ba2.1.A, 8ba3.1.A, 8ba4.1.A, 8bab.1.A, 8bem.1.A,

8bfu.1.A, 8bi5.1.A, 8bjt.1.A, 8bju.1.A, 8bm2.1.A, 8bm2.2.A, 8boc.1.A, 8bok.1.A, 8bpv.1.A, 8bpw.1.A, 8bu1.1.B, 8bu1.2.B, 8bu2.1.B, 8bu3.3.B, 8bu4.3.B, 8bu5.1.B, 8bu5.2.B, 8bu5.3.B, 8bu6.1.B, 8bu6.2.B, 8bu6.3.B, 8bu7.1.B, 8bua.1.B, 8bua.2.B, 8bub.1.B, 8bub.2.C, 8bub.3.C, 8bug.3.B, 8bui.1.B, 8bui.2.B, 8buj.3.B, 8bum.1.B, 8buo.1.B, 8bus.1.B, 8bvw.1.I, 8bx6.1.A, 8bxc.1.A, 8bya.1.A, 8byq.1.I, 8bzo.1.A, 8bzp.1.A, 8c01.1.3, 8c7x.1.A, 8c7x.1.B, 8c7y.1.A, 8c7y.1.B, 8car.1.A, 8cav.1.A, 8cav.1.B, 8cbj.1.J, 8cdw.1.A, 8cdw.1.B, 8cgc.1.A, 8cie.1.A, 8cij.1.A, 8cur.1.A, 8czo.1.A, 8d6e.1.A, 8d73.1.A, 8d7m.1.A, 8d7n.2.A, 8d7o.2.A, 8d7p.1.A, 8dcp.1.A, 8dcx.1.A, 8dd4.1.A, 8dd8.1.A, 8deg.1.A, 8dgs.1.A, 8dgs.1.B, 8dp5.1.A, 8dp5.1.B, 8ds6.1.A, 8ds6.1.B, 8dso.1.A, 8dsw.1.A, 8dwn.1.A, 8e04.1.A, 8e05.1.A, 8e06.1.A, 8e1x.1.A, 8e1x.1.B, 8e4t.1.A, 8edh.1.A, 8edh.2.A, 8efj.1.A, 8ej4.1.K, 8ejb.1.A, 8elc.1.A, 8eme.1.B, 8enj.1.A, 8eq5.1.A, 8eq9.1.A, 8eqd.1.A, 8erd.1.A, 8ewy.1.B, 8ex2.1.A, 8f1w.4.A, 8f1z.1.A, 8f7o.1.A, 8f7o.1.B, 8f7p.1.A, 8f7p.1.B, 8fac.1.A, 8fd9.1.A, 8fe2.1.A, 8fe2.2.A, 8fe5.2.A, 8fe9.1.A, 8fec.1.A, 8fec.2.A, 8ff0.1.A, 8fh4.1.A, 8fh4.2.A, 8fh4.3.A, 8fh4.4.A, 8fjz.1.A, 8fjz.1.B, 8fko.2.A, 8fko.3.A, 8flg.1.A, 8fll.1.A, 8fln.1.A, 8fow.1.A, 8fp1.1.A, 8fp3.1.A, 8fx4.1.D, 8g6z.1.A, 8g8o.1.A, 8gae.1.D, 8gds.2.A, 8gft.1.D, 8gmb.1.A, 8gmc.1.A, 8gmc.2.A, 8gua.1.A, 8gub.1.A, 8gud.1.A, 8guw.1.A, 8guw.1.B, 8guw.1.C, 8gw3.1.A, 8gw3.3.A, 8gxq.48.A, 8h4r.1.A, 8h59.1.A, 8h6p.1.A, 8h75.1.A, 8h75.1.B, 8h75.1.C, 8h75.1.D, 8h7f.1.A, 8h7f.2.A, 8haq.1.A, 8haq.2.A, 8hlt.1.A, 8hlt.2.A, 8hmt.1.A, 8hmt.2.A, 8ho6.1.A, 8hoa.1.A, 8hod.1.A, 8hoe.1.A, 8hv2.1.A, 8hv4.1.A, 8hv5.1.A, 8hy7.1.A, 8ilr.1.A, 8ilv.1.A, 8iyj.505.A, 8iyj.506.A, 8iyj.507.A, 8j5w.1.A, 8j5x.1.A, 8j61.1.A, 8j63.1.A, 8jmx.1.A, 8jpb.1.A, 8k5r.1.A, 8or0.1.G, 8or4.1.G, 8orm.1.C, 8otz.14.A, 8otz.15.A, 8otz.573.A, 8ouv.1.A, 8ov7.1.A, 8ow2.1.A, 8ow3.1.A, 8owg.3.A, 8oy2.1.A, 8p08.1.A, 8p4z.1.A, 8p4z.2.A, 8p6v.1.C, 8p7j.1.A, 8p7j.2.A, 8p81.1.A, 8pvp.1.A, 8pyi.1.A, 8pyj.1.A, 8pyk.1.A, 8pyl.1.A, 8q1z.1.A, 8q61.1.A, 8q6f.1.A, 8q77.1.A, 8qbu.1.A, 8qcg.1.A, 8qcg.2.A, 8qel.1.B, 8qf1.1.B, 8qlq.1.A, 8qlr.1.A, 8qqy.1.A, 8s93.1.A, 8s99.3.A, 8s9f.1.A, 8s9f.1.B, 8sam.1.A, 8sam.2.A, 8sao.1.B, 8sao.2.B, 8sap.1.A, 8sap.2.A, 8sbc.1.B, 8six.1.A, 8slz.1.B, 8sor.1.D, 8srq.1.E, 8ssn.1.A, 8ssn.2.A, 8sso.1.A, 8ssp.1.A, 8stg.1.A, 8stg.2.A, 8tb5.1.A, 8tdu.1.B, 8tdu.2.A, 8tdu.2.B, 8tgd.1.A, 8tgd.1.B, 8ts7.1.B, 8ts8.1.A, 8ts9.1.A, 8ts9.1.B, 8tsc.1.B, 8tu6.1.A, 8txz.1.A, 8tyq.1.A, 8tzb.1.A, 8tzc.1.A, 8tze.1.A, 8tzf.1.A, 8tzg.1.A, 8tzh.1.B, 8u2o.1.A, 8uap.1.A, 8uaq.1.A, 8wd4.1.A, 8wf4.1.A, 8wf4.2.A, 8wgf.1.A, 8wjy.1.A, 8wsw.4.A, 8wtf.1.A, 8x88.1.A, 8x88.2.A
