# Supplementary material for: Structural and functional characterization of peste des petits ruminants virus coded hemagglutinin protein using various in-silico approaches
Source: Front Microbiol. 2024 Jun 20;15:1427606. doi: 10.3389/fmicb.2024.1427606 (PMC11222573; doi:10.3389/fmicb.2024.1427606)
Supplement: Supplementary file 9 [file Data_Sheet_9.ZIP › TAB-1.pdf]

SWISS-MODEL Homology Modelling Report

Model Building Report

This document lists the results for the homology modelling project "Untitled Project" submitted to SWISS-MODEL workspace on Jan. 12, 2024, 7:43 a.m..The submitted primary amino acid sequence is given in Table T1.

If you use any results in your research, please cite the relevant publications:

- Waterhouse, A., Bertoni, M., Bienert, S., Studer, G., Tauriello, G., Gumienny, R., Heer, F.T., de Beer, T.A.P., Rempfer, C., Bordoli, L., Lepore, R., Schwede, T. SWISS-MODEL: homology modelling of protein structures and complexes. Nucleic Acids Res. 46(W1), W296-W303 (2018).
- Bienert, S., Waterhouse, A., de Beer, T.A.P., Tauriello, G., Studer, G., Bordoli, L., Schwede, T. The SWISS-MODEL Repository - new features and functionality. Nucleic Acids Res. 45, D313-D319 (2017).
- Studer, G., Tauriello, G., Bienert, S., Biasini, M., Johner, N., Schwede, T. ProMod3 - A versatile homology modelling toolbox. PLOS Comp. Biol. 17(1), e1008667 (2021).
- Studer, G., Rempfer, C., Waterhouse, A.M., Gumienny, G., Haas, J., Schwede, T. QMEANDisCo - distance constraints applied on model quality estimation. Bioinformatics 36, 1765-1771 (2020).
- Bertoni, M., Kiefer, F., Biasini, M., Bordoli, L., Schwede, T. Modeling protein quaternary structure of homo- and hetero-oligomers beyond binary interactions by homology. Scientific Reports 7 (2017).

Results

The SWISS-MODEL template library (SMTL version 2023-12-24, PDB release 2023-12-15) was searched with for evolutionary related structures matching the target sequence in Table T1. For details on the template search, see Materials and Methods. Overall 215 templates were found (Table T2).

Models

The following model was built (see Materials and Methods "Model Building"):

Model #01	File	Built with	Oligo-State	Ligands	GMQE	QMEANDisCo Global
	PDB	ProMod3 3.4.0	monomer	None	0.80	0.85 ± 0.05

Template	Seq Identity	Oligo-state	QSQE	Found by	Method	Resolution	Seq Similarity	Range	Coverage	Description
5nzz.2.A	100.00	monomer	0.00	HHblits	X-ray	2.60Å	0.61	11 - 412	1.00	TGF-beta-activated kinase 1 and MAP3K7-binding protein 1

Excluded ligands

Ligand Name.Number	Reason for Exclusion	Description
AGS.2	Binding site not conserved.	PHOSPHOTHIOPHOSPHORIC ACID-ADENYLATE ESTER
MG.3	Binding site not conserved.	MAGNESIUM ION
NI.1	Not in contact with model.	NICKEL (II) ION
NI.4	Binding site not conserved.	NICKEL (II) ION

```
Target     MAAQRRSLLQSEQQPSWTDDLPLCHLSGVGSASNRSYSADGKGTESHPPEDSWLKFRSENNCFLYGVFNGYDGNRVTNFV
5nzz.2.A   MAAQRRSLLQSEQQPSWTDDLPLCHLSGVGSASNRSYSADGKGTESHPPEDSWLKFRSENNCFLYGVFNGYDGNRVTNFV

Target     AQRLSAELLLGQLNAEHAEADVRRVLLQAFDVVERSFLESIDDALAEKASLQSQLPEGVPQHQLPPQYQKILERLKTLER
5nzz.2.A   AQRLSAELLLGQLNAEHAEADVRRVLLQAFDVVERSFLESIDDALAEKASLQSQLPEGVPQHQLPPQYQKILERLKTLER

Target     EISGGAMAVVAVLLNNKLYVANVGTNRALLCKSTVDGLQVTQLNVDHTTENEDELFRLSQLGLDAGKIKQVGIICGQEST
5nzz.2.A   EISGGAMAVVAVLLNNKLYVANVGTNRALLCKSTVDGLQVTQLNVDHTTENEDELFRLSQLGLDAGKIKQVGIICGQEST

Target     RRIGDYKVKYGYTDIDLLSAAKSKPIIAEPEIHGAQPLDGVTGFLVLMSEGLYKALEAAHGPGQANQEIAAMIDTEFAKQ
```

```
5nzz.2.A   RRIGDYKVKYGYTDIDLLSAAKSKPIIAEPEIHGAQPLDGVTGFLVLMSEGLYKALEAAHGPGQANQEIAAMIDTEFAKQ

Target     TSLDAVAQAVVDRVKRIHSDTFASGGERARFCPRHEDMTLLVRNFGYPLGEMSQPTPSPAPAAGGRVYPVSVPYSSAQST
5nzz.2.A   TSLDAVAQAVVDRVKRIHSDTFASGGERARFCPRHEDMTLLVRNFGYPLGEMSQPTPSPAPAAGGRVYPVSVPYSSAQST

Target     SKTSVTLSLVMPSQGQMVNGAHSASTLDEATPTLTNQSPTLTLQSTNTHTQSSSSSSDGGLFRSRPAHSLPPGEDGRVEP
5nzz.2.A   SKTSVTLSLVMPSQGQMVNGAHSASTLDEATPTLTNQSPTLTLQSTNTHTQSSSSSSDGGLFRSRPAHSLPPGEDGRVEP

Target     YVDFAEFYRLWSVDHGEQSVVTAP
5nzz.2.A   YVDFAEFYRLWSVDHGEQSVVTAP
```

Materials and Methods

Template Search

Template search with has been performed against the SWISS-MODEL template library (SMTL, last update: 2023-12-24, last included PDB release: 2023-12-15).

Model Building

Models are built based on the target-template alignment using ProMod3 (Studer et al.). Coordinates which are conserved between the target and the template are copied from the template to the model. Insertions and deletions are remodelled using a fragment library. Side chains are then rebuilt. Finally, the geometry of the resulting model is regularized by using a force field.

Model Quality Estimation

The global and per-residue model quality has been assessed using the QMEAN scoring function (Studer et al.).

Ligand Modelling

Ligands present in the template structure are transferred by homology to the model when the following criteria are met: (a) The ligands are annotated as biologically relevant in the template library, (b) the ligand is in contact with the model, (c) the ligand is not clashing with the protein, (d) the residues in contact with the ligand are conserved between the target and the template. If any of these four criteria is not satisfied, a certain ligand will not be included in the model. The model summary includes information on why and which ligand has not been included.

Oligomeric State Conservation

The quaternary structure annotation of the template is used to model the target sequence in its oligomeric form. The method (Bertoni et al.) is based on a supervised machine learning algorithm, Support Vector Machines (SVM), which combines interface conservation, structural clustering, and other template features to provide a quaternary structure quality estimate (QSQE). The QSQE score is a number between 0 and 1, reflecting the expected accuracy of the interchain contacts for a model built based a given alignment and template. Higher numbers indicate higher reliability. This complements the GMQE score which estimates the accuracy of the tertiary structure of the resulting model.

References

- **BLAST**
  Camacho, C., Coulouris, G., Avagyan, V., Ma, N., Papadopoulos, J., Bealer, K., Madden, T.L. BLAST+: architecture and applications. BMC Bioinformatics 10, 421-430 (2009).
- **HHblits**
  Steinegger, M., Meier, M., Mirdita, M., Vöhringer, H., Haunsberger, S. J., Söding, J. HH-suite3 for fast remote homology detection and deep protein annotation. BMC Bioinformatics 20, 473 (2019).

Table T1:

Primary amino acid sequence for which templates were searched and models were built.

```
MAAQRRSLLQSEQQPSWTDDLPLCHLSGVGSASNRSYSADGKGTESHPPEDSWLKFRSENNCFLYGVFNGYDGNRVTNFVAQRLSAELLLGQLNAEHAEA
DVRRVLLQAFDVVERSFLESIDDALAEKASLQSQLPEGVPQHQLPPQYQKILERLKTLEREISGGAMAVVAVLLNNKLYVANVGTNRALLCKSTVDGLQV
TQLNVDHTTENEDELFRLSQLGLDAGKIKQVGIICGQESTRRIGDYKVKYGYTDIDLLSAAKSKPIIAEPEIHGAQPLDGVTGFLVLMSEGLYKALEAAH
GPGQANQEIAAMIDTEFAKQTSLDAVAQAVVDRVKRIHSDTFASGGERARFCPRHEDMTLLVRNFGYPLGEMSQPTPSPAPAAGGRVYPVSVPYSSAQST
SKTSVTLSLVMPSQGQMVNGAHSASTLDEATPTLTNQSPTLTLQSTNTHTQSSSSSSDGGLFRSRPAHSLPPGEDGRVEPYVDFAEFYRLWSVDHGEQSV
```

VTAP

Table T2:

Template	Seq Identity	Oligo-state	QSQE	Found by	Method	Resolution	Seq Similarity	Coverage	Description
5nzz.2.A	100.00	monomer	-	HHblits	X-ray	2.60Å	0.61	1.00	TGF-beta-activated kinase 1 and MAP3K7-binding protein 1
5nzz.4.A	100.00	monomer	-	HHblits	X-ray	2.60Å	0.61	1.00	TGF-beta-activated kinase 1 and MAP3K7-binding protein 1
5nzz.1.A	100.00	monomer	-	HHblits	X-ray	2.60Å	0.61	1.00	TGF-beta-activated kinase 1 and MAP3K7-binding protein 1
A0A2K6G3T3.1.A	97.39	monomer	-	AFDB search	AlphaFold v2	NA	0.60	0.99	TGF-beta activated kinase 1 (MAP3K7) binding protein 1
5nzz.3.A	100.00	monomer	-	HHblits	X-ray	2.60Å	0.61	1.00	TGF-beta-activated kinase 1 and MAP3K7-binding protein 1
2j4o.1.A	100.00	monomer	-	HHblits	X-ray	2.25Å	0.61	0.79	MITOGEN-ACTIVATED PROTEIN KINASE KINASE KINASE 7-INTERACTING PROTEIN 1
2pom.1.A	100.00	monomer	-	BLAST	X-ray	2.27Å	0.61	0.73	Mitogen-activated protein kinase kinase kinase 7-interacting protein 1
2pom.1.A	100.00	monomer	-	HHblits	X-ray	2.27Å	0.61	0.73	Mitogen-activated protein kinase kinase kinase 7-interacting protein 1
2pop.1.A	100.00	monomer	-	BLAST	X-ray	3.10Å	0.61	0.70	Mitogen-activated protein kinase kinase kinase 7-interacting protein 1

The table above shows the top 9 filtered templates. A further 159 templates were found which were considered to be less suitable for modelling than the filtered list.
1a6q.1.A, 1txo.1.A, 2cm1.1.A, 2eva.1.A, 2i0o.1.A, 2i44.1.A, 2i44.2.A, 2iq1.1.A, 2irm.1.A, 2isn.1.A, 2j82.1.B, 2jfs.1.A, 2jft.1.A, 2p8e.1.A, 2p8e.2.A, 2pk0.1.A, 2pk0.3.A, 2pnq.1.A, 2pop.1.A, 2pop.1.C, 2xzv.1.A, 2y09.1.A, 2yiy.1.A, 3d8k.1.A, 3d8k.1.B, 3d8k.1.C, 3d8k.1.D, 3eq2.1.A, 3eq2.1.B, 3es2.1.A, 3es2.1.B, 3f79.1.A, 3f79.1.B, 3f79.2.A, 3f79.2.B, 3f79.3.A, 3f79.3.B, 3f7a.1.A, 3f7a.1.B, 3fxk.1.A, 3jrq.1.A, 3kb3.1.B, 3kdj.1.B, 3ke6.1.A, 3ke6.2.A, 3mq3.1.A, 3nmn.1.B, 3nmn.2.B, 3nmt.1.B, 3pu9.1.A, 3pu9.1.B, 3rnr.1.A, 3rnr.1.B, 3rt0.1.A, 3rt0.2.A, 3t91.1.A, 3t91.1.B, 3t9q.1.B, 3t9q.1.C, 3ujg.1.B, 3ujk.1.A, 3ujl.1.B, 3w41.1.A, 3w43.1.A, 3w44.1.A, 3w45.1.A, 3zt9.1.A, 4da1.1.A, 4ds8.1.B, 4gs6.1.A, 4jnd.1.A, 4l3p.1.A, 4l52.1.A, 4la7.1.B, 4lg5.1.B, 4lgb.1.B, 4lop.1.B, 4loq.2.B, 4loq.3.B, 4n0g.1.A, 4o91.2.C, 4oic.1.B, 4ra2.1.A, 4raf.1.A, 4rag.1.A, 4yzg.1.A, 4yzg.1.B, 4yzh.1.A, 5d2u.1.A, 5e7r.1.A, 5f1m.1.A, 5gjd.1.A, 5gjg.1.A, 5gwo.1.A, 5gwp.1.A, 5gwp.2.A, 5iti.1.A, 5j7s.1.A, 5j8i.1.A, 5j9l.1.A, 5jga.1.A, 5jh6.1.A, 5jk3.1.A, 5jo1.1.A, 5mn0.1.B, 5mqh.1.A, 5o90.1.B, 5or2.1.B, 5ucg.1.A, 5ucg.1.B, 5ucg.2.A, 5ucg.3.A, 5ucg.3.B, 5v5n.1.A, 5vs5.1.B, 5vsr.1.B, 5vt7.2.A, 5zcg.1.A, 5zcg.2.A, 5zch.1.A, 5zch.2.A, 5zcl.1.A, 5zcu.1.A, 5zcu.2.A, 6ae9.1.A, 6ae9.1.B, 6ak7.1.A, 6b67.1.A, 6b67.2.A, 6b67.3.A, 6ihl.1.A, 6ihr.1.A, 6ihs.1.A, 6iht.1.A, 6ihu.1.A, 6ihv.1.A, 6ihw.1.A, 6jkv.1.A, 6k4e.1.A, 6v0t.1.A, 6zuc.1.B, 7avw.1.B, 7kpr.1.A, 7l4i.1.A, 7l4j.1.A, 7mwn.1.B, 7nti.1.A, 8agg.1.A, 8alk.1.A, 8anp.1.A, 8anp.2.A, 8anp.3.A, 8ay3.1.B, 8ay6.1.B, 8ay9.1.B, 8aya.1.B, 8ey0.1.A, 8gw3.1.A, 8gw3.3.A
